# Third Generation Genome Sequencing Reveals That Endobacteria in Nematophagous Fungi *Esteya vermicola* Contain Multiple Genes Encoding for Nematicidal Proteins

**DOI:** 10.3389/fmicb.2022.842684

**Published:** 2022-05-03

**Authors:** Ruizhen Wang, Leiming Dong, Yuequ Chen, Shuai Wang, Liangjian Qu

**Affiliations:** ^1^Beijing Floriculture Engineering Technology Research Centre, Beijing Botanical Garden, Beijing, China; ^2^The Key Laboratory of Forest Protection of National Forestry and Grassland Administration, Ecology and Nature Conservation Institute, Chinese Academy of Forestry, Beijing, China; ^3^Forestry Resources Protection Institute, Jilin Provincial Academy of Forestry Sciences, Changchun, China; ^4^School of Pharmacy, Liaocheng University, Liaocheng, China

**Keywords:** endobacteria, *Esteya vermicola*, pine wood nematode, nematode-killing capabilities, genome

## Abstract

*Esteya vermicola* is the first recorded endoparasitic nematophagous fungus with high infectivity capacity, attacking the pinewood nematode *Bursaphelenchus xylophilus* which causes pine wilt disease. Endosymbionts are found in the cytoplasm of *E. vermicola* from various geographical areas. We sequenced the genome of endobacteria residing in *E. vermicola* to discover possible biological functions of these widespread endobacteria. Multilocus phylogenetic analyses showed that the endobacteria form a previously unidentified lineage sister to *Phyllobacterium myrsinacearum* species. The number of genes in the endobacterium was 4542, with 87.8% of the proteins having a known function. It contained a high proportion of repetitive sequences, as well as more Acyl-CoA synthetase genes and genes encoding the electron transport chain, compared with compared with plant-associated *P. zundukense* Tri 48 and *P. myrsinacearum* DSM 5893. Thus, this symbiotic bacterium is likely to be more efficient in regulating gene expression and energy release. Furthermore, the endobacteria in nematophagous fungi *Esteya vermicola* contained multiple nematicidal subtilase/subtilisin encoding genes, so it is likely that endobacteria cooperate with the host to kill nematodes.

## Introduction

Pinewood nematode (*Bursaphelenchus xylophilus*) causes pine wilt disease, resulting in severe ecological and economic losses in Asia and Europe (Liou et al., 1999; Vicente et al., 2012; Futai, 2013). *Esteya vermicola* is endoparasitic nematophagous fungus with high infectivity capacity that attacks pinewood nematode. *Esteya vermicola* attracts pine wood nematode through specific volatile compounds (α-pinene, β-pinene, and camphor) (Lin et al., 2013). The lunate adhesive spores of *E. vermicola*, whose concave surface has large surface area and thick adhesive layer, adhere to the cuticle of the attracted nematode and penetrate it. Then *E. vermicola* colonize in the body cavity and absorb the nematode’s nutrient, and produce a large number of mycelia and lunate spores in preparation for next infection cycle (Wang et al., 2011, 2018).

Symbioses have played pivotal roles in biological, ecological, and evolutionary diversification (Archibald, 2014). Various symbiotic microorganisms provide a metabolic capability absent from the eukaryotes host, resulting in increased ecological amplitude and often evolutionary diversification of the eukaryotes host (Douglas, 2014). Symbioses between bacteria and insects have been researched in depth (Ferrari and Vavre, 2011). However, there are few studies on the function and genome of fungal endobacteria.

So far, seven fungal endobacteria genomes have been published, and they are *Candidatus* Glomeribacter gigasporarum colonizing arbuscular mycorrhizal fungi (AMF) *Gigaspora margarita* (Ghignone et al., 2012); *Burkholderia rhizoxinica* colonizing *Rhizopus microsporus*, which participates in the production of rhizoxin (Lackner et al., 2011); a betaproteobacterial endosymbiont associated with *Mortierella elongate* (Fujimura et al., 2014), mycoplasma-related endobacteria (MRE) living in *Rhizophagus clarus*, MRE living in *Racocetra verrucosa*, MRE living in *Claroideoglomus etunicatum* (Naito et al., 2015), MRE living in AMF *Dentiscutata heterogama* (Torres-Cortés et al., 2015).

Based on the sequenced genome of intracellular symbiotic bacteria, intracellular symbiotic bacteria have the following biological functions. *Ca.* G. gigasporarum expresses type II and type III secretion systems and synthesizes vitamin B12, antibiotics- and toxin-resistance molecules, that may contribute to the fungal host’s ecological fitness (Ghignone et al., 2012). *Burkholderia rhizoxinica*, not its host fungus itself, which cause rice seedling blight, biosynthesize rhizoxin to destroy and kill rice (Partida-Martinez and Hertweck, 2005). The betaproteobacterial endosymbiont associated with *Mortierella elongate* encode the insecticidal toxin complex gene (Fujimura et al., 2014). Mycoplasma-related endobacteria genome suggests a high degree of adaptation to the fungal host (Naito et al., 2015; Torres-Cortés et al., 2015).

*Esteya vermicola* from various geographical locations, as endoparasitic nematophagous fungus, hosts endosymbionts, biotrophic endobacteria, in their cytoplasm (Wang et al., 2017). The endobacteria were coccoid, vertically hereditary, as yet uncultured, and essential symbionts (Wang et al., 2017). It was hypothesized that endobacteria play an important biological role in *E. vermicola*. We sequenced the genomes of endobacteria colonizing the *E. vermicola* CBS115803 to elucidate the endobacteria biology associated with nematicidal effect. We were particularly interested in determining the biological relevance of nematode-killing. We presented the annotation of the endobacteria genome draft, phylogenetic placement based on single copy genes, energy generation abilities, coevolution implications for nematode-killing abilities. Overall, the endobacteria possibly exhibit a high adaptability to the fungal host for nematode-killing capabilities, showing the significance of endobacteria in the biology of *E. vermicola*.

## Materials and Methods

### Biological Material and DNA Extraction

To acquire mycelium, *E. vermicola* CBS115803 was cultured in Potato sucrose medium. The Retsch Mixer Mill MM 400 was used to grind the mycelium at low temperature in order to break fungal cells and release intracellular symbiotic bacteria. The grounded mycelium were washed out with sterile 0.25M sucrose solution at 4°C. To remove ungrounded mycelium and a portion of the host’s fungal nucleus, the mixture was centrifuged at 1200g. Then the supernatant was centrifuged with an angle rotor at 16000g for 30 min to obtain symbiotic bacteria. Endobacterial DNA was extracted using 8M sterile guanidine hydrochloride after residual host DNA was removed with Sigma benzonase nuclease (Merck KGaA, Germany). For genome sequencing, 15 μg of DNA were used.

### DNA Sequencing and Sequence Assembly

Genomic DNA was fragmented using G-tubes (Covaris) and then end-repaired to prepare SMRTbell DNA template libraries (fragment sizes > 10 kb selected using a bluepippin system) according to the manufacturer’s instructions (Pacific Biosciences). Library quality was assessed by Qubit 4 Fluorometer (Life Technologies, Singapore), and average fragment size was estimated using a Agilent 2100 Bioanalyzer (Agilent, United States). SMRT sequencing was carried out with a Pacific Biosciences Sequel II sequencer (Pacbio, United States) at Frasergen Bioinformatics Co., Ltd, Wuhan, China.

The PacBio reads were *de novo* assembled using the Canu (v2.0) software (Koren et al., 2017). The depth of genome coverage were determined by pbalign tool (BLASR, v0.4.1) (Chaisson and Tesler, 2012). The sequencing data/the raw sequencing reads in this article have been deposited in the CNSA database^1^ (project ID: CNP0002484). For Benchmarking Universal Single-Copy Orthologs (BUSCO) analysis (Simão et al., 2015), version 5.2.2 was used. The lineage dataset was alphaproteobacteria_odb10 (Creation date: 2020-03-06, number of genomes: 744, number of BUSCOs: 432).

### Genome Annotation

The genome was annotated using Glimmer (v3.02) (Delcher et al., 2007). The tRNA and rRNA genes were identified by tRNAscan-SE (v2.0) (Lowe and Eddy, 1996) and RNAmmer (v1.2) (Lagesen et al., 2007), respectively. The functional characterization of the identified genes was achieved by BLASTP with an E-value threshold of 1e-5 against the databases of the NCBI Non-Redundant protein database (NR), Swiss-Prot, Clusters of Orthologous Groups (COG), Kyoto Encyclopedia of Genes and Genomes (KEGG), and Gene Ontology (GO) (Buchfink et al., 2015). The highest-scoring match was chosen as the final annotation for each gene. Tandem repeats finder and MISA-web were used to predict tandem repeats and simple sequence repeats (SSR) sequences in the genome, respectively (Benson, 1999; Beier et al., 2017). The predicted protein sequences of each genome were compared with each other, and protein alignment was performed using blastp (Altschul et al., 1990). OrthoMCL software was used to cluster and count the clustering results (Chen, 2006). The blast Ring Image Generator (BRIG) (Alikhan et al., 2011) was used to visualize the multiple copy genes incorporation in the genome of the E. vermicola symbiont being the reference, in comparison with the closest Phylobacterium genomes. Signal peptide prediction tool signalp (v4.1) was used to annotate whether the protein sequence was a secretory protein (Parameters: - t gram + - f summary) (Nielsen, 2017).

### Phylogenetic Analyses

Eight hundred and seventy-nine single copy genes for multilocus analyses were identified from 29 bacteria for the multilocus phylogenetic reconstruction. We used BUSCO v2.0 software to assess the integrity of 29 genomes (Simão et al., 2015). Multiple sequence alignment of single copy genes was accomplished by MUSCLE (v3.8.31) (default parameter) (Edgar, 2004). The maximum likelihood tree was constructed with RAxML (v8.2.12) (Stamatakis, 2014) using the automated protein model assignment algorithm and a gamma model of rate heterogeneity (-m PROTGAMMAAUTO) (Carroll et al., 2019; Sawada et al., 2021), based on concatenated 879 amino acid sequences from single copy genes of endobacteria and close relatives within the genus *Phyllobacterium* (Supplementary Data Sheet 1). The genes concatenated were shown in supporting information (genome_SingleCopy.phylip). 100 bootstrap replicates was used to produce the phylogenetic tree using amino acids sequence. Bootstrap values were given at each branch.

To infer the evolutionary history of subtilases/peptidase S8, maximum likelihood method and a JTT matrix-based model was used. The JTT model was based on the assumption that the amino acid frequencies are in equilibrium and remain the same throughout the evolutionary process (Jones et al., 1992). The subtilases/peptidase S8 genes used were shown in supporting information (Supplementary Data Sheet 2). The evolutionary history of the taxa studied is represented by a bootstrap consensus tree generated from 1000 replicates (Felsenstein, 1985). The tree with the highest log likelihood (-26864.23) is shown. Initial tree(s) for the heuristic search were obtained automatically by applying Neighbor-Joining and BioNJ algorithms to a matrix of pairwise distances predicted using the JTT model, and then selecting the topology with the highest log likelihood value. A discrete Gamma distribution was used to model evolutionary rate differences among sites [5 categories (+ G, parameter = 4.1038)]. The rate variation model allowed for some sites to be evolutionarily invariable [(+ I), 0.00% sites]. Evolutionary analyses were conducted in MEGA X (Kumar et al., 2018).

## Results

### Genome Sequencing, and General Features of the Genome

**FIGURE 1:**
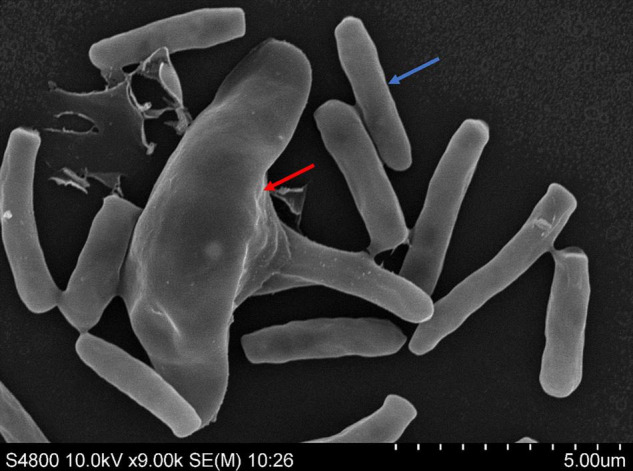
Lunate spores (red arrow) and rod shaped spores (blue arrow) of *Esteya vermicola* CBS 115803 were used to produce mycelium for obtaining endobacteria.

The lunate spores and rod shaped spores were used to culture mycelium of *Esteya vermicola* CBS115803. Then the mycelium were used to purify intracellular symbiotic bacteria. The spores were observed by scanning electron microscopy to determine whether there were epiphytic bacteria or contaminated bacteria on the surface of the fungus. As can be seen from the image (Figure 1), there were no cell-surface bacteria of *E. vermicola*.

To increase the DNA proportion of endobacteria, nucleases (sigma, Benzonase E1014) were used to degrade host DNA. The third generation pacbio sequencing method was used to improve the read length and assembly accuracy of the reads. The proportion of symbiotic bacteria DNA data increased from 0.1 to 3%. Before filtering the host sequence, the entire sequencing amount was 22 Gb, with 0.664 Gb of bacterial data remaining (3% proportion relative host DNA content). The length of contigs ranged from 1 to 86 Kb. The average read depth was 129.12×, and the estimated read depth in genome ranged from 4.87 to 9793.60×. The coverage was from 99.74 to 100%. The endobacteria genome had a 58.93% guanine-cytosine (GC) content, according to the metagenomic assemblies. Furthermore, the genome size was about 3.96 Mb based on the number of contigs, and the number of annotated genes was 4542 (Supplementary Tables 1, 2), accounting for 74.18% of the genome. Total 86.79% genes were annotated (Supplementary Table 3).

### Evolutionary Placement

**FIGURE 2:**
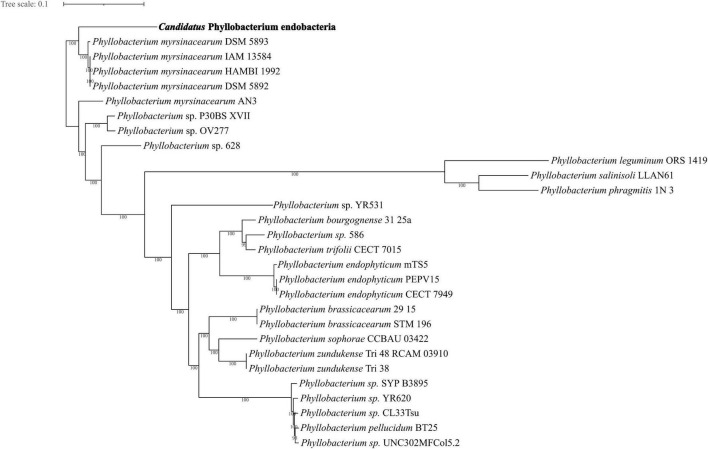
The maximum likelihood tree based on concatenated 879 amino acid sequences from single copy genes of endobacteria and close relatives within the genus *Phyllobacterium* was inferred using the automated protein model assignment algorithm and a gamma model of rate heterogeneity (-m PROTGAMMAAUTO). 100 bootstrap replicates were used to produce the phylogenetic tree using amino acids sequence. Bootstrap values were given at each branch.

The majority of the endobacteria sequences were identified as the genus *Phyllobacterium* according to nucleotide and protein database. A total of 879 single-copy genes found for 29 genomes including 28 genomes of *Phyllobacterium* and the endobacteria in *E. vermicola*. To determine the phylogenetic placement of the endobacteria, a maximum likelihood phylogenetic analysis of amino acid sequences from 879 single copy genes were conducted. Genome features, background information and BUSCO assessment are shown in Supplementary Tables 4, 5. The endobacteria was firmly placed in the branch of *P. myrsinacearum* (Figure 2). As a result, the symbiotic bacterium was most closely related to *P. myrsinacearum* species. The symbiotic bacteria clustered with *P. myrsinacearum* species and unknown *Phyllobacterium* species, according to the phylogenetic tree constructed by using the conserved atpD and recA (Supplementary Figure 1), and again showed that the endobacteria and *P. myrsinacearum* were the closest relationship.

### Gene Family Analysis

**FIGURE 3:**
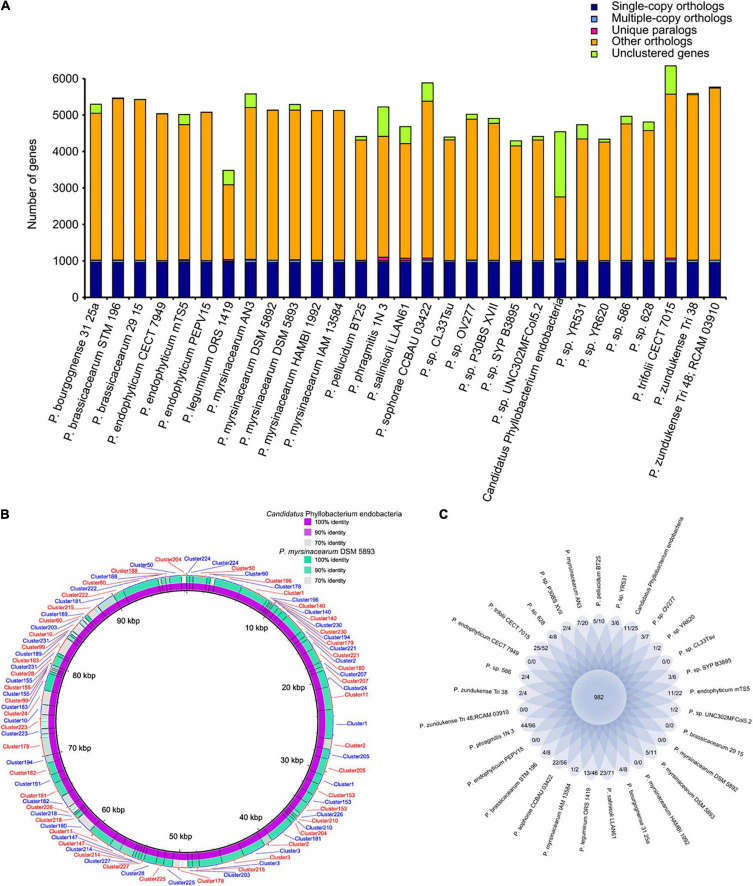
Gene family of the endobacteria living in *Esteya vermicola* and its 28 related species. **(A)** Statistical graph of homologous gene number of the endobacteria and 28 completely sequenced *Phyllobacterium*. Single-copy orthologs, single copy homologous genes in the gene families shared among species; multiple-copy orthologs, multicopy homologous genes in gene families shared among species; unique paralogs, genes of the strain unique to the family; other orthologs, all other genes; unclustered genes, genes not clustered into any family. **(B)** BLAST ring image of multiple copy genes in the genome of the endobacteria living in *E. vermicola*, in comparison with the closest *Phylobacterium* genomes - *P. myrsinacearum* DSM 5893, indicating the endobacteria having more multiple copy gene family (42). **(C)** Flower plots showing the common gene family number (in the center) and strain-specific gene family number/gene number within strain-specific gene families (in the petals) in the endobacteria living in *E. vermicola* and its 28 related *Phyllobacterium* species.

The genomes of some bacterial pathogens have gained genes through gene duplication, resulting in increased numbers of key gene clusters or the expansion of important protein families (Cole et al., 1998; Pallen and Wren, 2007). Gene duplication and protein family expansion are important genomic mechanisms that shape the evolution of pathogenic fungi (Powell et al., 2008). Similarly, gene duplication in bacteria facilitates adaptation to changing environmental conditions, which affects the fitness and immunogenic properties of many bacterial pathogens (Craven and Neidle, 2007; Ahmed et al., 2008). To investigate the features presented in the endobacteria, a comparative gene family analysis against 28 completely sequenced *Phyllobacterium* genomes was performed. The 29 genomes yielded a total of 11,332 gene families. Focusing on the endobacteria genome in *E. vermicola*, 4542 genes were categorized into 2637 families. Interestingly, among the 29 *Phyllobacterium* genomes, multiple copy gene families and multiple copy genes in the endobacteria were the most abundant (Figure 3A and Supplementary Table 6). Multiple copy genes in the genome of the endobacteria living in *E. vermicola*, in comparison with its the closest *P. myrsinacearum* DSM 5893, indicated that the endobacteria had more multiple copy gene family (42) and multiple copy genes (91) (Figure 3B and Supplementary Table 6). These genes are mainly involved in amino acid metabolism, carbohydrate metabolism, membrane transport, nucleotide metabolism, replication and repair. Nucleotide excision repair and cell cycle were the specific metabolic pathways of multiple copy gene family in the symbiotic bacteria, which were distinct from those of other free-living bacteria (Supplementary Tables 6, 7), which metabolic pathways could be related to the ecological adaptability of the symbiotic bacteria in fungal cells. Eleven gene families (25 genes) of the endobacteria were unique (Figure 3C and Supplementary Table 8), mainly involved in the synthesis of chemotaxis protein and mobility protein.

### High Proportion of Repetitive Sequences

Tandem repeat may act as an engine in genetic variability and bacterial adaptation, as pointed out by accumulating evidence (Zhou et al., 2014). Repetitive sequences may improve the ability to regulate gene expression, also important for bacterial adaptation and increasing their phenotypic plasticity (Zhou et al., 2014). Repeat sequence analysis was performed in order to better understand the genomic structure. It was showed that the endobacteria genome existed 235 short tandem repeats (STRs), which total length was 61839 bp, accounting for 1.56% of the genome. There were 98 genes involved in the 235 STRs. Intragenic STRs were found more frequently near the termini (45% at 5′ end, and 55% at 3′ end) (Supplementary Tables 9, 10). Within the annotated genes, there are 35 STRs involving 29 genes. Two-component system |Biofilm formation (ko02020| ko02025), beta-Lactam resistance |Cationic antimicrobial peptide (CAMP) resistance (ko01501| ko01503), Bacterial secretion system (ko03070), base/nucleotide excision repair (ko03410/ko03420), Quorum sensing |Protein export| Bacterial secretion system (ko02024| ko03060| ko03070), RNA degradation| Longevity regulating pathway (ko03018| ko04212), arginine and proline metabolism (ko00330) were among the involved metabolic pathways. There are multiple genes including more than two STRs, of which the functions involved are chemosensory pili system protein (ChpA), type VI secretion system secreted protein (VgrG), and membrane fusion protein (acrA, mexA, adeI, smeD, mtrC, cmeA). *P. zundukense* (Accession numbers: GCF_002764115.1), on the other hand, had only 98 STRs in its genome, involving in 72 genes. The total length was 8,395 bp, accounting for only 0.14% of the genome. Half number of STRs was intragenic, which were found more frequently near the termini (50% at 5′ end, and 50% at 3′ end) (Supplementary Tables 9, 10). Also, *P. myrsinacearum* (Accession numbers: GCF_004216655.1) had only 92 STRs (13,637 bp), accounting for only 0.24% of the genome, involving in 69 genes. 64% out of the 92 STRs was intragenic, which were also found more frequently near the termini (48% at 5′ end, and 52% at 3′ end).

Specially, both SSRs (CGC)5 and STR (5′-GGGGAGAG GGGGGAGAGGGGGGAGA-3′) was discovered in 3′ end of the *luxR* family gene (bacterial regulatory) of the endobacteria, which regulates motility, synthesis of virulence determinants and biofilm architecture (Chen and Xie, 2011). The gene is highly regulated and could play a key role in a flexible response to the changing environment conditions.

### Environmental Adaptation Pathway

Intriguingly, there were 8 Acyl-CoA synthetase genes in oxidative phosphorylation of metabolic pathway (ko04714) of the endobacteria. Only two Acyl-CoA synthetase genes were found in *P. zundukense*, and one gene was found in *P. myrsinacearum*, both of which were closely related to the endobacteria genome. The flow of electrons through the electron transport chain is an exergonic process. The energy from the redox reactions creates an electrochemical proton gradient, which drives the synthesis of adenosine triphosphate (ATP). The endobacteria genome had ten genes encoding electron transport chains, including *ctaA* (1), *ctaG* (2), *fbcH* (1), *petA* (2), *petB* (3), *petC* (1). While there were only six and five those genes in *P. zundukense* and *P. myrsinacearum*, respectively. The endobacteria possessed QseC gene, which is involved in metabolic pathway of two-component system. QseC is a bacterial adrenergic receptor that activates virulence genes in response to interkingdom cross-signaling (Clarke et al., 2006). Furthermore, the endobacteria had great potential to use trehalose, with three neutral trehalase and four trehalose consumption proteins in its genome. In contrast, there was only one such gene in the genomes of the two closely related bacteria, *P. zundukense* and *P. myrsinacearum*. The role of trehalose in symbiosis is to enhance abiotic stress tolerance (Sharma et al., 2020).

### Endobacteria Genome in Nematophagous Fungi *Esteya vermicola* Contain Genes Encoding Nematicidal Proteins

**FIGURE 4:**
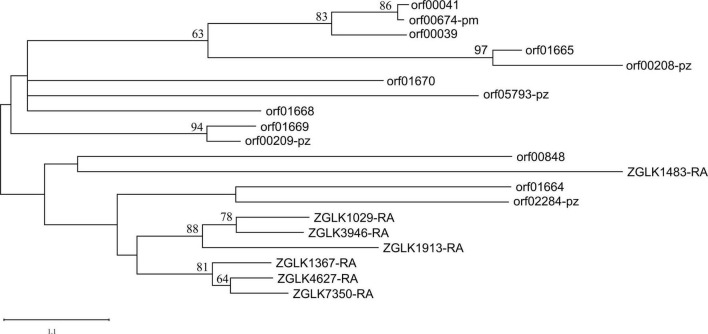
Phylogenetic placement of the endobacteria inferred by maximum likelihood phylogenetic analysis based on 20 Subtilases/peptidase S8 amino acid sequences (ZGLK, fungi gene; orf, bacteria gene). The 20 genes used for the phylogenetic analysis are listed in Supplementary Table 12.

Subtilases/peptidase S8 and subtilisin-like were prevalent in nematophagous fungi and played a role in nematode lethality and infection process (Wang et al., 2018). Surprisingly, there were eight subtilase/subtilisin genes in the genome of endosymbiotic bacteria, five of which could be transported outside the endobacteria cell (Supplementary Table 11). The endobacteria had more subtilase/subtilisin genes than closely related *P. zundukense* and *P. myrsinacearum*, which had four and one genes, respectively. We used subtilases/peptidase S8 genes in the endobacteria (eight genes) and its fungal host *E. verimicola* (seven genes), *P. zundukense* (four genes) and *P. myrsinacearum* (one gene) (Supplementary Table 12) to construct a phylogenetic tree to explore their genetic and evolutionary relationships. Almost all the subtilases/peptidase S8 genes in the fungi *E. vermicola* were found to be clustered together, with the exception of one gene, ZGLK1483-RA, which encodes a secretory protein. Almost all of the subtilases/peptidase S8 genes from bacteria were clustered together (Figure 4). According to the phylogenetic tree, the bacteria’s subtilases/peptidase S8 genes originated earlier. Four subtilases/peptidase S8 which are typically virulence factors, were found to be autotransporter associated with infection or virulence in pathogenic bacteria (Henderson and Nataro, 2001; Nishimura et al., 2010). Endobacteria genome in nematophagous fungi *E. vermicola* contain multiple nematicidal genes, which is most likely the outcome of coevolution, adapting to its niche and incorporates with the fungal host to kill nematodes.

## Discussion

### The First Identification of *Candidatus Phyllobacterium* Endobacteria in the Cells of the Nematophagous Fungi *Esteya vermicola*

According to blast against nucleotide and protein database, 282 of 306 contigs of the genome can match with known sequences. The genus *Phyllobacterium* was the closest blast matches (121 of the 282 sequences), and *Acidovorax* was the second closest blast matches (17 of the 282 sequences) (Supplementary Tables 13, 14). Therefore, the endosymbiotic bacteria were preliminarily identified as this genus *Candidatus Phyllobacterium*, so the genomes of this genus *Phyllobacterium* was used to construct phylogenetic tree. However, the 16S gene was not obtained by genome sequencing and assembly. Moreover, this preliminary genome-based identification result was different from the previous 16S identification result (Wang et al., 2017). The following are some plausible explanations: 1. More than one kind of endobacteria exists in fungal cells. 2. The endobacteria *Candidatus Phyllobacterium* may be enriched in this medium. The abundance and the thickness of cell wall of the endobacterium may alter when the culture medium changed.

*Phyllobacterium* have been found as free-living bacteria in soil, in water and associated with unicellular organisms, and *Phyllobacterium* strains were mainly isolated from plant rhizosphere and root nodules. Several strains are capable of communicating with plant tissues and promoting plant growth (Mantelin et al., 2006; Flores-Félix et al., 2013; Sánchez et al., 2014). For the first time, we discovered this endobacteria in the cells of the nematophagous fungi *E. vermicola*.

### Genome Assembly Integrity

That 16S rRNA gene and other conserved (*ftsZ*, *atpD* and *recA*) gene absent or not was researched in endobacteria of 8 fungal endobacteria and 1 insect endobacterium (Table 1). Only 2 fungal and 1 insect endobacteria genome had one single copy 16S rRNA gene. Cell division protein (ftsZ) were absent in four MRE genome, consistent with Kuo’ commentary (Kuo, 2015). *AtpD* gene, which encodes ATP synthase subunit beta, was not included in the assembled genome of the 7 endobacteria of fungi, except that of endobacteria of *E. vermicola* and *Wolbachia* endosymbiont. It is inferred that ATP may be provided by their host fungi. In contrast, *recA* gene, essential for the repair and maintenance of DNA, exists in all seven symbiotic bacteria and *Wolbachia* endosymbiont, except MRE living in the AMF *D. heterogama*, and this may be due to assembly problems, resulting in the deletion of the gene. These findings suggest that the cell functioning and proliferation of endobacteria may be under the control of their fungal hosts, that is likely the outcome of long-term coevolution (Kuo, 2015).

**TABLE 1:** Presence or absence of conserved genes in endobacteria of fungi (8) and insect (1).

Assembly Accession	Species	16S rRNA	ftsZ	atpD	recA	ispA	mreB	rodA
project ID: CNP0002484	*Candidatus* Phyllobacterium (alphaproteobacteria) living in *E. vermicola*.	none	2	1	2	1	none	none
GCA_002888635.1	MRE living in the AMF *Dentiscutata heterogama* (mycoplasmatales)	none	none	none	none	none	none	none
GCA_001017705.1	Mycoplasmataceae bacterium RC_NB112A (mycoplasmatales)	none	none	none	1	none	none	none
GCA_001017675.1	Mycoplasmataceae bacterium CE_OT135 (mycoplasmatales)	none	none	none	1	none	none	none
GCA_001017685.1	Mycoplasmataceae bacterium RV_VA103A (mycoplasmatales)	none	none	none	1	none	none	none
GCA_000775775.1	Bacterium endosymbiont of *Mortierella elongata* FMR23-6 (betaproteobacterial)	1	1	none	1	none	1	1
GCA_000227585.1	*Candidatus* Glomeribacter gigasporarum (Betaproteobacteria)	1	1	none	1	1	1	1
GCA_000198775.1	*Mycetohabitans rhizoxinica* living in *Rhizopus microsporus* (Burkholderiaceae)	none	1	none	1	none	2	1
GCF_013458815.1	*Wolbachia* endosymbiont of *Diaphorina citri* (a-proteobacteria; alphaproteobacteria)	1	1	1	1	none	none	1

*ftsZ, ispA, mreB, rodA: rod shape determining gene.*

The genome assembly integrity of the endobacteria was 43% (BUSCO, bacteria_odb10), which was higher than that of the other four MRE genomes (Supplementary Table 15). Nevertheless, all five genomes were assembled in fragments. Genomes of *B. rhizoxinica*, *Ca.* G. gigasporarum, endobacteria of *Mortierella elongata* FMR23-6 had a better assembly results (BUSCO > 85%). Because *B. rhizoxinica* can be cultured artificially (Partida-Martinez and Hertweck, 2005). While *Ca.* G. gigasporarum and endobacteria of *Mortierella elongata* FMR23-6 were closely related, both belonging to the family Burkholderiaceae (Sharmin et al., 2018), and their cell size was larger than that of other endobacteria. Further, the genome of insect *Wolbachia* endosymbiont assembled the completion map (PRJNA544530). Only endobacteria *Ca.* G. gigasporarum has all four rod shape determining gene *ftsZ*, *ispA*, *mreB*, *rodA*, which is consistent with its rod-shaped morphological characteristics (Jargeat et al., 2004).

### No α- and β-Pinene Monoterpenoid Synthase-Encoding Genes in This Version Genome

By phylogenetic analysis of terpene synthase from bacteria and fungi, it imply horizontal gene transfer from bacteria to fungi, of which the majority are *Metarhizium* species (Jia, 2016). Our previous results showed that the presence of pine wood nematode but not contact with *E. vermicola* would induce the fungus to produce more lunate spores that prey on nematode (Wang et al., unpublished). Meanwhile, *E. vermicola* can stimulate activity of pine wood nematode under untouched conditions (Wang et al., unpublished). So *E. vermicola* and pine wood nematode communicate with each other by volatile compounds. *Esteya vermicola* attracts pine wood nematode through volatile monoterpene compounds, α-pinene, β-pinene, and camphor (Lin et al., 2013). However, α- and β-pinene monoterpenoid synthase-encoding genes were absent in *E. vermicola* genome (almost completely assembled, 42 scaffolds without gap), so it was inferred that endobacteria produced the monoterpene that attracted pine wood nematode (Wang et al., 2018). Simultaneously, no monoterpene synthase gene was identified in the assembled version of the endobacterium genome, which was highly possible due to the low integrity of the assembled genome and the annotation. Assuming that the endobacterium produces volatile compounds that attract pine wood nematodes, the endobacteria probably mobilize host fungi to kill nematodes through volatile compounds, although different from free-living *Stenotrophomonas maltophilia*, which used urea metabolite ammonia to function as a signal molecule in *Arthrobotrys oligospora* to initiate the lifestyle switch to form trap structures against nematodes (Wang et al., 2014).

### Possible Significance of Highly Repetitive Sequences

In comparison to closed related species, the endobacteria had more repeats sequence. Bacteria can use tandem repeats to adapt to changing environments by reversibly shutting down or modulating the function of specific genes. Tandem repeats variations have been shown to have a role of in bacterial adaptation strategies, such as immune evasion, tissue tropism and the modulation of environmental stress tolerance, and tandem repeats and their variability provide regulatory strategy for adaptability (Zhou et al., 2014). In general, variable tandem repeats tend to localize in flexible genes involved in pathogens invasion (Moxon et al., 2006; Guo and Mrázek, 2008). Based on the above, we inferred that the repetitive sequences of endosymbiotic bacteria may be used to regulate genes about pathogens invasion.

### The Endobacteria Possibly Cooperate With Host to Kill Nematodes

Subtilase can degrade nematode and insect body walls (Lu et al., 2020). According to the phylogenetic tree, the diversity of subtilase was higher in bacteria than it was in fungi. The secretory subtilase in symbiotic bacteria and in their host fungi may work together to infiltrate the cuticle of pine wood nematode, enhancing the efficiency of killing the nematodes. A better understanding of its endobacteria may aid in finding solutions for use of the nematophagous fungi. We point out the probable synergistic function of endobacteria and its fungal host, in order to guide future research toward a deeper analysis of the importance of these endobacteria for the fungal life cycle and killing pine wood nematode.

## Data Availability Statement

The datasets presented in this study can be found in online repositories. The sequencing data/the raw sequencing reads in this article have been deposited in the CNSA database, https://db.cngb.org/(project ID: CNP0002484).

## Author Contributions

RW designed research and wrote the manuscript. LD analyzed data and modified the manuscript. YC and SW performed research. LQ designed research and modified the manuscript. All authors contributed to the article and approved the submitted version.

## Conflict of Interest

The authors declare that the research was conducted in the absence of any commercial or financial relationships that could be construed as a potential conflict of interest.

## Publisher’s Note

All claims expressed in this article are solely those of the authors and do not necessarily represent those of their affiliated organizations, or those of the publisher, the editors and the reviewers. Any product that may be evaluated in this article, or claim that may be made by its manufacturer, is not guaranteed or endorsed by the publisher.

## Footnotes

1
https://db.cngb.org/
