# Supplementary material for: Third Generation Genome Sequencing Reveals That Endobacteria in Nematophagous Fungi Esteya vermicola Contain Multiple Genes Encoding for Nematicidal Proteins
Source: Front Microbiol. 2022 May 3;13:842684. doi: 10.3389/fmicb.2022.842684 (PMC9111515; doi:10.3389/fmicb.2022.842684)
Supplement: Supplementary file 1 [file Data_Sheet_1.docx]

29 287513

ASM301095v1 MRLTRQTNYAIRILMYCAANDGNLSRIGEIAQAYTVSELFLFKILQPLVENGFIETVRGRNGGVKLGKPAKDITLFDVVRVTEENFAMAECFEND-AADCPLIDSCGLNAALRKALNAFFEVLADYTIEDIVDKR-DVRSLLGIDVLEKRLATASMAGHSIPHFQNSAGHATISIGVKEFMCVGANPPFDHPHVFLDLGDDNEKVCPYCSTLYRYDASLAADATV-PEGCTYVD---RAT--------MKKWMNTVAAAAMLLGTATMARAA--ENVEVLHWWTSGGEATALEVLKKDLESKGISWSDMPVSGGGGTEAMTVLRARVTAGNPPTAVQMLGFDILDWAKQGALGNLDDIAAKEGWDAVIPTALQGFSKYDGHWIAAPVNVHSTNWIWINKAALDKAGGKEPANWDELIALLDNFKAQGITPIAHGGQPWQDATIFDAVVLSLGNDFYTAAFIDLDPKALGSDKMKEAFDRMTKLRSYVDDNFSGRDWNLASAMVIENKAGLQFMGDWAKGEFVKAKKEPGKDFVCMRFPGTQGSVTFNSDQFAMFKVSDAQRAPQLVMASAIESPTFQSAFNVVKGSAPARTDVPDTDFDACGKKAIKDLAEANKAGTLYGSMAHGHANPAAVKNAIYDVVTRQFNGELSSEDAVKELVSAVDAAKMTGTTRINGSETPLLEASKLTKIFGTLKACDEINLTIGTGEIHSLLGENGAGKSTLVKMLFGALQPTSGDIAWKGQSVTVSNPAMGRSLGIGMVFQHFSLFDALTAAENIALSLDEKTPLDTIAARAKEIGQTYGLPVDPEALVGDLSVGERQRIEIIRCLLQNPDLIILDEPTSVLTPQEADLLFVTLERLRSEGKSILYISHRLEEVRRLCDRATVLRHGKVVGQCDPRAETTASLARMMVGSDIHIIDRHPVEASAPAQAALIEVKGLSQKPRGPFSVSLHDIHLAVRSGEILGIAGVAGNGQGELFEAISGEVLQERPDIIRIRGTDAGRIGISARRKLGAAFVPEERLGHGAVPDMSLTNNLLLSRHSTDRKVFLTGGTLKLVAEASLASAAKRIVTQMDVRKSAENPDASALSGGNLQKFLIGRELDRNPSVMIVNQPTWGVDAGAAAHIRQALVDLARSGSAVIVISQDLDELFEISDRIAAMVHGRLSESVPIEAMSRERVGLMMGGVDHSTALAN----AGAA--MVAPPIPVSVLTGFLGSGKTTLLNRLLKDPALADTAVIINEFGDVGIDHLLVEKASEGIIELSDGCLCCTVRGELVDTLADLIDRLQTGKLQGLKRVIIETTGLADPAPVLHAIMGHPVLLQAFRVDGVITTVDAVNGMATLDAHEEAVKQAAVADRIVITKTDLPESVSNLHSLQARLRALNPGADLIDVSDQLTGYAALFECGVYNPGTKTIDVQRWLKAEAYRDREQERNAIDGH--DH-HDHDHGGH----------DDHHHHDVNRHDASIRSFSLKHDAPVPLSTFDMFLDLLRSAHGEKLLRVKGIVQLADDPERPLVIHGVQHIFHPPARLAAWPDGVRETRLVMIVKDLPESYVTQLFNAFLG-QPQIDTPDRTALFDNPLAISGVKLG----------MHRVVPGLTKSSASDSSESVSADSVNSVEVIAPNFKRELSGVTSTIIQLIPLQRANGLNIVTMGPGLPAALPSIGWMSVPALWFKPRTRPFRIWHARRNNEMIAGILMRSVLRMRLKLLFTSAAQRDHKPFTKWLIRQMNAVIATSGKSGSFLKVPHTVIMHGVDTDLFHPPQDEQDRFAASGLPGELLVGCSGRIRPSKGTDLFVDAMINLLPQHPRWTAVMTGRTTTENKGFEQALRDKIAQAGLADRILFLGEVPDVRLWYRRMTLYVAPSRNEGFGLTPLEAMASQTAVVASDAGAYAEMIEAG-TGTVVTAGDGAALEAAIAPYLADPAMCESAGRSALAHVRENFPLQKEAVKILEVYENMFDSGSKL-------------------MARQSTRKVASP-LRNLRALLAGAS-SALPFLVTFTPHARAEDALGRINTPFGVEVGTFEVIQFAMFLGAMGAALLSAGWLIRERSRIAHENRQLRDKVADLNVTVQRNEAMLNLKDQRIIVWEGASSTPNLIGSLPAEIGVPEARALFLAFGRWLQIDSATLLDRSISALREKARPFDIAIETSKGAPLEVQGRTSGGYAIVRFINLGEARAAQALLKTENYQLNEAVGALRGLLESIDMPAWSRDGDGRLTWVNMAYAKAVDVRDPRAAISEARELFGVQARERIERDKAANRRFHDQLSTVVGGDRHVFRVTDAAGSAGSAGLATDISEIELIREEFKRTVLNHSDTLDQLNSAVAMFDANRKLEFFNQAFAKLWGLDTAFLDSNPDNAMLLDRFRSDGILPEQPDWRRWKETVLSAYHSVESQEHIWYLTDGRTLRVIVNPQPKGGVTWVFENLTEKLELQSRYNTLIKVQGQTLDHLAEGVAVFGSDGKIRLSNPAFATLWSLKREQIAEGMHISAIRRLCEPVSDVAHWENFVTTVTGFDDERQSLSGHIELSTGKILFYATAPLPNGQTMLTFVDVTDSVQVERALKEKNEALERTDELKNDFVQHVSYELRSPLTNIIGFTELLQTPMTGPLNDRQRDYLDHIGSSSSVLLTIVNDILDLATVDAGIMELDISDVSVTDAVTTASEKVAERLKEHNILLDIRVAPGSGSFRADASRVKQVLANLLSNAANYAPEGSTVTLACWREQAEMIFSVQDHGPGMPDYVLNSIFKRFQPYPNGGRKRGAGLGLSIVKGFVELHGGTVDIDSAAGKGTLVTCRFPLEARTYRIAAEMREYYSITELTREFGVSTRTLRFYEDEGLIHPVRRGRTRLFRPSDRHLLKQILRGKRLGFSIAEIHEIVQMYNEPPGEMGQLHLLMKRVEEKRADLRQKRRDIEETLTELDQVEEACIERLAELGVNTMEYFIQQSINGLTLGSIYGLIAIGYTMVYGIIGMINFAHGDVFMLGAFMAMIVFLALATFIGGVPVVLALL----LMMIVAMLLTGLWSWTIERLAYRPLRGSFRLAPLITAIGMSIALSNFVQVTQGPRNKPIPPLVNGGITIYGTSITIAYKQMVIIIVTAILLAAFWYLVNKTTLGRAQRSCEQDRKMAALLGVDVDRTISMTFVMGAMLAAVAGTLYLMFYGVISFTDGFVPGVKAFTAAVLGGIGSLPGAVVGGLLIGLIEALWSAYFSIDYKDVAAFSILAIVLIFLPSGILGRPEVEKVMDDNNDLFSRIVKTARERQAAAPQTRPESA--------PVA-------------TVRTVPSPAAKAAPRSAPESSGS-YSAADIEVLEGLEPVRRRPGMYIGGTDERALHHLFAEVIDNSMDEAVAGHANFIDIDLEENGYLSVTDNGRGMPVDPHPKFK--DKSALEVIMTMLHAGGKFDSKVYETSGGLHGVGVSVVNALSDHVEVEVARNRRLYRQHFSRGKALGPLEEVGEVQNRRGTRVTFHPDAEIFGHAAKFDPARLYKMARSKAYLFGGVEIRWSCAPSLLDPKDPTPDKAVFHFPGGLKDYLAASLGKEFQVTREMFAGKTEKSGGHGALEWAVAWHGGDGFVHSYCNTIPTGEGGTHEAGLRIALTRGLKAYAELTGNKRASIITTDDVMISSAAMLSVFIREPEFVGQTKDKLATVEAQRIVENAIRDPFDHWLAASPQEASKLLDWVVDRAEERVKRRQEKEVSRKSAVKKLRLPGKLADCSQSGALGAELFIVEGDSAGGSAKQARDRATQAVLPLRGKILNVVSAGREKMTANQQISDLIQALGCGTRSKYRDEDLRYDRVIIMTDADVDGAHIASLLITFFYQEMPELIRNGHLFLGVPPLYRLAQGGKVAYARNDAHKDELLKTMFTGKGKIEIGRFKGLGEMRADQLKETTMDRKKRTLLRVQIDEEWANETKLAVDDLMGTRPDARFRFIQDRAAFVEELDI--MKEIVVVGAGKIGGTIANLLAG--------TGDYHVTVVDRAQAQLDALDVSPLVATKQIEIAEAGALEQVLDGKFAVLSAAPFHLTTRIAEAAAKSGVHYLDLTEDVASTRIVKQLSAKATTAFIPQCGLAPGFISIVANDLAKRFDSLESVRMRVGALPQYPSNALNYNLTWSTDGVINEYCEPCEAIVDGELTEVPPLEEREEFSLDGVTYEAFNTSGGLGTLCETLLGKVRTLNYRTIRYPGHAAIMKALLNDLGLRDRRDVFKDILENALPSTLQDVVVIFVTVSGRKKGRLLQETYANKVYSNHIGAQLYSGIQITTASAICAVLDMLAKGDLPQQGFIRQEDIALDAFLANRFGKAYAQAESAGRLVA----------------------------------MPAISVQSPGDRIRGFASENPALLFVLLYFAFQIFFLTTISNGAGVDDAEQLAYVGALQWGYGGSQPPLYTWINSIAGSVLGISLFTIYLVKFGMLASLFASVYFGARLLGLSRTVAAAGMVGIFMLPQIAWESQRTLTHSVGATTGCAWAFLAFAWHMKSRSWLSAMLLGLGFACALLGKFNASFFLLALILAGISIPVYRSVLLSRTSILAALAFAAAIAPTGLWALAHTENLLARTHKFEMNSGGQFFISRLHGEWKLFLNSVLFAGVALAFSAIAWWRSRADQAPEKRVWADAERLIARILIFALAGVFVGVLISGAAEVKDRWLQPILFLSPLFLAIVLERSTASHLPLKRFAVVGAICALIVIPGLAINMLYARDGKAPSIGQLDYARLFAVTRAEGAFGTVVSDGPQLPGNLRLFDNSITPVHAEMPNAASRIHLPALFVWFGEGMNPT-VLGLMNGAGIVMPPANIKSIELTYKYYPDRSEKVSYVILPAR---MKGDKKVIERLNEALFLELGAVNQYWLHYRLLDDWGYTKLAKKEREESIEEMHHADKIIQRIIFLEGHPNLQSIGALRIGQNIKEVLEADLAGEYDAVKSYKKSREICDQLGDYVSKELFDELLKDEEGHVDFLETQIDLLNAIGVERYGQLNAASANEVEMTITCFVQYEIDPYQKDAFEEYARNWNEAIPRCGADLIGYYAPHEGSATLAYAAYNIESLAAYEAYRARLMADPVGRANYEFSKEKRFIRREDRTFLRLVTGTN-GGLIR-MILSLFRSLP----KKSATLKAERSYEVAGRILPLRVMENPRATRLTLRIDAGGKGLRMTVPPGMPTREIDKFLTRHEGWIETRIAKMPDQPKVRPGVKIPVRGVPHLIVHEPAKRGTVRQENGADG-PLLIVYGDRPHLARRVGDFLKREARRDLEILVARHTATVGRHAKVIRLKDTKSRWGSCTSDGTLSFSWRIMMAPPPIIDYLVAHEVAHLKEMNHGPKFWKLCGELCPDTERCKAWLKRNGGALQAIDFS---------------------------MIGFAKWKILSIAGIVAAAGLASGCTQT-GS-H-SKLSANLT-RTSG---S------------KTYAYTPKDKECLERAMFFESNRSSQEGLMAVGTVVMNRLDSGKWGNSICGVVGQKGQFAPGVLSRPMKSAALPDVQAAADAVLKGERNPKVRNAMFFHTAGLRFPYKNMHYTVVAGGNAFYEKRDRYHTAEIASKDASIAIANAYIASTRMAGAKPANT-------------VLVASATQPVSTTSSTPTSGRVT----NQPIVVAQA---------DIAPMAFDTKKVAVPMDPPPQAMDYRTP--------QPVQPVQAATQSNVLAYEAPNGKAVDAIGQMLLAQDRPNSL--MAE-LNSLADLGTVA--ASTQAAAPVHVQKLDAKGRAYATGKRKDAVARVWIKPGSGKIVINEKEYGKYFARPVLQMILQQPIVAANRAGQYDIIATVAGGGLSGQAGAVRHGISKALTYFEPALRGVLKKGGFLTRDSRVVERKKYGKAKARRSFQFSKR--------------------MPFALKGMTVGLFGGSFNPPHAGHALVAEIALRRLKLDQLWWIVTPGNPLKDTTHLASLAERLRLCEAQVHDPRIKITAFEAAHDLRYTADTLAMIKARNPGVHFVWIMGADNLADFHRWQRWQEIALTFPIAVIDRPGSTLAFLSSRMAKTFDYARIDETDAPMLARSKAPAWTFIHGPRSLLSSTAIRNGVA--K----MKPVFLQLQCFPGKTYEVAAALFEREIVSELYSTSGDYDLLMKIYIPEDQDIGKFINDNVLDISGIARSLTTMTFTAF-MVRKVMKVLRGETVFPPPIWMMRQAGRYLPEYREVRKNAGSFLDLCYSPDLAVEVTLQPIRRFGFDAAILFSDILVVPHALGRDLHFEEGRGPLMTPIDVDGIFWLESNGAADRLEPVYETVRLLRQQLPNETTLLGFCGAPWTVATYMIAGHGTPDQAPARLFAYRNPDAFQKLLDALADVSAEYLIRQIDAGADAVQIFDSWAGVLDETCFEKFCVAPVARVVKKVRAVHPDVPIIGFPKGAGSLYVNYRERTGVTALGLDWTVPLSTASSLQGGGPVQGNLDPLRVVAGGRALDEGVDAILDSLGHGPLIFNLGHGITPDAPVSHVEQMVSRVRQAARRK----MSTRKTFAATASIAGAIAILASAAVPATA-QQQRAPQGWFKVCSKQEENDICNTQNIVTADSGQLLTAVNLIEIKGKINRKIFQVSVPTGRLIPPGVGLQINGGKTQKIDYAICFPDRCISETALSDELLASFKKGNQLTLTSVNFQNKPNPINVALTGFTQAYDGPGIQQNELEQRQKTLQDEVQKRQKEFEDKMKAEQAKAKAGN-------MSMALAEAHGAAARGEVPIGAVLVKDGTILASAGNRTRELNDPTAHAEILVIREACKVLDNERLTGCDLYVTLEPCTMCTAAISFARIRRLYYGAQDIKGGGVENGARFFAQPTCLHAPEVYSGFKEQEAETILKAFFRAKRP---------MILFPAIDLKDGQCVRLKLGDMDQATVYNTDPAAQAKAFEDQGFEWLHVVDLNGAFAGESVNGAAVEAILKATKNPVQLGGGIRTLDHIESWLARGLSRVILGTVAVRDPALVMDACKRFPGKVAVGIDAKGGKVAVEGWAEASTLGVIELAKKFEGAGVAAIIYTDIDRDGVLAGINWESTLQLAEAVSIPVIASGGLASIADIVRMTMPDAHKLEGTISGRALYDGRIDPAQALQVLREARERAA------MISFVVAIAENGVIGRENGLPWRLSSDLKRFKATTMGKPIIMGRKTWDSLGRPLPGRTNIVITRDPAFAVDGVIAVRSVDEALVVAGSHASADKVNEICVIGGGEIFRQTLNRADRLYVTWVLAEIDGDVHFPPIDPKTWEEISSEDFPAGEKDNYATRFVVYERR----------------------MGGKTITRADLAEAVYRKVGLSRTESASLVETILDEVCDAIVRGETVKLSSFATFQVRDKNERIGRNPKTGEEVPILPRRVMTFKASNVLKQRILRSHQTRKKKSA------MKSAVVLLPGLNRDRDMIAALTKISGVAPHTVWQTDTEIPDVDLIVIPGGFSYGDYLRCGAIAARMPVMQAIREKADKGVMVMGVCNGFQILVEAGLLPGALMRNASLKFVCREVKLEVTNANTAFTRGYSHGQIIRSPVAHHDGNYFADTETLARVEGNGQVVFRYAEGTNPNGSINDIAGIINENGNVLGLMPHPENLIEAAHGGTDGRALFAGILGVAAMRIERRFTKENQSAYAEIEFRVATSEIKNPDGSVVFRLENIDVPAQFSQVAADILAQKYFRKAGVPARLKKVEENSVPSWLWRSVPDEEALAALPADERYGSEMDARQVFDRLAGTWTYWGWKGKYFDSEADALAFRDELAYMLATQRVAPNSPQWFNTGLHWAYGIDGPGQGHYYVDPETGKLTKSKSSYEHPQPHACFIQSVADDLVNDGGIMDLWVREARLFKYGSGTGSNFSYLRGEGEKLSGGGRSSGLMSFLKIGDRAAGAIKSGGTTRRAAKMVVVDVDHPDIEEYIDWKVKEEQKVAALVTGSKIVKQHLAAIMKACVNCEADN----GDCFEPTRNPALKREIKAAKKNQVPENYIQRVIQFAKQGYTDIDFKTYDTDWDSEAYLTVAGQNSNNSVSLKDEFLRAVESDGTWNLTARKDGKVMKTLKARDLWEKIGYAAWASADPGLHFNTTMNDWHTCPAAGPIRASNPCSEYMFLDDTACNLASINLLTYRGKDGK-FDVAGYEHTARLWTIVLEISVMMAQFPSKEIAKLSYEYRTLGLGYANIGGLLMTSGIPYDSDEGRAIGGALTAIMTGVAYATSAEMAKQLGTFPGYAPNAASMLRVIRNHRRAAHGESQGYEGLAVNPVALIAADCPDQELITHAKLAWDKALALGEKHGYRNAQATVIAPTGTIGLVMDCDTTGIEPDFALVKFKKLAGGGYFKIINRAVPEALRTLGYSESQIAEIEAYAVGHGNLNQAPGINPGSLKAKGFTDEIIATLNTALKSAFDIKFAVNKWTIGEDFAKNVLGFTDEQLNDISFEILPALGFSKKEIEAANIHICGAMTLEGAPFLKEEHYPVFDCANPCGKIGKRYLSVDSHIRMMAAAQPFISGAISKTINMPNEATVDDCKSAYMLSWKLALKANALYRDGSKLSQPL---------------------NSSLLADEDEDEDE----AIDAYIEAPAAARAVQVTEKIVERVVEKYIHDREKLPNRRKGYTQKATVGGHKIYLRTGEFDDGRLGEIFIDMHKEGAAFRAMMNNFAIAISLGLQYGVPLEEYVEAFTFTKFEPAGMVQGNDAIKNATSILDYLFRELAVSYLDRHDLAHV-DQSDFSNTSLGRGISEGKAEPVSKGLTRGASLKVVSPGKADPKG---FSSAGGTGVSSAPKATASSNVTTLTSRAATAIAA----KPAIAAMPQESTAFKRDYEERAKDQTEAESNAAAALF-DNTDAEAAAEEAK--AEAKKVLSDRRIKSMMQGYTGDSCSECSNFTMVRNGTCLKCDTCGATSGCS-------------------MPIKKILVANRSEIAIRVFRAANELGMKTVAIWAEEDKLALHRFKADESYQVGRGPHLAKDMGPIESYLSIEEIIRVAKLSGADAIHPGYGLLSESPEFAEACSEAGIIFIGPKPETMRRLGNKVAARNLAIEIGVPVVPATEPLPDDMEAVKILAEQIGYPVMLKASWGGGGRGMRAIFSPDDIAREVTEGKREAKAAFGKDEVYLERLVQRARHVEVQILGDTHGNAVHLFERDCSIQRRNQKVVERAPAPYLNEAQRKEISDYGLKIAKATDYIGA-GTIEFLMDADTGEFYFIEVNPRIQVEHTVTEEVTGIDIVKAQIRILEGAAIGTPASGVPAQKDIKLNGHALQCRITTEDPEQNFIPDYGRITAYRGATGFGIRLDGGTAYS------GAVITRFYDPLLEKITAWSPTAEETIHRMHRALREFRIRGVATNLTFLEAIITHPKFTDNSYTTKFIDTTPELFAQVKRQDRATKLLTYLADVTVNGHPETKGRATPPKEAALPRVPFI-ETPIPDGTKQLLDQLGPKKFAEWMRNEKRVLFTDTTMRDGHQSLLATRMRTYDIARVAGTYARALPQLFSLECWGGATFDVAMRFLTEDPWERLAEIREQAPNVLLQMLLRGANGVGYKSYPDNVVKYFVSQAARGGIDVFRVFDSLNWVENMRVTMDAVVEENKLCEAAICYTGDILNSARPKYDLKYYVDLARQVEKAGAHIIAVKDMAGLLKPGAAKVLFKALREATDLPLHFHTHDTSGISAATVLAAVEAGVDVVDAAMDALSGNTSQPCLGSIVEALKGTERDPGLDPEWIRRISFYWEAVRTQYAAFESDLKGPASEVYLHEMPGGQFTNLKEQARSLGLETRWHEVAQAYADVNQMFGDIVKVTPSSKVVGDMALMMVSQDLSVADVKNPDKDIAFPDSVVSMMRGDLGQPPKGWPKDIQKKVLKDEKPFTERPGALLKPADLDAERNEIEGKLERKISDQEFASYLMYPKVFTDFALTHNTYGPTSVLPTHVYFYGLEQEEEVFLDIERGKTLVVRNQAVGEADEKGMRTVFFEMNGQPRRVKVPDRARAGSGSGVRRKAELGNDKHVGAPMPGIVSIVGISTGQKLNAGDVLLSIEAMKMETALRAERDGTIAEVLVRAGDQIDAKDLLVVYEMTHQPQQSPQFFLTAPSPCPYLEGQLERKVFTHLVGGRASELNDLLTQGGFRRSQNIAYRPACENCRACVSVRILAGEFKQDKSMRRVWNRNRDLIGNIHQAEPSTEQYSLFRHYLDARHNKGGMSEMTVLDYAMMVEDTHVPTQIIEYRRRGPDSFITKKGEGELIAVALTDTMGDGLSMVYSFFNPEHASRSLGTFMILDHIQRAAAAGLPHVYLGYWVEGSRKMQYKIRYRPQEHLGPKGWERQD-MTVLVTGGAGYIGSHMVLALKDAGRSVVVLDDLSCGFAWLVPDDVPFVQ-----------GDIGDQGLVRDLIRRHNITAILHFAGSIVVPDSVRDPLFYYRNNTVQSRALIEAAVAEGIKHFIFSSTAAVYGEPERTPITEDMPHQPISPYGTSKLMTEWMLRDAAIAHSDFNYVALRYFNVAGADPQLRSGQSFPRA--THLIKIASQAATGERSHIEVYGTDYPTADGTCIRDYIHVSDLAQAHLCALNYLEAGGKSTAANCGYGYGYSVLEIIDAVKRVSGVDFPVRTAPRRAGDPAILVAGNERVRNQFGFVPRRADLDLIVGDALAWERQLIDKRN-DEPEERIAVMRANYKMQRLYVTADLSFGKRAETAPEAVNYLANVLRMKDGDEILAFNGRDGEWRAALRFESKKKLFLEPQELTREQPAAPDLIYCFAPLKQGRLDYLVQKAVEMGAGVLQPVITQHTQVARIGIERIEANAIEAAEQCGVLAIPQTREPVKLENLLAGWDGQRRLIFCDEGHDTHNPLPLLQSLSRGPLGVLIGPEGGFSEQERQILRKLPFVTAIPLGQRILRADTAAVAALALIQATIGDW---------------------MTRITDLRVFDIRFPTSQSLDGSDAMNPDPDYSAAYVVLDTDVDGLSGHGLTFTIGRGNEICCLAIEAMKHLVIGLELDWVKENPGRFWHFITGDSQLRWIGPDKGAMHLATGAVVNAVWDLLAKEAKKPVWQLVGEMTPEEIVNIVDFRYLTDVLTRDEALALLRKAEAGKQQRIATLKAEGYPCYTTSAGWLGYSDDKLRRLCQEAVDAGFNHIKMKVGRDLDDDIRRLTIAREVIGPDRILMIDANQVWEVNEAIDWVKKLSFVKPFFIEEPTSPDDVAGHRKIRQAISPVKVATGEMCQNRIMFKQFIAEGAIDVVQIDSCRMGGLNEVLAVLLIAAKYDLPVWPHAGGVGLCEYVQHLSMIDYVAVSGTKEGRVIEYVDHLHEHFIDPCDIQSAAYMPPSLPGFSIEMKLESIRKYLYRAKAMQEMKLQELKNKTAVDLLAFAESLEVENASVMRKQELMFAILKKLASQEVEIIGEGVVEILQDGFGFMRSANANYLPGPDDIYISPSQIRRFSLKTGDTVEGPIRGPKEGERYFALLKVNTINFEDPEKIRHKIHFDNLTPLYPNERFRMELENPTTKDLSARVIDLVAPLGKGQRGLIVAPPRTGKTVLLQNIAHSITANHPECYLIVLLIDERPEEVTDMQRSVKGEVVSSTFDEPASRHVQVAEMVIEKAKRLVEHGRDVVILLDSITRLGRAYNTVVPSSGKVLTGGVDANALQRPKRFFGAARNIEEGGSLTIIATALIDTGSRMDEVIFEEFKGTGNSEIVLDRKVADKRIFPAMDILKSGTRKEDLLVPRQDLQKIFVLRRILAPMGTTDAIEFLIDKLKQTKTNGEFFDSMNT-------------------------------------------------------------------MKPEELKKLDSYFKRTFRNTELTVKARPRKDDSAELYLGDEFLGLIYKDEDEGELSYNFSMAILEMDL---MDIILASKSPFRATLLKNAGVAFTTQSADIDERAVEAPLYNSGASPEDVALVLAEAKAIDVSERNPAALVIGSDQTLSLGDEILHKPENMEAARRQLLKLSGQTHHLNSAVVLARDGQALWRHVSVARMTLRNLDPGFIGRHLARVGAIALQSVGAYQYEGEGVQLFEKVDGDYFTIVGLPLLPLLAELRREGAIDGMPFSVATWNINSVRLRLPLVLQFLADHQPDVLCLQETKCPDEFFPRDAFREAGYEHVEVSGQKGYHGVATLSRRPLRDTARVGFCNIADCRHLSTVVTAGSKRLRIHNFYVPAGGDEPDPEINPKFAHKLGFLDEMKAMNGDYGDGHASLLVGDLNIAPLETDVWSHKQLLKVVSHTPIETEGLEALRKNGGWSDLMRHHIPRHEKIYTWWSYRAADWAAADRGRRLDHIWGS----------PDLEND------------------------------------LTDITVLREARGW-------------------------------------------------------------DRPSDHVPVIARFDLDMSEKWARLVLSTYRWVGAATFPIVGAYVAFRASKGKEERGRHGERYGVASVARPSGPLIWVHAASVGETSAVTPLVEAIVDMGIHVVLTTGTVTSAKMVQDRLGDRVIHQYVPLDLKPAVDRFLTHWQPDLAVICESEIWPMTILELGTRRVPQVLVNGRLSDRSFANWHKRPSIAEALFENLAHVVAQSDIDGERFRALGARPVTVSGNLKVDTAVPPVNNGDLAAMQRMIGSRKTWAAISTHQGEEDIVAQVHHMLKARHRDLLTIIVPRHPDRAAEIAETIEKMGMKVALRSRGDQIEPDTDIYLGDTIGDMGLYLRLTEIAFVGRSLTAQGGQNPLEPAMLKSAILSGRNVQNFRDSYQRLIKNGGAKLVRDKDMLAGAVNYLFNNPAERQVMIDAGLKTVEDMRGSLSRTMTALEPFIQPLVLKARLSSREGGWK-MAGSAAHEAHD---AHKPTGWTRWVNSTNHKDIGTLYLIFAIVAGIIGGCLSIAMRAELQQPGIQIFHGLASMVYGFEGDAAVDAAKHMYNVFSTAHGLIMIFFMVMPALIGGFANWMVPIMIGAPDMAFPRMNNISFWLLPPALLLLVLSMFVPGPAGGFGTGGGWTIYPPLATSGQPGPAMDFAILSLHIAGASSILGAINFITTIFNMRAPGMTLHKMPLFAWSVLITAFLLLLALPVLAGGITMLLTDRNFGTTFFAPEGGGDPLLFQHLFWFFGHPEVYIMILPAFGIISHIVSTFSRKPIFGYLGMAYAMVAIGVVGFIVWAHHMYTVGLSLDTQRYFVFATMVIAVPTGVKIFSWIATMWGGSIEFKPPMVWAIGFIFLFTVGGVTGVQLANAGLDRAMHDTYYVIAHFHYVLSLGAVFGIFAGWYYWFPKMTGYMYNHTIAHTHFWVMFVGVNLVFFPQHFLGLAGMPRRYIDYPDAFAGWNEVSSIGSYISGVAVLIFLFGVFEAFAKKREAGANPWGAGATTLEWQLSSPPPFHQWEQLPRIKMNKLSIALAAS-LLVLSSAANALDQVASPQ----------PAIA-----------KPACGGELEPWFNGLINEAKAAGVSDKGISELFKASMDDKVLERDRAQGV-FNQTFLTFSSRMVSDYRLKQGAANLKKYADVFARAEAEYGVPGPVIAAFWGLETDFGAVQGDFDTLNALLTLSFDCRRPELFRPQLIALLKLFDSGVVDAKTTGAWAGEIGQMQMLPTDYLERGVDGDGDERVDLKHSAPDAIMTAARVMRDLGWRKGEPWLEEVKLTRELPWENAIRTNRLPHSKWAEWGVTGMKGPLGPDDGNASLLLPMGRKGPAFLSYANFDVFVEWNKSIIYATTAAYFATRLAGAGPLEPTNPEPGLNPEQMKELQNKLAARGHDMGPKIDGVFGILTRDAVREEQIRLGLPADSWPTPELLGKL--------------MTDAALRERLIVGLDVPTLTIAEQVVSELGDTVSFYKIGYQLAFAGGLGFASELVGSGKKVFLDMKLLDIDNTVAKGVENIVKMGVSMLTLHAYPKAMRAGVAAAKGSDLCLLGVTVLTSMDQQDMIDAGYEHDPHTLVLTRSEQALQAGMGGIVCAATEAAAVRKIVGPDLAIVTPGIRPAGSDHGDQKRVMIPADALRAGASHLVVGRPIVGAHDRKASAKAILAEMANV------------------MNK--HPAFQANSPRTVDEITQGRRLRRMRKADWSRRLVQENQLTVNDLIWPIFLCEGTNRREDINAMPGVQRFSVDMAVREAERAAKLGIPAIATFANIDPDLRDDAGSGILDADNLINRATRAIKNAVPEIGMITDVALDPFTSHGHDGILRDGIIVNDETVEQITKGAVLQAAAGADIIAPSDMMDGRIGAIRDALDQNGFQDVAIMAYATKFASAFYGPYREAIGTSGRLKGDKKTYYIDPANTDEAVREAEQDLAEGADSLMVKPGLPYLDIIRRLKDTFAMPTFAYQVSGEYTMIKAAGANGWIDEERVMMETLLAFKRAGCDGILTYFAPIVAGRLKAGS-----------MPRNNAY-SLPNILTYARIIAVPLVVLCFFLEGRLQSSDAARWGALAIFLIASITDFFDGYLARIWKQTSTIGRMLDPIADKLLVSAVLLLLAADGTIAGWSLWAAIIILCREILVSGLREYLAELKV--SVPVSRLAKWKTTIQMVAIAFLLAGPAGDKIVPIITIFGIILLWIAALITLYTGWDYFRAGLKHVVD---MSENTSEPKQLLHLVFGGELKKLGGVEFRDLDKLDIVGIYPNFETAQRAWKSKAQQTVDNAHMRYFIVHMHKLLDPDT-------GKAGMSSPYALTGAEIFDGENWHANSALIVDGGVVQSLTANSDLPAGLRQVALRGGKLVPGFIDLQVNGGGGVLLNDGPSVDVIRTICDAHFAFGTTALLPTLITDTPQITTAALAAGKAAAQEKVTGFIGLHIEGPHLSLTHKGTHDPKLIRPMDDADFMALIEAVQEVPVLLTTVAPETVPIEKIRALTDAGVIVSLGHSGASYEVAMAAKDAGATMVTHLFNAMSQLGHRSPGVVGAVLQSGELSAGLIADGFHVDKASMAIALRAKQGPSKIFLVTDAMSTIGTDITSFTLNGRKITRAGGRLTLEDGTLAGADLDMISAVRFVHRDLGLPLGEALRMASLYPAESIRIDHRYGRLAAGYVANIVHLDNELDVNHVWIDGDVHYAR------MLKEFQEFALKGNMVDLAIGVIIGGAFGRLVESIVADLFMPIIGLMTGGIDFSNLYFQLSGAPA-----PT-LAAARDAGATIA-YGNFITILINFLIVAWVLFIVVKAMNQFKKKEAAA---DPAPKKEEILLTEIRDLLAK-R----------MNAKTLDGEIITNRLDDWRLYQGVRTSRIFAFLIDYVIVFFLCIPVAVLIAILGVATLGIGWTLYGIMFPAVALGYVATTMGGQRQATKGMQMMGLRLERLDGQKVDGLLAVVHTVLFWGLNVVLTPLILLATLFLDRKRTVHDLLLGTVVVRAYD-MSEAPMT-STVAAETAR--TPQIPVTVLTGYLGSGKTTLLNRILSEDHGKRYAVIVNEFGEIGIDNDLIVESDEEIYEMNNGCICCTVRGDLIRVVEGLMRRPGRFDAIIVETTGLADPVPVAQTFFMDDDVRSKTALDAVVALVDAKHLPLRLKDSREAEDQIAFADVVLLNKTDLVTSEELANVEATVRAINPHAVIHRTTRADIDLSRVLDRGAFDLSRALESDPHFLDHDHPDHACGPDCDHN-HDH-HEHGR-HHHDHD------HGHDHASPIHDVTVQSVSIRTGE-LNPDKFFPWIQKITQEQGPNILRLKGILAFKDDHERYVIQGVHMIIEGDHQRAWKDNEKHESRLVFIGRQLDDAALRSGLESCLA-MKPKIFIDGEHGTTGLQIRTRLAGRDDLEVLSIPEAERRNREMRADFLKEADVAILCLPDDASKEAVSILEGHNSTKIIDTSTAHRVHPDWAYGFAELDRAQRQKIEGARLVANPGCYPTGAISLVRPLRDAGILPGDYPVSVNAVSGYTGGGKQMIAQMENPDAPDHIAANHFLYAMPLKHKHVPELQTHGRLERKPIFSPSVGRFPQGMIVQVPLFTDDLNGNASMQDIHAALSAHYEGQDIVQVVTLAESAMLSRLDPEELHDTDIMKLYVFGTEGQGQVNLVALLDNLGKGASGAAVQNLDLMLGNHR-------MNAHDTLLYLDGVSVSFDGFRAINNLSLVLQAGEMRAIIGPNGAGKTTMMDIITGKTRPDAGDVYFNGEVDLTKHDEAEIALLGIGRKFQKPTVFESHTVEDNLVLALSGPRSAFSTLFHRQSRDEIDRIHEILYTIRLYDKRVWLAANLSHGQKQWLEIGMLLAQDPKLLLVDEPVAGMTDAETAETAKLLRDIAKTRSVLVVEHDMHFVRELGVKVTCLHEGSVLAEGSLDFVSNDQRVIEVYLGR---------------METQLEINRPFAGLGGLFGAAGIAAYAAAAHSANGHMATIAPILFIHAPAFLVLSPLAKLSRAAHSGGLILVLGLLFFIGDLVSRDFAGDRLFAFAAPLGGTLLILGWLVVAATAFRILDYRK--MSDISEKPHGELWSREGFREDPYITAETLEEAGDAPAIILPLAVWLGLDEEVRNATNRKIGVSVAPGESIDPLLTLLDTIPVIALHFPAFNDGRSYSKAEMLKNQHQFKGELRAVGDVLIDQVAYMLRTGFDTLKVTHAVTLTRLADHNLHDTPVYYQPGRGSHTAPGTYSWRRVPAV-----------------MAFWLFKSEPDAWSWDKQKAAGAKGTEWTGVRNYQARNNMRAMQIGDKGFFYHSNEGLEVVGIVEVCALAHHDSTTDDPRWECVDIRAVKDVPVPVTLKDVKVNPKLEKMALVTSMRLSVQPVTADEWKEVCRMGGLVPAPEMAGTAVYKRVLLKASGEALMGDQGFGIDVAVADRIAADIAEARALGVEVGVVIGGGNIFRGVAVASKGGDRVTGDHMGMLATVINSLALRTSLVKIGIDTVVLSAVAMPELCETFSQRQATAYMDMGKVVIFAGGTGNPFFTTDSAAALRAAEIGADALFKGTQ-VDGIYSADPKKDPNAVRFDHLSHKDVLDRGLAVMDTTAVALARENNIPIIVYSIHEKGGFAEVLQGKGRATVVSDS----------------MRLDQLLVDKGIFASRSRARDAIIRGTVQVNGVLVTKPGMTVALDAAIEADDPASSYVSRAALKLIAGLDHFAIDVEGCTALDIGASTGGFTQVLLERGADHVIAVDVGHDQLHDSLRGNTRVINLENLNARDLLRDHLGGKSVDLVVSDVSFISLKLALPPALDLAEPGACCVLLVKPQFEAGREAIGKGGILRDPKDGERVANDLRNWLDGLPGWTALGVCPSPIEGGDGNREFLLAGLKVKA--MTARTILIVDDDSDLRAILVEQLSLYEEFQIIEEDNATKGVQAARNGVVDLLIMDVGLPDMDGREAVKLLRKGGFKAPVIMLTGHDTDSDTILGLESGANDYVTKPFRFAVLLARIRAQLRQHEQSEDATFVVGPYTFKPGQKMLIDAKGGKIRLTEKEVAIIKYLYRAGGKVITRDVLLEEVWGYNSGVTTHTLETHVYRLRQKIEHDPSNSTILVTESGGYKLIPMMSWLRFIVMAMAFALTILVLVPLQYVFLKTGWGPRTTTPILFHRTVSRLLGFRIHVHGEMAKQRPLLLTANHTSWTDIVILGSLREMAFIAKAEVATWPLFGMLAKLQRTVFIEREKRGKTHHQASVIATRLAAGDAMVLFAEGTTSDGNRVLPFKTSLFGAAQVAIRET--------DVETVTVQPVAIAYTRVHGMPMGRAHRPLVAWPGDVPLGPSLIGLLKDGAVDVDVWFGEPVVIDGKSDRKSLARTMEERVRAMVRTSLMGRDLASPQGESG------DDPILNGAKNR-MADILRKIEIYKRDEIAAAKASVTVEELKARVQDQEAPRGFLDALQAKRTAGQFALIAEIKKASPSKGLIRPDFNPPELAKAYELGGAACLSVLTDGPSFQGAPEFLQAARGATSLPALRKDFLFDTYQVFEARSWGADCILIIMASVTDDVAKALEETAMSLGMDVLVEVHDEAETERALRLSSPLLGINNRNLRTFEVNLAVSERLAMMVPPAKFLVSESGVFTHADCQRLAKSGIGTFLVGESLMRQQDVTAATKALLHSTAETQTDAA-MLIADRFLSPLV----GIGLLMSAMVGPCFAFDTQNEVKEDSSPFALFKFGFSAYKNGHKDEAVKALRFAAEKGHQGANWKLARMYAEGDGVKEDDYQAYKMFEQVVQEGADQGTENESYVADALVALAGYVKRGIPNSPIRSNPGAARDLYLQAASNFGDSDAQFELGKMLMKGEGGQPNPNQAARWFRLSAQKGNAGAQAMLGNLLFQAGKTVRGLAMMTAALEHASKQDRAWIRDVQEQAFSISDEADRRTAMVLAEDIIKTGDF-MKRIFAIALGAASLVLAGAAAADAADV-----PVYRDQGLRGGVKIGFLDCFVGGGIGYVLGSAKEIDCGFYSAL-SGEPLDRYSGAIRKLGVDVGFTTRTRVIWAVFAPTAGYRHGSLSGLYKGATAEATVGAGIGTNVLFGGTSGSVHLQPVSVTGQIGLNVAATGTSMTLASVN--------MQRSKLLGSVMGATL-AAFVVGSLPASAA---TGN-PPTLNAGTLSGAFLSARTAERGNDFASAIAFYRQALAYDPKNTELRQSLLLVLLTDGQFKQALPIADELKAVPEIERFSRLALGIDALNRKQYQKVNTLMMLSLQSDLDRMITGLISAWAKAGAGKPNEALSMIDKLQGPEGFSLFKTYNSALIADMAGQKDKAAVFYQSALEDRANVAAAPDTYERLVEAYASFKIRSGDKAGAEALVKEATDVLSGRMVLVEFGKELTDAKTVKPLIQTPQQGAAEVLYTLATAINRNGGEAFSKLYLQMSLPLRPDHDATLYQLGDISAKLDQPEKAIDFFGRISNGSAYRRDAEMQLAINLAILKRNDEAIQHLNGLLASDPGDMRTYLAIGSIYSQDKDYRKAADTYDKAVAALEAPTRNDWTIFYQRGIAYERLKEWDKAEPNFLKALDLYPNQPQVLNYLGYSWIDMNIKLEQGLDLIKKAVEARPQDGYIVDSLGWAYYRLGRYDEAVAQLEQAVKLRAEDATINDHLGDAYWRAGRKLEATFQWAHARDAKPEPEELVKIENKLKNGLSDEKDAGVAKNGVDAPKPPVEPAPAP------APDKKGMDRPDIPAILPEALEDKKVAATAWFAELRDKICAAFEQLEDELDGPLSDRTPGRFKQTPWRRDEGRGGGGIMSIMHGRVFEKVGVHISTVHGEFSQEFRKQIPGADEDPRFWASGISLIAHPQSPFVPAVHMNTRMVVTTRQWFGGGADLTPVLERKRTQEDSDTVAFHKAMKFVCDKHSAVANHEKFKAWCDEYFYLPHRKEPRGTGGIFYDWLHSSEEIGGWDADFNFTRDVGRAFSVVYPYLVRQNFNQDWTDADRQEQLVRRGRYVEFNLLYDRGTIFGLKTGGNVDSILSSMPPTVKWP-MLLQMIAATAMTLLT--VSAYAGDSIAIMGPSAKPNSVSYVGYDSFDANGNPICTPCQAKRAEEAARLKAYAERRERSRQYMARLQGKEVPAG----------STSMIAANASPLVP------AGEPVPEKTITN--VAETPLRAGM-MWIKLTDVNGDHLTLNFTHVVSFNPYGTGTHIVTATPGLTFFVKETTEEIQRKVGITAS---MRLSLISGVALAAVMAMTSFAKADITVGIIAPVTGPVAAYGLQVKNGVESAAEAINAAGGIKGEKIVTKIFDDAGEPKQGVSVANQVVGEGIKFVVGPVTSGVAMPASDVLAENGIVMVTPTATTPDLTTRGLETVFRTCGRDDQQADVAGKYILDNFKDKRVAIVYDKTPYGTGIGNGLKAVLNKGGVTEVVFEGINAGEKDYSALVTRLKSEKADVIYFGGYHAEGGLIARQLSDQGIKAQIIGPDGLSNTEYWAIGGDAAAGTLFTNSADPTKNPAAAKVVEALQAKNIPAEAFTLNAYAALQVIAAGIEKAGSSEPT-EVAAKIKSGEAIPTVVGDLTYSSTGDLTTPAFVFYKWEGGKAAQIDMSAFKSEFLHTLSMRGFIHQTSDDAGLDALFAKETVSAYIGFDPTASSLHAGSLLQIMMLHWMQKTGHRPVALMGGGTGMVGDPSFKDEARKLMTPETIQSNIDGIKQVFSNYLNFGDGSQDALMVNNAEWLLPLNYLEFLRDVGQHFSVNRMLSFDSVKQRLDREQSLSFLEFNYMILQAYDFVELNKRYGLRLQMGGSDQWGNIVNGIDLGHRLGTPQLYALTSPLLTTSSGAKMGKSLNGAIWLNADLLSPYDFWQYWRNTEDADVERFLKLYTTLPLDEITRLASLGGAEINETKKVLATEITALLHGRQAADEAAETARKTFEEGALATNLPSVEVATGELEDGIGILTLLVKAGLASSNGEARRHIQGGAVRVNDASISDEKAQIGASEITSD-GVIKLSLGKKRHILVKPVMAQEKRSREDR-GRSEERDSEFVDKLVHINRVAKVVKGGRRFGFAALVVVGDQKGRVGFGHGKAREVPEAIRKATESAKREMIFVPLRSGRTLHHDVEGRHGAGKVLLRAATAGTGIIAGGPMRAVFETLGVQDVVAKSLGSSNPYNMVRATFDALKHQMHPKDIAAQRGIKYSTLQARRRDVVGSEEMSTLASIADNAPWLKDSSLQKLLAVLSSGGEGARVVGGAVRNTLLGQRVGDLDIATTCLPNETSRRAEEAGFKVVPTGIEHGTVTVIANEQPYEVTTLRADVETDGRHAEVAFGREWRVDAERRDFTINALYVEADGTIIDLVGGLADIESRTLRFIGDPEQRIREDYLRILRFFRFFAWYGKGRPESEGLKACARLKDGLARLSAERVWSELKKLLAAPDPSRALLWMRQAGVLTAVLPESEKWGIDAIHGLVTTEQDLHWDADPLLRLASMIPPNVERVAELASRLKLSNSERDRLNAWTTTLPPQPEISEAGFAKKLYRSSEQGMRDRLSLALASARADA-VNDNAAMMRAGHLSKLLTFLERYEKPKFPLSGGDIVAAGLDQGPAIGQVYRALEDEWIESGFTLNRDDLVARIPDYPGRA--MTIFRFDLRGGAFRSVLGFTWKHWQKQPVRLAVILVAVLLSTLADVMTPLYSGRLVDAVVNGHPTEAVVWNAAIAAFSTLLGLSLGTIILRHIEFLAVVKLTLIMMSDIASGAFYRIQRFSTDWHANSFAGSTVRKVTRGMWALDLLNDTLLLALFPSIVMLVGSTALMAWYWPLMGVIIGIGSLTFVTVTVMMSLGYVAPMASLANRWDTKLGGALADAVSCNMVVKGFGAESREDARLAKVLMKWQDRTARTWVRGTRNGTTQGAMLLVLRAAVIGFALILWSKGQASAGDITFVLTSFFILQGYLREVGMHIRNLQRSVNDMEELVDIQGQPLGIEDQPGAKPIRITNGKIEFDNVTFLYGAHRLPLYDRFSVTIRAGERVGLVGHSGSGKTTFVKLIQRLHDLKAGQILIDGQDIASVRQASLRQQIAIVQQEPILFHRSLAENIAYGRPGATQEEIEEAAKQASAHDFISALPKGYGTLVGERGVKLSGGERQRVAIARAFLADAPILILDEATSSLDSESEVLIQQAMERLMLGRTTLVIAHRLSTVRALDRLLVFDHGRIAEEGNHEALIRLNGGIYRSLFERQALELTKGLVLNDD-----MLDS-------------------------LD-RTISLAAPAKINLALHVTGRRSDGYHLLDSLVIFAAFGDKVNVKHAPADSFAMSGPFGSELPDDENNLVLRARDALRRHFPEQAT-PVAIHLEKRLPVASGIGGGSSDAAATVRSLAALWGIEAEPEQLAAIGLMLGADVPMCLQGR----SLIARGIGEDIEHVAGLPHLPILLANNGLSVSTPQVFAALEKRDNPPLPALPALATVLDVCAYLAETDNHLFAAAARLTPAICETMDELRNTDARLVRMSGSGGTCFAIYDSDGEAETAAKNLRQRNPDWFVVATHSVKEGS--------MAISVAIETPLQDDVRALVDDLNEHLLPLSPIEFQFKMTVEQMADSDTTVFVARDESGHAVGCGALKDHGGGIGEVKRMFTRPTVRGQRVGSALLDAITALANKRGVTRLVLETGTGPGFADAYRLYERSGFTRCGVVLDYPDSEHSAFYEKWLAFAPAQ-MNTPCFFRKDDNGVTLFVRLTPKSSRDAIEGLETTDDGRAYIKARVRAVPEDGKANAALEKLLAKWLELAPRDVTIASGATSRLKQIRISGDPEALAVKLFALAPPEQS----------------------------MSMQGIGFGYRERAPYACDPNASRGRLVPESESPTRTVFQRDRDRIIHSTAFRRLKHKTQVFIAHEGDHYRTRLTHTIEVAQIARALARSMRLDEDLAEAIALVHDFGHTPFGHTGEDALNEKMREFGGFDHNAQSLRIVTNLERRYAEFDGLNLTWETLEGLVKHNG-------PLTDDAGKGLGHPVPAPILEYNAHHDLELARFASLEAQCAAIADDIAYNAHDIDDGLRSGLLSLDALEDVAITGDILRAVRQKYPALDDVRTSHEIVRRQITIMVEDVIRAAQANLEMLKPQTVGDIHGAGRTIVGFSAPLREEEKMLKAFLYKNLYFHASVVRVRKSADRIVRELFDAYLADEQCMPEGWRDGRKQADLAARARLVADFLAGMTDTYAVREHRRLFDDTPDLA-------MVVIRRATEADIAAIAAVGSRAWASNIFSFEPELPGMRAHVGQAFQDFAEASFSHVLVAEADGTVVGWGARDEDNDYISDLWVEPAVQGQGIGSSLLRAMKSAIAEAGYAKARISTHARNVGAIRLYQREGFEIVEQGPEWSTSLEREIDKVKMLAELG--------MSDTSHQNDPAA-PPKGIAGQESDAEGQSITRAPGPEKRSLLSAIFPFLRARPGTSLREDLDAALAEGRHGDTAFSPEERAMLHNILRLRELRVEDVMIPRADIEAVEFTTTLGDLLEMFEKSGHSRMPVFAETLDDPRGMVHIRDVVNHITKISRVK------ASRRS-ARTPK-APV-----AAKFNLANVDLTKTIAELSLMRPVLFVPPSMLANDLMGRMQAQRIQIALVIDEYGGTDGLVSLEDIVEMVVGNIEDEHDDDEVMIIEEPEGVFIADARADLDEVSDKIGAGFVVGEHGEDVDTVGGLIFSILGRIPVRGEMVQAVPGYEFHVLEADPRRIRKVRIVPLRH-AERR---PRTARQ-GMAEEPAETDE-----------------------------------------------------------------------------------------------------------------------------------------------------------------------------------MTKFLQYRNIQTPIGPMVAMANDVG-LSLLEFDDRPALPPEIEELERRYG---YTIEPGRNAILDHIEAELNGYFAGKL-TQFETPLVVPGRPFQLEIWSRLQDIPYGEACTYGDLARAIGNPGSSRAVGAANGQNRVAIVIPCHRVIGADGSLTGYGGGQRRKRFLLDLEHRVFAGMANRPVFHPITAQGSLF-MSGGIEQYIGIEQDLGTHTFSAEEIIAFATKYDPQRFHVDPEAARKSNFGALCASGWHTTAVWMRLNVDDTKSQVQKAIARGEKPPQFGPSPGFENLKWLKPVYAGDTIRFTRTLKRIRALQSRQGWSMMQMSSAAYNQNGDKVLEFDSAALIALPD------MSSAISVSSLYKTYSTGFEALKNINLDIRHGEIFALLGPNGAGKTTLISAICGIVSPTSGTILADGHDIIKEYRAARSKIGLVPQELTTDAFETVWNTVSFSRGLFGKPANPAHIEKILKELSLWDKKDSKLMTLSGGMKRRVMIAKALSHEPEILFLDEPTAGVDVELRRDMWGVVRALRESGVTIILTTHYIEEAQQMADRIGVISHGEIILVEEKDELMRSLGKKQLRLQLQGKRESVPEALEKYGLELSPDGCELIYTYDTQKDRTGITALLQDLSKAEVRFNDIQTKESSLEEIFVNLVRERKMAYNQKRVVDALRAFERGEIVVVMDDDGRENEGDLIVAAVHCTPEKMAFIVRHTSGIVCTPMPRDHARRLNLAPMVADNDAPHSTAFTVTVDYKHGTTTGISADDRTLTVRNLANPNSGAVDFVRPGHIFPLVAREGGVLMRSGHTEAAVDLCKLAGLPPVGVICELVNDDGSVMRGPQVASFAETHKLQQVTVADLIAYRQRKETLIERIGEYEIDTCAGNAKAITYALPWEPMHHIAIVYGDIRDGEDVPVRLQREDVLSDVFGARNTLESIMKRLTEEKRGVIVYLREGSVGV--GRDDDRNRTNLRD-----REAHAEARVREEEWRQIGLGAQILKDLGITSIRLLSSRERHYVGLEGFGIQITKTDIIMAHKKAGGSSRNGRDSESKRLGVKKFGGEKVLAGNILVRQRGTKWHPGVNVGLGKDHTLFALEAGVVSFRTKANGRSYVSIVNPIAEAAEMLRCMIVDGSPVVRKVARRLIASDTTVVTTADTGYQTVAACSVEMPDVIILDSSLSDMPAPDIIQQVRALPGGENSKIYLCLPEIDVTKIMRAKRAGAAGYLLKPFNRASITEILPQLPLAG---MTSNS------KKHALFLAAMLSA----TAVPMTSAFAANNLMELLFGSRRIESQPVLQ--PNQVR----PGAGESP--------KRPSVPVKRVTVTAPTNYDYKPEGLVQVDFAKVDPQLTASA-----------DHGIV----------SPP-------Q--MEQFGLKIDHLKDAHVLAEKEIADAIVSYYATKQRPIWTASYDVTPKAKAVAALFAKAAEDGLDPADYAVSLPSDDYDRSDIPARLKELSAFELMMSARALRYAMDQGEGRINPNRLSGFHDLAMDRVKPRDVIDQLGSSNDPSATLQAFAPQNKWYSELKQNLQELGD--SNEPVVRVTAGTMIRPGDENPEIKNVVALIKAKAPKDYLLNHEAVLQAHENANVYDESLVEAVKDFQKGQGRKADGVIGTNTIQALQGETTASKRDRIIYSMERLRWLPHDFGKRYVFINQPAYRAQYFDDYAEKLAMNIVVGSPTNQTYFFNDTIETVVFNPSWGVPRSIILNEMMPKILGNPSYLENSGYEVYDKSGRVIPSSSVNWYQVAANGGGVGIRQKPSLDNALGELKILFPNSHDIYMHDTPAKSYFKRDMRALSHGCIRLERPRDMAAAVLGVPVEDLKQYFGKNE-RSVRVKDQLPVYVSYFTAWPDAATGDIQYYDDVYQRDAYLEKAFLKTRAVRQTNVQAAQTARLMSSHLPRYAYEDFTVGSEWSLGSKLVTAEEIIDFAGQFDAQPFHLDEAAGKSSILGGLAASGWHTVSMFMRMFCDAYLLDSTSQGAPGVDYVRWKRPVLAGDTLTGKTTILDQRTSRSKPSLGFIKMHHDVFNQDGLLVCELEHTAMFSLREPVES------MNTDEKNSWTPFSHSSEAVRRVETAPETRQTQSDTYRLAFADPEFMTRRELRAVRLQLELLKPEMALAERGIRSTVVLFGGARIPEPGGAAWAAKNATQKKNLEASSHYYAEARKFAQLCSTYSSTTYNREFVVVTGGGPGVMEAGNRGAADVGAPTIGLNIVLPHEQAPNAYVTPELCFNFHYFAIRKMHFLMRAKAMAIFPGGFGTMDETFEALTLIQTGRMERIPFLLFGKSFWEKAVDFDFLAEQGVISPSDLKLITFVDKADEAWEVIRTFYDLE-----MVTRLYTHPIYLEHLTPLGHPERPDRLRALDKVLEDSAFDALDRVQAPLGDEATILYAHPESFLDRVRNTIPEEG------------LTRVDADTTVSPKSWEAALTAIGAANAAVDDVFEGRADNVFVASRPPGHHAERDKAMGFCLFNNAAIAARHAQKAHGAMRVAIVDWDVHHGNGTQDIFWDDPSVLYCSTHQMPLYPGSGAKDETGAGNIVNAPLSPMTGSDHFREAFNTRILPALDAFQPELIIVSAGFDAHHRDPLAEINLYEDDFDWATGLLMERAGSFASNRLVSLLEGGYDLKGMSLSAGAHIRRMMEG-----MQQVDVLIIGAGAAGMMCAIEAGKRGRSVIIVDHAAAPGEKIRISGGGRCNFTNLNAAPQNYISRNPHFCISALRRYTQRDFIGLVDRYSIAWHEKTLGQLFCDGSAKQIIDLLVTEMNRAGVELRLRTSADTIEKSGDGYVVDLSGER-----VRCASLVIACGGKSIPKMGATGFGYDVARQFGLALTETRPALVPLTFDDNTLAPLKPLSGISVDAVVSHGKTRFAEAMLFTHRGLSGPSILQISSYWREGDTISVAMLPGTRAFDVLRAARSRNGRQALQTALGELLPKRLAQLIAESSKIEGHLADLSDKQLLAVEKAVNEWQVKPAGSEGYRTAEVTLGGVDTDVLDQKTMETKSEKGLYFIGEVVDVTGWLGGYNFQWAWSSGWVAGQAVMRDIMGMMKQAKEMQAKMAAMQEEIAALEAEGHAGGGLVTVKLSGKGALTGLTIDPSLFKEDDIEVIEDLIIAAHNEAKAKVEAIMAEKTQALTAGLPIPPGFKLPFMATKTDIARRVFNHTWKLDPIVRSLLDTDFYKLLMLQMIWGVYPKVDATFTLINRTSKVRLAEEIDEQELREQLDHARTVRFSKKEMIWLGGNTFYGKRQIFEPDFLEWLSNFQLPEYELTRRDGQYELNFYGPWMYTTMWEIPALTIINELRSRAAMKKMGRFELDVLYARAKAKMWSKVERLRTLPDIRISDFGTRRRHSFLWQRWCVEALKEGIGEAFTGTSNVLMAMDNDLEALGTNAHELPMVLAALSTTRAELLKSPYKVLQDWNRYYGGNLLIVLPDSFGTAAFLRDAPDWVADWTGFRPDSAPPIEGGEKILAWWQSKGKDPRQKLLVFSDGLDVDTIEETYHHFRGKVRMSFGWGTNLTNDFEDCAPRYNDQLNAISLVCKVSEANGRPAVKLSDNPKKATGDPAEIKRYLELFGAEDRVEQAVKV-----------------------MVLVLTAALVVPYFVDWAGYRSSFEREASALLGRPVTVAGSASARLLPFPSVTFSDVKVGDPGTEPVMTIDRFSMDAELAPFIRGQILIFDMRLEAPIVSVAIDKDGVVDWAIRPHVPFRSAKVRLEDMRITDGSVVIRDASTATTRTITELDATLSAADLSGPWKFDGTLLFNGEKTAISAATGEVKPDGTLKLRTRIIPDGVPGALETDGDVTLNGGALKYAGSIAIRSAD----ELVA--------SAGTKAVDQVEKPLLSSLRVTGRFEADHGKINIPEFRMEQGAPDDPYVVNGNALFDYGSDPRFEVRADGQQVTFDN---------GKEQANGTKARAVTA-AARLGVFRRLMDQLPIPTIPGTIDLKLPAIVAGDTTIRSVAIDAAPSENGWTINQLKAELPGRTQFEANGVLQVGTDFGFDGKLLVASRQPSGLAAWLTDSVDESIRRLPGAGFSGDVSLRDELQKVDNLELALGGSSLKGSLVRSAKGESLPLTQLTLEGGALDADALAAFAAIFGNPANPAAG-RSPQLQGQDLDVHLKAGPVTHDGLVAESLDTAFRLRDGVFDIDRLTIGNVAGATITATGKLDPFKADPTGSIDSTILADDLAPFISALATRIPDFPFIKALNEHAANFPGLFQETQLTVLANTLR----------AKAGTDEFSLSAAGKTGGMDITLSGTTTKN--TDKLRTLELTMNARSGQAETLMALIGLPSLPLGLAGELEADLALKGNEKEGLQTQLSLKATDGQALVDGGFRDAGDGLTGEGRASIKTGDLEGYLATAGYSLPGFGNGMPADLASSFQLAKGRLTLPDLSGQLSGTKVSGRLGLAI-ENDVPVVQGDVKLARLDLPSVAQFVLGGDALDGDGR----TPWPKQVFAAAPLFPVSFNVKVTADEADAGVLGPISQFQTNAALKDGALRLDEAKGDLLGGRFDGMFELRNTSGTGLATGQFTLDQTALDALYKPAEGE---PPLKGKAKITASVNATGTSVAEMMESVAGSGVVSASDLTISAINPAALKPILADADAMEGPVTPALLNGTIGKYLHAGTFKAGAAEFAFTIAGGMARTSTFQLGSDGATLAADLRLNFPNMTVASQGRIAFDPGTAVVAGADPVIEFTLNGPWDNPKLALNRQPLEQFLTQRALEREQQRVETLQAALVEKQRLRRETQLYQARADERVRLAEEARLKAEQEA--REAAERAEAEK-----------RAAEERAA------AEKKAAEDAAA-KALEQSQPATP--------QPDQ---GGTATPS---PGAGTINKQSVDEFLKTLEPKSLEPNIQ-----------------MRDGLDLIEVSGAIKWFDVAKGYGFIVPDNSDLPDILLHVTCLRRDGFQTALEGARVVCEVKQGERGMQCFHVKSMDNSTAIHPTEMPPVRTHVTVTPSSGLERVLVKWFNRTKGFGFLTRGEGTEDIFIHMETLRHFGLTELRPGQVVLIRFGHGEKGLMASEIHPDIGTQLPSSHMIAVIFEVEPA-EGRGKDYFDRAADLRPLLETMDGFISVERFRSLANENRYLSLSFWRDEASVAAWRQTEEHRMAQRDGRAGIFADYRLRIASVVRDYGLRARDQAPFDARAYHDA---------------------------------------MTSR--PAYGLSDWGTSRFRLWLVDARGEAVAEKRSDDGLDASRVRGFAATLESHLAELGAPGNLPVIVCGMAGSRQGWVEANYVPVPADLSAILSGAVRIPGINRDVRIIPGLSQSGGSPNVMRGEETQLLGAILSRNLSNGIIAMPGTHSKWVTLENGRAKHFATYLTGELYALLASQSILRHSIGDTAASASPDHPQFAAALDLMLSGE-RMLGELFGIRAAMLLEDLSPEGAAARLSGLLIGAEVAGARAKNEGR---VTLVASGAMAALYGKALAHAGLEFDRVDADEAVRKGLFVAASHTWPITQ-ERPAMQPVFDGHNDVLYRLWKHSRDGADPVEEFIEGTTSGHIDKVRALKGGLIGGLCAIYVPS-GDLAFKAPDSNGHYSTPLAAPLERAPSLDIALEMASIALKLQQAGGWKLCRSTAEITECMSDGMFAAVLHMEGCEAIGADLAALDVFYAAGLRSLGPVWSRNNIFAHGVPFAYPMDPDTGPGLTREGEELVKACNRLGILIDLAHITEKGFWDVARISDQPLVASHSNVHALTPVARNLTDRQLDAIRESKGLVGLNYAVAMLRPDARENADTPLSDMLRHVDYMVNRMGIDCVALGSDFDGAKIPGEIGDASGNQNFVAALRDAGYAGDDLAKICRENWLRVLRSAWHEEN------MTEAIPTTRPLSRREWLMSDRPQSRMQARLGRAYLVWSRFASNRLAVVGLGIIIALLLVAIFAGVLAPHSPVSGDLRNARLLPPGGEYLLGTDDQGRDILSRLIYGSRLTLYVVILVAIIAAPLGLLVGTISGYAGGWVDSVLMRITDIFLAFPKLILALAFVAALGPGIENAVIAIAITSWPPYARIARAETLTVRNSDYIAAVRLMGASPTRIVLRHIMPLCLSSLIVRVTLDMAGIILTAAGLGFLGLGAQPPLPEWGAMIASGRRFIMDQWWVAAAPGFAILIVSLGFNLLGDGLRDALDPRGSNQMADNYIQ-RSHRQDDSRLGNDDPLLELSRIMG--AP-EDEESTAGSNDDLALDLERELMGGFD--------------------------------EQVT---VQSAGAIPDDQHAADDDHLAV--------------------QEFQDSIEREM--ALSEPSSDHQAI----------------------AAAVAAYEEPDYA-----DYEPDASYADGEAGEG---TAPVANA-EPVAPQYE-------------ERPGVAAPSLSLEDELETLLSGS---AQPVVQRSANASWGYGSQTQVAGRTE-PAPLISRTTSYQPPAA----PEPEIPQAAEVHEPITYD--------ATED------------------------QVQDSEHED-FDTEELMAAFDDFEVSADSDEVTAHSEEEQALPVEHNEPVFPRYH--SVVQSEVNAREPITVPVA-SPVASAPRLPEVHDLSDYADELDRAASSLDA-PDVDTVAVTENRVEQTEALNLPAVHYEDDVPPHSGLGELETEFAEVFGSIEAEDPRTAHAAHEEAP--KSETEQDQYADIFADVFG--------------------------NQAEQEHYSPSGY--A-AAAGAA-AV---GMAGAAAQKSR---SQAYRNGPE-EDADFGYDPQR-DEVEIAATPYGQQE-TKARRSSYFVPGVAAAVVLVAVAGAVAYKWTGNSGGEPVVIMADKTPIKVQPETTSTAVVPNQDKAVYDKEATIAPEAPKQDQLVTTREDPVDLAA-DEDE----------DMP------ATDKVEERVDPASEDVATSEPPA-QRNGAIAPRKVQTMVVKPDGTMVAAIPQEGEALSAGPAAAPAER-----PEPALAPVASNAPAPAPAADANMSAQPDESADDAIGSLVQ-GNEPPA---PGQAGAPAPVAAKPAPT-----APAAGKLP--AKPVETKKITQETVASAS--PKNI--------PVVESRPSEQPLDIVDRV--PP---KN-----AAGQQVASTAPAAGS-------YMIQIASQPNVEGAQKTYASLSQKYASVIGGRGVDIKQAEIAGKGTFFRVRIPAGSKSDAVALCTKYKSAGGSCFVTQ---------------------------------------------------------------MLTTLGGFAFVTIGITPIITLVLAAAYLLFALGQWFFDREDR-DRPVPQTHFTIAIDGPAASGKGTLARRIADAYGYHHLDTGLTYRAVAKVLMDQNMPLDDEFLAEKAARTVDLSKLDREVLSAHDIGEAASKVAVMPAVRRALVAAQQGFATRSPGAVLDGRDIGTVVCPDAPVKLYITASPEARAKRRFEEIKSNGGGADYEEILEDLTRRDARDMGRTDSPLKPAADAHLLDTTEMDIETAFLNAKTLIDQALAKNTH-----MHFIDLGAQRERISGKLDAAIAKVVREGKYILGPEVTEFEKRLADYVGVEHVIACANGTDALLMPLMAKGIGPGDAVFCPSFTFAATAEVVALAGAEPVFIDVDPDTYNINVEQLEAAIAAVRAEGRLQPKAIIPVDLFGLAANYTSLSAVAAREGLFVIEDAAQSIGGKRDNAMCGAFGDVAATSFYPAKPLGCYGDGGAMFTNDGALAEVLRSVLFHGKGETQYDNVRIGLNSRLDTIQAAILIEKLAILEDEMEARQVVAKRYANGLKDVVKVAHMPEGSRSAWAQYAIETRNRDALKAHLQANGIPSVIYYVKPLHLQVAYERYMRAPGGLPVSETLPQNILCLPMHPYLSEADQDRIIGTIREFHEKRA-MTTKSAALLTAVMLSAILFMGVGLFSVDAGGHTVSSDGYGISTASRMALSPKLDLRQTQALVMTPLLMQSIRLLQLTHVELEQFIDQEIEKNPLLERDETYG-DRLSLDERNLGGDRS-YSDGEDFNRASASGDSD----ARDDRDDPNDQSDH-WLVSGESTSAGSMSDTFDSSLENIFPDDPGTQDFIAGDLASQWKSSSGDGYVSVGGEGYNLEQVTASPLTLRDHVGEQIVFAFISAADRLIAAELADHLDEMGYLRADTCEIAERLGVDMAYVEKLIAVMQGFEPAGLFARDLAECLALQLHARNRLDPAMKTLLQHLDLLAKRDFNSLKKLCEVTDADILDMLREIQLLDPKPGTAFSSGVADSIIPDVQVDAGPDGTWRIELNPDALPRVLVNNNYYATVTKAKVSTEEKTFLSECLQNANWLTRSLDQRAQTILKVASEIVRQQENFLLYGIAHLKPLNLRNVADAIGMHESTVSRVTANKYMLTKRGVFELRYFFNAAISAT-EGGEQHSSQSVRHQIKQLIDAEAADDILSDDTIVDLLKQQGVEIARRTVAKYREAMNIASSVQRRREKKAQTSVRKGDGRSGFVRNTGVGFLRVGK----MLAYLTPLAGKRVLFAMAVDAEYGPHLQQLFTPLITGVGPVEAAVSVSATLASLAANQSLPDLVVSLGSAGSRTLEQTEVYQVTSVSYRDMDASPLGFKKGATPFLDLPVTVPLRHRIPGVKEATLSTGANIVSGNAYDLIDADMVEMETFAVLRACQRFELPLIGLRGISDGAAELRHVSDWTEYLHVIDEKLARAVEAMAEAIVSRAIVL------MGQTVHHYLVFETAGGFCGIAWNRIGITRFQLPTRSAEATERNLLRRLPGAEPGSPPPEVAEAVAAAKRYFEGEETDFSGLGLDLGEQDEFFGKVYAAVRRLGWGHTTTYGALAKELGAGPEAARDVGQAMARNPVPLIIPCHRVLAAGGRVGGFSAPGGSTAKMRMLELEGVSPGSLDAAQQSFGF-------MSESKAWIAGTAGLKLTPDEIAFFRDERPWGFILFARNVSEPAQIEDLCAHLRDLVGRDEALVLIDQEGGRVQRLRPPLAPNYPAGSALGALFREDEEKGLRAAWLLSRLHAFDLLKLGINVDCLPVLDVPIEGANDVIGTRAYGKHPEIVAAMGRAAADGLLAGGMLPVMKHIPGHGRAFADTHHELPTVSTPLEELAEHDFAPFRTLADLPMAMSAHVIFSAVDPKAPATTSGKVVEEIIRDYIGFDGLLMSDDISMNALSGDYFDRTKAIFAAGLDIVLHCHGIMEQMRAVASCTPELEGKAFERAVRAIDYRKQPDASNEEELREEFQRTFEAVAMTSSAK--IPSVDPVKLDRLAEVAIKIGLQLQPGQDLLITAPLAAVPLVRRITEHAYKAGAGLVTSFYSDEDATLMRYRNAPDDSFDRSAGWLYEGMAKAFSANTARLAVAGDNPMLLSSEDPEKVARANKAQSRAYQPALEKIAGFDINWNIVSYPNPSWAKQVFPDQDPEVAVTMLADAIFAASRVDVDDPVTAWRDHNATLRKRTEWLNARTFSALHFKGPGTDLSVGLADEHEWHGGASVAKNGITCNPNIPTEEVFTTPHALRVDGHVSSTKPLSHQGTLIENIRVRFEGGKIVEAKATRGEEVFNKVLGTDEGARRLGEVALVPHSSPISASGLLFFNTLFDENAASHIALGQCYSKCFINGGELSPDEVAARGGNKSLIHIDWMIGSGEIEVDGINKDGSVVPVMRKGEWV--------MTSTQAPLVDMENISIAFGGIRAVDGASVNLYPGEVVALLGHNGAGKSTLIKILSGAYKRDSGTIKVNGEEAAISNPRDAKSHGIETIYQTLALADNVDTAANLYLGRELMTPWGTLDDVAMEHSAREVMGRLNPRFQRFKDPVKALSGGQRQSVAIARAILFNARILIMDEPTAALGPQETAQVGELIKQLKSEGIGIFLISHDIHDVFHLADRVAVMKNGQVVGTAKVGDVTEDEVLGMIILGKCPPGAIPGPGAVN---------MSTFEPLVFSGVQPTGNLHLGNYLGAIRRWVALQESNNCIYCVVDQHAITQAISVWGGPAELMRNTREVTAAFLASGIDAKKHIVFNQSRVHQHAELAWIFNCVARMGWLNRMTQFKDKAGKDRENASVGLFTYPNLMAADILAYRATHVPVGEDQKQHLELTRDIAQKFNTDFSDRIAELGLGVETQSGDETIASFFPLTEPVIGGPAARVMSLRDGTKKMSKSDPSDLSRINLIDDADTISKKIRKAKTDSEPLPSDVDGLKDRPEADNLVGIYAALIDSDKESVIRDFGGQQFSVFKPALADLAVEKLAPIAAEMRRISADPGHVDAILKDGGE----------RASVLAEKTMRNVRDIMG----FLQD------------------MKIEKEETEKGGRYVAVVSGHEAEMTFSRASPHLIIVDHTAVPDALRGQGVGQALALHAVEDARAGGWKIIPLCPFMRAQSLRHEEWADVIQQRMTESLVNARTP--TVLRPPAPPKGWTRSRVFGYLLVGLWIALLLGLVVYLYSAWNIELVKKYGPTYLSGLGTTITLVGSSIILGAVLSIPVVLGRLSKNRIIASIAYFYVYFFRGTPLIAQVFLIYYGVGSFSKELQSVGLWIFFREAWFCALLAFGLNTAAYQAEILRGAIRSVPLGQWEGAASLGISKSVTFRKIILPQALIVALRPYGNEVILMIKGSAIVALISVYDLMGYTKLAYSRTFDFQTYLWTAIIYLILVEILRHLWDWMERRITRHLIRMAYKDPENTILLETTKGDVVIELYPDLAPEHVNRIKELAREGAYDGVVFHRVIDGFMAQTGDVKFGKQGGISFNPARAGMGGSDKPDLKAEFSNANHVRGACSMARSQNPNSANSQFFICFDDAGFLNRQYTVWGQVIEGMSNVDKIKRGEPVQDPDSIVSMRVAADVEMTNAIYAGSFDPITNGHMDVLQGSLRLADKVFVAIGIHPGKAPLFSFEERVDLINRVAAEVFGKDSSRLEVIAFDGLLIDAARKYGASLMVRGLRDGTDLDYEMQMAGMNGKMAPELQTVFLPADPSVRTITATLVRQIAAMGGDVRHFVPALVAEALETKFKSMADTDDLRFQNDQPRIHPSAQLKGVKLGKYAEVGERVILRDVTAGDFTYFERHSEGIYADIGRFCSIASNVRINALEHPMERLTTHKISYRPNEFFRYQGIDNAFRARRQAKRVNIGHDVWIGHGAVIMPAVKIGHGAVIGANAVVTRDVAPYTIVAGNPARLIRPRF--SDDVT--------------------------------------ARLLQLEWWD-----WPSATIFEAIPDIQSLSIENFLAKWETRGAG-MSDPAI----SLDRARASELGIVARFFKATELDTRLLGMIGALVIIWLAFQFLSG---------------GQFLTPRNLWNLSVQTSSIAVMATGMVLIIVTRNIDLSVGSVLGFVGMIMGVTQAEFLPKLIGFEHPATWIIALGIGILTGALIGGFQGFIIAFLGVPAFIVTLGGLLVWRGAAWWVTSGRTVAPMDSTFRLMGGGPEGAIGATWSWVVGIIACLAIVAMVVNSRRQRKRFNFPRRPVWAEVFMSAVGCGLVLGAVALANAYYWPIGIVRKYAEASGITIPDGGLFISHGIALPVLIAVATGIIMTFVTTRTRFGRYVFAMGGNPEAADLAGINTRWVTMKIFIIMGILCAIAGAISTARLNAATNAQGTLDELLTIAAAVIGGTSLAGGVGTIAGAMIGALLMQSLSSGMVLLGLDTPLQNIVVGLVLVVAVWLDTIYRRRTAMRTATAIETFLEMMSAERGAAQNTLESYRRDLEDAAAFSTATGLQLTQADPATIRAYLDDIAGRGFAATSQARRLSALRQFFRFLYTENIRTDDPTSTLDTPRKDRSLPKILSEADVERLLRRAEEETGDAK-ATSTAHFHAVRLHALLEILYATGLRVSELVSLPVTVARSDNRFLMVRGKGSKDRMVPLSGKAREAMSKYLAARDATPHLSDSLWLFPAISESGFLARQVFARELKGLAARAGLKTSAISPHVLRHAFASHLLQNGADLRAVQQLLGHSDISTTQIYTHVMEERLHRLVTEHHPLADMNLPTTGTVTICAMMISLAALAGCQSQGKPGPGSLAFNSEKAALPTMERVALAANTCWFKSGDSTFKPYRLAPELNSFSGRPRILVVPASNPGGRPLLVVHAEGNPAKVEAFGPLMSNSAGNRIAADVRRWAGGQSSCSASG-----MTSAFENPDWLEHRVTRIINADDERLAPYRNVRERDLVGREGRFIAEGKVVLNVFLSNPALAVESLLILENRLPGLAGQLKSCRDDVPVYCVSRQTMDAIAGFPMHRGILAVGRRNAPPTVDALIDALPNTALTIVLCGISNHDNMGSIFRNAAAFEASCVLMDETSCDPLYRKSIRVSVGAALKVPFARGGSIEDIVDKLQNRGFEVFALSPSGTTSIYDAAPGPRTALLLGTEGEGLPKSLLQKLQTVTIPMSKSFDSLNVATASGIALSRFSRFS-----GI-----MLRIAVWAVIAALTLFL--VFTYANTRFGDKAGAVQLGAPFTLQDQDGAPITEAAFNGHPTVLFFGFTHCPEVCPTTLFEMAGWLKDLGDEGKDLRVFFISVDPERDTPEVMKGYTAAFTDRITGITGKPEEMEKLLKSWKIYAKKVPTENG-DYTMDHTASVMLLDRNARLKSTIDYKESPDVALKKLRLLIAG----------------------MPGRLLLAAVLLLLMLASAFTAELPALTGRVVDAANVIDPATREQITQKLAAFEAKSSDQVVVVTVPSLDGEEIEPYSNRLYRAWALGQKQENNGVLLVVAPNDRKVRIEVGYGLEGTLTDLLSKLIIENAIIPGFRSGDFSGGISRGVDGILTVLSGDAAELEAR-----------ARRNIQEESS--DVDWFMLIFVTIFVFMFFSG-LILPAIVTSFGRKISPGRYEWLGMIFDIGSSRNGGGGGGGWSG--GGGGWSGG-GGG-WSSGGG---GGGGFSGGGGSS-GGGGASGSWMKAYNRQMLGAGRLATLAKRFLSDKRGVAAMEFAFIAPILIALYLGSIEATSGLDVNKKLGRSTNMVADLVTQQQ-TINTDQLRDIMEIGTALLLPYRSDT--PQITITAINIPA---AGSP-TVAWSRRVVNNVFSRPYAPGSNVVLDPNLVIPGTTV--IRVETKIAYVPLMAFNYKDSVGTAAGGSKLGIPMGRTAFGRVRQG-TAVACSNC-------------MKDSNHPFFRPLWRRVAVVAFCVAWAIFEFATGTPFWGVLALGFAGYGVWQFFIIFDASEPAAGTE-DKKEE------MRCPYCQSEDTQVKDSRPAEDGAVIRRRRVCPDCGGRFTTFERVQLRDLLVIKKSGRKVAFDRDKLMRSVEIALRKRQVDPERVERAVSGIVRQLESSGEPEVSSDEIGRLVMDALKGIDDIAYIRFASVYRNFREAKDFHDVIDEITSGDVTSDP-VENEMFAK---IEHGRLMDRVYRHQRHFYDATRKYYLLGRDPMIAGLNPPERGSILEIGCGTGRNLVLAGEAYPKTSLYGIDISHEMLATAHKNIAAAGLGGRAHLAYADAADFNPVELFGRRKFDRIFISYAVSMIPQWEAVMREAVSHLTPGGALHVVDFGDLKDLPRFTRTALYKWLEWYHVTPRNELLDVADKIAVETETETEAQRLYGGFAWISVIRRKA---------------------MHLIRTFFLGALALVCLVSAASALQLEPYKDDLFGYTGVLKTGDGGDYVVVDYNEMRDINGRDQVPERRVKQNYVSLQPKRSQQDLIVTTDYGPVKVMATGKLDGGASVITIYLHGQGGNRQQGMNDFTFGGNFNRLKNLMVRNDGLYLSPDFTNFGEKGEAEVAGLISHFKSQSPQARVFVACGSMGGSLCWRLAQDTRAAKNISGLIILGSLWDDHFIGSPSFKRRIPVFFGHGSRDPVFGVDKQEAFYREIRGSAKGYPVRFHRFENGNHGTPIRMTDWRTTLNWMLSVAP-----MFIQTEATPNPATLKFLPGKVVLEEGTADFRDAASAGEASQLAGKIFAIPGVTGVFFGYDFVTVTKSEGPEWQHLKPAILGAIMEHFMSGAPVMANATQVADTAQRETDGEFFDSADTEIVDTIKELLETRVRPAVAQDGGDITFRGYENGTVFLHMKGACSGCPSSTATLKHGIQNLLKHFVPEVQHVESI-MIQDHLYGDTIFALSSGRLPSGVAVIRISGPQTRFAVETICSNLPEPRQAVLKTFKDQDGNIVDRGLALFLPGPRSFTGEDCGEFHLHGGKAVVDAMLSALYSFERCRLAEPGEFTRRAFANGKFDLTVAEGLADLIAAETDSQRRLALQISSGAQAQLYATWRTELIRARALIEAELDFADESDVPGSVSDQVWLAMEDLAKRIRRHVADGKRGSIIRDGYRVVIVGAPNAGKSSLLNALAGSDVAIVSDEPGTTRDLIEIKLDLGGLPVLVTDTAGLRETDGKIEKIGIERALERASAADLVLALTDLSDPREPRLSDVDDRAILRIGTKSDL---ASTTDKYDLVISTRMDHGLDALLNALTDRAAAAAGNFSDPLPTRRRHMELLLDTGKEIDAAIEGIAAPLEVRAEFLRRASHSLGRITGDVDVEDILDVVFSQFCIGKMPVDILMPALSPTMEEGKLSKWLKKEGDKVTSGDVLAEIETDKATMEVEAVDEGTIGKILVPEGTDNVKVNAVIAVLLGEGESADAV-SAPKA-----AEAPANAEEAPKQEPA--SEQSAA----SVPAAPKTEVAADPDIPAGTEMVSTTVREALRDAMAEEMRRDPDVFIMGEEVAEYQGAYKITQGLLDEFGPRRVVDTPITEHGFAGVGVGAAMTGLRPIVEFMTFNFAMQAIDQIINSAAKTLYMSGGQMGAPMVFRGPSGAAARVAAQHSQCYAAWYSHIPGLKVVMPYSAADAKGLLKAAIRDPNPVIFLENEILYGHSFDVPKLDDFVLPIGKARIHKKGTDVTLVSFGIGMNYTVKAEVELAKMGIDAEIIDLRTIRPMDIPTVIESVKKTGRCVTIEEGYPQSSVGTEIATRVMQQAFDYLDAPVLTVAGKDVPMPYAANLEKLALPNIQEIIDAVKAVTYTA-----MLFEIRLLRAFACVSLA-LSLGACTTTEPKLPNA-ASAPVPESSPR---------------------------------------AAAATQARPTAK-----SVDGLISKYSVAYAVPESLVRRVVKRESSFNPQARNGPYWGLMQLLPATARGLGHDGSAKDLLDAETNLKYGVKYLAGAYKVADNNPDQAVRLYSRGYYYQAKRKGLLSV--LEPDT-----AP-VDPAPVAFAAAPSVAAAGPAAAATALAAPT--QLAFADERVVLPSVVPLPFERQDIMTV--TPATTGG--NGARARVQAPLMWTKQRRKTNRGRLIVPLMATLFLAYFGFHAYHGEYGLYSAIKLQEQTKLLQAQLDAVTASRTELERQVQLMHDGTIEKDMLDEQARKALNVSRPDEVTIMRGSGDLAINMFNLPARFIGTLAAAAMGASLAMSP-ASAADDTIKVGILHSLSGTMAISETTLKDTMLFLIDEQNKKGGVLGKKLEAVVVDPASDWPLFAEKARQLIEQDKVAAVFGCWTSSSRKSVLPVFEELNSLLFYPVQYEGEESSRNVFYTGAAPNQQAIPAVDYLAKEEG--VERWVLAGTDYVYPQTTNKILKAYLNAKGVKDEDIMINYTPFGHSDWQTIVSDIKKFGSAGKKTAVVSTINGDANVPFYKELANQGIKAEDIPVVAFSVGEEELAGLDTKPLVGHLAAWNYFQSVDADVNADFIKEWHAYIKNDKRVTNDPMEATYIGFNMWVKAVEAAGTTDSGAVIDSIVGVSVPNLSGGSSTMMPNHHITKPVLIGEVQDDGQFDIVYQTPGLVVGDEWSDYLPGSKDLISDWRKPMACGNFNIASGKCGGVTN--MFRWGILSTAKIGVTAVIPAICDAENSVVSAIASRDLAKARAVADRFGAPQAFGSYEAMLASNEIDGVYIPLPTSQHVEWSLKAAEAGKHVLCEKPISLHASEIAALQRARDSNGVLISEAFMVTYHPQWLKVRELIAGGAIGQLRRVQAAFTYFNKDAGNMRNQLSLGGGALPDIGVYPTVVTRFVTGKEPIRVAATVERDPDFGTDRYASVRADFDGFELTFYVATQLAARQSIVFHGDEGYIDVLAPFNTGKYDHARITVHNNNHSSATEYNFSDVNHYRLQVEAFARAAKGEDVPVFTLENSVLNQKLIDAIYRADETGKWETV-MSLGSILVADDDAAIRTVLNQALSRAGYDVRITSNAATLWRWISAGDGDLVITDVVMPDENAFDLLPRIKKSRPDLPVIVMSAQNTFMTAIRASEAGAYEYLPKPFDLTELVSIVGRALAEPKKK-TEKWAADEQPDTMPLVGRSPAMQDIYRVLARMMQTDLTVMISGESGTGKELVARALHEYGRRRKGPFVAINMAAIPRDLIESELFGHERGAFTGAQNRSSGRFEQADGGTLFLDEIGDMPMEAQTRLLRVLQQGEYMTVGGRTPIKTDVRIVAATNKDLRTLINQGLFREDLYYRLNVVPLRLPPLRERGEDIPDLVRHFFKMAAKDGLPEKRITADGLDLMRRYPWAGNVRELENLVRRLAALYPQEEINAEVIEAELKADLRPTEPASSAMANEEITIAQAVELNMQRYFLSYGDDLPPTGLYQRVLEELEYPLILSCLTATHGNQIKAAELLGLNRNTLRKKIRELGVNIYKSSKSD--RMKSATFISTDVIRASFSSAMSSMYRTEVPAYGTLLSLVTKVNDQTLAASPELRHRLEQTDTLDRISEERHGAIRVGTPAELSMLRRVFAVMGMYPVGYYDLSEAGVPVHSTALRPVGDAALKINPFRVFTSLLRLDLIADAELRDEARKTLQARQIFTATAISLVEKAEAQGGLALDDAALFVKEIIESFRWHDQASVSRDLYRRLHDAHRLIADVVSFKGPHINHLTPRTLDIDAVQAMMPAYGISPKAVIEGPPTRACPILLRQTSFKALEESVSFKNDQGAWEAGSHSARFGEIEQRGIALTPKGRALYDRLLAESRKVVRPAPDGSNADAYTAALADAFNAFPDDWDSMRRQGLGYFRFTPVQNFPHTSLSPRDASDVETLVALGAVQFDPIVYEDFLPVSAAGIFQSNLGDDDSQKFAASPSQREFEADLGTPVLDEFAHYEGLQKASIERCRQHYRPVALASMTKTRVPFRAIGSLARASAVASMVGMGLLVG-ASAALVTPALAQAAPAGIGAAQEIANKFSGVKTLTGNFVQFGPRGEQTEGTFYIERPGKIRFNYNKPSPIRVISDGSSVVINNRKLDTWDLYPLSKTPLKLLLSNQIDLSGGKVKSVKQEPDMTTIVLGDKSVFGNSTITMMFDPRSYDLRQWTITDAQGLDTTVMITNVRTGVRFADDMFKIDYTRIAMK-K---MNDLIAEAKAIAVLETCRRKGLLLATAESCTGGLIVASLTDIGGSSDVVDRGFVTYSNEAKHEMLGVPMELIAAHGAVSEEVAMAMAAGALAHSRAGIAVAVTGIAGPGGGSTAKPVGLVWFGLALQGKRPVAMRRVFLDHGRASIRRAATNTALDWILEALSADQVR----MAISG-----EALKAVTKPENHPRIETSKIGVLLVNLGTPDGTDQKSMRRYLKEFLSDKRVIEWPRAIWLPVLHGIVLNTRPKKSGALYDKIWNRERNESPLRTYTRSQGEKLGAALASHPEIIVDWGMRYGQPSIEQALERLDKQGCKRILMFPLYPQYSATTTATVNDKFFEALIKMRFMPATRTVPSYQDEATYIEALATSIERHYASLDFEPEVLIASYHGIPQSYFKRGDPYPCHCWKTTRLLRERLGWSEDKLISCFQSRFGPEEWMQPYTDKTLEKLAKDGVKSVAVFNPGFVADCLETLEEIAVGGAEIFHHNGGVNFTHIPCLNDSDEGMKVIETLVRRELQGWI-------MAFL----------ADALSRVKTSATIAITQKARDLKAEGRDVISLSVGEPDFDTPDNVKEAAIAAIRRGETKYTPVAGIPQLREAIVRKFKRENELEYKSNQVIVGTGGKHVIYNALLATLNPGDEVICVAPYWVSYPEMVALCGGTPVIVSAVQENEFKLQPEDLERVITPKTKWIIMNSPSNPSGAAYSWDEMKKLTDVLMRHPHVWVLTDDMYEHLTYGDFKFVTPAQVETNLYDRTLTMNGVSKAYAMTGWRIGYAAGPLELIKAMDMVQGQQTSGTSSISQWAAVEALDGTQAHLTVFKKAFEARRDLIVSMLNQTNFLECPKPEGAFYVYPSCAAAIGKTAPSGKVIATDEDFVSELLATEAVAAVHGSAFG--LGPNFRI---SYATSDANLEEAGKRIQRFCANLRMAQKPDEKAGNGNEATNGEGT--AQQQTLNILAQYVKDLSFESPGAPLSLRPRDKAPGISINVNVNANPIAENDFDVVLTLSAKAGEGKEVLFNAELVYGGVFRLVGFPQEHLLPLLFIECPRLLFPFARQIIAEATRNGGFPPLMIDPIDFAQMFQQRMAEEQAKAKVS--MRMTQIRHAKFQIGQVVSHRIFAFRGIIFDVDPEFNNTEEWYQSIPEEVRPRRDQPFYHLFAENADSEYVAYVSEQNLVPDTTGVPLRHPQIKNVFEKLENGVYRVKRPHINMTTASTSAHFKTAT---FLVLAMIVTVGSALGFQYIGGYLPCKLCLEQRYPYYAAIPLMAFAVASSSLKWPAGLTRLLLALGGVLMLIGLGLAIFHAGVEWKFWAGPTDCTAVAMSITTDAGSLLNDLNAVHPPACDTAALRVLGLSFAGWNAIASLILMVIAFRGAAKA-----MALLDVTTADKNIVDRRKLPRNKDLSAVMRGARDRLMERAGINHFERELLFMHTRALIVNSATIPLLIVMIALIGVFSGIGKSIVLWAAFTIALYAVLGLLARRLAKQGIDNEHIRQWQMIYFGGHFMTSIGWAYFAYLECTACGISLFPVIKAVVIILAMAITAIVSSALRAAILAAFTLPVITYTLLASNNLGNPLTAIMVVMLFAGLAFFYLVATRLNQSVAVTLALQAEKDALIAELETANAMSDEGRRRAEEANLAKSRFLASMSHELRTPLNAILGFSEVMAKEVLGPIQNGTYREYASDIHASGEHLLNLINEILDLSRIEAGRYSVNEEPLLLTDIANECIHMMNLKARNKDVSLIPQFELGMLRLKADERSIRQILLNLIANAVKFTPPHGKIHVKVGWTAGGGQYISVRDNGPGIPPEEIPVVLSTFGQGSIAIKNAEQGTGLGLPIVQALVHIHDGEFHLFSKLREGTEALATFPRSRVIQGNHPVA--------RKHGQKKAA-MLNDECSHGLWEKTAPPAPKTDRLQGDIAVDVAVIGAGYTGLSAALHLAEAGTKVAVVDAVEIGFGGSGRNVGLVNAGMWVMPNDLPGVLGQLHGERLLELLGNAPQSVFDLVARHKIDCELERNGTLHCAVGTAGVKQLEQRAEQWLARGAAVRLLDAKETAAKVGSTAYMAALLDNRAGTIQPLGYVRGLARAALGAGAAIYTGTPVAATERTGDRWILRTPAGTVSADWVIVATNAYTTSPWPELRSELLRLPYFNFATTPLPENIRETILPERQGGWDTKEILSSFRMDKAGRLVFGSVGALRGTGMAVHKAWAARSIAKIFPQIKDITFEAGWYGQIGMTRDSLPRFHTLAPKVISFSGYNGRGIAPGTVFGRILAEQIVGKITDADLPLPVTQPKDQ----------------------GLR----------------------AVRE---------GYYEVGAQLAHLVGNRF----MISAAQGRVQGRQPAAVIDIGSNSVRLVVYEGVTRSPTVLFNEKILCGLGKGLVKTRKLNVKAMQSALRALRRFRALADQAGAVSLDVLATAAAREAENGSQFIKEAEALLKEPIKVLSGREEAYYSALGIISGFHDPDGIAGDLGGGSLELVDIKGRQIGDGITLPLGGLRLQDMSNGDLTEATNIARKHLSTAKLLANGKGRAFYAVGGTWRNLAKLHMSAKHYPLHVMHHYEIPFDEAQRFLKLVAAGDLDYMRGIDDVSKNRRSLLAYGAIALLETIRLMRPSNVVFSAIGVREGFLYSLLPESEQLEDPLISAADELAVLRARSPAYARELADWSGEAFAALGYEETEDEKRYRRAACLVADISWRAHPDYRGSQALNMISNAAFIGIDHPGRAYIALANFFRHEGITNSVADPELAAIASPRLLEYSRVLAAIMRIVYPFSASMSGVVPNLTWKPTAE-GIDLVVNKSKADLIGDVPEGRLQQLARLTGKNLNMVVG--MTDPGFSLFQIALQQRAAAVETLLSGLLSDRPQGGEISRPPRLMAAMRHGVLNGGKRLRPFLVMESAALFGADNPAALRVAAALECIHSYSLVHDDLPAMDNDDIRRGQPTVHRKFDEAAAILAGDSLLTYAFELVASEETELDPHARVTLVTALARAAGVGGMTGGQALDLEAEKDQPDEEGIIRLQAMKTGALIRFACEAGSIIGNAHVKDRERLAEYGSAIGLAFQLADDLLDVTADASHVGKATGKDAAAGKATLVSMHGIEWTRQQLAGLVAQAEDLLEPFGEKAVLLKDAARFIAERQSMTLTADEIRRHLGLEPHPEGGSYIQTFRDNQ--SDWPEGGRGHSTAIYFLLERGEVSAWHRVKDAAEVWHWYGGAPLLLTIA-SESSPRETHKLGLDLAAGERPQAIVPAGQWQTATTLGDWTLVGCTVAPGFDFAQFELAQPGWEP----MNIVVQADKAAFGQGRAQRANALIEEAGLRIDSERRRGRGAGMNPSGRFEAESRHVYDDGWETIEDLPPFKTEVQVEKPRTIITRNDSPDISFDRSINPYRGCEHGCIYCFARPTHSYMGLSAGLDFESKLFAKPDAAKMLDKELSRPGYTAKMIAIGTNTDPYQPIEKKWRIMRDILQVLEAHNHPIGIVTKSALVMRDQDILTRMAEKGLAKVALSVTTLDGKLARTMEPRASTPTRRLQALRSLSDAGIPVSVMVAPVIPGLNDHEIERVLDSARAMGALEAGYVLLRLPLEVSPIFKEWLLRNYPDRYRHILSLIRSMRNGKDYDAEWGKRMRGEGPYAWQIGRRFEIAAKRLGMNLSKRRLRTDLF-AATPGSEQLSLFMSDKKNSNEMWGGRFASGPAAIMEEINASIGFDQKLYAQDIQGSLAHAAMLAKTGIISGDDHDKIASGLNTILSEIESGKFIFSRKLEDIHMNVESRLAELIGASAGRLHTARSRNDQVAVDFRLWVKAEFEKTANALKNLIEAFLKRAEEHAATLMPGFTHLQTAQPVTFGHHCMAYVEMFGRDLSRVRDAIRRMDESPLGAAALAGTGFPIDRHMTAKALGFREPTRNSLDSVSDRDYALEFLSTAAITATHLSRLAEEIVIWSTPQFGFVRLSDSFSTGSSIMPQKKNPDAAELVRAKTGRITGSLVGLLTVMKGLPLTYSKDMQEDKEAVFDAAETLELAIAAMTGMVGDMTINTAAMKKAAGSGYSTATDLADWLVRELGLPFREAHHVTGRAVALAEQKKCELGKLSLEELQQIYPGITDAVFGYLTVDKSVRSRRSYGGTAPGEVRRQIRFWQKRITKL--------MSQAEILNPP-PKTRLGKLDVLRGIALIAMATYHTGWDFEFFGYLESGTTGHGGWRIYARIIASTFLALVGFSLVLAHGRHIRWRPFGIRLAQIVAAALAITLATWYFTPESFVFFGILHEIAVASVLGLLFLRLPAWVTAIVAAAVIAAPHFLISSTFDAPIFWPLGLSEIIVRSNDYVPIFPWFGAVLAGMTIAKIMQQYDILRLLAGNIVPAWLDRSLRFIGRHSLAFYLVHQPVLISCVFLISQLFPPAVATPREVFGQACVQSCQNDNDRAFCQKFCDCVIGQTEALGIFDEVFAGQRDQ-NDPQMQEIAGICTQENMPQ----MAR-------------------------------IDETRPFVPVKIAVLTVSDTRKLEDDKSGSILAERVLAAGHTLAERAIVTDDRDKIRDRVLSWSKDPQIDVIITTGGTGFTGRDVTPEALEPIFEKRMDGFSEVFHRISYDKIGTSTIQSRATGGVVNATFVFVIPGSPGACKDAWDNILQYQLDYRHMPCNFVEIMPRLDEHLKRS-GKS-----------------MPQQ-VSMAFALPGGEKSTPSRNRPIDLVHLTRETFGNRALEIEILNLFSRQICGIVDRLAHANPDDRVRLAKSLKGSARAIGAFRVAEMAEAVEQAPSDLRKIKDLRPVIDDTRDYIAAITRMDETERLQES-----ASDEQDADIYGEDGAVRTSYLARVGAAIADRDVLYLRSHVGKLHQSELGHVLEALHSDQRAALVELLGDEFDFASLTEVDEAVRLEIVDAMPNEQIAEAVQELDSDDAVYILEDLDQEDRDEILAQLPFTERVRLRRSLGYPEETAGRRMQTEFVAVPPFWTVGQTIDYMRDNDDLPESFSQIFVIDPMFRLLGAIDLDRILRTRRDKKIEEIMHETRHAIPATMDQEEAAQIFEQYDLLSAAVVDENERLVGVLTIENIVDVIQEEAEEDIMRLGGVGDEELSDSVLSTSRSRTPWLVINLFTAFLSASVIGIFDGTIQQMVALAVLMPIVASMGGNAGTQTMTVTVRALATRDMDIYNAGRIIRREASVGLINGVIFAIMIGTVAGLWFENSNIGGIIAAAMIINMLAAALGGILIPLLLHRFGADPAIASAVFVTTVTDVVGFSSFLGLATWWFGFK---------MSDDPNTDAGKPARVAISRLKLANFRNYESLSLRLGLSHVVLTGENGAGKTNLLEAVSFLSPGRGLRRATYTDVTRNDAPDGFAIHAAIETAE-GSVDVGTGLAGNAPGDTARRVRINGVTAASADELLDYSRILWLVPAMDGLFTGPAADRRRFLDRMVLAIDPAHGKRVLDYEKAMRSRNRLLTEDRGNDAWLDAIETPMAELGIAIAAARAELMRLINGMIERLPDDSPFPKADCLLDGELEQRVGIEAATDVEETFRRALHDGRSRDRAAGRTLDGPHRSDLLVRHRPKAMPAELCSTGEQKALLIGLILAHAQLTGELAGMAPILLLDEIAAHLDEGRRAALFDIIEDLGGQAFMTGTDKALFRSLEGRAQFFDVSNGEIQQG-MTNKPDPLNFAHVTDWVFDLDNTLYPHHSNLFSQIDVKMTGYVADLLKLSHTDARELQKRFYKDYGTTLKGLMDRYDIDPDDFLQKVHDIDYSWLVPNPSLATAIKQLPGRKFIFTNGDRGHAERAARQLGVLDQFDDIFDIRAAELTPKPARETYDRFLSLHKIEAGSCVMFEDLARNLIEPKALGMTTVLIVPHNFEPTFSEIWERDPGNTDHVDYVTDDLTSFLNSILPMSARETV------MSNAPLKGIRVIELARVLAGPWAGQILADLGADVIKVENPAGGDETRGWGPPFITSKEGENLS-AAYFHSCNRGKRSITADFTSAEGQETVKALCRTADVVLENFKVGGLRKYGLDYESLSADNPRLVYCSITGFGQSGPYTTQAGYDFIIQGMSGLMSVTGEPD---REPQKVGLAVADVFTGLYSVIAIQAALTHAAKTGEGQLIDMALFDVQSAVMANQAMNYLATGKSPHRMGNAHPNISPYEVVPTSDGHMILAVGNDGQFARFCSIVGLTSLASDPRFLTNRARLENRTELTALIRSETIKHTRAELLGACARNAVPAGPINQIGEMFDDPQIVSRQLKLDLDDAHGSTIPSVRTPIIMSKTPLSYDRPSPRLGEHSAEILSELKDTSDENRRMAFRTIIAFIRSEREAKRVIGAARLIASSSDRTHIIGLYTIPSPIVYADPTGFADTTLFEAHEQRHKENSEAIAALFKAEMAKGTISHEFRIVRCESSSPSAGVTQSALRADIIIAGQPDPDDPDSVNDVTDPLVMESGRPVLFVPYKTALPERIDRVVVAFNGKREASRAAFDSLPLLLKAESVDIVWVDPKKSIDPSMD-IPGTPLSDALRRHGVNVNPHAIESHGEYADKILRRRIAENNASLFVMGAYSQSRLKEWVFGGVTSSIMADMPCLTLMSR--------MPSASLNGRAILKVTGEDAENFLQNLITTDLDALEQHDLKPGALLSPQGKILFEFLVSRDGNGLRLDTLRTSADDLLKRLTLYKLRAKVQIAVDLESLVQVSWENDSGTSE-------SDSTLHDRRFPDALNVRRHYGATRTAGNDEIEWTKLRIAHGVAEAPLDYLLGDAFPHDVNLDQTGGVSFRKGCFVGQEVVSRMQHRGTARRRILIVSCASELPATGTSITADGREIGTLGSVAGNAGLALVRIDRVKEATHKGIPILAGEVAVHLAIPPEHRFTFPE-ATQEA-MLDYIRDGQAIYDRSFAIIRSEADLSRIPADLEKLAVRVAHACGMVDVIQDLAFSDGAATAGRNALLIGAPILCDARMVAEGVTRSRLPVNNEVICTLSDPSVTTLASDMGNTRSAAALELWRPHLGGAVVAFGNAPTALFRLLEMIDDGAPKPALILGFPVGFVGAMESKVALAENSRGVPYVVVHGRRGGSAMAAAAINALASEKEMKIPTSLTSAARSTLLGSLALIAFGSLAAFAQEAAPA---APAAAAPAFTVDKGDTTWMMISTVLVLLMTIPGLALFYGGLVRAKNMLSVLMQVFTITAVVMLIWVFYGYSLAFTPGNA---FFGGFSKAFLAGVDVTTLSETFSKGVAIPELVFVVFQMTFACITPALIVGAFAERIKFSAVVLFVILWVTFVYFPIAHMVWFWGGPSAYSDPSGLIFGFGAIDFAGGTVVHINAGIAGLVGALMIGKRAGYKKDIMAPHSMTLTMVGASLLWVGWFGFNAGSNLEANAYAVLAMINTFVATAAATVVWILLESILRGKASMLGAVSGAVAGLVAVTPAAGFAGPMGAIVLGAIVTVVCYFFVAVVKNTFDYDDSLDVFGIHCVGGIVGALGTGILVNPALGGAGIVDYSTADFAAGYAGTATQVWAQFKGVAVTLLWSGIGSAILYKIVDLIVGLRAAPEAEREGLDLTSHGEAAYHS-MSLDNRPVYTGGCQCGAIRFRVEGKLGDASICHCRMCQKAFGNFFAPLTSVRGADFSWTRGEPKRFQSSNHVKRGFCANCGTPLTYEAPDGMALAIAAFDHPEELGPTVQWGLEGKLPYVDDLSALPGYTTEDDPESAEFVKTMISYQHPDHDTDQWPPMGPRGEAGE-------MSIQTDPVIIIGGGLAGLFCALKLAPRPVTVLAAAPIGRGASSAWAQAGIAAAVSEGDTMEKHVADTVAAGDGIVDEGIARLMASEAAARVHDLLEYGVPFDRDLEGR---LQVSREAAHSESRIVRVRGDMAGRAIMQALVKTVEDTPSIRVMEGYVVENIVTENGAATGVIARSEAG-RGKQIRLASRIVVIATGGIGHLYAVTTNPSEARGEGIGMAARAGAMMADMEFVQFHPTALNVDKDPAPLATEALRGHGATL-INRAGERFMLKIDPDAELAPRDVVARGIFAEV--TSGRGAWLDCTKAVGATFAEEFPTVYKYCRDAGIDPVVEPIPVVPAAHYFMGGILTDQDGRTSIDGLWACGECTSTGAHGANRLASNSLLEAVVFSARIAAKIGKG--------------------DEKHDGD----WSRGSVDRDDLQPE----------------------------PDTAAMSTLRQMMSASFGVIRDREGMLQGLKTILELERANRD-----------AAFRNVLATAKLVAVSALRREESRGGHFRTDFPEKRKQWQHRTFLTLSDATSTVDEIMELA----MATAGSSQIRGILFDKDGTLVDFNRTWFGITMELAHKAADGDEARARALVEAGGYDWEMEKFRGGSVVAAGTIHDIVDLWHPELTLAEKRERIRAYDDYAVREGSRRAVGIEGLRETLEALVAQGFVLGIATNDSEAGARATAGALGLTALFSAIIGYDSVTRAKPHADQLHLFATRTGLKPDVIAMVGDNAHDLEMAHAAGAGLAIGVLSGNSTLDDLGPLSDAILGSIAELPQYLKSRA------------------MTDGNQTQKSPRNGSRKIILLAGLAGIVAGAFAVYVMERPSGNIVAGTNPA-D--QCAIKADAAKAIDAAAMGAVAAMRGADAPQSLSSLTFNGPDGKPVRLEDFKGKTLLVNLWATWCVPCREEMPALDALQTKKGGDDFKVVAVNIDTGDDAKPKVFLDEI---GVKSLELYRDASMGVFNDLKRKNLAFGLPVTILVDKEGCQIAAMNGPADWGSDDAARFIDAARALN-METNRNFFITIALSIVILTLWQVFYMNPKIEAQREQAQIEATRQGQ-TRVDGQASA------PAGT------SADGT---NLPSS-TPATPG-ANIPGQSQDAAQAGSLTRAAALGQSGRVKIDTPSLRGSINLTGARLDDLQLKEYHETVDDSSPNIELLSPAQMADGYFAEIGFTGNELTGTVPGPTTVWTVEGNGALSPATPVTLVYTNDKGLTFKRTFSVDDNYMFTVKDAVTNSTSAPVSLASYGRVTRFSKPLHAS-TYVLHEGPIGVIGDDGLQEYTFSAIEKEKEVSPPKATTGGWIGITDKYWAATLVPSSDKPFQARMSYFEDGRPRYQSDYLSDPTTIEPGQSATIENLIFAGAKEVGKINAYEKERNIRQFELLIDWGWFYFITKPMFYLIDWLYKAIGNFGVAILVVTVLLKGVFFPLANKSYASMARMKLVQPKMTEIREKYADDKVKQQQALMELYKTEKINPIAGCWPILIQIPVFFALYKVLYVTIEMRHAPFFGWIQDLAAPDPTSLFNLFGLLPWAVPAFLMIGVWPLIMGVTMFLQMRMNPTPPDPTQQMIFNWMPLIFTFMLASFPAGLVIYWAWNNTLSIIQQGVIMKRQGVKVELWDNLAGMFKKKPKPAEMARSAILNVMVQAAMKAGRSLARDFGEVQNLQVSLKGPGDYVSQADKKAEEIIHAELRRARPDYSFLMEESGVIEGSDSQHRWLVDPLDGTTNFLHGIPIFGVSIALERQGQIVAGVIFNPAMDELYTAERGGGAFLNDRRLRVAGRAKLVDAVIGTGVPHLGRGHHGNYLVELRNVMGEVSGVRRMGAAALDLAYVAAGRLDGFWESGLSPWDIGAGIIMVREAGGFITDLDGGQDVVESKTIVAGNEAIQRALLKTLKKPV---MTIFDSIAPALSGALAARGYETLTPVQTAVLAPEARDADLLVSAQTGSGKTVAFGIAIAPTLLDGEDRFNSIGAPYALVIAPTRELALQVRRELEWLYEQTGARIASCVGGMDMTKERRALSQGAHIVVGTPGRLRDHITRGSLDMADLRAVVLDEADEMLDLGFREDLEFILGEAPEDRRTLMFSATVPKPIAQLAKQFQNDALRISATNDREQHSDIEYHIMPVAPRERENAIINSLLFYDAQNTIIFGSTREAVKHLTSRLSNRGFNVVSLSGELSQAERTNALQAMRDGRARVCVATDVAARGIDLPNLDLVIHADLPNNSETLLHRSGRTGRAGRKGICVLIVPPSRRRTAERLLQGAKLATTMVPPPDAAAINKRNHDRILNDPSLTEVVGEDEAAHVKELLALHSAEQIAAAYLRQQMAARPAPEELSSTPSHVLEPMRDTGRKGR-FEDRPE------RGERTPR-GRIEDFESGA-WFSISVGRKQRAEPRWLLPLICKAGDLTKTDVGSIKILETETRFEISASKADDFLARIKQFGSGEKGVNITR-SDGP--GFSSPRR-EGGDYKGKKSFGEKRDWDSAK-PRS-----RDDKPK-------SDWSAKPAKAKPEGWATEKPEKPKKTPKERKPGELHGFDKYKAKKARKAAAGGAE------MRHTRPSVSASILAAVRNRNKRPTGVTRVVRNDLLIASYNVHKCVGIDKKFDPDRVAHVIGEIGADVIAIQEADKRFGERSGLLDLARLHRENSLVPVPVTSLYPKGHGWHGNLLLFREGVVRRVQQLGLPGLEPRGALVVDIDLEAGPLRIIAAHLGLLRHSRQQQSEAIVRAVQADPARPTLLMGDLNEWRVGKGSSLHFLHPVFDPAKNAVPSFPSRFPVLALDRVLGHPHNLVTAIEVHDTPLARVASDHLPIKAHVDLKSALAEAEVLGVRDRATLARMTLSADAVRRLEEKTRHTFRDLGRLERALTHASARPSAGSDYERLEFLGDRVLGLVIAELLFRAYPTASEGELSLRLNSLVNADTCAAVADEIGLHEFIRTGSDVKGL-ADKRLKSLRADVVESLIATIYLDGGLEAAQPFIERYWNARSREINSAQ--RDPKTELQEWAHQQNGAQPQYTVVDRTGPDHDPQFTVKVDVKGFEPATGMGRSKRIAEQEAAVALLYREGVRQKAQEEDMK---------MDRIKIVGGNKLNGVIPISGAKNAALPLMIASLLTDDTLTLENVPHLADVEQLIRILGNHGVDYSVNGRRERQDGAYSRTIHFTARNIVDTTAPYELVSRMRASFWVIGPLLARMGVATVSLPGGCAIGTRPVDLFIDGLRVLGADIEIENGYVKASAKGGLVGNRYTFPKISVGATHVLMMAATLAKGETILENAAQEPEVVNLADCLNAMGARVRGAGTPTITIEGVTSLSGARVRVIPDRIETGTYAMAVAMTGGDVILEGADGNLLSAALVTLGQAGAEISETNSGLRVVRNGHGIMPVDVTTQPFPGFPTDLQAQFMGLMTKAKGTSHITETIFENRFMHVQELARLGARISLSGQTAKIEGVERLKGAPVMATDLRASVSLVIAGLAAEGETTVNRVYHLDRGFERLEEKLTRCGAVVERISD-------------MARIAGVNIPTNKRVIIALQYIHGIGQKFAQEIVEKVGIPAERRVNQLTDAEVLQIRETIDRDYQVEGDLRREVSMNIKRLMDLGCYRGLRHRRSLPVRGQRTHTNARTRKGPAKAIAGKKKMALPDILKTKLRIPVVGAPLFIVSHPPLVLAQCKAGVVGSFPALNARPEAQLDEWLAEITEELRNHDEKNPGRPAAPFAVNQIVHRSNKRLEHDLMMCVKYKVPIVISSLGAVPEVNEAIHSYGGIVLHDIINNRHANSAIRKGADGLIAVAAGAGGHAGPLSPFALVQEIREWFDGPLLLSGAIATGRAILGAQAMGADLAYIGSPFIATNEARAVDGYKQMIVGSTSSDIVYSNYFTGIHGNYLKPSITNAGMDPDHLPEADPSKMDFEAATT-GAKAWKDIWGCGQGIAAIKEVLPAGDLVARLEQEYHAAKAALCAA-------------------------------------------MAKLSKRVAKVREGVDRNKLYDLTSAIAMVKERAVAKFDETIEVAMNLGVDPRHADQMVRGVVNLPNGTGRTVRVAVFARGPKADEAKAAGADIVGAEDLFEIVNGGKIDFDRCIATPDMMPLVGRLGKVLGPRGMMPNPKVGTVTADVTAAVKASKG-GAVEFRVEKAGIVHAG-VGKASFDAKALEENIKAFADAVTKAKPTGAKGEYVKRVAISS--------TMGAGVKIDPSTVRSA---------------------------------------------------------------------------------------------------------------------------------------------MQQADIGLIGLGVMGANLALNIADNGYRVAVFNRTVVKTSEFYEEA--GRLKD--QIIPCETLEELAKSIRAPRPIILMVKAGEAVDEQIVALTPYLAKDDIIIDAGNANFHDTVRRLAELGPNDPTFVGMGVSGGEEGARHGPSIMVGGTPESYARIEPVLTAISA-KYEGESCSALLGPDGAGHFVKTIHNGIEYADMQMIAEVYGVLRDGLGLAPEAIGSIFEKWNAGPLNSYLIEITAKVLAATDKKTGKPAVDVILDSAGQKGTGRWAAIEAQMLGIPATGIEAAVAARSISSLVKERSEASKAYGDLGTAKLADGGQAFIDALEDGLLAGKIAAYAQGFDVMAGASKEHGWNIPLATTARIWRAGCIIRSQFLDQIAAAFEGSEGKNLLLAPAFIERMKTGSASLRKVVAQAALVGLPTPALSAALNYFDGYRQARGTASLIQAQRDFFGAHGFKRLDEEGDFHGPW-SDVG---MPEQKSSYGYEELLTCARGEMFGQGNAQLPAPPMLMFDRITEISETGGENGKGYIRAEFDIKPDLWFFPCHFIGDPVMPGCLGLDAMWQLTGFYLGWLGEPGKGRALSTGEVKFTGMVTPKTKLVEYGIDFKRVMRGRLVLGIADGWLKADGETIYKATDLRVGLFKESAA--MTSENFEFKLIANDGKARRGEITMPRGTVRTPAFMPVGTGGTVKAMYMDQVRELGADIILGNTYHLMLRPSAERVAKLGGLHEFARWPGPILTDSGGFQVMSLAQLRKLTEKSVTFRSHIDGSAYEMSPERSIEIQGLLDSDIQMQLDECVALPSTPQDIQRAMELSLRWAERCKAAFGNQPGKAMFGIVQGGDVADLRIRSAQALKAMDLKGYAVGGLAVGEPQHVMLEMLDITCPELPQEKPRYLMGVGTPDDILKSVARGIDMFDCVMPTRAGRHGLAFTRRGKINLRNARHADDPRPLDEESPCPAARDYSRAYLHHLVKSGEALGGMLLTWNNLSYYQQLMQGIRDAIEGKRYSAFMDETQNTWTRGDIPVFA-----MALA--PR----RALSVLALALLVPL-LSACGFNTIPTNEERAKAAWSEVLNQYQRRADLIPNLVETVKGYAAQEKDVLTSVVEARAKAT--QVQVTPETLTDPNAFKAFQDNQANLTGALSRLLAVVENYPELKSNQNFLALQSQLEGTENRIAVSRRDYIEAVRVYNTSLKTMPTMIWSWIWFTGNEPYQTFTIDDA-AKQTPQVKF-----------MKLMNEADHKSIAEAIRDAESRTSGEIYAVLARRSDSYFFVAGFTVACGILLAAVIVAVTAHW-------------FWYTVPLP--VFGLAIL---AAFLTAVLVLWLAPSITLWLVPKRILYRRAHLNAIQQFLARNVHLTTERTGILLFVSLAEQYAEVVADAGINAKVRQDEWNNIVAILTDHADRDDLTA-GFLKAIEKAGQLLEEHFPTGPDDVNELDDHLVEL--------------MSMHANQLSVFPAFFRVRNEVLVVVGNGEEALNKVRLLAQTNAAIRVVAAEPEPELADFLRNGNYDHVAENFAPKHLTDVKLVFVATGDEDQDSAIAAEARRQGVPVNVVDRPDLCDFFTPAIVNRAPVAIAIGSEGTGPVLTQMIRARIDAAFSPRLGDLARLANAYRPVVEKLVKKGLPRRLFWRSFFAGDVASNIYNNDLTGARRAATNLLEIQDEPKGYVWLVGAGPGAEDLLTLRAHRVLMEADAIVYDALVPEAVVAMGRRDATRLSVGKRKGCHSKSQSEINDLLVSLGREGKRVVRLKSGDPLVYGRAGEEMAALRDAGIAFEIVPGITSAFAAAADMQLPLTLRGVASSLVFTTGHDMAGEVLPDWARLAISGATIAVYMGRSVAASVAERLMQAGLHADTGVAVIENAGRPERRMFHGTLNDLPALEQRGDLDGPVMVVIGDAVAGAAIDKAEPLAAQN---KNSMVLRHLETNAA-MTPDVRPLVAGNWKMNGTGESLDELRMI-VNRPNSAIGEKIDALICVPATLVFRAAQSVEGEALSIGGQDCHFKKSGAHTGDISAEMLKDAGASHVIVGHSERRTDHGETDAVVNAKAKAGWDAGLVAIICVGETEAQRKAGSTLDVISTQLAGSIPDNANARNTIIAYEPVWAIGTGLTPTADDVQQVHASIRAALEKRFAADGGKMRILYGGSVKPANAVELLGVANVDGALVGGASLKAADFLAICEAYHSLEMTMITTTDQLEQAVAALSQSDFVTVDTEFIRETTFWPELCVIQLASPDHTAIIDALAPGLDLAAFFKLMADEKIVKVFHAARQDIEIIFHLGNLIPHPVFDTQVAAMVCGFGDAIAYDQLVQRVVGAQIDKSSRFTDWRRRPLSEKQLDYALADVTHLRDIYLHLKKSLEEEGRTEWVLDEMKILTARETYDMHPDDAWKRLKMRLRKPVELAVLRSVAAWREREARERNVPRGRVLKDDAIYEIAQQQPRDTEAMSRLRTIPKGWERSNMATGLLNAVNLALDLPKEDMPRLPKSVQSPEGAGAAAELLKVLLRLVTEEHGVAAKVVATTDEIDRIAAEGDVEDIPALQGWRREVFGEKALQLINGELCLKFENRKIRAVTWPAE-------------MAIGASKEKPATRRIRIGMVGGGQGAFIGAVHRIALRMDDQYELVAGALSSDPARAKASAKEIGISDDRTYTSFEEMAKAEAKRPDGIEVVSIVTPNHMHGPAAKAFLKAGIHVICDKPLTTTVKEAKELVSLVNKTGKLFIVTHNYTGYPMIRQAKAMVEKGMLGTLRLVQAEYPQDWMTEKTEATGSKQAEWRVDPKRSGAGGAIGDIGTHAYNLACFVSGLKLKQLCAELTSFGEGRVLDDDVQILMRFDGGAKGMIWASQVAPGNENGLKLRVYGTKGGLEWTQADPNYLWYTPFGKPKQLLTRGGAGASSEAARVTRVPGGHPEGYLEGFANIYTETAQAIIAARSGKKTPKDVIYPTVEDGLAGMEFIEAAVKSSKAGGVWTKV-MLEKTYDAAAVEPEIAKRWDEAGAFKAGAGSKPGADPFAVVIPPPNVTGSLHMGHALNNTIQDILVRFERMRGKNVLWQPGMDHAGIATQVVVERQLMERQQPGRRDMGREKFVERIWEWKAESGGIIADQLKRLGASCDWSRERFTMDEGLSKAVLEVFVTLYKEDLIYKDKRLVNWDPKLLTAISDLEVEQREIKGNLWHFRYPLENVAFDPENPHTFIVVATTRPETMLGDTGVAVNPDDSRYHALVDNNVVLPLVGRHIPIVADAYADPEAGSGAVKITPAHDFNDFEVGKRNDLRQINILNTDATIHLKDNDDFLEGLEPSRNLTQLIHMLDGQDRFAARKMIVGMMEEGGYLDKVEDHTHMVPHGDRGGVPVEPYLTDQWYVNAAELAKPAIASVREGRTNFVPKNWEKTYFEWMENIQPWTISRQLWWGHQIPAWYGPDGKIFVERSEEEALSAAIQHYLAHEGPMKAHVENLIENFKPGEILTRDEDVLDTWFSSALWPFSTLGWPDKTPELKTYYQTDVLVTGFDIIFFWVARMMMMGLHFMNEEPFHTVYVHALVRDKNGAKMSKSKGNVIDPLELIDEYGADALRFTLAIMAAQGRDVKLDPSRIAGYRNFGTKLWNSTRFAEMNGVARDPSFLPENAKLTVNRWILTELTNAARDVTDGITSYRFNEAANAAYRFVWNQFCDWYIELLKPVFNGEDAEAKAEAQACAAYVLDEIYKLLHPFMPFMTEELWAHTAGEGQKRETLLCHAGWPEPEFLDAEAAADINWLIDLVTGIRSVRAEMNVPASAMAPLVVIGANEVTQERLQRHDASIKWRARVETIALADDAPKGSAQFVIGEATACLPLGSLIDVNAEIARLTKEASKIADESDKTGRKLANEKFVANAKPEIVEAERERLAELRAAAEKIETAIRRVREAS-----MSNLQIEQFTARTDNFGVLIHDPEANLTASIDAPEEQPILAALKRRGWNLTHIFTTHHHGDHIEANIALKKRFGVQIIGPQDERSQIPGIDRAVSHGERFQFGNFSVDVISTPGHTAGEISFHIPAAKVAFTGDTLFSLGCGRIFEGTPVMMFRSLQRLLALPGDTEIYCGHEYSESNARFALTIDPENSALKERAREITALREAGLPTLPTTLLREMATNPFLRWHDPSIRKNLNMEKASDEAVFAEIRKRKDNFMSDTKTIADTRGFGRKFADLDKTAVAPFLALAALFLLGALVNPNFLSVDNLLNVLTRSSFIAIIAVGATFVISAGGLDLSVGSMAALVAGIMILFLNSGAIDGDTAMLAAGVVVALGVGALAGLLNGTIITIGKIEPFIVTLGTMAIFRSVTVWLADGGSIAMKSIAMRNMFRPVYTGVFLGLPFPVWLIIAVGLLGAFILYKTSFGRHVIAVGSNED-VARYSGIHVNRVRAMTYILQGICVAIAVVVYVPRLGAATTTTGMLWELQAITAVVIGGTALRGGVGRIWGTICGAFMLEIVGNIMVLSNIVSEYLIGAVQGTIIIIAMLVQRTLQKKKA-MTETPPLLFPHRHLLGIKGLSPQDIVQLLDLAESEIAVSRQSEKKKATLRGRTQINLFFEASTRTQSSFELAGKRLGADVMNMSVGNSSVKKGETLIDTAMTLNAMRPDILIIRHSSAGAAALLAQKVG--CSVVNAGDGAHEHPTQALLDALTIRRAKGKVERLTVAICGDVLHSRVARSNIILLNALGA-RVRVVAPSTLLPAGIAHMGAEVFHNMEEGLKDADVVMMLRLQRERMAGSFVPSVREYFRYHGLDREKLKYAKPDALVMHPGPMNRGVEIASDVADGSQSVIQEQVEMGVAVRMAVMEALLDSRRNLGRNAEG----VAG-------------MVLAAGLGKRMAPITNTIPKPLVKVAGKPLIDWGLDALEKAGVERAIVNTHYLADQMDVHLAERKAPAILVSDERDELLDSAGGIVKALAQIGTEPFYVVNADTFWIDGARPNLVLLAEGWDDARMDMLLMIATKDQSTGYDGR--GDFHMSTEGRLSR--------LGPGETSPYIYAGAAILHPRIFAGAPAGKASLNRYFDEAIAADRLYGLSMDGLWLTVGTPAAIGDAEAAIAL--QEKNG-------MNGKSTTPMEVLWESPSDRGLSEPSLVIDIKGFEGPLDLLLHLARNQRVDLARISVLALVEQYLVFIRQAQALRLELAADYLVMAAWLAYLKSKLLIPKAPGDDGESGEELAATLQFRLKRLEAMRDAAAKLVNRNRLGRDVFARGMPEAVIVDQTNTYAATLYELLTAYASQRQKR-AVSQVQIARRTVWSLKDARVILMRLVGETKDWTALDQYLLEFIVSPADRATAIASSFAASLEMVREGHLEIRQTGAFDPIYLRNKPVRTVEKRVAPF----EDMVDGMLPIHSLKSGARVLAATSALVVVFAGSAFAVDGNAVAARLKAVYAEQGGAIEYGSVETNGSTVILKDTKVSTAGVK---ESFNAGDVTLSDVSDVSGGGFKIGSLTMPDIDFSPEGAPENKVSIDGISLQGITLPAEGATEPLAKMLQYERAEVKHLNVNAKGKDVFTADNLTATISPLSETSPVTFSSNVESFEADLSDVEDPKTKDALKALGYETISGKIAMNGTWSLSDGRLNFEKMDFIADNAGTLGLTFDISGYTLDFIKGLQEATKNMEGKPD-DAQGMAMLGLMQQLSFTGASVRFEDASVTYKALDYVANQQGAKRADLINQAKAIIPMAAAQLGNAEFSQALGQAVSTYLDDPKTIEVKAAPAKPVPFAIIAAGGMADPKSLIKTLRVTVVANQ----------------------------MLIKLEDLVPEILARAAGIPRLIVAIAGPPGAGKSTAAASLCAAINMQDEAAAVVVPMDGFHLDNAILDAMDLRKRKGSPPTFDCAGFEVLLRRLRETREDVVIPLFDRKLDLARAGAGIVKADQRILLVEGNYLLLDQPPWNRLAPLFDVTIFLDVDRLELENRLVHRWLTHGYNVGSAQARALSNDMPNAELVLEESRAADYTVQN---------MSESTTVQRLAFWSIFIGLTVLAMKYGAYYLTGSVALYSDALESIVNVVAAMAAWWAIRVSYKPADQNHPFGHHKAEYFSAVLEGVLIVVAALLILREVWLAWATPRTLDQPWLGLAINGGATIINAFWASLLINRGRKHRSPAMQADGKHIMTDVVTSVGVFAGLVGAIATGWTFLDPLLAFIVALNILWQGWNVIGSSVQGLMDVGVATEETMRIRDVISANAGGALEVHDLKTRIAGRMTFIEFHLVVEANMSVGDAHIICDRIENALMQQIPDASVVIHVEPEDEAKLPLGTVAVPFAMTDTK-TPQPAQEFERVLSQSATAVASFDTHEKSPLEKIRHFLHSSPASVPLIVLVMSLIIFAVIVGSRFFTPFALSLILQQVAIVGIVGAAQTLVVLTAGIDLSVGAIMVLTSVIMGQFTFRYGLPVELSILCGIALGALCGFINGVLIAYIKLPPFIVTLGTWQIYLATTYIYSANETIRAQDIEANAKLLQFFGEKVPIGGAT-FTYGVFIMILLIAILWYVLNHTAWGRHVYAIGDDPEAAKLSGVRTKRVLIAVYVLAGLICALAGWVSIGRNGSVSPQIGQFANIESITAVVIGGMSLFGGRGSIMGMLFGALIVGVFSFGMRMYGTDVQWTYLIIGVLIILAVAIDQWIRKVAGMAGTDRQRTLIAEGPAIILVEPQLPENIGMVARAMANFGLAELRLVKPREEFPNDKARSAASRADHIIDAAQVFDDLPSAIKDLNFVYATTARERDAFKQVRGPVEAGQTLRQRFNKGEKTGILFGRERIGLNNDEVSLADELVTFPVNPAFSSLNIAQAVLLMSYEWMKSGLEKHTDTAFRGPELEPAPKEQLQGFFNHLEDALQARGYFRPMARKEIMVNNLRGVLTRAGFSDSELKLLRGVLVSLDYFSPKTPRGSGAPEGRKREKPAVTQT-----SNEDSSNDE---------MTLNSLDLPGRPEDTRIVVAMSGGVDSSVVAGILKRQGYDVVGVTLQLYDHGASTHRAGSCCAGQDIEDARRVSERLGIPHYVLDYEARFREAVIDPFAASYVSGETPIPCVSCNQTVKFADLLQTARDLGADALATGHYIRSHANGVHRALYRPVDTDKDQSYFLFATTQEQIDYLRFPLGGMTKTETRAIAEELGLTVANKQDSQDICFVPQGKYSDIISKLKPGAANPGDIVHIDGRVLGRHEGIVHYTIGQRRGIGVATGDPLYVVHLDAQNARVIVGPREALETRKVFLRNVNWLGDGPLSDIPEGGVEVYAKVRSTRPPRPAVLHHQGGDTWVELVDGESGIAPGQACVLYSDDSNTARVFGGGFIGRSERAPQAEAMLKRLAQQGQHA---MRLTAETTGTFDPDSALVESFEIINRRGLHARASAKFVQLVDGYDAHVRVEKDGMTVGGTSIMGLMMLAASPGCCIKVSASGIQAPEVMKALGALIADKFGEEAMSIWRRISDFITSTAIDAFSSIIEAVRTTFEGDPETRRRVAFSIAMIALSAKMAKADGVVSQVEVNAFHDIFHVPQQYQDQVSRLYNLAKQDVAGYEAYATQLAGLCGSGKPNCKMLEDVLDGLFHIAKADGALHDKELTFLGTVAEIFQLDEEQFDQILARHAGRGHADPYLVLGLPRDVSFEAARKQYRALVRENHPDILIARGVPEEFIAIANQRIAAINAAWDKVEKDLKAYESV--------------------------MFAAGSFLAPEKAHADFRVCNTTQNLVGVALGYRAKTGWITEGWWHVNPSSCTTLVVGPLTSRYYYLYAEDAQSGGRWDGKVNMCVAENQFKITGINDCFARGFQRSGFQEYDTGEQTSWMVQLTEETPPSSPIVTDTPPQMSQVYLLRHAKAVWPSPGQKDFDRTLDTAGIEAARILGQELRRSGLKPEIVVCSTAIRARQTLEY--LELEPPFVLDQSEKLFSGGPDAYLMAIRMAGLEHETANSVMLVGHNPMMEELAIALAGRGQPPSHPDFESGFPTAGLAVIRFDAPLAEVRPGTGELQAFFTPSAHH--MRHGNSGRKLNRTASHRKAMFANMAASLIEHEQIVTTLPKAKEIRPIVEKLVTLGKRGDLHARRQAISAIRDAKLVAKLFDTLAPRYGQRNGGYIRIMKAGFRTGDNAPLAVVEFVDRDTSAKGSKDLARVAAEQANEAEAA---------------------------------------------MSHFAEAVHIITTDGPAGRRGVTISAVCSVSDNPATMLVCLNRSHEFNHLFIENQVFALNTLSIGQQALSEAFSGKGELSQQDRFAMGRWQTLQTGAPVLMDALASFDCRIIATHEVATHYVIYGKVTALNMGKRRRSLIYLNRSYHGSGDMLSRFFGHRRHDLTSLSEQQILALAISSEEDDARIYLAYADGLREDFPQSAKVFEEMAEEENNHRQSLIDLHKERFGNQIPLIRREHVRGYLERKPDWLVRPLGIDKVREMAEKMEEQAYRFYTEALKLVRDASTRKLLGDLAIAEKAHESLAHRLGVKHTPDDVQEVEKQTERRQFILTYIQPGLAGLMDGSVSTLAPIFAAAFATQDTWQTFLVGLSASLGAGISMGFTEAAHDDGKLSGRGSPLKRGLANGSMTTIGGLGHALPYLIPDFWTATTIAALVVFIELWAIAYIQHRFMETPFFRAAIQVVLGGSLVMATGILIGNA---------------MAAAVLIPIVQRAKNKIRVFLPGTGTRFLLRDFQTTIAERISLSGAGVHSGTPVTMAFAPADPDTGIVFQRIDGSGGSHEIRALVSEVGTTDLSTTLANRAGVTISTVEHVMAAIAGAGIDNMVIEIDGPEVPILDGTSAAFLDAFEQAGFVRQAAKRRFIRILKTSRIEAGASWAEFRPYDGTRYEVEIDFETPVIGRQKFAGDMDETIFRKEISRARTFGFMRDVEKLWAAGLALGSSLDNSLVIGDDHSVINPGGLRFKDEFVRHKTLDAIGDLALAGAPFIGCFRSYRGGHRLNAAVLRALLSDHTAFEIVEMAGKRGQNRGASLVAVNAPVFAPWAI--MKLIVNGKTQDVSAQSLDALLRELEFEGEWLATALNGELVRSKERHACRLNEGDRIEILTPRQGG--------------------------------------MSTIDTIRRSDGAESHPARSQNSGLLHTAST----LIGWFGNAMMKRRTRLHLSELSDDLLNDVGIAPAEARREIKRFFWD--------------MSSDSVRK--PGRGRIYNSIVDTIGDTPIIRLDKFAKEKGVGANLLAKLEFFNPISSVKDRIGVALIDALEAQGKATPGKTTLVEPTSGNTGIALAFAAASKGYRLILTMPETMSVERRKMLKLLGAELVLTEGAKGMKGAIAKANELVETTPDAIIPGQFDNPANPEIHRRTTAEEIWNDTDGKVDIFVAGIGTGGTITGVGQVLKQRKPDVRIIAVEPKDSPVLSGGNPGPHKIQGIGAGFAPAVLDTHIYDEVIQVSNEDSFANARLVARLEGVPVGISSGAALTAAIEVGSRPENKGKNLVVVIPSFAERYLSTVLFEGLE-------------MPNA----VDQLFAVQNEIRKAEAEANRPQGSVTLVAVSKTFDADTIRPVIEAGQRVFGENRVQEAQLKWPYMRSDFPDLELHLIGPLQSNKTQEAVALFDVIETVDREKIAAALSLEMKKQNRFPRLYVQVNTGSEPQKAGIEPKEAVAFVTRCRDVHGLIVEGLMCIPPADENPGPHFALLEKIAREAQVEKLSMGMSGDYEIAIGFGATSVRVGSAIFGSR---MRLLILGTGHMANTHARHFIEIEGVTLAGAVDVDPARAEAFREKYSIERAFTSLDDALTWGEFDSVANVTPDSAHHATTLKCLTAGKHVFCEKPLATNYQDALEMAEKAEAANLVGMVNLTYRNVAQLQAARKLVTGGEVGKIRHVEASYLQSWLVSKQWGDWRTESQWLWRLSKKHGSNGTLGDVGVHILDFVVYGTDTEIDEVFCRLKAFDKADGNRIGEYDLDANDSFTMTVSFTNGALGVVHSSRWATGHVNELRLRVYGDKGSFEVVHRHDGSKLLACLGDDIETATWKEIEVEPVATNYQRFVEAVGQGKVLEPSFLHAAKLQKALDLANVTESDRREHRLEN------------------------------------------------MNGYVLAIDQGTTSSRAIIFDKNRKVVGKSQQEFTQIYPQSGWVEHDPEEIWQSVLSTCQVAIENAKIHAGDVAAIGITNQRETVIVWDKATGKPVHNAIVWQDRRTAPICQRLKKQGLEKTFTKKTGLLLDPYFSGTKLAWILDKVPGARKRAANGELLFGTVDCFLIWRLTGGK---VHATDATNASRTLLFNIATNEWDDELLDILRIPRAMLPEVKDCAANYGAAEAELFGATIPILGVAGDQHAATIGQACFEPGMFKSTYGTGCFALLNTGSDMVLSKNRLLTTIAYRL-NGKTTYALEGSIFIAGAAVQWLRDGLKIIGSAPETGKLADQADPSQNVYLVPAFVGLGAPHWDAEARGAIYGLTRNTGPAEFARAALESVAYQTRDLLDAMRKDWKSSGTKTVLRVDGGMVASDWTMQRLADILDAPVDRPEILETTALGAAWLAGSKAGIWPNRREFSKTWERDVQFTPKMDEKTRKTKLAGWKTAVKRTLTS--------MNELIDLDYKPTDDEPFMSDRQKSYFREKLINWKNDILREARETLEILQQENANLPDIADRASSETDRAIELRARDRQRKLISKIDAALQRIDDGTYGFCEETGDPISLKRLDARPIATLSIEAQERHERREKVYRDD---------MKAKDDNQRRSGEAAVEVTEGERPTLKTIAFMAGLGVTTVSRALKDAPEIGQATKNRVQLIAKQIGYRPNRAGVRLRTGKTNVISLVLNAQSSIMGLTSNMVYGISEVLSQTPYHLTVTPYSLENDPMQPIRYIVETGSADGIIMSRTEPDDPRVRYLTEHGIPFATHGRTEMGIEHPFHDFDNEAFAYLSVKSLLARGRRKIAMLAGPSPAVTYYRHLDRGFSRSIEEGGAVGVTFSGIDVDTSLGRIRDRMEEIMTGPNRPDGIVCCSGGAAIALVAGIEAAGLKIGEDVDLVSKQSTDVLKWFRPQIIVFNEDVRHAGRELARAVLRRIAGVDARELQSLSYPDEHELR-------MQREWKLAHGRSLTLGDRALIMGVLNVTPDSFSDGGVHYSLDHALAGARQMIAEGASIIDVGGESTRPGATRVEPAVEQSRVLPVIEALVKETDIIISVDTYREETARLAVAAGAHIVNDVWGVQREPA----------------------------------IAGLAVETGAGLVIMHTGRERERDPDVIVDQFAFLNRSLEIAKLAGVSRDQIVLDPGFGFAKDTAENISLMDRFAELSAFGYPFLVGTSRKRFLGATSGGEAEQRDVATSASSAILRLAGADIFRVHDVAMNRSALALADAVLNARRQRS-MSIRFHRNDLPNLSNYQVDAVAIDTETLGLNPHRDRLCVVQISPGDGSADVIQIEKGQKTAPNLVKLLGDDAITKIFHFGRFDLAVLFHTFGVMPKPVFCTKIASRLVRTYTDRHGLKEICSELLDINLSKQQQSSDWAAPELSQAQLEYAASDVLYLHRLRTILQQRLVRDGRSDEASACFEFLPTRSKLDLMGWEEQDIFAHSMKRSAAAATLSLFACALFALPAHA--KDKDAEFFQTIEGQWVGPGEIVAGKYKGTKFTCTLDGTTPAEKAGMTLDGSCRVGIFNQPMKATVVRAGNSYKGSFLDGSAGKGLDITGGNVSGDRVVLAISRKQLSGAMQAKFTGANSMNVTISVRLEDQMIPVVGMSLKRVDATAVGSIAKN----MMSEYYSQPFARRPSVGVDVGGIMVGGGAPVVVQSMTNTDTADVDGTVAQVAALSRAGSQIVRITVDRDESAAAVPKIRERLDRLGLSVPLVGDFHYIGHKLLADHPACAEALAKYRINPGNVGFKDKKDAQFGAIIEMAIRYDKPVRIGVNWGSLDQELLTTLMDQNHAQGAPLTAEQVTREAIVQSALISSQLAEEIGLPRSKIILSAKVSQVQDLIAVYAELARRCDHALHLGLTEAGMGSKGIVASSAALGILLQQGIGDTIRISLTPEPGGDRTREVQVAQELLQTMGFRQFIPIVAACPGCGRTTSTVFQELAQSIQNDIRNNMPTWREKYPGVETLNVAVMGCIVNGPGESKMADIGISLPGTGETPSAPVFVDGKKVATLRGAGIAQEFQKMVSDYIENRFGQ-G---AGSHAAE---------------------MNELHR-----GSCLCGSVRFETR-GALRGVVFCHCSQCRKQSGHFYAATNVQDDSIDISGEEHVSWYEASDFAKRGFCKHCGSVLFWKHHKLDYVSVMAGAFDQPSGLHGESHIFVGDKGDYYEIDDGLPQYEKSGGSVVVAGD-----MGFKCGIVGLPNVGKSTLFNALTKTAAAQAANYPFCTIEPNTGEVAVPDSRLQAIAKIGKSANIVPTRISFVDIAGLVRGASKGEGLGNQFLANIREVDAVVHVLRCFEDDDITHVEGRIDPVSDAETVETELMLSDLESLERRIVQFRKRASS-KDKEALTVLPVMEQALALLQDGKPVRFMLNGIAAEDLLILQSLNLLTSKPVLYVCNVAESDAATGNEYTEEVEEMATKQGAETVTISAAIEAEVAQLPDEEAKEYLEAMNLDEPGLDRLIRAGYKLLHLITYFTVGPKEARAWTVERGSKAPQAAGVIHTDFERGFIRAQTIAYSDYVTLGGEVPAKEAGKARDEGKEYVVQDGDIMLFRFNTMVTVLD--TIDQKRRLRHPEKAHRPDSEILKKPDWIRVKAPVSKGYSETRDIVRSHNLVTVCEEAGCPNIGECWDKKHATFMIMGEICTRACAFCNVATGLPTALDPNEPENVGKAVLKMGLSHVVITSVDRDDLADGGAQHFADVIHAIRRISPGTTIEILTPDFLRKEGALEIVVAARPDVFNHNLETVPSNYLKVRPGARYFHSIRLLQRVKEIDPTIFTKSGIMVGLGEERNEVLQLMDDLRSADVDFMTIGQYLQPTRKHHPVLNYVTPDEFKSYATIAKTKGFLVVASSPLTRSSHHAGDDFAKLRAAREALYASRSMQGILYEEPSVWLFLLVTCIMGGWAAWMTGRACARTWRPIPILVFYMLLLGIAVRFIHFALFGGTMLTLHYYVVDTIVLIVIGTLGYRYTRTKQMVRQYHWLYEKVSPFSWKERTRA----MQNTISRRGFLAAMAATAAAGLAGCAQIPGDLPVIQVDDYGNPLR------QPQMDVDPAFASYAAMYSAREDDGYQLPAIPIEKMDKRYLRQIVQDPTGEQPGTIVVDTNDKFLYLVREGGQAIRYGVGIGKEGFAWSGRAVIQWKRHWPKWTPPDEMVARQPELVKYSARNGGMEPGLKNPLGARALYIFQNGVDTLYRLHGSPEWWSIGKAVSSGCVRLINQDIIDLYDRVPNKSPVLVIMVSEVQKNTSAILRVERLSMKFGGLIAINDLNFEAKRGAITALIGPNGAGKTTVFNCITGFYKPTEGMLTLTRQNGEEYLLERMADFVITKKAKVARTFQNIRLFSGLTVLENLLVAQHNPLMLSSGFTVLGLLGFPTYRKAAADAIEKARFWLEKINLVDRADDPAGDLPYGAQRRLEIARAMCTEPELLCLDEPAAGLNARESAELNKLLLDIRAETGTSILLIEHDMSVVMEISDHVVVLEYGTKISDGTPDMVKNDPKVIAAYLGVEDEEVVELIEQAEAA-GVDDITAAVDLLSTEALHEIVEAHPEAEPVELI---PAA--------AIAEVASSKARS---------------ARK--PVAKKTQPT---------------------------------ATAAAIKAGSTSNALAGPRGGKPDKLILIKGIGPVNERKLNEHGIFHFDQIAAWKKADVIAAETYLAFDGRIAREDWIAQAKKLAKESQVKPAPKKTGGAK--MTMEDRKNAFENKYAHDAEVRFRAEARRNKLLGLWAAEKLGKTGEEAEAYAKDVIKADFQEAGDDDVFRKIRTDFDAAGIDTSDHRIRRTMEELMIEAIRQIEGS---MNDLTNEQRTALEAAAFRRLVEHLRERSDVQNIDLMNLAGFCRNCLSNWYREAAEGSGIALSKEASREIIYGEPYADWQAKHQREASDAQKAAFEANKP-----SHMTRESTR-NAQNNGGTAETGSTAREATRAVVIVPVLPQRFADGGKTDENNRSKYQHEFQRSDDARLEEAEGLARAVDLDIVHSDIVQVSAPRPATLLGTGKVETITEIIEASHAELVIVDHSLTPVQQRNLEKEWNAKVLDRTGLILEIFGRRAQTKEGALQVELAHLTYQKGRLVRSWTHLERQRGGGGFLGGPGETQIEADRRALQEKILRIKRELETVVRTRTLHRAKRKKVPHPVVALVGYTNAGKSTLFNRLTGAGVVAEDMLFATLDPTLRRIKLAHGETIILSDTVGFISNLPTHLVAAFRATLEEVVEADLILHVRDISDPDTAAQAEDVHAIMTSLGIERGDTKRIIEVWNKIDLLDDAGREAAQRLSAAAIGNEHPIPVSAITGEGVDALLKEIEDRIAGKLTKVKVTLQHNQMRLMDWIYQHSSNVKRKDLDDGSIALTMDMTANSQTMLDEKLLSRNNR-MAKEATRVRRRERKNISSGVAHVNSTFNNTMITITDAQGNAIAWSSAGAQGFKGSRKSTPFAAQMAAEDCAKKAQEHGMRSLEVEVCGPGSGRESALRALQAAGFTITSIRDVTPIPHNGCRPRKRRRV-------MTGSYRRRWRRP-STPRSFGRKVLDFALMAIFFALVALLAAKLNR-QEPVEALAGRAYVIDGDTISISGSHIRLKGIDAPELTQSCGTESVASACGKVSRQELVRLVGGREVRCEGYGQDKYNRSLATCYVGETNLNRAMIEAGQAISYGEYQDDEAQARKERKGLWVTTFETPQDWRKEHE-EMP---EQIQPQPNRPADLLDQLFDWINRLLGGLW----------MSVVEVKQGSSPIVLGFPHTGTDLPADVWNRLNENGQRLADTDWHIDQLYDGLLPNVTTVRATFHRYVIDANRDPQGISLYPGQNTTALIPETDFDGKPIWRDGEKPGPADTKIRLEQFHRPYHNALYEEIKRVRKEHGLVVLYDCHSIRSHIPFLFQGKLPDFNIGTNEGASCSPEIASAVGRIAFAARGYDAVINGRFKGGWTTRHYGKPSSGIHAIQMELTQSSHLATEAPPFALDEGKAEKLRRHLKDILEIIEQTAIKLGSR---------------MREIIFDTETTGLDRLDDRVIEIGGVELINRFPTGRTFHVYINPEGKTVHPDALAVHGISNEQLADKPLFAGVLHDFLDFIDGAKLIAHNAMFDMGFINAELNRLGHPPIHNDRVVDTLALARRKHPMGPNSLDALCRRYGIDNSHRTMHGALLDSQLLAEVYIELIGGKQAALGLTIAEQS-GNQQTSDT--AGLIVIPRRPTPLRPRLTEAEKLAHAKLVDKIGEKALWHERIPLQ-----------MKIWKSALVAGAIALLSSTASYAAKTDLVLGIVLEPPHLDPTAGAAAAIDEIVYANIFEGLTRIGSKGEVLPALAESWTLSDDGKIYTFKLHKDVKFHDGTAFDAEDVKFSLDRARGEKSTNAQKGLFAAIDTVDVVDPTTVKITLKQPNGDFLYNMGWGDAVIVAPESAETNKEKPIGTGPFEFDTWAKGSQIVISKNPGYWGTPAVLDKATFRVIPDPAAATAALMAGDVQAFPVFPSYETIAQVQADPRFKVVIGTTEGETVLGMNNKKPPFDNLKVRQAVARAIDRKALIDGAMFGLGTPIGSFFPPHHPAYLDLTSETAYDPETSKKLLADAGQAAGFKTTLKLPPPVYARRGGEIIAAELREIGIEAEIIPLEWAQWLEQVFKGKDYDMTIVSHTEPNDLNIFAR-DDYYFNYDNPAFKDLIKQIEATSDAEKRTELYQQAQKLLATDVPAAFLFQLPKTGIWDAKIEGLWENGPIQANDLTAVKWTDMRILRAGDHRQMPWKNGGGVTTEVAISSPDATVSNFDWRISMAKVPASGPFSAFANIERVLAVLDGE-MLLTIGNDSAVFMGPASPAIAFPGDTPTSADVI-GDVTDLNVMTRRGKFDAGVR--RLDQAEVVAHADETFVLFRSDAETATGEV-----LGVDDVIQLSRFERLA--FAIGPENAWLIEIRAI---------------------------------MQRSYLDYAMSVIVSRALPDVRDGLKPVHRRILHAMNEMGLSWNRSYRKSAGVVGEVMGKYHPHGDASIYDALVRMAQDWSLRDPLVDGQGNFGSIDGDSPAAMRYTECRLEKVTESLLEDIDKDTVDFQDNYDGREQEPVVLPARFPNLLVNGSGGIAVGMATNVPPHNLAEVINGCIALIDNPAIDLPELMEIIPGPDFPTGGIVLGRSGIYSAYSTGRGSILMRGKVAIESMRGDREAIIITEVPYQVNKATMIEKMADLVREKRIEGISDIRDESDRDGYRVVVELKRDAVADVIVNQLYRFTPLQSSFGANMVALNGGKPGLLNLLDILRAFVSFREDVVSRRTKFLLRKARDRAHVLVGLAIAVANIDEIIALIRRAPDPATARDQLMERRWPANDVAPLITLIADPRHTLNDDNTYNLSEEQARAILDLRLQRLTALGRDEIADELNKIGVEIKDYLEILASRLRIMGIVKDELVAIRDEFGTPRRTELTDGGADMDDEDLIAREDMVVTVSHAGYIKRVPLATYRAQRRGGKGRSGVTMKDEDSVTRLFVANTHTPVLFFSSRGIVYKEKVWRLPIGTPQSRGKALINMLPLEQGERITTIMPLPEDEASWANLDVMFATTRGTVRRNKLSDFVQVNRNGKIAMKLEEEGDEILSVDTCDEFDDVLLTAAGGQCIRFPVTDVRVFAGRNSIGVRGITLAPGDKIISMSILGHVDASPAERSAYLKQAAAERRAQGADDVEEIALVGEEATDVADLLPERYAELRDREQTVLTVSEYGYGKRSSSYEFRVSGRGGKGIRATDTSKTDEIGKLVSLFPVEASDQILLVSDGGQLIRVPVNGIRIAGRSTKGVTIFNTADGEKVVSVERISETESDD--VEAETAVQGE--------TS----SPNTTDGTGEV------------------MRSSFVPLFLLALPFLEIAGFIVVGGKIGVLATLGLVILSIFLGVFLLRLQGFGLIQRIREETAAGRTPKRELVHGVMLVFAAFLLIIPGFITDIIGLLLFIPAVRDIGWRF-ISDRVVVVSSGARG------------------SSQPGSTRIKDRVIELDPEDYSSKPDPDSPWNPKQ--------------------MGTTRTEQRNDRASGLDERSPEDILRLLYEAQVEAAASVARATDSIAKASLLAAETLRAGGRLAYAAAGSSGLMALADALELPGTYGIAPDRIVILLAGGVASLNRLAGTYEDDTAQAARDIAAAGLAEGDCLIAISASGSTPYALAAIDEATNRGLKIISIANNPDVPLFSLADVAITLPTPPEMVAGSTRMGAGTAQKIALNLLSTLMAVHLGHVHDGYMVNLTADNIKLRERAARIVSAISACDISDAARLLEASGGSVKSAILLAAGAPSAEQATEILESNQQRLRPALSK-----LKGSLGSR-------MTAPKAITTVPVKLGDRSYDILIGAGLIDQAGREIASRAK-SVRVAIVTDETVARLHLDRLSQSLSDAGIDSTPIIVAPGEKSKGFATLEIVTNAILSARLERGDVVIAFGGGVIGDLAGFAAGIARRGMGFIQMPTSLLAQVDSSVGGKTGINTAHGKNLVGVFYQPQLVLADTQVLDTLSPREFRAGYAEVAKYGLIDRPDFFSWLEKNWQGIFDGGRERTEAIAISCQAKADVVARDERETGDRALLNLGHTFGHALETATNYDSARLVHGEGVAIGMVLAHQFSAKMNLASPDDAKRVEAHLRSVGLPVSIADIPGDLPSASTLMGYIAQDKKVARGALTFILTRGIGQSFIAKDVPPSAVLSFLQERHPKMDLSGEERIAASRDAVWKALNDTEILKACIPGCESLERVSDTELDAVVGVKIGPVKARFNGKVELTNLNPPLSYTISGEGKGGVAGFAKGGADVTLTEEGAETVLAYVVNADVGGKIAQVGSRLISSTSKKLATQFFENLNAAVSGS-----------MTVSSVASEKPNDISLSLYRAIWRWHFFAGLLVIPFMLNLAITGSLYLFKDEIDNTAFA-YRNVVQPRGE-ALSPSILSDHAKAAVPGSKVLRFRAPAEPTQSARVTVGTDA-GKTLVFVDPFSGSVLGKVGEKEEFNWVVKKIHSLDYFGFAFNRIVEAVGGLALILVVTGFYLWWPRKQTGGVMSVRGTPDKRVFWRDIHAVTGAIAGALIFFLAITGMPWSGYWGDKVNTALSSAGLGYPAQLWDDVPVSHVPTKDVLTNAGWTVENAPVPTSTPNGTG-QAVGIDQIVASANAVGLAPGFEVSFPSDKPGVYTAAVFPDDIAKQRTIHFDQYTGKPLVDIKFADYGTGAKAIELGIGVHQGEYLGVANQIIMLLTCLAIMLTSVSAVVMWWKRRPSGRLGVPPMPSQRHIFATLTLVILGFGILFPLTGFAILATLVLDQLILRIPSPLKRVFS-MKLLTGLLFLLILIFAGCKTLTPEERRAIDAQSCASFGFKRGTTAFSTCLLDLELDRRADRRAQFE--QFN---T-PVIVYDG-------RYRG---W----------------------------------------MALVRNRKAIRHVDYWPGFVDALSTLLLSIMFLLSVFVLAQFLLSQEITGKDAVLNRLNSQINELTQLLSLEQGNKQDLEDTIANLQASLTSAQNEQSRLQSLLSQGAGAGAAAEGKISELSTGLENEKQISARALSQVEILNQQIMALRKQIGALEEALNASETRDKESNTKIADLGKRLNVALAQRVQELNRYRSDFFGRLREILSDRENIRIVGDRFVFQSEVLFPSGTAVLNEAGSEEMKKLAVALIDLQKEIPDEINWVLRVDGHTDNVALSGTGQFKDNWELSSARAISVVKFLIANGVPANRLVAAGFGEFQPLEPGDTPEVRARNRRIELKLTER---MSGRDPEEIFRIDPALLLRAYATGVFPMAEEADNPEIFWVRPEKRGVIPFENFHVSRSLRKVVRQGRFDIRYDTDFAGVIDGCASGSGERARTWINAPIREAYSKLFELDYCHTVEAWRDGELVGGLYGIALGSAFFGESMFSRQRDASKVCLVHLVGHLIEKGFTLLDTQFTTNHLESFGAVEIPRRQYQKMLENALAQPAEFMPQDMIRYDILAQEALRGVIRKVLGEVAKAGLPGNHHFFVTFLTGAPGVRISTRLHEKYPEQMTIVLQHQFWDLQVTDTLFEVGLSFGDVPERLVVPFSAIRGFYDPSVNFELEFDVEVEETVGDNDQG--ITSIAPAQSIDKPKVATPSAKPKPPV-KAAKKPVKPG-EKPDAASDESG-EKSSASVVSLDSFRKKK--------MARIIGTDEQAIRTAANALAEGNLVAIPTETVYGLAADATNGDAVAGIFEAKGRPHFNPLICHVSDIAMAERYALIDPLSRRLMDVFWPGPLTLVLPLRPDTGLHPLVAAGLDTIALRMPRGVAGKIIAALNRPLAAPSANTSGKISGTSASAVEDDLGLKIALILDAGPSEVGLESTIVKVDGDAVQLLRPGGLAAEDIEALIAGTLKRIDQRSAIEAPGMLASHYAPSAGVRLNVHEVRAGEALLAFGPQRVSGAE-GAAASLNLSETGNLREAAANLFDYMKRLDGSGSAVIAVEPIPFNGLGEAINDRLVRAAAPRVPPIA-LEQIKMNKGTKIAILATAALATVMVPL--TSASAD------GWRG--------------------DRYY-RHRYHNN--NSDAWAAGAVGLAAGALLGTALAQPR---EP----EVIYRD---YDDGY---YR-RPVQV--YRE--APRY--YG-GAQPWSREWYRYCSDRYRSFNPETGTFRGYDGRDYFCNANMAGIRKAAAIGGGVIGAGWVARLLLNGIDVSIFDPDPEAERKVSEVMKNSRRAYKTMVPGGLPKEGKLTFAKTIEAAVAGADLIQESVPERIDLKHKVLAEIDKHAAVDAIVGSSTSGILPSEMQTAMKHPERLVVGHPYNPVYLLPLVEIVGGKQTSPEAVEKARELYTSIGMKPVVIRKEIEAFVGDRLLEAVWRESLWLIKDDITTVEELDDIIRYSFGIRWAQMGMFQVYRVAGGE----AGMRHFIAQFGPCLSWPWTKLMDVPELDEELVDKIANQSDEQAHGLSIRELERIRDDNLVAIMEALSKQNKGKGWGAGALHNDYTKRLSKLAHKPATSKAAEKAKAGKPKKKKGMNEDLSKKRLGRGLAALIGEMDRLPEEN-APRVATLDRQVPIEFVTRNPRNPRRLFTEADLEDLTQSIREHGIVQPVVVRPSKDSADHY-ELIAGERRWRAAQRAGLSTVPVIIREVNDRVALELAIIENVQRADLNPIEEGMGYQQLIDDHEYTQADLAQVIGKSRSHVANTLRLLKLPEPVRDMITNGALSAGHARTLITAENPAALADKIIRDGLSVRQAEALAQSGGPKPAASKAKSDGEDAKDADTRALEKLLSDVTGMRVTVNHQNKGGDVRIHYATLEQLDEICRRLQS--------MGDMRTPLGKVRGLGSAKEGTDHFWRQRLTAVSNVPLMLFFIGLILSLHGAGYVETRAALSHPLTSLVLLMVVISGLYHMRLGMQAIIEDYVHGEGMKIVLLMLNTFFALAVGVACIFAILKLSFGGMVFKTGRPVLRSATAAIALIAFAATSATPAFVTVA----RAQTQPVPVPNSEQPRAQGPASVADLADGLIDAVVNISTSQTVKGQDG--DSGPIPMPKVPEGSPFQEFFDEYFGKQ-NPGQNNPSHKVQSLGSGFVVDAQQGIIVTNNHVIADADEIEVNFNDGSKLKAELVGKDTKTDIAVLKVDPKKHKLVAVKFGDSSKTRIGDWVMAIGNPFGFGGTVTVGIVSARNRDINSGPYDNFIQTDAAINRGNSGGPLFDMYGQVIGINTAIISPTGGSIGIGFAIPAELASGVIAQLREFGETRRGWLGVRIQPVTDEIAESLGMPSSKGALVAGIIDGGPVANGSILAGDVIIRFDGKPVNNVKDLPRVVAESPVGKEVDVVIVRKGKEETVKVTLGRLEDSEQ--QAKKDDQTGQGED-GAVQ-----NVDVLGMTLDTLNDDTRKSFGISKEVQGVLITEVQNDSVAANKRIQAGDVIVDIAQEAVSSPDDVAARIEKLRDEGRKNALLMLASKTGELRFVTLQMDMPIFTLDGHAPQFEHPKSNWIAPDATLIGKVYLGENVSIWFGSVLRGDNELIHIGRNTNVQEHSVMHTDMGYPLTIGAGCTIGHRAMLHGCTIGENSLIGIGAIVLNGARIGKNSLVGAGALITERKEFPDNSLIVGSPARVVRTLDEAAIEQLRLSAAHYVENGKRFMRGMEIA----MQLYRDDSSPNPAATGKYYSVKVQLYGRFMLEDRTEHVCQIEEMSPGDVIITTDVLANNGERVIAYIDHIGRVEGTVERMVRGGFLLSLVASDRKKNKIAAQLTWLANKHELDMPEDRRHQRIAPRNPITTLAMADGRQYPCRIIDLSISGAAIEIRMRPALNSQVILGSMRGRVVRHFEEGIAIEFAIVQSREAIDIAFEH-MSSVNTRTAKIERSTNETSIAVSVNLDGTGKFDISTGVGFFDHMLEQLSRHSLIDMDIKAKGDLHIDDHHTVEDTGIAIGQAIAKALGDRRGINRYASLDLAMDETLTRAALDVSGRPFLVWHVEFSAAKIGTFDTELVREFFQALAQNAGITLHVSNLYGANNHHIAETCFKAVARTLRTAMETDPRQKDAIPSTKGTLNG------------------------------------------------------MTAFLAVFAAAVPAHAVDPLYEAKLLRLAEVIGSIHSLRNLCGEKSSQWRDRMDSLLLAENPDPARKARLIASFNRGYRTFNETYSSCTSQAIAAIDRYQAEGMRLSSEIVSGYGN------------------------MVDNVVLVGCGNMGFAMLKGWLDAGILKPDQVHVIEPTDALRERAATLGVHAHANASRLDEDLNPRVILIAVKPQVMRDVLPAYERFADDATFVSVAAGIKVALFEQYLGEKAAIIRTIPNTPAAIGKGMIVTFRNAHVSDSDAEFVDQLLKTSGKVAAVDDESLIDVATAVSGSGPAYVFHFIECLTEAAVTAGLERKTAELLAMQTVYGAGVLAASSEDTPTKLREQVTSPKGTTAAALDVLMGNDELKRLLTKAVDAAHQRAIELGSQ--------------------MTDLHSVSIKDLSLNFGSISVLRDLNIEVADGEFLVLLGPSGCGKSTLLNCIAGLLEISDGQIFIKGKNVTWEEPKDRGIGMVFQSYALYPQMTVERNLSFGLRVAGLPKPEIQKRIAAAADILQIGPLLQRKPAELSGGQRQRVAIGRALVRDVDVFLFDEPLSNLDAKLRSELRVEIKRLHHKLANTMIYVTHDQIEALTLADRIAVMKGGLIQQLDDPLTIYNKPRNLFVASFIGSPAMNFVKGEIATDDGEPVFRSADLDVSLATYTAHDKLAPGHSVIFGFRPEHITIGDKPGPI-GRSCPAVVDLDEPMGADSLVWLKVAGKAISVRVDSGRRYQPGEKVFLTFNPAMASVFDAETENRL---------------MARQRKRKGRPVSGWLIFDKPKGMGSTEAVSKIKWLFKAEKAGHAGTLDPLASGMLPIALGEATKTVPYVMDGTKIYRFTVTWGAERTTDDLEGEVTVISDNRPDEAAIRALLPKYTGVIAQIPPKFSAIKIAGERAYDLARGGEEIEIPAREVEIDRLDLIECTDASTATFEVECGKGTYVRSLARDMGRDLGCYGHISDLRRIEVAPFDESDFVTLAELEAVHPPVPTE-ETSDDRSYERL-AER------FSVMDAFLIDTGAALESLPQVPVSDDQAHRIRMGNPVILRGRDAPVEADEACVTSKGKLLAIGFIEQGQFKPKRVFTA-MSHSALPRPAIARRSKALSGDIRIPGDKSISHRSFMFGGLAAGETRITGLLEGEDVINTGRAMQAMGAKITKEGDVWVIQGTGNGCLLQPEAPLDFGNAGTGARLTMGLVGTYDMETTFIGDASLSKRPMGRVLNPLREMGVQVTASEGDRMPLTLRGPKVAAPITYRVPMASAQVKSAVLLAGLNTPGITTVIEPVMTRDHTEKMLQGFGANLSVETDRDNVRHIRIEGQGKLTGQTIDVPGDPSSTAFPLVAALLVPGSDITIRNVLMNPTRTGLILTLQEMGANIEILNARLAGGEDVADLRVRASELKGVNVPAERAPSMIDEYPVLAVAACLASGETVMNGLEELRVKESDRLSAVANGLKANGVDCTEGPESLSVRGRPDGKGIGGGTVETHLDHRIAMSFLVMGLAAEKPVSVDDSTMIATSFPEFMDMMTGMGAKITEHNAAD------------MILSLFSRKAKANEAITIALYDAIVAAARQPYFYSDLDVPDSPLGRYEMLSLHVFLFMRRIKGRTPALKSIGQEVTDEFFRDVDHSLRELGIGDSGVPKRMKKLARMFYGRIESYDKALENNDYVALVAALARNVRPGHESWPGADSLGKYVLLATRHLEDQTDDMIAGGKVTFPDAGPALEEA-------MAKTSAVEKNKRRGLLVKRFAAKRARLKAIVMNQSLPLDERFRATIQLAELPRNSTKVRIRNRCEVSGRPRGFYRKLKMSRIALRQLGSLGQVPGVVKSSWMARQFIYHMAGLNKAYGTKKVLENIHLSFYPDAKIGILGPNGAGKSTILKIMAGMDKEFTGEAWLADGATVGYLAQEPVLDPTKDVMGNVMDGVADKKAILDRYNELMMNYSDETAEEGAKLQDLIDHKNLWDLESQVEMAMEALRCPPGDSEVTNLSGGERRRVALCRLLLSQPDLLLLDEPTNHLDAETTAWLEKHLREYAGSVLLITHDRYFLDNVTGWILELDRGRGIPYEGNYSAYLGAKAKRMLQEGREDDSRQKALEREREWISASPKARQAKSKARIKAYDALVDAANDQRPGDAQILIPAGERLGQVVIEAEGLTKSFGDRMLIENLTFKLPPGGIVGIIGPNGAGKTTLFKMITGQEKPDSGSIRVGDTVHLGYVDQSRDHLKADKNVWEEISGGNEIIKLGKYEMNSRAYVSAFNFKGSDQQQKVGNLSGGQRNRVHLAKMLQAGGNVLLLDEPTNDLDTETLAALEDALEKYAGCAVIISHDRMFLDRLATHILAFEGDSHVEWFEGNFEDYENDKIRRLGPDSVNPKRPTYKPLTRMQKARLVILIVAVAAAGAAGYIATRMKPTTVVVENAK---PQIKLQDILVATENLGVGAEMQGQMRWQAWPEDALADGYIKRTDRPNAEEELRGSTVRLQIFPGEPIREAKLIGKGQSFMSAQLPAGMRAVATQIAAETSAGGFILPDDYVDVIMIRQM-N--DE--MPKQFTTETILRNIRVLAIDQTIQEDETGQKTKVGSTATLELTPEQAEILAVAEQMATRMTLALRSLQDAQEATGPGAEYLVNSPGRAGNVKLIKSGT------------------VIEILGRK----------------------------------MVSK--------QTLRIVHCFRAPVGGIFRHVRDLIEAQVKAGHQVGIICDASTGGALEEALLADLRGKLALGLERIAMQRQIGPGDAMAALRTYKIIKKLQPDILHGHGAKGGIYARLFGSVL-RVFGSRVARFYSPHGGSLHFDPAKLQGRIIFRVERTMERMTDRIIFVSAFEQGIYVRKVGEPRCASELIYNGLADGDFEPVATDDDSADFLFIGEMRALKGPDIFINALARASAACGRQLTGVMVGDGKDRARLIEQSAKVQTDRSIRFLMPMKARQAFRLAKVVVIPSRAEALPYVVLEALAAGQPVIASRVGGIPEILGDSSPALVEPEAGDLAAKMAIAIDDVAAYRQTLPSVEELKHKFSIETMAARLEAEYFAALG----MSQLAFETENQSGESH---DRPAGSGLHAFRPIPRISIQAFCETDAVARQVGRASEDRRMAKAHTQVHMGGVERAVELFQTVPTPNLILLETSAAPRDMLQSLTALSEVCDPSTKVVIIGHFNDVWLYRELIRNGISEYMVAPIDLSDIVGVIGGLFVDPEAKPLGHAIAFIGAKGGVGASTIAHNIAWSISSLFKNEVVIADMDLPYGTANINFDQDPAQGIAEAVFSPERIDDVYLDRLLAQCAEHLSLLAAPSTLDRVFDFRDDSFAHLIDVAQRSVPHVILDVPHTWNGWTRTTLARADEVVIIASPELANLRNTKNLLDTLKQLRPNDAPPRLVLNQVGVPKRPEIAPLDFCSPLGILPMATIPFDPALFGSAANNGRMLAETDAASPIVQSINEIAHILTGRMTLKAKKKPGLTSVLSRLKRKK--MPRANSVGNDVDIDVGALFASLWQNKVRIILGSLGLALLAFLVLSLVSPKYRAETRILIETRESVFTRPANQQQGDERPILDPEGIKSQVELIGSSDLLKRVITKLDLGKNEELAAETQPSLISQLFGTVGLGKGMDAATRDDYILQALRERLLVYSVQNSRVIVIQFSSKDPALSAAVPNAIADEYIATQQASKSQSDADATDWLQPEIADLSKRVKDAEAKVAAYRSSSDLLIGQNNAVLATQQLSELSSELSRVRANKAASEAKAESVRSALAKGAAVETLPDVIASPLIQRLRERQVQLNNDIADQSAALLSGHPRIRALRSQLADLNQQVRLEARKVLGSLENEAQTARLREAELTRSLNQLKAQSSKAGEEEVELRALEREATAQRQLLESYLTRYREASSRTDRGYLPADARIFSRADKPIEPYFPKIIPMTIAAFVASLLLLSIGTLLSALFSGRALRPAYTQTP-PVDKLDEVKEVLPVQAAVIPV---------APEVPEKQTF---IEEPVRPEEATPDMDVASAVDDDPA--VDDQVEAPLIHPDHS-GIANVADRLMASGAARAVVVSPEGDEASALTILLVRELADRGVRVILIDMTGSGEIGRSMLDGKELAGITNLLVSDKHFSDVIHPDHYSEAEVIPLGNHDPVKAMQSVGRLPMILNALQSAYDLVVIDSGPATATELKPLLADGTELVLALVDPDDRDVAATAKSVFEARLIEPELLTPFRPNLLRVDSGRIIRSKAMAGA--MCGRFSLLGTPDEIEALFDLMDIGTFPPRFNIAPTQPILMVVSGETPPPGSNRPDRRALLVRWGFIPGWVKDTANYPLMINARSETAIEKASFKTAMRHRRALIPASGFYEWRRDGDRKSQAYWVRPRDGGIFAFAGLYEPWANAEGSEMDTGAILTTGASENLRFIHDRMPVVIERQDFARWLDCKTQEPRHIADLLKPAQADFFEAIPVSDKVNNFANTGPEIQERVTET-QEVRKKPGRAKPPEPDD----QMKLFMNLTPREKDKLLISMAAMVARRRLERGVKLNHPEAIALISDFVVEGARDGRSVADLMEAGAHIINRDQVMEGIPEMIHDIQVEATFPDGTKLVTVHEPIR--MKSLIDSFDTPREKILDTVKQSLDGADDGELFIEYRESEGLAFDNGRLKNGSFNQDQGFGLRAVAGEAVGYAHAGELSLGALKRASDAASAVRTGHAGTYTAAPPGTNRSIYSDENPLGSPTFEAKVKLLQEIDAYLRGKDPKVRQVSVSLAASWQQVEILRADGHFVRDVRPMVRLNVSVVVGDGDRQESGTYGAGGRKGFGEFIATDKWQYAADEALRQALVNLEAIPAPAGTFDVVLSSGWPGVMLHEAVGHGLEGDFNRKKTSAFAGLLGQKVASKGVTVVDDGTITERRGSLTVDDEGMPTNKTVLIDDGILVGYMQDRQNARLMGMDPTGNGRRESYAHAPMPRMTNTYMLGGDKTPEEIIASVKKGIYAVSFGGGQVDITSGKFVFGCTEAYMIENGKLGAPVKGAMLIGNGPDAMQRISMIGNDLQLDTGIGNCGKGGQWVPVGVGQPHLRMDQMTVGGTAVMSRIGKKPVAVPQGVTASVEGQTIKAKGPKGELSFVANDDVVVKFEDGAVSVNPRDNSKLARSKWGMSRTMVVNIFTGVKDGFEKRLEISGVGYRAAMQGKNLQLSLGFSHEVIYQVPEGITVAVPKPTEIVVTGIDKQQVGQVAAEIREYRGPEPYKGKGVKYAGEKIVRKEGKKK--MAEKKPLTIRLCGPRGFCAGVDRAIQIVVLALKKYGAPVYVRHEIVHNRYVVEGLQSRGAVFVEELNEIPAEHRNQPVVFSAHGVPKSVPADAESKNLFYLDATCPLVSKVHKQAMRHQRLGRHVILVGHSGHPEVIGTMGQLPEGAVTLIETIEDAETYSPQDPDNLGFVTQTTLSVDDTAGIIATLQRRFPELTAPAAESICYATTNRQDAVKSAAPGCDLFLVVGAPNSSNSKRLVEVAERSGAAQSLLVQRASDIDWTQIGDISVVGLSAGASAPEIIVDEIIQSFSERYFVTIDLAETTIETENFHVMRDLRDMKLTSDDMAFVNGSH-MDKRDLNLDLMVDTLKAAAEPSRLRILALLSRGDLTVSDLTSILGQSQPRVSRHLKLLFEASLINRYQEGSWAYFRLADSLIVGDIARSLLERLDSSDLLLERDLERLSSVKLQRRERAAAYFSANATSWDVIRSLHVPDGAVEDALLKIIGRKPFQAMLDVGTGTGRLLELFAPLYVRGVGIDINRDMLTIARSNLDRDGITNAQVRHGDVYSLPVDRESFDLVTIHQVLHFLDNPADAIREAARALRAGGRMLIVDFAPHELEFLRDNHAHVRLGFADEQMREWLTEAGLMLEDSLQLEPKGKDK-LTVKLWLARDPRLLIADPTLTS--RVTESV----MDENSNKSTAEPSPQKRKLSDRTIAISCLAFFFGMVGMAFAAVPLYAMFCQVTGYGGTTQRVEQMSDTILDKKIIVRFDANTSGGMPWHFEPVQRDVTMNIGETTLIKYEARNITDKPTAGRASFNVTPQAAGAYFNKVECFCFTDTVLKPGEDLEMPVVFFVDPDIVNAPELKGVNTITLSYTFFPIAMPAPVAANADVKKN-ASGQL--MNEQ------------SPAIPEAVEK-VDL--------PDLPTSVDRFVNREFSWLQFNRRVLEEAHNKHQPLLERLRFLSISAANLDEFFMVRIAGLAGQVRAGVSDRSDDGRTPQEQLEFVLEEVGRLQQAQQIRLNELRSEMLHEHIEIVRPDRLTKGEQAWLEDHFLETIYPVLTPLAIDPAHPFPFIPNLGFTIAMLLNRSTDNRPMTALLRLPQALKRFIQIPDEGGRFRFLTLEDAIGLFTGRLFPGYEVKGAGTFRIIRDSDIEVEEEAEDLVRLFESALKRRRRGQVIRIEFDDEMPETLRSFVATELGVPDNRISVLNGLLALNMISEIVNIPRADLKFVPYNPRFPERVREHGGDCLAAIREKDIVVHHPYESFDVVVQFLRQAAADPDVVAIKQTLYRTSNDSPIVRALIDAAEAGKSVTALIELKARFDEEANIRWARDLERAGVQVVFGFIELKTHAKMSLVVRREEHRLRSYVHLGTGNYHPITAKIYTDLSFFTCEPSIGRDVAQIFNYITGYTPPSGEMGIAVSPISLRPRILQHIAEEIEHAKAGRPASIWMKVNSLVDAEIIDALYEASRHGVEIDLVVRGICCLRPQVPGLSDNIRAKSIVGRFLEHSRIFCFGNGHSMPSEKALVYFGSADMMTRNLDRRVETLVPITNPTVHQQILSQIMLANLLDNQQSYELLADGTSRRIEPADGDEPFNAQEYFMTNPSLSGRGKSLKSSAPRLIAHRKRSK--RNKAAMTTVAEVSIDRGADKRQRRWHILVFLAPAVLVYTAVMILPLIETLRLSLFNVKDGQSVFVGFGNFQVLFGDPRWAASFWNALRNNFVFFLIHMLVQNPIGVALAALLSVPKLRFGAFYRTAMFLPTLLSFVIVGFIWKLILSPIWGVSPYLMDLVGLKSMFSPWLGKPGTALIAVSLISVWQYVGIPMMLIYAAMLNIPDEVLEAAECDGVTGWSQFWKIKLPLILPSIGIISILTFVGNFNAFDLIYTVQGALAGPDGSTDILGTLLYRTFFGFQLQIGDKSMGATIATVMFLIILSGVCLYLFAIQRRMRRYQF-MSEHEADPLFDEKP--QVRGLTGSRIPMRYLAPNVITVLAICAGLTGIRLAFENRFELAVSMVLLAAFLDGIDGRIARMMKGSTKFGAQMDSLADIVNFGVAPALVLYAYM-LDQARSFG--------WIAALLYCIACCLRLARFNVMLDVVDKPLWQNNFFTGVPAPAGAMLVMLPVYLGFLGLAPTRTLAFTAAAY-----TVGVAILMISRLPVYSGKAAGTKLRSDWVMPTFLFIVVYVAF-----LMSFTWETLTLTTIAFFIT-------------LPF------------------------SARAWKRFEAADAA---SLMSS--EKDGSEAVS------------------------------------MRIVGGKFRGRALATPASNSIRPTTDRTRESLFNILVHNYPEKFESTRVLDLFAGTGALGLEAMSRGARYGVFIEESTEGRGLIRTNVETFGLLGNTKIFRRDATKLGEAGTIEPFDLVFADPPYGKGLGEMAFKSALDGGWLNPDTLLVLEEEAEAMVDLDPRFSVVEERPYGGTVIRLVTLKT----------MLGVGLIGTGFMGKCHALAWNSVRAVFGDVPPVRLVHLGEANAELAVRRANEFGFREGSGDWRAVIDDPEVNVVSITTPNQFHPEMAIAALKAGKHVWCEKPMAPAYRDAEAMLA-AANASGKVAALGYNYIQGPAIRHIRKLLAEKIIGDVNHLRIEMDEDFMADPDALFYWK--HEATSGYGALDDFAVHPLSLLHILFGRVSRVMCEMSKPYPARKTQDGGSRAVETYDSASVLMHMENGVAGTLQVNRSAWGRKGRIALQIFGSKGSILFDQERANEFQLYVTADRATEQGYRTILTAPHHEPYSQFIPAPGHGLGFNDLKIIECHELIKRINGQ-PAHLIEFADGLEIERTVHAMASSFHEQRWVDVRMSHAIVP----SSGVLVGRARVTGHDHPRIVTVRDGKVFDITNKTAPTVRDIVELGNPAAYVAGSTGAALGDVDAIVQNSWTKKFDPAVPALLSPIDLQAVKASGVTFVASLLERVIEEQAKGDKAKADSLRDDVLKLIGADLSKIVPGSPAAMQVKEALIARGVWSQYLEVGIGPDAEIFTKGQPMSTVGFGADVGLHPISSWNNPEPEIVLVVSSAGRIIGATLGNDVNLRDVEGRSALLLGKAKDNNASGSVGPFIRLFDGDFTIETIKAAELSMRVEGEDGFVLDGHSNMAKISRTPESLVEATIGRHHQYPDGLVLFLGTMFAPVKDRDGPGKGFTHKIGDVVSISTPSLGTLSNRVLLSTECAPWTYGASHLMRDLGKADLLMSGLFQSIGRRMLFSLDPEDAHGLSIKALKTGLVPACVPNNDPALRVAVAGLSFPNPLGMAAGYDKNAEVPDALLKLGFGFAEVGTLTPLAQSGNPKPRIFRLVEDKAVINRLGFNNQGHEPAFRLLTERRSHAGIVGVNIGANKESADRVADYVMGIRKFHSLASYFTVNISSPNTPGLRDLQARESLKELLGAVLEERD---AQPGVRRPVFLKIAPDLAETSLDDIAAEVALHPLDGLIISNTTLSRTGLRSSRNVGEAGGLSGTPLFERSTIVLAKMRQRVGPELPLIGVGGVDSAATAIAKIRAGADLVQLYTSLIYHGPGLAGEIVKGLSAALMRDRITTIAALRDCDVADWAKKPIPAMPLKADLDAIDWKILKQLQDNGRITNVELAERVGISAPPCLRRVRKLEESGVIQGYRAILNGSILGQDIVAFCMIRLHRQSDADLKSFAEKTRDWILVRRAWMVSGESDFLLHCVASDLSTFQNFVIEELTSTPNVDSVRTSLTIRSVKNEPLMVLMGVEISRLSNGLTIATETMPHVESVALGIWVKAGSRNEASNQHGIAHLLEHMAFKGTENRSAWQIAADIEDVGGEINAATSVETTSYYARVLRDDMPLAIDILADIMTGSKFDAEELEREKNVVMQEIGAAHDTPDDVVFDRFTEAAFQQQTIGRTILGTPETVQSFSSADLRRYMDEQYSAERMVVVAAGGVKHDEFVREVEKRLGSFRSKSTAPEPDASHY-VGGDFREQRELMDAQVVIGFEGRAYHVRDFYASQLLAMILGGGMSSRLFQEVREKRGLCYSVYAFHWGFSDTGVFGIHAATGRSHLKKLVPVIINELHAAAQNISQEELNRARAQYRASLLMSHESAASRAGQIARQMLLYGHPVSTEELVDRLSKITTERLTDLAGRLFLDTTPTIAAVGPVGSLMKFADVRHGLTAPTSSPRKIAV-MTQSAGSAS--PSSQRWMKLAVVGSAGLVIAGGLAFYYASK---GTKSGNSADTIVVTIKDGTCTPNAITVPAGRSTFTIVNNSDRALEWEILDGVMVVEERENIVPGFSQTLQAKLKAGDYEITCGLLNSPRGTLHVTPSAEADAEAAKPALRAYLGPLAEFQVFLVTQANALVKATQALDDAIKAGDLEKARESWQKARAPYSQIEPMADLFADLHNAVNPVADYLEKREQDPGFTGFHRIEYGLYASNSLDGLAPVSAKLLADVSALKDRMRGLRIPPEQIAKGAARLLSSIAETKIIVGEDHYAKTDLADIEANLAGVSRMMALLKPVAMPAAPEAVTDIDSRMADADSALKALRGPDGYPPFDNVNAESRKTLAQRVQALAEAIGKLNAAVGLE-------------MSDGEAR-----ERDYQIGSASFRARPLEPALYIVATPIGNLGDMTIRGIETLAAADVIACEDTRVSRVLLDRYGISRRPYAYHEHNADQAGPKLIEALLAGKSVALVSDAGTPLVSDPGGRLVPEAKAAGIRVVPIPGASSVLAALTASGL----------FKDSFYFAGFLSSKQGQRRTKLESLKALDTALVFFESPNRAVATLADMVDVFGP--EREASLCRELTKTYETVVTLPLKDLAS--EFDGEDRIRGEVVLVIGP--PLVDENVPRSDDDIDTLLRALVLEMPPAKAAGEAARMTGRPKGELYQRLLAMK-----------MILCCGEALIDMLQRKSDAGEPAFAPYVGGAVFNTAIALGRLGVATEFFSGISSDFFGQMLLDSLAANHVGTAYAHISPQPTTLAFVRLTDGQASYMFYDENSAGRSLAIEHLPELDDNVTALQFGAISLIPEPCGSTYEALMAREHEKRVIILDPNIRPGFIPDKEMHLARMRRMIAMTDIVKISDEDMRWFGESGTLDEIARRWIVPAGRQGPKLILVTHGADGVTGYTADLTVQVAAQKVPVVDTVGAGDTFTAGVLASLEEAKLLSKSALNALSEAQIREALLLGVRAAAVTVSRAGANPPWREELAI-----MTDIA--TPARRH---RFPWIT----LIIGAIVFAILIALGTWQVERLYWKEGLLAEIEARTHAAPASLADTEKVWADQKDVDYRTVTATGRLLNDRERHYFATFDGYSGFYVYTPLLLNDGRSVFVNRGFVPYDKKDPGRRAEGEIEGEVTINGLARNPLSTKPSSIVPDNDLATNTYYWKDLATMAGQSGIEPDKLVPFFIDADKAANPGGLPIGGVTIIDLPNSHLQYALTWYGLAATLVAIIGVWLRRRYKFPSDGEPEVGQSESSDLTSRMSSTKTAINPALTEWTGPLGLPDFTAFSDDDFAAAFDAALASDLADIDAIVNHHEVSTVDNTLKALQLSGKDLDRVSAIFWLRAGAHTNDAIQALERVIAPKMSRHSSSIMMNPLLFSRIDSLYEQRDLLGLDHETDRVLEKTWKGFVRSGARLDEKGKARLADINERLASLGAQFGQNVLKDEAEWALFITDETELAGLPDFVRDAMRGAAEERDRPEAWAVTLSRSIIEPFLSFSENRALREKAFRAWAARGENGGETDNTAIVAEMVKLRAEKAGLLGYQSFAAFKLDDTMAKTPKAVMDLLEPVWDKARARAAEEEIDLQRLIAGEGRNHKVEPWDWRYYAEKLRNERFAFDETELKPYLQLEKIIEACFDVATRLFGIRFQEKTGIPTWHPDVRVWEVLNPDGSERGLFLGDYFNRQSKRSGAWMSALQSQYKLDGEHKPIIYNVMNFAKPPRGEPALLSLDGARTLFHEFGHALHGLLSDVTWPAVSGTSVSRDFVELPSQLYEHWLTVPAILETYAIHYRTGEAMPRALLDKVLAANTFNAGFNTVEFTSSALVDMAFHADGTPPTDPIRFEADTLKQLQMPDAIIMRHRTPHFNHVFSGDGYSAGYYSYMWSEVLDADAFKAFEETGDVFNADLAAKLKRYIYSAGGSRDPEELYKAFRGKMPTPDAMIEKRGLGMTAL--DIGSNAPDFTLPRDGGGTITLSGLRGNPVVLYFYPKDDTSGCTQEAIEFSGLKPQFEKLGAKVIGMSPDPIKKHDKFKTKHELKVDLVADEDKSIIESYGLWVEKSMYGRKYMGVERTTFLIDSGGKIAKIWNKVKVPGHAAEVLEATRMLQT-------MAQNSLRLVEGNSVDKTKALDAALSQIERAFGKGSIMRLGRNEQVVEIETVPTGSLSLDIALGVGGLPKGRIVEIYGPESSGKTTLALHTIAEAQKKGGICAFVDAEHALDPVYARKLGVDLENLLISQPDTGEQALEITDTLVRSGAIDVLVVDSVAALTPRAEIEGEMGDSLPGLQARLMSQALRKLTASISRSNCMVIFINQIRMKIGVMFGSPETTTGGNALKFYASVRLDIRRIGSLKERDEVVGNQTRVKVVKNKLAPPFKQVEFDIMYGEGVSKTGELVDLGVKAGIVEKSGSWFSYNSQRLGQGRDNAKLFLKENPEIANEIELALRQNAGLIAEQMLDKG-PDDVEDD-GPDAAEGMVKWVYTFGDGKAEGSAGDRNLLGGKGANLAEMCSLGLPVPPGFTITTEVCSWFYENSRQYPDSLEADVKTALTHIASLTGRTFGDKSKPLLVSVRSGSRASMPGMMDTVLNLGLNDETVEAIARETGDERFAYDSYRRFIQMYSDVVMGLDHSVFEEILEDTKANLGYEVDTSLSAADWKGIIALYKAKVEEELEQPFPQEPERQLWGAIGAVFSSWMNTRAITYRRLHNIPENWGTAVSVQAMVFGNMGDRSATGVAFTRNPSTGEKKLYGEFLVNAQGEDVVAGIRTPQNITEEARIAAGSDKPSLEKVMPQAFAEFCAVSERLEKHYTDMQDLEFTIENGKLWMLQTRSGKRTAKAALKIAVEMADEGLISRDEAVLRIDPASLDQLLHPTIDPNAKRNVIGSGLPASPGAASGEIVFSSEDAEQLKSEGRKAILVRIETSPEDIHGMHAAEAILTTRGGMTSHAAVVARGMGKPCVSGAGSLRVDYRNETLLAMGVVLKKGDIVTIDGGTGQVLKGTVPMLQPELSGDFGKIMEWADVARRMRVRANAETPADARMARSFGAEGIGLCRTEHMFFEGERIIAMREMILADNEKGRRAALDKLLPMQRSDFVELFEIMNGLPVTIRLLDPPLHEFLPKTEEEIAEVAAAMNVNAEKLRERTESLHEFNPMLGHRGCRLAISYPEIAEMQARAIFEAAVQAAKSTGAPVEPEIMVPLVGLRAELDFVKARIDAVAADVMKEADIKINYLVGTMIELPRAAVRAHSIAEVAEFFSFGTNDLTQTTFGISRDDASSFLMTYQTKGIIEQDPFVSIDVDGVGELVRLAAEKGRATRPNIKLGICGEHGGDPASIHFCEETGLDYVSCSPFRVPIARLAAAQASAKSK---------------------------------------MSEGMNRFLGDTPGRVLVKLILVSLVVGVVMNAFDWSPMDILYGVENFVRRIWNLGFGAIERFAAYFLLGAVVVIPCFIILRLLSYRRMKANIHPDYHTIKVVMTDGTEYMTRSTWGKEGDTMNLDIDPSTHPAWTGGSQQLTDRGGRVSKFKSRFANLGI------------------------------------------------MNYEVYDLRGLNCPLPVLKTRKKLQDLAPGTRIWVETSDPLAVIDVPNFCLTDGHRLIETVSITGGHRFHIERG---MAGHSQFKNIMHRKGRQDAVRSKIFSKLGREITVAAKQGLPDPAMNPRLRLAVQNARAQSMPKDNIERAIKKAAGGDADNYEEVRYEGYGPGGVAVIVEALTDNRNRTASNVRAAFTKAGGAMGETGSVGFMFNRVGEITYKPEAGSADKVMEAAIEAGADDVSSDEDGHVILCAFEDIGEVSKALEGVLGEAESIKAIWKPQTTAPVDEEKAQSVLRLISTLDDDDDVQNVYANFEVSDEVLAKLSAA--MRLGGRLQAAIEVLDDIENRKRPASDALKDWGLSHRFAGAGDRAVIGNIVYDALRRKLSLGWRMDSDAARHIAFGVLLSDAELDIEDINTALEGDKFAPEPLEAERMASWGERDLSSAPDHIRADVPEWCIPHFQAIFGDRWVEEAAALSDRPPVDLRVNTLKADRDKVLKELARAGANPAPLLETAVRVPPLRAMGRHPNVQAEPAFQRGLFEVQDLGSQLAAKLSSARAGEQVLDYCAGAGGKTLALAAEMGNKGQIHAYDAERARLAPIFDRLKRAGVRNAQAHANVGDLAPLEGQMDLVLVDAPCTGSGTWRRRPDAKWRLSDQQLERREVEQREVLDAAKAYVKPGGRLVYITCSLFAPENGNQVATFLEANSDYSRVDTRALWDQAVSAKTELKPLFKDGTAILSPLSTSTDGFFISVLRREK-----------------------------------------MSKQAK-GNKPAAWPESAPLLVDVAMGRRHADMVVRNGRLVSVYSGEIIAGMDIAIVAGRFAFVGHGVEHCIGPKTKVVDAGGRYLVPGLCDAHMHVESGMVTVTEFTRAVIPHGTTSMFIDPHEIANVLGLEGVRLMHDEALAMPINVHVQMPSCVPSAPGLEHAGAVITPADVAEAMTWPNIVGLGEVMNFPGVANNDPTMRGGIDATVKAGKTVGGHFASPELGRAFHGYVAGGPEDDHEGTRMEDAIARVRQGMRAMLRLGSAWYDVASQIKAVTEQGLDPRNFILCTDDSHSGTLVNEGHMDRVVRHAIAQGLKPVTAIQMATLNTAQHFRLEREIGSITPGRLADFLIVSDLANLTIDRVYARGLHLAEKGKLITEIPTYNYPSFAKNTIKLGKKLNARDFDIKAPKPARHLTARVIGVIENQAPTKALEADLAVSDGIVQMDRRNDVCQIALVERHRGTGAVVNGFVSGFGYTLDCAMASTVAHDSHHMIVVGTNKDDMAKAANRLGQVGGGVVLFSKGRELALVEMPIAGLMSDQRAEIVAEKADKLVDAMRAMGCSLNNAYMQHSLLALVVIPELRISDVGIIDVRTFEKVDLFLMKKILLGLAAAGLGTMLA---LGGAYAAEEAGHNAAEPTHFPIEKPAELDWTFAGPFGHYDKQQLQRGLKIYKEVCSACHSMSLVAFRSLEELGYTEAQVKAFAAEYEVPDAPNADGEIVNRKAIPADHFPSPYPNAQAAAAANGGAVPPDFSLIAKARAVERGFPLFVADI--FTQYQEGGPDYIHALLTGFGQTPPEGMQIPEGTHYNPYFIAAKSLAMAQPLQDGQVTYDDGSPQTLDQYSKDISAFLMWAAEPHLEDRKKTGFRVMIFLIVFAGLVYIAKRRIWADVEH-------MTIKVGERLPDAVFKTKTDEGVIEVLSDTLFKGKKVVLFAVPGAFTPTCSMNHLPGYLENRDAILAKGADSIVVVAVNDPHVMGAWAKATNGEGKITYLSDGNATFTKAIGLDIDLAAVSMGVRSKRYSMLVEDGVVKQLNVETTPGQAVTSSAATILEQLMARF-ASTGPTQRQLRVSEQVRHAVSQVLQRGDVRDDTLENAVIAISEVRMSPDLKIATCFVSPIGATDSDGVIGALNKNSKFIRGRAATYLKQMKYMPEFRFRIDTSFDNYAKIDALLRKPEVTRDL--ADDDEDD-GG--DPNPGDVPGH--DGND--MKLNDLSENPGATKARKRVGRGIGSGSGKTAGRGVKGQKSRSGVAINGFEGGQMPLYRRLPKRGFTNIFSKSFNVVSVGRIQAAIDAKKLDAKASVTIETLKAAGVIRRPKDGVRLLADGDIKTAVTIEVSGASKAAIEKIEKAGGTVKLPAAPAAAE----------------MAPPTF------AQTTEAP-A--TAEPAKPA---MMPPPPA----------AV-TPAKPDSPETTAKQDKP---KSPGD-PALEAVNPTRFGKAPADEAFGAYQRGLYKTAYNLALPRAEAGDGAAQILLAEILARGLGMPVNMTESAKWYGKAAEQGIPEAQFRYAAILFQGRYAPKDPKKAKELTKAAAEAGNAAAQFNYGQILMQERPGPAGVENAYPWFQKAAEKGLPDGEYAVSQVLANGTPTIPRD-MAKARLYLIKAAIKGYDTAQLDLGSWLVAGLGGPHEYKAGFGWMLRAATGGNVAAQARLAKLYRDGIGVEGDSIKAAAWYIVAKRAGLNDPDLDSFMDGLDDAQKQAAIAEANKLQ--MRDPYSVLGVAKTAKPEEIKSAFRKLAKKYHPDQNQDDPKAQAKFSEINQAYEIVGDKERRGQFDRGEIDAEGKQRVPHGF--EGFSGG--G--GDPFAGF--GGGR-QRA--GGFEFR-SGTGGN--AGF-------GAEDILSSLFGETAGMGGF-GGGAR---RAGPRKGADLQATIDISLEQAAGAEKVEAVFPNGKRLAIKLPAQVENGQTIRLKGQGEAIPGGTAGDALVTVRFKPHPKFRVEGRDLHIDLPVPLRDAVLGSRQEVETLNGRVAVKVAPWTSSDRVLRLKGKGLPKKPDGHGDLFAHVRIMLPENGDPALEAFL-RDTSHMRLILLGPPGAGKGTQSQRLVDKLGIPQLSTGDMLREAVKAGTEVGLKAKAVMDAGNLVSDEIVNAIVSERIDQPDAAKGFILDGYPRTLVQADAVEAMLADKGLDLDCVIELEVDDNVLVERISGRYSCAKCGTGYHDTNKKPLVAGVCDKCGSTEFKRRPDDNAETVRTRLEAYYKQTSPLIGYYYAKGKLKKVDGIADMEDVTASIEKILSDL---------MAIREGALQ---SPGGLEALISRAA-----AQG---KELPPVDKWNPDFCGDLDIEIRADGTWFYMGTPIGRQPLVRLFSTVLRKDSDGKTYLVTPVEKVGIRVEDAPFLAVELNVSGEGNSQIMTFRTNVGDVIEASADNPLRFVVDEANGGLKPYVLVRGRLEALLARPVMYELVSCGEDIAVNGENMFAVRSRGKIFPIMPASELEKQSLMSDHIQIERHGAVQIIRMNRPDKKNALTRAMYATMTKAIVEGDADVSIRAHVFFGVPGAFSAGNDLQDFMAFATSGNMGSEVIDFLITLVNAKKPLLAGVDGLAIGIGTTIHFHCDLTFATPQSYFKTPFVDLGLVPEAGSSLLGPALMGHQRAFAFLAMGEGLHAVEAKEAGLVYKLVETDELESTVLNVAAEIAAKPPEAMQISRDLLHMPRQDAVERIKLEAKLFAERLQSEEARGALMAFLTRKKG---MRVLLIEDDSATAQSIELMLKSESFNVYTTDLGEEGVDLGKLYDYDIILLDLNLPDMSGYEVLRTLRLSKVKTPILILSGMAGIEDKVRGLGFGADDYMTKPFHKDELVARIHAIVRRSKGHAQSVITTGDLIVNLDAKTVEVSGQRVHLTGKEYQMLELLSLRKGTTLTKEMFLNHLYGGMDEPELKIIDVFICKLRKKLDSASDGINYIETVWG---------RGYVLREPDATEMREIA---------------MAAIGAQVRRTETGDALARARQRAALLPDLLVEARRILNTVNTGWHGRRKSGVGESFWQFRPYTEGEAVSRIDWRRSARDDRTYIRDQEWEAAHTVWLWADPSPSMLYKSEQAHVSKESRALVLVLALAELLSRSGERIGFPGITDPSSARNGAERLATLLSHTSSLPAKPDLSKVRRFSDVVIVSDFLDPVEETLAVLQKLAHDGARAHLIEVYDPAEEEFPYRRRTEFIDPENGTSLTFGRAQDYAEDYRRLFFARRDTLSAFCKRIGWSYTTNRTDRLASDALVSVHMNMTASIGYQGGTT--MKTETITLAGDVPGNSIELRVLRFEGRNEMAVAAYLQSSLHGSELPGQAALHFLIPMLEKAEKEGRVAGNITVVPQANPIGSAQWLAHHHLGRFEFFSNVNFNRSFPLLETFDTAALPAIDAPKSLAERLKAQLLRLALPHEIVLDLHCDDESESYLYVHKAFLPEMYDLASALGSTAILSWDSTADAAFEEACTHPVLQLPEAQR--KARAVTTVEFRGLNDVDIETGKSDAEGLYRFLVHRGVISDSSVELKVDFNGPVTPLENVEMIRAPQGGMILFHADIGDEVEAGAKLVTVVTIPGDPGGDITLTAPQAGRILTRRSHRYTRRGDDLMKLLGTKRSDNA-RPGSLEA-MSLKSAYSPSDFILPVLAMCAVVAASNILVQYPFGHFGLGEILTYGAFTYPIAFLVNDLANRTYGPAFARKVVYVGFTIAVILSVWLATPRIAIASGTAFLTAQLLDITVFNKLRQLTWWKAPFASAIFGSLLDTLLFFSIAFAAQFAWIDAATGQADSSLAMLVPFFGSEIPLWMSLGFGDLMVKLVMAIVMLVPYGAILAIFAPAIYSASRNSR--------------------MRNDLFNMDTKDGLSDPDPVRRAQIQSKLPLPKRFYKQAAVAEHDGA-FAVELDGRVVKTPARQNLSLPTRAAAQLIADEFSAQDKEIDPARMPATRLANTAIDGIVNDPQAVLEDVLRFASSDMLCYRAGSPERLVNRQTELWDPVINWAASHLGARFALAEGVMHVEQPREALGAFSAHLGAFTDPFAIASLHTITTLTGSAILALAVAKGEVSGAEAWELAHVDEDWTIEHWGEDAEAAARRAMREREMIAAVKMLEAVTGG-MSNPAITIVIPCRNEAANLAFLLDEVDAAMDGRSYEVIVVDDGSTDATGAALEARIKAGKPLRHVRHDKSAGQSAAVRSGVFAAKGKVVVTMDGDGQNDPAYLPRLADALLQAGPAIGIAAGQRLKRTDTKLKQISSKYANMLRGAILQDYTRDSGCGLKAVHTHLFRELPFFDGWHRYLPALVIREGYGVVHVDVVDRERRHGKSNYGILDRGLRGILDLYGVWWLRKRRKTVPTITEI-----------------------------------SHGMFRLAHISDIHLSPLPALTLRELVSKRITGYINWRSNRKGSMTGGTLDTLIADMLAQEPDHIAVTGDLVNLALDEELETAQRWLKTLGDPANVSVVPGNHDAYVPGALKKTRRFWEPWMRGDREAAANIPVEFPYLRVRGDVALVGVSSARATAPFMATGDIRDRQARKLGEILDETGKLGLFRVIMIHHPPVRGAAPSHKRLLGIGLFQRTVREHGAELVLHGHTHLATRYEINGPDWKIPVICVPSASQSF--GGN--HKPPAAFNLFSIARTNAG-WSCQMVERGIVDDQMTVRTIREHDLTVGAIFNPAL---MAVSANRLELLQIADAVAREKSIDREIVIAAMADAIQKAARSRYGQETNIRADINAKTGEIKLQRLLEVVEDVEDPVTQISMEAARDRNPDAQPGDFLADQLPPMDFGRIAAQSAKQVIVQKVREAERDRQYDEYKDRVGEIINGSVKRVEYGNVIVDLGRGEAIVRRDELIPRETFRYGDRVRAYVYDVRREQRGPQIFLSRTHPQFMAKLFTMEVPEIYDGIIEIKSVARDPGSRAKIAVISRDSSIDPVGACVGMRGSRVQAVVGELQGEKIDIIPWSPEPASFIVNALQPAEVAKVVLDEDAERIEVVVPDDQLSLAIGRRGQNVRLASQLTGWDIDILTEQEESERRQKEFVERSNLFMEALDVDEMVGQVLASEGFASVEEIAYVDSDDISSIDGFDEETATEIQQRAQEYLGRIEEERDARRKELGVSDELRDLPGMTNAILVAVGEDGVKTVEDFAGYAVDELTGWRERKDGETINYPGILSQFDVSRADAEQMVLAARLKVGWITEDDLAAEE-PEAEVDQEVAG-----MPDLLLELRSEEIPARMQRKAAGDLKKLVTDGLVEAGLTYEAAGDYWTPRRLTLDIRGLNARSKDVHEDRKGPSVNAPEQAIAGFLRSAGLSDISEAHVHTDPKKGDFYVAHLTKPGRAAEDIIAELLPSIIREFPWPKSMRWGAASSKPGSLRWVRPLQSILCTFGPETEEPVVVDFEIDGLRAGNVTYGHRFLSDGKPITVRRFDDYVAKLEKAKVVLDADRRKEMILSDAHNIAFASGLELVEDEGLLEEVSGLVEWPVVLMGEFEEEFLAIPPEVIRLTIKTNQKCFVTRRH---------------------------------------GEADRLSNNFILVSNIEATDGGREIAYGNGKVVRARLSDALYFWHTDQHDLPDMHTLAAAGIKFDLDLKKPLDQRMARLDALNVTFHAKLGTQGERVTRIRALARDIAPLIGAD-------PT---------LAMRAAVLAKADLTTEIVGEFPELQGGMGRKYALLQKEHLSVAAAIEEHYKPQGPADFVPSDPVSVAVALADKLDTLVGFWAIDEKPTGSKDPFALRRAALGVIRLLLSSDRKFELLPVFEAAFAQLREDSIEQRLEQFEERQAAQQTGDVDGEEDVINAISGFEKRVRDQVSARARPV---------LLDLLGFFHERLKVHLRDLGARHDLIDAVLTEGADNLLLIARRVEALVVFLNTEDGKNLLAGTKRAANILSAEEKKGTVVTETVDPALFSLDEERHLHAAVNQAESQAAQAIQKEDFSGAMVALSALREPVDSFFEKVLVNDENPDIRANRLALLSRIRTATNTVADFSKIVG------------------------MVFAVGL-----SGC--------------STSGTKTEDPNSPKAQATAE--AQKRISESELRAFCPTVTIREGTAVLTNYGKSSDKTPDNLIYQASIANETRSCQPGDTTMTMNVAIAGKIVPGAKFSPGTVTVPIRVAVV----QGTNVLYSKLHKQAVSATDSSAATQFVFNDPNVTFPKPTSQNIQIFI---GFDEG---PESSGTKA-KQKTQ-MPSLKDLRNRIASVKATQKITKAMQMVAAAKLRRAQEAAEAARPYSQRMAAVLANIASNVDGDDAPVLMSGTGKDDVHLLVVCTSERGLCGGFNAQIARLAREHTRRLIADGKTVKIITVGKKGADILRRDLGKQIIDHVDLRDVKQLGFVNADQIGHKLIALFNEGAFDVATLFYSEFKSVIAQIPTAQQLIPANSISDAE----PESAGDAVYEYEPDPASILGDLIPRNISVQVFRALLENAASEQGARMSAMDNATRNAGDMINKLTMSYNRQRQAQITKELIEIISGAEAL-------MRSVFVFSIGSLLAISPALAMA-------QAQE------------------PVQQQAALPN-----QTLAEKRAARLNELFDSLKRESNEVKAARIASLIQGQWQNSGSATVDLMMSWAAKAMEDKKYSVALDFLDQVVVMKPDYAEGWNRRATLHFMMNDYGRSMADIQRTLSLEPRHFGALSGMAAILKDTGRKEAALEAFERVLAVYPMLRAAQTQAGELADELTGQRT----------------------------MSFFAIATIISALYGDARLKSFQQLLSAHLFSVIASVSVN-DGALAGRPDPGEVRYSSPLSGWYWSVEPVADNLKGSLRSVSLGDKMIDAPSTLNVPFDTSFQRTYTVPGPNGQQLSVVETEVVLDAENRVARFRVMGNLSEVEREIAGF-RNTLYFYLGIFALGGTLINAAVILFGLRPLDRVRRSLAEIREGK------VSRLNENLPLEIAPLAREMNALIENNRRIMERSRTQVGNLAHSLKTPLSVLVNESRTIGGTEGRIIAEQSAAMQVQVQHYLQR-------ARVAAQRDSVVFRAPVTPILARLARVTAKLNPNMDVQFVNHMDEAI-FAGEREDLEEIVGNLLENAGKWGRSIIRLTLA------ESD----DG---LEISVADDGAGLAPEQIKDALMRGRRLDETKPGTGLGLSIVHDTVREYGGTLRLERSADLGGLNARLTLPVAGNMSRTCELTAKSVQYGNNVSHANNRTRRRFLPNLCHVTLISEALGQSFRLRVSAHALRSVEHRGGLDAFLVKADEKELSQRARLLKRQIAKKLLEAPVAA--MEIFRDANKETGFLNGQFLLAMPGMEDSRFARSVIYVCAHSEKGAMGLIINRVQDMEFADILVQLGVLDEQQAIMMPETTRDFLVRNGGPVEVRRGFVLH--------------------SDDYLTE------STMPVAEEICLTGTVDILRAISGGRGPRKALMTLGYSGWASGQLENEIANNGWLTCQAPHEMLFDTDIDSKYDRILAYMGVDPSRLATVAGHA-MEFYWPATTGEWLAWLSAAITVIFGLVLFFAPRVALKILRLQTKPERPEALSEIRGTFAGFYLGLGLSALIFAQPFLWIALGLSWGFTVFGRLISMMSDRGNTLYNWVSVIVEIALAAGPLLFAFGFIA---------MADKRADVAIIMGSQSDWATMRHAAETLDSLGIAHDDRIVSAHRTPDRLVDFAKGARKAGFKVIIAGAGGAAHLPGMAASMTPLPVFGVPVESKALSGQDSLLSIVQMPAGIPVGTLAIGRAGAVNAALLAAAVLALHDDALAKRLDDFRQAQSDKVAMRPSDEASMNQNRFPLYAGLIAFIALLAYSSIFVVRAGQQAIVLRFGQIVDVKADPGIYFKLPFGFLEADNVQMIEDRLLSFELDNIRVQVSGGKFYEVDAFLVYQITDPRKFRQTVSGDVTLAEARLRTRLDASLRGVYGLRGFEAALSDERAGMMNEVREQLRPDAESLGLQITDVRIRRTDLTQEVSQQTFDRMKAERLAEAERLRARGREAAQRIKAIADRQVVEIIAEARKESEIARGQGEGERSRIFAEVFSKDPDFFAFYRSMTAYGTALDNTGTTMVLSPNSEFFRYFQDSN----GALPPVSGTTPP----VTPAPPAPAPQMPLIEKNASLDTSIGLSEANASDYIALLKPRVMSLVVFTGFVGLMAAPGQINPVLAAIAILCIAVGAGASGALNMWYDADIDAIMKRTAKRPIPAGRISREEVLTFGLLLSVFSVVTLGLFVNLLSAFLLAFTIFFYAVVYTMWLKRSTPQNIVIGGAAGAFPPMIGWVAVTNSISMESIVLFMIIFLWTPPHFWALSLFMSDDYEKAKIPMMPNVMGEASTKFQIFLYTLLVAPVAVLPWVMGFAGPVYGVFSIAMGAAFLGYAWSVWRSPVGEAMMRPAKKMFAFSIFYLFSLYAVLLGEILVRKALLVAGA-----------MDSQGFAVGINDVEAAASRLQGHLMPTRFIESEALNARYGARILFKPECLQRTGSFKFRGAYNRISQFTAEERARGIVAFSSGNHAQGVASSARLFGINAVIIMPADAPQTKIANTRSYGAEVVFFDRYKESREAVAIPFIEERGMVLVPPYDDPSIMAGQGTIGLELVAEARERGLTLDEVFVPSGGGGLISGISVAVKAASPQTRIWGVEPENFDDLRRSLIAGERVSNEAGHRSICDAILTPQPGELTFPINKHNLAGGVAVSDGAVRSAMRDAANYLKLIVEPGGCVALAALATGEVDIKGKTVAVVLSGGNVDLDVYGGLIAAA-MVNRIVVVLILLPLAVILIALSVANRQAVSLTIDPFNPGNPLLSYSAPLFVWLFLALIVGLVLGSLATWYNQGKHRKLARQRKLEAELLRKEARKASAENTSTP----NLPSLHMSQETDSAHPRNPALGIVRLPHGEGVELPSYETSGAAGMDLRAAVPEDRPLLLLPGRRALVPTGLIFEIPKGFEAQIRPRSGLALKNGITCLNTPGTIDSDYRGEVKVILINLGDDDFYVTRGMRIAQVVLAPVVQLAIEERQHASDTARGAGGFGSTGTS--MNAILLVASGGAIGSVARYLVGILMARILGVAFPWGTLTVNIVGGLLMGLFIEL--LARRFEGSPELRLFVAIGVLGGFTTFSSFSLDFAVLWERGDLAAAFFYVLASVILSIGALFFGL----WLARIAA------------------------MIRWLLPSVAVFGIVGLASLVTFTSHQGFRRPVPETPKPQTAEAPPQPTKPAP-----------VQSSSRSDAGKVI-SPE--GGAKVIVVGDPTKRMQDPRTAHIRERDMLEESPQGPLPVISPDGRRPLDVYARPWSGARGARVAIVIGGLGLSQTGSQQAIRALPGEITLAFSPEGNSLGRWMQAARQDGHEILMQIPLEPYDYPRVNPGRNTLTVDATPQAILENLHKTMGRITNYTGVMNYMGARFTAEPAAMTPVIQDVAKRGLLYLDDGTSARSQADAIAAQQGAAFAAADVLIDASQERGSILKKLDELERIARAKGTAIATGSAFDVTVEAVTSWANEAKARGIEIVPISALVRDPERGMTVLQRVKSITHRICEQSKPTRDVYLDHLREAASRKPKRSALACANLAHGFAACSPSDKAALAGDVVPNLGIITSYNDMLSAHQPFETYPQLIKAAAKEAGGVAQVAGGVPAMCDGVTQGQPGMELSLFSRDVIAMATAIGLSHDMFDAAVYLGVCDKIVPGLVIGALTFGHLPAVFIPAGPMTTGLPNDEKAKTRQLYAEGKVGREALLESESKSYHGPGTCTFYGTANSNQMLMEIMGLHMPGSSFINPGTPLRDALTREAAKRALAITALGNEYTPVGEMIDERSIVNGVVGLHATGGSTNHTMHLVAMAAAAGIKLTWQDISDLSDVVPLLARVYPNGLADVNHFHAAGGMGYIIRELLDGGLLHEDVKTVWGGGDGLRAYTIEPKLGENGTVVREPVAAESADKKVLSTCKQPFQVTGGLKMLKGNLGTAVIKTSAVKADRHIIEAPAIVFDSQAALQDAFKAGKLDRDFVAVVRFQGPRANGMPELHKLTPALGVLQDRGHMVALVTDGRMSGASGKVPAAIHVTPEALDGGIIGKIHDGDVVRLDAEIGTLDVLEDPDVLAARPTPEVDINHNSYGMGRELFAAFRNVVGKAENGASVFG-MAFYDTLLNAFVTLMVTIDPPGLAPLFLALTRGMNRKERSQVGLRASLIAFMVLTLFAVAGAQILALFGITLGAFRIAGGLLLFWIAFEMIFERRNERKEKSAEVAITKDHIRNIAAFPLAIPLIAGPGAISATILLAGTLSDATSRLALIAIILVCIAASYVVFLLAERVDRFLGETGRSILTRLLGVILAALAVQFVADGIKTLVLSMVKLNKIYTRTGDDGTTGLGSGERRLKHDLRVEAYGTVDEANSCIGLARLYTEKDFPELDAMLMRIQNDLFDLGADLSTPDTGETLAYEPLRIIDTQVLRVEADIDQLNANLAPLRSFVLPGGSPASAALHLARTVARRAERHMVELTQKPDETVTPAALKYINRVSDFLFVAARVVNDNGAKDVLWVPGHNRMKIKNSLKALKGRHRDNQLVRRKGRIYIINKTAPRFKARQG---MAEAFQFELVSPERLLLSEQVIEVVVPGTEGYMTVMAHHAPLMATVKPGVVTVKMADGKPDSYVVFGGFVDITPDGCTLLAESAVHVTDIRADELQRRIQDAKEDFEDATTREDRAKAEDLLGQLTTLEEAIKAA-------------MSITAERKQAVISEYATKPGDTGSPEVQVAVLTERIVNLTEHFKDHKKDNHSRRGLLKLVSTRRSLLDYLKKVDQGRYQTLIEKLGLRRMDNFEKYALALMVVFGALIIGGLMAVHIAWEHKAGFLYALGAAVVVWAAGFAVLFDKPRLYGLLLLIATSLITASVVVLVR----MTNGALKTLFLPFDQEILAVPSASERWLFLGAEADREISDGWRGVLTCLQPFKPDYLALQKAGFNAIPRLQGNERFDGALILIGKHRGRNEAWLALALSHVNPGGRIVVSGDKKLGVDSFRKTVEGLAPVIDRLSKHHAVAFWFDRSEQLNSDKIQALMAAPTRLENRFTTGPGMFSHTAIDKGSAMLVKHFEGRISGHVADFGAGWGYLAAQVLNYPDKLKSLALYEADFEALEAARLNVAG-SPDVPVSFHWFDVNSEAISEIYDTIVMNPPFHAGRSADPTMGQGFIAAAAKRLKPGGKLLLVANRQMPYEADLKALFKSVQPLEDDAGYKIIEAKKMNNAALIRPGWTPATIALMIIGFMLFWPLGLAMLAYIVWGNRFGGFKSEMSNATDNFCGAFRRN--RRSHHFGRGPFGTGNIAFDEWREKELERLDEERRKLDEMRGEFEEYARELRRAKDREEFDRFMAERNA--KKPS--GSVPGVNDTDGEMSDTKSTDDKTLGVN-KKTLTLKRPGVEQSTVRQNFSHGRTKSVVVETKKRKFSLPDHKPEVAPP--AAPAPA-PAQVA-APAPT----APV---------RPAPQPA-ATQV-----SPAAAPRPAATSATAPA--A---QPSARPAAPAQQQRPA-QQPYRPAGQRPNDRSGMVLNTLSTAELEARRKALEGSKARDAEDRAKAAVEAVRRAEEEALRAKEREESARRQAEEEERIRKEAEAKLRAEEESRKRQP-ELKTAD---------------DAAALK----APVR-K-PNLAEEDEDRR--G-PVGARRG--SSAAPAKPEVRAPKVVKGEDERRRGKLTLGSALNDE-GRSRSLSAMRRRQEKFKRGMQQESREKIAREVILPETITIQELAQRMAERSVDVIKFLMKEGQMMKPGDLIDADTAQLIAEEFGHTVRRVAESDVEEGIFNVQDDETAFESRPPVVTIMGHVDHGKTSLLDAIRQANVVSGEAGGITQHIGAYQVEQNGQKITFIDTPGHAAFTAMRARGAQATDIAILVVAADDSVMPQTIESINHAKAAGVPIIVAINKIDKPSADAQKVRNGLLQHEVFVESMGGETLDVEVSAKTGVGLDKLLEAILLQAEILDLKADPTRTAEGVVIEAKLERGRGSVATVLVQKGTLHPGDIIVAGSEWGRVRALVNDRGENMKEAGPATPVEVLGLQGPPQAGDRFAVVENEAKAREISEYRTRLAREKAVAKQAGSRGSLEQMMSQLQVTGLKEFPLLIKGDVQGSIEAIAGALDKLGTDEVRARIIHSGAGGITESDIALAEASNAAIIGFNVRANKQARDLAEREGIEIRYYNIIYDLVDDVKAAMSGLLSPERRETFLGNAEILEVFNITKVGKVAGCRVTEGKVERGAGVRLIRDNVVIHEGKLKTLKRFKDEVSEVPAGQECGMAFENYDDIRAGDTIEAFRVEHVTRTLMNANETHCMQFLRDADPDRYLSVLYAPEDKRGSLAALYAFNAEIARIRDMIHDPLPGEVRLQWWRDLIHGTEHGAASGNPVAAALLHTIKAHDLPRSAFDNYCEARIFDLYDDPMPSRNDLEGYCGETASALIQLASLI-LDGNAAPAHADTAGHAGVAQAIAGLLRLLPLHRRRGQVYIPADILAAVGTSAPTLLNGLDPAATKRAIDAMIALAEDHFAKFEAAAKTLPVTLRPAYLPARLTRTYLEKLKADGADAANEMANISPIRRQWAMFRASVR-------------------M-THSPAHANT--------RKVYVKTYGCQMNVYDSQRMADSLAAEGYSTTDTADDADLVLINTCHIREKASEKLYSALGRLRKMKDVRAKEGRELTVGVAGCVAQAEGDEITRRAPVVDLVVGPQTYHRLPQALARVRQGERVVETEYAIEDKFEHLPAPGKEQTRSRGVTAFLTVQEGCDKFCTFCVVPYTRGSEVSRPVAQIVREAEKLADAGVRELTLLGQNVNAWHGEAAEGREWGLGELLYRLAEIPGIARLRYVTSHPRDMDDTLINAHRDLDMLMPYLHLPVQSGSDRILKAMNRKHTAADYLRLIERIRGARSDIALSGDFIVGFPGETDEDFEATMQVVRDVTYAAAYSFKYSPRPGTPGADMDGHVAEAVKDDRLQRLQALLSEQQYAFQRSLIGKSMDVLIEKAGRVSGQMVGRSPWLQPVIIDVHGGRIGDIINVRIIDTGTNSLI-----------GVRE-MNSASNGTI---------MTARWYIVHAYSNFEKKVAESIEEKARQKGLSHLFEKILVPTEKVVEVRRGRKVDAERKFFPGYVLVRADLTDDAYHLIKNTPKVTGFLGSDNKPIAISDKEADRILTQVQEGVERPKASISFEIGESVRVADGPFASFNGTVQEVDEERSRLKVEVSIFGRATPVDLEFGQVEKV---MSSEALHSEQPRPLVRNRTDGHVIECNGEKAIIHAKTGPSVLASDDYWVVGQLISIQVATSRVVGLVYKVDLPIAEWGQGIDQSIHIHVELVGQVKTLADGTLRFSSGIATYPHMGAIAHRIRAADLATIYTTTLKNTVSVGHLSQNADIPALISIDSLISRHFAVLGSTGVGKSSAVSLLLRKIIKQRPDLRVLILDPHNEFASAFPEHAISIDYTTFNLPYWLFRLEEFAEAVFRGRPVIPAEVDILRDLIPVAKERFASADAATSASLLKKDRDYSSMTADTPAPYRMADLIKLIEERIGQLDGKPDRPTMKALKQRLQALVADPRYRFMFSAPTTLDTMRQTIGHIFRIPKNDRPICVFQLAGLPSEVVNSVASVLCRIAFDLAVSSDGRVQTLVVCEEAHRYIPADANAAFWPTRQAIARIAKEGRKYGVYLSVVTQRPGELDATILSQCSTVFAMRLGNERDQEIIRGAITGASQSTIGFLSSIANREAIAFGEAVPTPMRMMFETIPADQLPAAHMAKTQESVRLGDSHLSLDTILMRMRQLNAAETSGSDD-TADTL-----EGIVS----APVASQRLS--TLSQEPARSQWRSTDTT--VRNPEP-LKPQPENTERSRASDLIRTFRNDKTMGSFSIWHWLIVLAVVLLLFGRGKIPELMGDVAKGIKNFKSGMSDDDAA---ADNSKTIDNQTSQPVNSVK------EKTTKS-MNKVDRDIANLPDTTVSVAKLFGFESDMMVPAYKAGDAYVPEIDTDYLFDKQTTLAILAGFAYNRRVMVSGYHGTGKSTHIEQVAARLNWPCVRVNLDSHVSRIDLVGKDAIVVKEGMQITEFRDGILPWAYQHNVALVFDEYDAGRPDVMFVIQRVLESSGRLTLLDQSRVIRPHPAFRLFATANTVGLGDTTGLYHGTQQINQAQMDRWSIVTTLNYLPHDNEAAIVAAKAKHYQNKEGKEIISKMVRVADMTRSAFINGDLSTVMSPRTVITWAENADIFKDVGFAFRLTFLNKCDELERPIVAEFYQRAFGKELPESTANVVISMSQETNSDLYPEPIIANTKSNKAGLRLVILGVLLVGVSVLYFIYRDQLGQQFLLGLLGTLAMTGVFYLFGSAINVIQFTPRGTSDELTRAFVDTLPEGTVVSDQKGRIVYANRAYAELTGVTNSNDVRAIEHILSGEAAASDPIYRLANAVRDGVSGQEELRLSRPL----DPSRE----PVPTWYRVKARPVAGIPDQTAPVYAWQIADISAERAEQERYFQDIQEAIDHLDHAPAGFFSADADGRIIYINATLAEWLGVDLTRFTPGSMMVRDIVAGNGMALINAVKTEPGTSRNTVIDLDLAKANGQSLAVRFYHRVQAQRDGRRGPTRTIVLNRAEGEDTSAALRAAEVRFTRFFNSASMAIAAVDATGKILRTNARFLGLFSPVVDRDDIDRRIPLETVVHERDREAFDRALAAAFAGQAEISPVDTVIPGNEERHLRFYISPVFDAGSEDGAEEAAIVSVVETTEQKALEATMAQSQKMQAVGQLAGGIAHDFNNVLTAIIMSSDLLLTNHRASDPSFADIMNIKQNANRAASLVRQLLAFSRRQTLRPEILNLTDVIADVRMLLTRLAGTQVNLKIEHGRDLWPVRADLGQFEQVVVNLTVNARDAMPEGGDLIIRTRNMNVDENAALTYRDLVPGDYVLVEVADTGTGMTPEVIAKIFEPFFTTKEVGKGTGLGLSMVYGIIKQTGGSIHAESELGKGTTFRIFLPRFVEEKQPQVLNADGEAVVTEIPKKEKKTEKATDLSGSATVLLVEDEDAVRMGGLRALQSRGYTVHEASSGVEALEIMRELDGKVDIIVSDVVMPEMDGPTLLRELRKDYGDIKFIFVSGYAEDAFARNLPEDAKFGFLPKPFSLKQLATAVKEMLES--MSVKSEPTKGGKNGLTYAQAGVDIDAGNLLVEKIKPIVRSTRRPGADGEIGGFGGLFDLKAAGFKDPILVAANDGVGTKLKIAIDAGVHDTVGIDLVAMCVNDLVVQGAEPLFFLDYFATGKLDPDQGAAIVSGIAAGCLQAGCALTGGETAEMPGMYRDGDYDLAGFAVGAAERDQLLPTADIAEGDIIIGLASSGVHSNGFSLVRRIVELSGLGWDADAPFKSGTTLGEALLTPTRIYVKPLLAAIR--ETKAIKALAHITGGGFPDNIPRVLPKNLTASIDLSAIKVPPVFSWLAKTGGVERDEMLRTFNCGIGMIAVVAPENVDAVLEALKAQGEQAVRLGTMIARADAGVVYQGTLTL-----MLTEADHSLLSLLRENARASTADLARKLGVSRTTVQSRIERLEKRGMIRGYSVQLSPEYERNLVRAHVLVTALPKLATKVEAALRKIAWVRTLHSVSGQFDMIVIVEAPSIQELDKLLDEIGALEGVERTMSSIILSTRIDRMNKIDDDIDKSSAPLIEHLIELRRRLIWALVAFFIAFLGCFHYAKELFNLLVIPYQWAVGWAGFDPAKAQLIYTAPQEFFFTQVKIAMFGGIVIAFPVIASQIYKFVAPGLYRNERMAFLPFLIASPILFLMGGALVYFFFTPMVMWFFLAMQQQGVAGEVQISLLPKVSEYLSLIMTLIFAFGLVFQLPVVTTLLARVGLVGYEGLKTKRKYAIVIAFIVAAVITPPDPVSQIGLALPTILLYEISIWMARLVEKKRAAAETQAQSGS--SETSSS---------------------------------------------MTDTAM---PAQRGSAESTVFAIIFAVSFCHFLNDMMQSLLAAIYPMLKQNYGLDFKQIGFLTLTFQVTASLLQPVVGTYTDKRPMPYSLPVGMAFSLVGLGLLSIATHYVMLLAGAAFIGIGSSIFHPESSRVARLASGGRHGLAQSFFQVGGNFGTASGPLLAAFIVLPRGQQSIAWFSVAALLGMIILYQVGSWYQRYRAANANRPPASKVLRLPRKKVVASLIILTLLVFTKNIYLASISSYYTFYVIHKFGITVQQSQMMLFLFLGAAAVGTVLGGPIGDKIGTKAVIWFSILGVLPFTLMMPYANLFWTGVLTVFIGFILSSAFPAIVVFAQELVPGRVGMIAGIFFGFAFGMAGIAAAVLGFVADIKGIDYVYAVCSYLPFLGLLTIFLPSMREVRGESVAI--MSDQHKFVLASGSPRRLALLSQIGIEPDRLFPADVDETPLRAEHPRSLARRLSRLKAEKIAEELKGDADLGESYIVAADTVVAVGRRILPKAEILDDATACLRLLSGRSHRVYTGLCLITPKGKLRQDLIETRVRFKRLSREELESYLASGEWRGKAGGYAIQGLAGGFVVKLVGSYSNVVGLPLYETASLLHGEGYPVYANWQASKHMPTEPFWKTKTLDELNRSEWESLCDGCGRCCLHKLEDEDTEEIYFTTVACTLLDAGTCQCSDYTHRKRKVPDCVFLTPEIVRTVDWLPETCGYRLIDEGRDLYWWHPLVSGSVETVHEAGISVRGK-ITAYDHDLSSDEDY-FEHMMQ------AELPSLNRE--------------MFSDRSNRFLALWFPHLPTDRLKRENPERGRTDQHLIVVDKLANALRLTAVDERAAKAGLHFGMALTDARAQFEHLDIAFASSRKDKLLLDRLADWCDRYTPLVAFDEPHGLLLDMTGCIHLFGDEPSLVKDIHKRLHQHGMIVRSALASNAAAARALARYSQGGIVGGQD--------------QQRRLYGLPLVSLDIEENHLAGLKRAGLRSVGDVDVLPRGALTARFGTRLVEHLDAMFGRQSEPISPRRIIPVCMVERRMAEPLIAMESVELVLQSLACELFLRLQQRAEGAREVEASFFRADGNVRRISVQTGRPVTETKTLFRLLKERLSMLSDPLDAGYGFDLIRLAAVRVERSQAIQKSLDNRAQETEEINTLIDRLSIRLGNARVLRPVAVDTHVPERAVSLEPAAKTKHDNALE-----WAPE------HYPVNPPERPIRLFEPPERIDATFEVPDAAPAQFVWRRVRHTIVMAEGPERIAPEWWREH------------SG-------------ALTRDYYRLEDTTGKRFWVFREGLHERGNPNPRWYLHGLFAMKKLMMALTI-----ATSFAVAGAANAQEKLKVGFIYIGPPGDFGWTYQHDQGRKELEKALGDKVETTFLENVPEGADAERSIERLARAGNKLIFTTSFGYMDATIKVAQKFPDVKFEHATGYKTAENVAVYNSRFYEGRYVQGVIAAKMSTTGVAGYIGSFPIPEVVQGINSFMLGAQSVNPNFKIKIIWANSWFDPAKEADAAKALVDQGVDIITQHTDSTAPMQVAAERKIKAFGQASDMIKFGPETQLTSIVDNWGPYYIERAKAVLDGTWKTHNVFHGMHEGLVVMAPYTNMPDDVKKLAEETQAKITSGELKPFTGPIKKQDGSEWLKAGETADDKTILGMNFYVAGVDDKLPQMRRRRKVKILATLGPASSDEAMIRKLFEAGADVFRINMSHASHDLMRELVRRIRTVEKSLGRPIGILADLQGPKLRVGKFEGGKVDLVPGQTFTLDNNDTPGDKDRVFLPHPEILEAVEPGHRLLIDDGKLALVAEASDGKSIRCRVVSGTKISDKKGVSLPDTTLGVGALTEKDRRDLDAVLQEEIDWVALSFIQRPEDLAEVRKISRGRVALLSKIEKPQAVARLDEIIELSDALMVARGDLGVEMPLESVPGIQKQIIRACRRAGKPVVVATQMLESMITAPVPTRAEVSDVATAVFEGADAVMLSAESAAGEYPVEAVSTMVRVAEEVERDPNYPGIIHAQRPEPNATGADAISLAARQIAETLNLSAIVTYTASGTTGLRAARERPLLPIIALSPVVETARKLSLVWGLHCVVTEDASDLDDMVDRACHIAYQEEFGNPGDRIIISAGVPLRTPGSTNMLRIAYIGSDGLTGI--------------------------MVHAADLMRKAVMRLAW----------HS---------------------------GFASRTRDRFG-------GLGSIIMLHRVRPDCISP-LGVTRSLSITPRFLDQILVSLKSEGTELVSMDEMIERMKSGSQDGRRFAAVTLDDGYNDNLNHAYPVFQKHNVPFTVYGAPGLIEGAAVLWWEVVEKLVAKASVIRIP-G-GPEW-RCGTLFQKRSAFHTLMRYFSYTLTELEQLPVLREMCAAEGMICGVS-ADQDVMTWKELRQIASDPLCTIGAHTVHHYNLKKLTAEMVAQEMRLSADMLEDNLGKRPAHFAYPYGSAKAAGRREAEIAAEEGFLSAVTTRHGTIHPEHRANLHALPRISINGRFQKLVYAQTMMSGATTPIANAGRRVVQA---------MTVELGHFALVLALALSIVQAVLPVLGARRGDDRMMAVAVPAAFTVFALIALAFAALTAAYVLSDFSVQNVWENSHSQKPLLYKFTGVWGNHEGSMLLWVLILSFFTALVAFFGNNLPPALKANVLGVQAWIGTAFLLFIVATSNPFARMNPAPIEGQDLNPILQDIGLAVHPPMLYLGYVGFSVCFSFAVAALIDGRIDAAWARWVRPWTLTAWIFLTGGIAMGSYWAYYELGWGGWWFWDPVENASFMPWLAGTALLHSALVMEKRSALKIWTILLAILTFSLSLLGTFLVRSGVLTSVHTFATDPGRGLFILAILVLFIGGSLALFAWRAQTLAPGGLFQPISREGALVFNNLFLTTAAATVLIGTLYPLLLESLTGEKISVGAPFFNLTFGPLMIPLLAAVPFGPLLAWKRGDILAVAQRLMTAFFIALLVVAIVLFRTSASGVLAAFGIGIAVWLMMGALTDLALKAGIGKASLGTMLQRVAGLPRSVFGTALAHFGLGVTLLGIVSVSTFATESVLVMKSGDGIRAGGYTLRFDSIDPFKTSNYTEDQGKFTVLNDEGEPVTELVSSKRFFPVRKMQTTEAGIKTFLFSQLYVSLGDAAKDGGVVVRIWWKPFVTLIWLGALVMMAGGSMSLLDRRLRVGAPAKSKVL--KPRAAT----MHDVSHGPV--AAAAFSDEERAAVYKAIYSRRDVRDQFLPRPVPDEVLMRLLDAAHHAPSVGFMQPWNFILIRDEARKAQVHRAFMRANDEAKGLFTDERQALYASLKLEGIRNAPVNLCITCDRTRGGKVVLGRTHNRQMDVYSTVCAVQNLWLTARAEGIGVGWVSIYHDQDMRHILGIPDHVEIIAYLCIGYADELYDAPELEIRGWRKRLPLEGLIFEEEWQRSA----ISSDPPRPDPATGLPATRSTCDMPPYRSRTTTHGRNMAGARGLWRATGMKDEDFGKPIIAVVNSFTQFVPGHVHLKDLGQLVAREIESAGGVAKEFNTIAVDDGIAMGHDGMLYSLPSREIIADSVEYMVNAHCADAMVCISNCDKITPGMLMAALRLNIPVVFVSGGPMEAGKVVWEDKEKKLDLVDAMVAAADDRISDEEVKVIERSACPTCGSCSGMFTANSMNCLTEALGLSLPGNGSTLATHADRRRLFVEAGHLVVDLARRYYEQDDDTVLPRSIASFAAFENAMTLDIAMGGSTNTVLHLLAAAHEGEVDFTMSDIDRLSRRVPVLCKVAPAVANVHMEDVHHAGGIMGILGELDRAGLIDTSVYSVHSANLGAALDRWDVVRTKNKSVHDFFMAAPGGVPTQVAFSQERRFADVDLDREKGVIRNAEHAYSKDGGLAVLYGNLAEDGCIVKTAGVDESILKFSGPARIFESQDSAVLGILNGAIQPGDIVLIRYEGPRGGPGMQEMLYPTSYLKSKGLGKACALITDGRFSGGSSGLSIGHVSPEAAEGGLIGLVEEGDIIDIDIPNRKIHLAVDDAVLADRRAQMEANGSAAWKPAEKRKRKVTTALRAYGAMATSAAKGAVRQIPDEWSMDKSPDAFRTISEVAEDLDLPQHVLRFWETRFTHIKPMKRGGGRRYYRPLDVDLLKGIRHLLYDQGYTIKGVQRLLRENGTQFIIALGSGDTAAAEAISQQKQATARQEEALAAEQSRAEADEEAAVMGTPKSVPQGRRLFGLIKGDDEGPVAADGKRLSKDNRALLQETLFDLLECKRLLDQVRMDKTTAGWINGVLGVLIFSGSLPATRAAVADFDPIFVTSARATIAALLGLALLLAFREKRPAKSDLGALFVTAMGVVVGFPLLTALALRHINAAHSIVFIGLLPLATAIFGVIRAGERMRPLFWLFSVLGSLIVAGFALTQNLSASITGDLLMFAAIVVCGLGYAEGAQLSRKLGGWQVICWALVLSLPVMLPLTVYVMPGNWSAVGEPAWIGLVYVSMFSMLIGFVFWYRGLAQGGIAAVGQLQLLQPFFGLVLAATLLHEPVGWPMVVVTVAVILCVAG-------------------------------------AKRFARMSLRVSGKHMDIGDAFRVRIEERIGEMVTKYFDGGYTGHVTVEKQGSRFIADCLIRLDTGTVLQAAGEAQDPLLAFDAAGERVDKRLRRYKRRLKSHQAPGSNGVFDDVSYRVMAPVP----DDDEDFPEDYAPTIVAESSMALRSMSVASAVVELDLKDSPVVVFRNSGSTQVNIVYRRPDGNIGWIDPSAVAGQNAAQQ--------------MTSTRTDPIVIAGAGHVGLIAAIAIGQTFDNVISLGTIPEGNDKRTTALMVPAITYLQKIGLWEAIEPHAAPITTMRIIDGTNRLIRSPTVTFEAGEIDEAAFGYNIPNVTLTSVLSDALKHST-VRHVAASASHYHPTEGAIAVEASDGSMYTANIVIAADGRSSLAREAAGISARTWSYNQTAIVLSFSHTREHHDISTEFHTEHGPFTQVPLAGKRSSLVWVTTPAHAVELLELDRNQLATRVEERMQSMLGAVTIDVDPQSWPLSGLVPTSFARNRVFLAGESAHVFPPIGAQGLNLGVRDVETLLETLPNDGTDLGASSVISTYNRKRSPDIIARTGAVDALNRSLLSDFLPVQMVRSGGLELLRAFSPLRGFVMREGLRPGSGFR--FPRKGAA-H------MSLPDKAFPVSWDQFHRDARALAWRVAGMQREWRAMVAITRGGLVPAAIICRELGIRMIETVCIASYHEYDEQGELKVLKDVDSKLLENGGEGILVVDDLTDTGKTAAIVRAMMPKAHFATVYAKPKGRPLIDTFVTEVSQDTWIYFPWDMGLTYQEPITKGHRG-MTNNLDGALSFPVSVGRLPQKGLNIKIEANARELEAL-RDFHELLDVKFFKADLQIVPWKKDGVRVRGEVHAEIVQSCIVTLEPIDAKVDAEIDTIFVPENSRLARLPLDENGELIISAEGPDIPETFSGDTLDAGAIAEEFFELAIDPYPRKEGVESVPPVVVQYG--DDAVEEPANPFVALKDWKQKPMRIDAISVGNNPPEDVNVIIEVPVGGQPIKYELDKDSGALFVDRFLHTPMTYPGNYGFVPHTLSDDGDPIDVLICNTRALMPTSVINVRPIGVLVMEDNKGQDEKIIAVPSPHLTLRYEKVHNYTDLPEITLKQIQHFFEHYKDLEPGKWVKIGDWGDEDAARQLITEAIERHKAKKA-------MKAFESNHLRAEVHASPNFGPRKDGKRPSLLIMHYTGMETGEAAEHWLVNPASEVSAHYIVHEDGRIVQMVPESERAWHAGQSSWKGETDINSSSIGIEIVNGGPLLDFPEFESRQIDAVIDLCKGIVSRHGIKPEAVLAHSDIAPARKIDPGEKFPWPVLYNAGVGHWVNPSPVRGGRFFSVGDQGQPVEALQSMLALYGYGVPIDGVFGSTTELGILAFQRHFRPEKVDGVADMSTIETLHRLLSALPVLTA--MAQKKAHEVDSFLKRLDRAYP-IVLLYGPDKGLVSERAGVFIKLTGLAQDDPFAVIPLDADDINAEPGRLSDEANTISMFGGERLIWVRNAAGQKGLADAVAYLCKNPPADTFILVEAGDLKKGAGLRGTVEQSASAMALPCYSDDARGIDSTIDDMLARNGLQITLDARGLLKESLGGDRLATRAELEKVCLYAHGKERITVDDVRDIVGDVSATSYETMVDAVMIGNVTDFTHAFDRVIGTGSASYLILSAAIRQFQSLQTLRYGIENEGKNAASAVATARPPVFFARKKFVETALQRWTTQSCARAIERLQRTVLESRRNAPLAIAIIRQSMIALAAEAARAGR--NGRMTDDGFFREVNEELRSDKVKAIWTRYGSLLIGAVIAIVVGTGVFAFYEYWTEKRASQSGDQFLAALNLVRDGKNDEALKTLEQLEKDGYGAYPVLARMRAAGVIAQKGDLAGAVAAFDKVSADTSIPQAIRDLAKLRAAFILVDTGSYDDVASRVEALSSDSNPMRHSAREALGLAAWKAGRGADASKLFQQISDDETAPANVRQFADTMLDMLKSTGATPAAAAG-----------------------------MLASTVLASS--ASAQETP-----PLRGAVQENVLQAQ-----NTTNANQ-TNGANNN-PARQTDSGIPARPYEPVSPGALPQDDDSSNLGLLGVPIDDDSPPSGPADNSGGLNAESAAPAA-GTVPVPAQRP--GAVR---RAGVE------DRLDEEQTGSISNPPIEQNREANPPAAAAERDNLPVPAIERRT-IRPDPEPFAPLGIRAGTVTLRPSLSTGLRATTNADGSSDGSSAVLSETRLRARATTDWSRHSAFLDFDGTYDKTISGEEYSAPDAGLRGGFQLDLGERTTVKGEAGYRIRQEDPSAPTTIVGTSNRPLVQELDGSLGVRHEFGKFFADVKGNVDHTTYGNAEFSDGSTVSQGDRDNTFASIALRGGFEMSPAIKPFVEVELGKLMYDEAVDVNGFRRSGPLLGLRGGVELDIAEKLSGELAIGYLRQDIDDPRLAAIDGLSVDGALKWSPQRGTDVTLGLLTRVEGATAPDDSGSIFYEGTLGIKRQVRSNLDVHATLIGSLRDNTDGSGWDKGFGAEIGTTYWFNRFVGLDVSARHEFTHSEVDTRQTEETSIFMGVTLQRMGVVVSLDDKKTSTGSVQPLIDLTKADMGRVNELILSKAGSDVEMIPEVANHLISSGGKRLRPMITLTAARMFGYKGDGHVKLATSVEFMHTATLLHDDVVDESDLRRGKSTARMIWGNQASVLVGDFLLGQAFKMMVDVGSLEALDVLATSASVIAEGEVMQLAAAKNLETTEDEYLAVIKAKTAALFSAAAEVGPIIASASRTDRQALRSYGLNLGLAFQLVDDALDYGGNAKDLGKNTGDDFREGKITLPVVLTYRRGTSEERLFWRDALENGANDDKGLEKAKGLMTRYGALGDTIQRARHFGGIARDALAPLDQSPQKDALLEVIDFCISRVN--MSRPLGKKLAEKFGEEIRFFKGWMHGPKAVGSILPTSSITARRMASIVNPHSGLPVLELGPGTGVITRAILNRGVAPSDLYSVEYSPDFAEHLREDFPGVNIIEGDAFDLDATLGDKRDQKFDSVVSAVPLLNFPVGDRVKIIEDLLRRIPHGRPIVQITYGPMSPVPAGRGNYKIEHLDFIIRNVPPARLWVYRRVD-MTATGPDETNARKIPLLPRAIAAFVAIGAILLAFL--YPAEAFLWVKAIHVIAVISWMAGMLYLPRLFVYHCAAKAGSAQSETFKVMERRLLRAIIDPAMGVAWLAGLWLAWQSAAYMDGWFHVKFLAVLTMSGIHGYFAKAVRLFGEDRNEKTARSWRLMNEVPTVLMVIIVIMVIIKPFMDAVIKNLQSAAVK-QDNKRRPTQAEAEAAVRTLLLWAGDDPDREGLLDTPERVAKAYRELFSGYSQDPADVLGRTFEEIAGYEDIVLIQDIPFFSHCEHHMVPIIGKAHVAYLPDGEKVVGLSKIARVVDIYAHRLQTQEAMTAQIANAIQESLQPRGVAVMIEAEHMCMSMRGIRKSGSMTTTTTFTGTYKTDVNEQVRFMTLVRGR------MADHPTAFVTGFPIRHSRSPLIHGHWLKKYGLDGSYQAIEVAPEGFADFVHNLQNNGFVGGNVTIPHKESAFALCSRHDTAAMEIGAVNTLWFEDGVLWGGNTDAYGFVANLDAMSPRWDERKSALVLGAGGASRAVVYALKQRGFTDIRIVNRTVERAKELADRLGCGISAHEWRMVPELLGDSALIVNTTSLGMEG--KADESIDLTAAAPDALVTDIVYVPLETPLLKTARARGLKTVDGLGMLLHQAVPGFERWFGTRPDVTPELRKVILADMDRSARSGQPK--MRKTVAL--------FLSSCVVSAI---AGIST-ALAGANITVDAGTGEIVSQQDAFQRWYPASLTKLMTTYVAFRMIQSGQVTLDTPITMTANSVKEPPSRSGYKTGSELTLDNALKIMLVRSANDVAMAIGETLGGSEEKFSGLMNEEARRLGMTGSHFVNPNGLHSDDHYTTARDLAVLTLHLRHEFPQYARYFDIEAINYG-TKKSQTNYNALIGRFQGADGMKTGFVCPSGYNLIGSATRDGRTIITVVLGERTINSRVTKAADLLGKAFEEKGTGQ-TLDTLAPYGSASRDVAVNMRPQVCSKEAAGTDNWDGKDAEGHLVQGSAYLTRMDHQPKTEDIALLPAKVNSVG---------IEMSRIPVPKPRPDRASLTDASNA-LKAVNMRAQPKASHFINGRFVEDKAGKPLDVIYPATGEIIARLFGATPVLIEQAVQAAAAAQPAWAALKPVERGRILRRAADILRERNAEIAELETLDTGKAIQETLVADPASAADALEFYGGIIAGFNGEMIELGGSYAYTRREPLGVCVGIGAWNYPIQGAGWKSAPALAAGNAMIFKPSENTPLSALALAEIYKEAGLPDGLFNVVQGFGDVGSQLVDHPLVAKVSLTGSVPTGRRVLAQAGSHMKHATMELGGKSPLIIFDDADVENAVGGALLGNFYSTGQVCSNGTRVFVQGGIKDKFLDRLTARTKAIRLGDPLDPDIHLGPLVNAAQRNKVVSYIEKGKAEGATLHLGGGVPQMQGFE-GGCFVEPTIFTNVTDDMVIAREEIFGPVMAVLDFKDEDEVIARANDTEFGLSAGVFTKDLSRAHRVISQLKAGTCWINTYNLAPVEMPFGGFKQSGVGRENGLAALYHYSQIKSVYVETGKVESPY-----------MNDPANQSS-STKQALS-DE-----VKEDDIDIDMENDEDE----TPDASSEPPRDV-----AALISAIEWDSAVVSDGT-----SGADVIAALVKRLPNKPGVYRMFNEGGDVLYVGKAHSLKKRVSNYARGQGHNNRIARMIRETAKMEFVVTRTETEALLLEANLIKRLRPRFNVLMRDDKSFPYILLTAPKQTDRRLAPGIFKHRGARSSKGDYFGPFASAGAVGRTINALQRAFLLRTCTDSFYENRTRPCLLYQIKRCSGPCTGEISDADYDELVSETKAFLSGKSQAVKTHLSDAMREASADLDFERAAVYRDRLSALSHVQSHSGINPQSVE----EADVFAIYQDGGVTCIQVFFFRTGQNWGNRAYYPKADSSLSGA---------------EVLGAFLSQFYDDKPCPALILLSETVEDQELLGVALTAKSDRKVTISVPQRGEKRDLVEHALSNAREALGRQLAETSSQSRLLKGVAETFGL---EKTPRRIEVYDNSHIMGTNAIGAMIVAGPEGFVKNQYRKFNIRSTEITPGDDFGMMREVIERRFSRLVKEHGTPETKDAPT----EDIEIDEFDDDSDAFPAWPDIILIDGGQGQMSAVRGILQEMGIADKVTAIGVAKGVDRDAGR-----ERFFMEGKPSFTLPPRDPVLYFLQRLRDEAHRFAIGTHRAKRKKEMVKNP-LDEIAGIGPSRKRALLHQFGTAKAVSRAAVEDLMKVEGISEAMAITIRDHFRT--MTDTSTTIDYSKTLYLPQTNFPMRAGLPAKEPEFVARWQEMNLYRKLREEAKGRPLYVLHDGPPYANGNIHIGHALNKILKDVITRSFQMRGYDSNYVPGWDCHGLPIEWKIEEQYRAKGKDKDDVPINEFRKECREFATHWIKVQSGEFKRLGIEGDFDNPYTTMAFHAESRIAGELLKFAMSGQLYRGSKPVMWSVVERTALAEAEVEYADIESDTIWVKFPV-------VKGPE----------------DLGQA-------------HVVIWTTTPWTIPGNRAVSYSPRVAYSLYEVESVENDFGPRPGEKLIFADALAEESAIKGKMTFKRIRTVSADELASLTLAHPFHGLGGGYEFAVPMLDGDHVTDDAGTGFVHTAPGHGREDFEAWMNAARDLEVRGISPAIPFAVDDAGYYTKDAPGFGPDREGGAARVIDDNGKKGDANKVVIEELIARNMLFARGRLKHSYPHSWRSKKPVIFRNTPQWFVYMDKDLG-------------------------------------------DQT-----------TLRSRALSAIDETRFVPAGGQNRLRAMIEDRPDWVLSRQRAWGVPICVFADENGNVLQDEAVNARILAAFEEEGADAWFAEGARERFLGEKANEPWTQVRDILDVWFDSGSTHVFTLEDRPDLKWPADVYLEGSDQHRGWFHSSLLESCGTRGRAPYNTLVTHGFTMDEEGRKMSKSLGNTVTPQDVIKDSGADILRLWVMTTDYWEDQRLGKNIIQTNIDAYRKLRNTIRWMLGTLAHDEGDEIAYADMPELERLMLHRVAELDELVRSGYDAFEFKRITRALIDFMNVELSAFYFDVRKDALYCDAPSSTRRKAALQVVRHLFDRIVTWLAPMLPFTTEEAWLELHKDAVSVHLEQFRDTPAEWRNEQLAAKWRKVRDVRRAVTGALELERADKRIGSSLGAAPVVFITDPELLAAVKDIDMAEVCITSAITIKDETPPANAFVAEDVKGVAVVPERATGTKCARSWRYTHDVGSDAAYPDVSARDAAALRELAQLGKLA-MATVAFIGLGVMGYPMAGHLNSRGGHDVTVFNRSSEKAKKWAEQFKGAHAPTPKEAAADKDFVFSCVGNDDDLRSVTIGENGAFHSMKNGAIFIDNTTASAEVARELDAEARKKGIHFLDAPVSGGQAGAENGALTVMVGGEQDAFDRAKPVIESFAKMVGLMGPVGAGQLTKMINQICIAGLVQGLAEGIHFGKRAGLDIEKVIEVISKGAAGSWQMENRHKTMSAGKYDFGFAVDWMRKDLGICLAEADRNNATLPVTALVDQFYKDIQVMGGNRWDTSSLLARLEK-MKRLAAGLLTLLL----LTGCTTVDYDFSEAKRPS---AVQQIAAPSGKVGAARFGDRDPHDWNGKSPWGYPIHGTDISKYQSKIDWQTVRGNRISFVFIKATEGGDRVDDRLAENWAAARAAGISRGAYHFYYFCRTAKDQARWFIRHVPKDPDALPPVLDMEWNAASPTCKLRPNAAVVRSEMRTFLDIVERHYGKRPIIYTTVDFFDDNDLRQFVGYPFWLRSVAGHPDEKYGPHPWTFWQYTGTGSVPGIRGDADINVFAGNESAWKQWLVRNKARMAKDALIVVDVQNDFCPGGALGVEAGHEIVPIINRLMVRFDHVIITQDWHPAGHSSFASSHPGKAPFESIMMPYGEQTLWPEHCIQGSVGADFHHDLDWTKAELVIRKGFRPHVDSYSAFFENDHKTPTGLAGYLRERKITNVTFAGLATDYCVAYSALDAVEHGFAADVLLDACRGIDLGGSLAAMITKMRKAGVTLIMAKPTASPLRTGLVHLALGTYTLIALFPVFLTVINSFKDRASIFRDPLGLPTPSTFSLVGYNTVLGQGDFVLYFQNSFIVTAVSIFFVLLFGAMAAFALSEYRFKGNTMMGLYLALGIMIPIRLGTVAILQGMVAAGLVNTLTALILVYTAQGLPLAVFILTEFMRTVSDDLKNAGRIDGLSEYSIFFRLVLPLVRPAMATVAVFTMIPIWNDLWFPLILAPSDATKTVTLGSQVFIGQFVTNWNAVLAALTLAILPVLVLYLVFSRQLIRGITSGAVKMP---ILRLTDPSHLPEHLKGAVVAIGNFDGVHRGHQAVLERALEAAHGVKKPSLVLTFEPHPRSVFVPDQPVDRLTPAPEKALILEALGFDAVIECPFTREFSQLTADVFVDRILVGGLAASRIVTGFDFHFGRNRQGGPAFLMDAGEKRGFHVTLVDAFRDEGGQAISSSRIRELFGEGEVVEAAGLLGYRHRIRAEVIGGRKLGRTIGFPTANMVLPPETHLKHGIYAVRFRRADGTLYDGVASFGRRPTVDTDGEPLLETYVFDFSGDLYGESCAVSFFGYLRGEEKFDGLEPMMQQIKRDEEEARALLQGVRPLSALDQRINFD-----MTVERDFPDSLPYELRQPADQRIPFVFNSPHSGQCYPAAFVRQSQLDPQTIRRSEDCFVDQLFAAAVMLGAPLMVAHFPRAYLDVNREPYELDARMFLEPLPPYANGNSARVAGGLGTIPRLVGEGQEIYAQKLHLDEALGRINNIYKPYHAVLGDVLVATRDRFGYGVLIDCHSMPTGIRFPESGMRPDFIIGDRFGTSCTPELTNSAIHLLREMGYVVAHNKPYAGGYITEHYGRPLKSFHAMQIEVNR-------------GLYLNEHNYERKAGFDQLQRDIGLF-------LADLVSLPDFHFLDTPLAAE---------MREEILSIPAVTARFLDASRDTLAAAGAKLREKDPPFLASIARGSSDHVSAFLKYSSELTAGIPVASLGPSVASIYGVTLKLGSAATLSISQSGKSPDIVSMTKAASESGALTIAITNVINSDLANASDFPIDILAGPERSVAATKSFVSSAVAGLALIGHWTEDKQLLAAIDALPTALEKAVDCDWSPFATTLKDENSLFVLGRGPSLAISNEVALKFKETSAVHAESYSAAEVMHGPKAIVGKGFPVLVLAARDAAEDSIIESANRLAEQGAAVFVTSAKPSKATAVPFVATSHPLTDPLALLVSFYAFVEAFARSRGLNPDEPPHLRKVTETI---------MRQDNVKAQFRGSKRN--------------------------AIALVGAAIFLFSPTITAYQDMASLLSGSESGQGRWTSYLEKSPAGSIQKANMSFVDETAITSSVPASGVNAPGIGRIAI-NGSQKSP-----DATPDEDRINRAEKQGRIVSVSKKAPPKAFSAGSILQRSSLLIRPSHGVEVEMAFAKPKIAGKEIQIALAFHKRAPEKTADDIAPMLAGLVNNDQPDVLALGYAPATPDYSKTSPFDSILSEPKESGRFVPQLGKGDHNWLATPLPPVVFTKEEQKCLATGIYFEARSESVKGQAAVAQVILNRVRNPAYPKTICKVVYQNNDWINRCQFSFACEGRKLNVTEPKQWKVAQEVALAVTSGRIFLPEIGSATHYHAVYVRANWAHTMKRIDRIGQHIFYRTYGGGWI---MKILVIEDDREAARYLEKAFAEAGHSADVAGDGETGYTLAEQGSYDALIVDRMLPRRDGLSVVAALRAQGNDTPALILSALGQVDDRVTGLRAGGDDYLTKPYAFSELLARIEVLQRRSSPKEAETIYRVGDLELDRLSHTARRAGQNIVLQPREFRLLEYLMRHAGQVVTRTMLLEHVWDYHFDPQTNVIDVHVSRLRGKIEKGFDTPLLHTVRGAGYMLKAS----PTGTNA---MMTQWFFLHVDRRGSIVIALLVAIAVLVPLSNLLLPVTSPLHVPTYLVSLIGKYLTYAMLAVALDLVWGFCGILSLGHGAFFALGGYAMGMYLMRQIGPRGVYADPVLPDFMVFLNWKELPWFWYGFDSFWFAALMVVLVPGVLAFGFGWLAFRSRVTGVYLSIITQALTYALLLAFFRNDMGFGGNNGLTDFKDILGFNIQAGSTRSVLFAATALFLALAVFVTSFIVRSKLGKVLVGVRDAESRMRFLGYRVDRYKLLVWTISAMMAGIAGALYVPQVGIINPSEFEPANSIEAVIWVAVGGRGTIIGPIIGAVLVNFGKTYFTAAFPEIWLFALGALFIVVTLFLPKGILGLYGMWKGKRADRAARKKDVA-PPPVAAVSG-RSPIPEPQAAEMSNTDNPFGGGYGAT-SGAQTVSFGT--APNGSAASAGE---VIMDTTTAGFSKDVIAESKNQPVLVDFWAPWCGPCKQLTPVLEKVVRAAGGRVKLVKMNIDDHPAIAGQLGIQSIPAVIAFSDGKPVDGFMGALPESKLKEFIDRIASPDGGRDEAIRSALEAAAHAVEAGDLEQASQIYAAVLAEIPDHVPALAGLANCLIELDDIEQARATLERVPADKREDAVVRAADAKIALADQVATLGDPVALEARLSDNPKDHRARFDLAMILNAKGQRNEAADHLLAIMRADRAWNDDGARKQLLQFFDAWGAGDEASVTARRKLSSLLFS---------------MSRITTIEQLETIYSG--ITEASTAKVTDQITPEYRQLIEGSPFVALATSGPEGLDCSPRGDLAGFVRVHDDRTLMMPDRRGNNRVDSLRNIVRDPRVALLFLIPGLTTTFRVNGRAFLDTDQALLDTFMVDGKVPRSVIVVEVMEAYFQCARALIRSELWNPAKQRSPKDFPTPGQILAALSEDRVGGESYDREWPERAAKTMW------MLTVENASLHYGAAQALRGVSLEVKPKSITCVLGRNGVGKTSMLRAIVGQHPLSSGSISFDGSPLGKKPPYDRARAGIAFVPQGREIFPLLTVRENLATGFAAAKRGDRNVPPEVFELFPVLKTMLGRRGGDLSGGQQQQLAIGRALVTRPKLLVLDEPTEGIQPSIIKDIGRAIQFLRDQAGLAIMLVEQYLDFCRELADHVYIMDRGEIVHSGDAASLDRPDVRKFLTV----------------MTDEKRETPIDRKLLELLVCPLTKGPLTYDQQRSELISKSAHLAYPVRDG------------------------IP-----------------------IMLPSEGRAIED----MTIRTLVWGENVHEKVNKTVAEIYPEGMHNQIAKLLASDGNIAVKTTTLQEPEHGLSDKVLDETDVLIWWGHRAHGDVADEIVERVAKRVWEGMGLIVLHSGHFSKIFKRLMGSPCALHWREAGERERLWVVNPGHPIAKGLPAYFELENEEMYGEPFSVPEPLETVFVSWFQGGEVFRSGLTYRKGAGNVFYFRPGHETYPTYHDETVGLVLRNAVNWAYNP-NRYPEITTAPNVPVEKALEPIVARGGSLHEAGEEGFR-----------MHVIKSWFWPGVITTALLTALAGWFLAGPVDQQLTDTVNAALESQNA-WASAEVDGRDLILKGIAPSEEALAEALKIARETFGVRVVENAATLLPLAEPFSFVVTKSDDGILLSGNVPYGDARAKILAAAETAMPGIEILDEMAVARGEPQGFFDLANFALMQAAQLTNGEVEVSGETYSISGTAANAADYESINAALRLALPGNGKPGEIKLEAPASASSN-----------------------------MAFEDIKARIALLLEAMVQRPTDAHEIHENLREQLNELKAMGMPLPDDLVELEKQLEADFDSMLDSLRNVAQTWVVKVLMGLLVISFAGWGVSSTILGAIGGNAAIRAGNSEVSPVDYRLAYDRQLAALSQRFGQRLTREQADAIGIGHQVQAQLVAGVVLDEQARTMSLGLSKDRLAQLAAEDPAFQGADGRFNPAQFDAVLRNAGMSAQNYLDNRAQVARRQQIVEAVADGIKVPDTLLKAVAIYKGESRTVDYVSMPKSFVSDIADPSDNDLKAYFEANKEAYSAPQYRKVSYVKLEPKDIANPASVTPEEIKTEYDKNIARYSTPETRTVEQLTFANDDAAKAAHDKIVAGTSFEDIVKAEGKTMDDVRLGTFPKTALPDQSIADPIFGLQANGVSDVLKGGFGPVIVRVSAITPEVVKPLAEVEQDIRETIALTQAASDIADVHDAYENARAEGASMSEAAAKQNLKLVTIEAIDQTGRGPDEKEVANLPFDDNQLAAIFQADVGFDNEPFNLGTNSYLWYDVDSVTPARERPLDEVKPRVIEHWKTAELEKRLNEKAEEVRKRVADGTSLDDIAAEFKFTKDTKRGITRGSKDVDLGEGGVASVFDGPQGLVGVTESASDGSKLIFKVAESIEPANVGPESVTTVEREAFSSRLADDLLDQLVAQLQTVYPVTINQTVINQALAR-MAK---TTTGGRGGTGTRGLHTKIKKKAGTIKESSRRWLERHINDPYVQRSKQDGYRSRAAYKLIEINDRYKLLKKGQRIIDLGAAPGGWSQIAASIVGSPDDFPTVVGIDYLHVDPLPGVTLLEMDFLDDAAPEKLIEALGGKPDIVLSDMAAPTTGHRRTDHIRTTHLCEVAADFAVSVLRPGGHFLTKTFQGGTENELLTLLKQNFKSVHHVKPPASRAESVELYLLARDFKG---------------MSIVESPCILVCAIDINTGYCFGCGRTREEIGAWSVMSPESRRTIMADLPGRLETVERRPRRETRRSRMAR----EKQQEAMAQFHVIAGASETNVYPEVRRISVADVFAALREGLDDFWDKPSHYVFLCLIYPIAGLVLARWTSGANLLPLLFPLMSGFALVGPFAALGLYEISRRRELNLDTSWRHALEVRHSPAIPSIVAVGIMLLVLFVAWILTAQSLYTSLFGAEAPASIGAFLNQVFTTPEGQRMIILGNAIGFVFAVVVLCTTVIAFPLLLDRDVGALSAVVTSVKAVLTNPVEMALWGLIVAALLLVGFLTLFVGLAIFIPVLGHATWHLYRKVVVPLPREVTSAGR--------MRYAIYFTPPSSDPLLKVAANWLGRNAFTGQPVKAPQIRDLAHEDFAQLTESPRRYGFHATLKAPFHLVDGVEERELLSALMHFASSMDPVEIPKLQIACLDGFFALVPTEPLDNLNQLANDVVVAFDRFRAPPSEKEIKRRNPETMSIAQRRNLEQWGYPYVFEDFRFHMTLTGPVPERDRPKIEHILTEFLEPVLEEPVEINNLALFTEAERGFPFEIHSLHPLA-----------GTN--R-RKTA-MARSNQTADWGNRLSPSLGEIESLAIQAYAHLPETFRALCGDITIQVSEFPDDQIVEDMGLESPFDLLGLFEGTGIGERFSLQTGEQANRITIYRRAVLDYWAEYEEALGDIVTHVLIHEIGHHFGLSDEDMERIEATVE---------MIKRFVATLLLVLFAAAPGYAVQIKEVVSPKGIRAWLVQDDFVPLISMRFSFKGGSTQDPEGKEGLANLMTGLFDEGAGDLDSNTFQEQLDDVGAEMSFNADDDRITGSVRMLADKRDAAAELLTLAVNQPRFDQAPIDRIRQQVVAGINASERDPNTIAGRKFAVALYGDHPYARQSDGTEQSLNTITRDDLVAFHKRNFARDNLIVGVVGSISPEDLAPLLDKVFGDLPEKAQLEPVSDAQLGFGQTTRFDYVLPQTSISMVYPGVRRQDPDFFPAYIMNHILGGGTFSSRIYNEVREKRGLAYSAGSNLINRDHMAALMVSTATRADRANETLQILKTEIARMAKDGPTEEELAEAKKYLVGSYAVNNLDSSSAVASTLLGLQDENLGRDYIDKRADLINAVTLDQVRAVAGKLLETEPAVLIVGPPQSMNTEIFGHTPDGEAVHRITISGGGLTAKVLTWGAVIQDLRLKGHESPLVLGYQDFADYPAHSPYLGATVGRSINRIRDGDLTIDGTTYSLERNFRGLHNIHGGSEGAGNRNWEVVAVGADYVTLTIQLGDNEMGFPGNLNITCTYMLEPKGTLAIRMDAVTDKPTICNLAHHSYFNLDDGGASDALDHQVRIGAEAYLPVDGDLIPDGKVLPVAGTTHDFREYRTIRREENGDQVVYDNNFCLSSQRQPLRPVASAKGARSGVEMSVSTTEPGVQFYAGNTLGGRPPIGLTGNTYGNYAGFCFEAQVWPDSTHFPYFPQAILRPDETYSQMTKYSFRKAKA----MTLNFEDLRIKMVDNQLRTTDVTDKPLLQAFLDVPREKFVPSAREPLAYIDDDVLLATT---EAGARYLMQPSPFAKLLQLATIHPDDTVLDIGCATGYSAAVLARIARSVVAVENEADLADQARSILPTLGIENVEIVQGALQSGCPGKAPFDVIIIEGSVDQVPGTLFEQLKDGGRLVVVEGAGNAGKSMLYLKSDGIVSGRRAFNSAVKPLPGFKKAAEFEF-----MAQLIFFALVGAAAYYGYRSFKREAHRVSQRVREAEKEVQNHAQGTLVQDPETGEYHVRKDMTDLTALTIAEARDQLKAKAITATELTGAYIQAIENANEALNAYVVTTPEKALDMAKASDARLAAGNAGALEGIPLGIKDLFGTEGVHTQACSHILDGFKPHYESTVTANLWADGAVMLGKLNMDEFAMGSSNESSYYGPVKNPWRAKGSNTDLVPGGSSGGSATAVAARLCAGATATDTGGSIRQPAAFTGTVGIKPTYGRCSRWGIVAFASSLDQAGPIARDVRDAAILLKSMASVDAKDTTSVDIAVPDYEAAIGKPLAGLKIGIPKEYRVDGMPEEIEKLWQQGIAWLKDAGASIVDISLPHTKYALPAYYIVAPAEASSNLARYDGVRYGLRVPGRDIVDMYEKTRATGFGDEVKRRIMIGTYVLSAGYYDAYYLQAQKVRTLIKKDFEDVFHAGVDAILTPATPSAAFGIADQDMSADPVKMYLNDIFTVTVNMAGLPGIAVPAGLDTRGLPLGLQLIGRPFDEETLFQTAHVIEQAAGKFNAPKWWMFGMARVVKSMGFAMTVFVASL---GANSALASEAFMTTGGLTSQPIGHYEFCKRQPNECSIRSRNVMPEKMNHDFWQLIVNVNSNDNQQVKPLTDMEIYGAEEYWSYPDK---VGDCEDYVLLKRRDLMKAGISPANLLITVVRKPDGEGHAVLTVRSDKGDFILDNLVDGVKNWSETEYTYLKRQATNNAGRWVSIEAPTNILVGAVQ-----------------MGGARP--------SLHHGTNQSGMRDHNERVVLSLLRQHGGLAKTAIARMTGLSAQTSSIIMRMLEADELLLREEPLRGRIGQPLIPMSLNPEGAFFLGLKIGRRSADLVLIDFVGQIRSMLHMPYDYPAPEPIFRFVAEGTAEMMAGLTEKQRERIAGIGIAAPFELWKWADTVGAPQEIMDQWRNCDIRERVAALCPFPVYLQNDMTAACGAELVFGQTKDVNDFVYFYIGSFIGGGIVINGSLYAGRSGNAGALGSMPVPGA----------DG--SARQLIDVASITALEKVVTDLGRDPSPLWASPEDWGAMGDELDAWIDQAGKGIAQAVIAASSVVDFEAAIIDGWLPVSVRARIVESVRRNVETFDLEGIELPVILEGTVGIHARSLGAASLPLSDRFLLGQK-IFRKGNMPGSNRSFATNPMLANDAPYTIDAFHRGRFHLLQPAAKGHRSGVDAMILASVVPDEFKGHVADLGAGAGAAGLAVLSRCSTARASLIERSDFMADFARRSLALPENKAFASRAEVIEADVTLTGRARVAAGLGDNGFDFVIMNPPFNPPTDRSTPDPVKAEAHVMTQGMMEQWIRTAAAIVKPGGAIGIIARPTSIADLLDALKGRFGGLIIVPVQPRPQDAAIRIVIKGIRGSRAGLSLHPALVMHHETGNGFTERANAINNGLASLF----MTDPQSTALTPSEIERYARHIILGEVGGPGQQKLKRARVLVVGAGGLGAPVLQYLAASGVGTLGIIDDDRVSLSNLQRQIIHSTELVGSAKVESAARAIAAINPHVTVEAHETRIAPDNVAELVRRYDIVVDGSDNFDTRYLLADTCLEEKRPLVSAALGRFDGSVTTLIPYAANAASEPNPSYRDLFPNPPPPGLLPACAEAGVLGVLPGVIGTLQATEVIKLITGIGEPLIGRLLLYDALTSRFETIK-----YKARKA-----MESTLKQTLIDAIRNIPDYPKPGVMFRDITTLLGNARAFRRAVDELVHPFAGGKVDKVAGIEARGFIIGGAMAHQLSSGFVPIRKKGKLPHDTVRVAYSLEYGVDEMEMHRDAIAPGDKVILVDDLIATGGTAEAAVKLLHQMGADIVSACFMIDLPDLGGRKKLEALGVNVRTLIAYEGH---------------------------------MIDWTDRHCRTFHRQFTRHALLYTEMVVADAAIHGNRDRILGFAEAEHPVALQLGGSDPAKLAAAARIGEAMGYQEINLNVGCPSDRVQSGTFGACLMQTPGVVAACVEAMKQVAAVPVTVKCRIGVDDQDTEVALDELAGSVFNAGANALWVHARKAWLKGLSPKENRDIPPLDYDRVYRLKRQNPSHFIGINGGVQTLDEAFEHLLHVDGVMLGRAIYHNPMLLLDVDARMYGDGAADSPSMDAIIDHMCAYADTHIARGGRLSHITRHMVGLFHGAPGARRWRQLLSTAANQTGATSTVIRQAYEVIQTAVTDEAA----MSQNSKQILISLALALSLSTSMTASGFAEEKAAAAIHEAKAPVINVVPASQNEMVATLTVTGTIVPRQEVAVGTDVAGLLVLELNADQGDVVKEGDILARLDKSSLEIQLAQIEAQRASAEASIAQSEAQIVDAEIAVRQALEALGRARALSAKGINSKMELDNATNAHDSANARLNTARQALAATKSQLQLVAAQKRDVMLRIQKADVRAPASGVVLSRNALLGGIVSFNAGPLFRMARDQDFELAANIPEADLPRLKENMPVAVRVSGMQEPVAGRVRLISPEITASSRLGSVKIALDRNPAIRPGNFARAIIELARRDGISVPLSAIVYHGKSALVQVVKDGVVESRKIALGIRSDRTVEVLQGLNEGEDVVARAGTFVANGDRVTAVRVTNE---------------ATGAVK-------------MRVLGIETSCDETAAAIVERDDQGHGRILSNVVLSQIADHAPYGGVVPEIAARAHVEVLDRLIGQALQEAHITLDDVDAVAATAGPGLVGGLIVGLMTGKAIAMAAQKPFYAINHLEGHALTPRLTDRIDFPYLLLLVSGGHTQMVLVKGLGDYERLGTTIDDALGEAFDKTAKLLGLPYPGGPEVEKAAVLGDPDRFTLPRPLKGEERLDFSFSGLKTAVRQLATTLEPLSQTDINDICAAFQTAVADTLDDRVSRSLRRFRQSFPDVEEPALVVAGGVAANKVLRDGLQELCSENGFRFVAPPLALCTDNAAMIAWAGAERASDQAPADSLDIAPRSRWPLDMQSTPLIGAGRRGAKAMALDATFFALVGLVIFLLLVVFLKVPGTVGKSLDARADRIRDELEEARRLREEAQSLLAEYQRKRKEAEKEATEIVAIAQREAHGLFEEARKKTEEYVVRRNKLAEQKIAQAEVEAVNEVRASAVDIAIAAASRILADKVDTKTSSDLFKSSLTEVKSRLNMLALIRTIDLALDIYTWIIIASAIFSWLFAFNVINSSNRFVASVGDFLYRVTEPALRPIRRFLPDLGGIDISPIVLLLIIFFIRQLLWTTIAPMLV----------------------------MALAVSSALAVL-----PVSGTAVAESNVVKLNQAQGASKRINLGLSKSLVVDLPADAYDILVANPTVADAVTRTARRIYLFGKQVGQTNIFVFGPNGEQLASFDLVVERDVAGLEDSLKKYIPDSDIKVELMNDNVILTGTVQTPLDAKRASDLANLLVSGGEATTGQYAITASGQGGSNGGGGSDVAISDPDEARQTSKIVNLIQIAGEDQVTLKVTVAEVSRTVMKQLGVNLVGKTKSDGIFFS--STNIASLVGKGLTNSGITAPISIGSATIDSYLNAMELAGVMKTLAEPSLTAISGEQAQFKVGGEYNLITGQTVNNQGGI-----QYTITKVEYGIGLEFVPVVLSPGRISLKVRTSVSEPTLEGSVSLGAGVNSSFDGVGNKNRAQQPATNLISLRKRLADTTVELPSGGSMMIAGLMRDENRAAISGLPGMSKIPVLGTLFRSREFVRNESELVIIITPYLVRPVPRTALARPDDNLNPASDAAGIFLGKVNRVYGTKEANLPDGRYEGAVGYIYK-----MTRPNILILMVDQLNGTLFPDGPAEFIHAPHLKALAQRSARFANTYTASPLCAPARASLMSGQLPSRTRVYDNAAEFVSDIPTYAHHLRRAGYYTGLSGKMHFVGPDQLHGLEERLTTDIYPADFGWTPDYRKPGERIDWWYHNLGSVTGAGIAEITNQMEYDDEVAFHARQKLYQLSRESDVASRRPWCLTVSFTHPHDPYVARRKYWDLYEGSPALDPVVGGIPFDEQDPHSQRLMEAAEHREFDITPEQVRLARQGYLANISYIDDKVGEILEVLRTTRMLDDTIIVFTSDHGDMLGERGLWFKMNFFEGSVRVPLMIAGKGIAPQLISQPVSTLDINPTLAALGGIDIAEVLPWTDGEDLTRLMQGKD-RIGPVLMEYAAEGSNAPLVCIREGRYKFVHCEIDPPQLFDLESDPHELTNLANDPAHAKLLADFMAKARGRWDMRAFDAAVRESQARRWVVYDALRNGSYYPWDYQPLQKASERYMRNHMDLNVLEENARYPRGEMSLLEIEIARDLHPVDVIEHVAHTNEWAFERTGEDEIAITVEGNWTDYQISFSWMEDFEALHLACAFDIAVSEPRVNEVMRLLSLINEQLLMGHFDLWRQEGAVMYRQSLLLSGGAEPTSKQVEVLLSSALDACETYFQAFQFVVWSGSSARESLDSILFETVGSAMSLPDRFQPVPKR---LLCA----CVLGMALVVGGCQVRPLYSDPSPVASTGAGVNGSVRSRLATVAINQPGTREAQEVRNHLIFLFAGGAGEPASPAYSMQLSVAPQYLSLMLVQ-NATNDKSGQPTAGLVRMTGSYVVTRISDGQVVGKGSRLVTANYDAPRQRYAVTRAQRDAANRAARELAEALNLSVAQDLSKF-----------------------------------MKKTGAKPVWAGREFRLDPFHLPQTVTYATRDDRGDITFTLHERGAVVKRLLPASHLPVSLALPACAFLGVTARAAEDDLGDITVTLELMHTDPHLSVPLLVAHDLNDIAADWRAWSTLFKLPMMLVEEDGVARQLEQSIGPVTVSAPKERRQGREPRRRRPRFLARRKMGDLGLRLVIGGEEIIAQGAK--MADAKIEPRLKKQYQEVVRKALLEQFKYDNEMQIPRITKVVLNMGVGEATADSKKPTVAAEDLALIAGQKPVITRARNSIATFKVRENMPIGTKVTLRKERMYEFIDRLITIALPRVRDFRGLNPKSFDGRGNFAMGIKEHIVFPEINYDKVDQIWGMDIIVCTTAKTDDEARALLRAFNFPFRQ-MIELKNIEKRFGDHTVLNDVSLSVAEGAVMALVGPSGGGKSTLLRCINLLEIPTSGSVEIAGDRLEFQPGLRLNARSVQMLRRHTGMVFQNFQLFPHRTVVENVMEGLVTVLKWPKARAHERATMLLEKVGLAHKAGAWPSTLSGGQQQRVAIARALAPSPKVLLCDEPTSALDPELSGEVVDVLAQLAREGTTMVMATHDLRLASKIANEVIVLDGGVIVETGTSREIFTQPKRERTKRFIATLTHEEG-HTHGEGIMTSAWPNELRVSKDRRSLRIAFDNGESYELSAELLRVASPSAEVQGHSPEQRVTVPGKMNVEIVKMEPVGNYAVRIVFDDMHDTGLFSWDYLMDLGRNRDARWQTYLDELRKKGLSREPVSPRRAHMNIYAIKAIYLFEMHRTWRTIMQSVISPVVSTSLYFVVFGSAIGGRIPEISGVSYGAFIVPGLIMLSLLTQSISNASFGIYFPKFVGTIYELLSAPVSYVEIVIAYVGAAATKSIILGVIILATASLFVPLRIEHPFVMLLFLVLTAVTFSMFGFIIGIWADGFEKLQLVPLMIITPLTFLGGSFYSVDMLPPFWQTVTLFNPVVYLISGFRWSFFGLSDVHIGTSLAMTAVFLIACMLILRWIFRTGYRLKS-----MSKPDLTSLEKTIDKAFDERDAISTETRGEVREAVETSLLLLDSGQVRVAEKQADGAWTTNQWLKKAVLLSFRLNPMGIIEGGPGGAPWWDKVPSKFDGWGATEFAKAGIRTVPNAIVRRSAYIAPGVILMPSFVNLGAYVDEGTMVDTWAGVGSCAQIGKHVHLSGGVGIGGVLEPMQAGPTIIEDNCFIGARSEVVEGCIVREGAVLGMGVFIGKSTKIIDRATGEIFYGEVPPYSVVVAGTLPGKPLPNGEPGPGLYCAVIVKRVDEKTRSKTSINELLRDMRRVVVTGMGIVSSIGNNVQEVLESLRTAKSGIVFAEEYAKHGFRSQVHGAPTLDPSTLVDRRAMRFHGGGTAWNHVAMDQAIADAGLEADLISNERTGIIMGSGGSSTRTIFESAEKTIESGSSKKVGPFAVPKAMSSTASATLATWFKIKGVNYSISSACATSNHCVGNAYEMIQYGKQDMMFAGGCEDLDWTLSVLFDGMGAMSSKFNDTPSVASRAYDKNRDGFVISGGAGVLVLEELEHAKARGAKIYGELVGYGVTSDGADMVAPSGEGAARCMRMAIANVKGSIDYINPHATSTPVGDLKEIEAIREVFGSGNQ-CPPISATKSLTGHSQGATGVHESIYSLLMMNNDFIAESANIEELDPAFGDMPIARKRIDNVKLNRVLTNSFGFGGTNASLVYQRYED-MAGSGSNAMSGADGDGRHIPVLLDEVIAALQPAKGQLIIDGTFGAGGYTRRILETGASVTAVDRDPSAIEAGRSLEKEYGGRLTLVQGTFSTLDQVALL-DAPDGIVLDIGVSSMQIDEAERGFSFQKDGPLDMRMSSSGPSAADVVNRLKTGDLARIFNFLGEERHAGRIARMIEKRRASEPFTRTRDLANAIEALVGRNPKVPIHPATRVFQALRIFVNDELGELVRALHAAERVLKPGGRLVVVTFHSLEDRIVKRFFSDRSGTGG-GSRHLPQLEQRLASFTPVGKGIVVPTEAESARNPRARSAKLRAGVRTDNPPLAEDHSIYGLPNLPVFHETVR------S-MLYALLCTDKPDHLQVRLDTRAAHLDYLNGLSDQIKFAGPFLGEDGKPNGSLVVVDAADPMAAKNIAASDPYSKAGLFASVEIRPWNWAIKNPDNNMADVQNNSDIAAMSFEQALDQLEKIVDDLERGDVPLEQSIRIYERGEALKKHCDTLLRAAEDKVEKIRVGRNGEPVGTEPLDPE-MKVWKQLALALVILAVAGAGWMYLFPGGKDLLRRVGIDRNA-TAATEPAGGRQGQRRG-G-----QALPLVVVRAAGEAKINDRLTAIGTGKAKSSVAVTPFTAGRLTEIFVTSGTKVQGGDVIARLDSEAESIVVDKARVALKDAQTKLSRAESLRLSNTVSAVQLSEAELAVDNAALNVREAELALDRRAIKAPIGGIVGILPINAGNYITTSTTVAMIDDRSDLLVDFWVPERFAPAITIDLPATATSIARPGETFSGAISAIDNRVDPASRTLHVQAKITNPKDTLREGMAFQMSIGFPGDTYPTVDPLAIQWGTEGAYVWRIVDNKAERVLVRIVQRNTASVLVDAALDPGDQIVAEGVQSVREGGLVQVQGQPAPEN-NTKAGPLAS-AGEQG-----MAVSRRPFAPMRNLNAKVAAVPMIVTVFVVFVGCTLWTVIYSFTSSRSLPTLDFVGMDQYVRLFRTSRWTVSLKNLAIFGILSLTFSLVLGFILAALMDQKVRFENTFRTIFLYPHALSFIVTGLVWQWILTPAYGVQKVVRDLGFETFNFPILTDGRFTIYAIVIAGLWQGTGLVMALMLAGLRNIDDEIWKAARVDGIPAWKTYLFIIIPMMRPVFITTLVIISATIVKVYDLVVALSNGGPGISSEVPAKYVYDFMFSRGNLGQGLAASTIMLTTVLIILVPWAMLEFRPEKRRGGGSKMASKTNPITFFQQVRAETAKVTWPSRRETLISTAMVMVMAFFAAIFFFAADQLMAYGIDLVLGLGRMIAPQQKPEVTDM----QLHAGQ-----------RLPVLKRLSGKLLRLTLLFGLLAEVMIFVPTVADMRMRWLSDRLNTVAAASVVLAASSDKDIPQSVQNDVLLATGTKAIALSEQGASRLLAVSEMPSKVDKHIDITVTDPVTMIRDALDTLINGGHRVIRVYGPVGDTGKIIELLTSDAPLRNAMLHYARNVALISLFISLITASLVFLAISRLLIRPIQRMSANMLAFAQSPDDPQRIIKPESREDELGIAQRELADMQRQLQRTLSEQKHLADLGLAVSKINHDMRNILASAQLMSDHLANTSDPTMQRFVPKLVRTIDRAIHYSQTVLAYGGTQEAPPQRRRVKLRTVVNDVEEMLNLDA------KSGIEFRNLVPESLEIDVDPDQFFRVISNLCRNSVQAMAGDTRSDASVIKRLTITAGQIGTTSIIGVEDTGPGLPPKARENLFTAFKGSARSGGTGLGLAIVHELVRAHGGTIELRESEGAGTSFEIRIPDQPVSLAEWRSRRDSAS-----MIGFPFDNMRSRSFVIEPVVRTDAAQLAALHALTFRQHWSDDEFHALLVEDNVFGFIAREEGNPKAMAGGFIVARLVLDEAEILTIAVAPPAQRRGLGHALMDATLRHLHNVRASMLFLEVDELNAPALALYRRLGFKQVGKRPGYYETAAGRSTAFTMRRDLKA----CR------------MSKN---PDILRIAIAQLNPVLGDIKGNLAKAREARADAARQGADLILYTELFISGYPPEDLVLKRAFIEACEKAVQDFALDTADGGPGVIMGTPLKRETGLHNSVMVLDGGKVIAERFKVDLPNYGEFDEKRVFQPGPMPGPVNFRGVRIGIPICEDIWGDHDVCETLAESGAEIFCVPNGSPYYRGKVDVRYQVALRQVIETGLPLVFANQLGGQDDLIFDGASFAFNVDKNLAFQMSQFEEQLIVTTWRRGPNGWVCSEGPMSRIPEGEEANYRACMLGLRDYVNKNGFKDVVLGLSGGIDSAICAALAVDALGEERLRAVMMPYTYTSKDSLKDAEDCARMLGCRYDIVPIFEPVQGFLKALGPTFEGTKEGITEENLQSRARGTILMAISNKFGSMVVTTGNKSEMSVGYATLYGDMNGGYNPIKDLYKMQVYAMSEWRNGNVPAGGLGPSGEVIPVNIITKAPSAELRENQTDQDSLPPYPVLDDILECLVEHEMGVDEIVERGHDRTTVERIEHLLYIAEYKRRQSAPGVKVTKKNFGRDRRYPITNRFRDRG---MSMTDPLGDMLTRIRNATMRKKGKVSTPASKLRARVLDVLQAEGYIRGYSQVDFENGKSEIEIELKYFENVPVIREITRISKPGRRVYVSVKSIPQVANGLGISILSTPKGVMADHEAREQNVGGELLCRIFMSRPLVIAPSILASDFSKLGSEVTTVLQAGADWIHIDVMDGHFVPNITFGPDVVKSIRPLTDAIFDTHLMISPCDPYLEAFAKAGSDIITIHAEAGPHLHRSLQAIRALGKKAGVALNPATPESAIEYVLNDVDLILVMTVNPGFGGQKFIAETQEKIRKIKAMIGDRPIDLEVDGGVTAETAPLATAAGANALVAGSAVYRGGTEAA----YRANIDLIRNACLKG---MSQANPTREDFEALLAESFATNDLAEGYVVKGRIVAIEKDMAIIDAGLKVEGRVPLKEFGAKAKDGSLKPGDIVEVYVERIENALGEAVLSREKARREESWVRLEEKFNRGERVDGVIFNQVKGGFTVDLDGAVAFLPRSQVDIRPIRDVTPLMHNPQPFEILKMDKRRGNIVVSRRTVLEESRAEQRSEIVQNLEEGQVVEGVVKNITDYGAFVDLGGIDGLLHVTDMAWRRVNHPSEILSIGQTVKVQIIRINQETHRISLGMKQLESDPWDGIGAKYPIGKKITGTVTNITDYGAFVEIEPGIEGLIHVSEMSWTKKNVHPGKLLSTTQEVEVVVLEVDPVKRRISLGLKQTLDNPWTTFADKFPVGTIVEGEVKNKTEFGLFIGLDGDVDGMVHLSDLDWNRPGEQVIEEYNKGDVVKAQVLDVDVEKERISLGIKQLTGDKVGEAATSGELRKNAVVTVEVTAITDGGLEVRLVDHDLDTFIRRADLSRDRDEQRPERFTVGQKIDARVIAFDKKTRKLQVSIKALEIAEEKEAVAQYGSTDSGASLGDILGAALKKQEKN---MLHLQRLDALMDRYDVLLCDVWGVLHNGVDSFTSASEALSRARQAGLTVVLITNAPRPFAGVAQQIHSLGVPETSYDRIVTSGDVTRELIRNAPRHVFHLGPDRDESLYDGLDIELVEEREADVVVCTGFFDDETETPEDYSEMLTRFRSRNMPFICANPDIVVERGDRLIWCAGALARDYGQLGGRTLIAGKPHRPIYDAAVAAAEEVRDKSVDRSRVLAIGDGMLTDIKGADLFGIDALYISGGIHAGDYVMGGVHDLEKMLAFLEKHGSNPVATMPALA------------MEEPR-----PGKEPKRFKVSPLLGLDAWIDSTLYETRFRLAEFWEDTTIFFRRFHVTGWRRAIFEVLGEAFTWGTVGSVVMLTLAIPAFHETEKNWRTRDDFAVTFLDRYGNEIGQRGILHRSAVPIDELPDHVIKAVLATEDRRFFDHFGIDFLGLSRAMTENLRANSVVQGGSTITQQLAKNLFLTNERSIDRKIKEAFLAVWLESNLSKKEILQTYLDRAYMGGGTFGIAAASQFYFGKSVKDVNLAEAAMLAGLFKAPAKYAPHVNLPAARARANVVLSNLVQGGLMTEGQVVGARRNPATVVDRGRSDSPDYFLDWTFDEVRKVAQKMPTHTLVVRTTLDTGIQKAAEESAEFHLRKFGKEYNVAQAAIVVLENNGAVRAIVGGRDYGESQFNRATAALRQAGSSFKPYVYAAAMEKGLTPKTLVSGGAVSWGNWSPQNYSRQYLGKIDLTTALVKSINTVPVRLAKDYLTTAPIVALTKAFGVESPISSHKTMVLGTSGMTVLDQATGYNVFATGGIAGMRHPFTQILNQNGDVLWDWKKDGVKPHRVLSETAANNMNFMLAQVPEWGTGRRAALPMTRAAGKTGTTQSYRDAWFCGFTGNYTAAVWFGNDNFTPTKELTGGILPAMTWQRLMNYAHQNIDLKAIPGIDPTLPKQEKVAAAEEEAV-EEDTDALKAEERPRVLSNATAAAVREIGQALKDAPRLKPPVPPAKVSAL--MKTFIIQFFTWWNGQTLGTRFFTWRKGTKVGEDQFGNVYYEG--TFDSEGRKRRWVIYNGYAEASAIPAGWHGWIHHRVATPPSAENYQPHSWELNHQPNLTGSPAAYRPKGSIAH-GDN---RPTVTGDYDAWTPGSMTGSSRTTADLQPRLRKILYRSWHRGMREMDLILGQFADAHIDTLSDDELDQYEALMEALDRDLLKWVTGEADVPAEFDTPIFRKVVASRNNISS-------------------------------MTAPVFRFAPSPNGHLHLGHAYSALLNLRMARAMKGRLLLRMEDIDRERCTPELDQAMLEDLHWIGLDWELPVRRQSEHFAVYSEALDKLIGMDLVYPAFLSRGDIKREIEKSAGGKDNWPRDPDGTLLYPNADRQLSQRERDRRIGEGRLFSWRLNMDRAVELLRVPLSWTEFA-----PE-HTVLARPQDWGDIIIARRDMPTSYHLSVVIDDALQGITHVVRGRDLLHATSVHRLLQHLLGIEPPLYHHHTLILDRDGQKLAKSRKDTSLRDLREQGKTRQDIFDQVGL----------------MCGIVGIIGREQVAPLLVDALKRLEYRGYDSAGVATLE-------SGVLDRRRAEGKLVNLDNLLKENPLGGTIGLGHTRWATHGAPTVRNAHPHTTP-----------RLAVIHNGIIENFAELRAMLEKDGYVFETETDTETVAHLVTRAMDRGLDAIEAVRQTLPQLRGAFAIAIMFKGEDDLLVAARNGPPLAVGYGDGEMYLGSDAIALAPFTDSLSYLEDGDWAVLTRSGVTIYDETGVTVDRPVQKSAGTAFLVSKGNHRHFMEKEIHEQPEVISHTLAYYLDFSKGTIRAGSAPVDFAKIDRIAASACGTAYYAGLVGKYWFEHIARLPVDIDVASEFRYREMPLSKDSLALFVSQSGETADTLASLRYCKQQGLSIGTIVNVRESTMARESNTVFPTLAGPEVGVASTKAFTCQLSVLASLAVAAGKMRGTINEEEERQLVRELSEIPRYANQALRLEEQIETISRDLSQVKHVLYLGRGTSFPLALEGALKLKEISYIHAEGYAAGELKHGPIALIDESMPVIVIAPYDRWFEKTVSNMQEVAARGGKIILITDEKGAAASTLKTMHTIVLPNTPEIIAPIIYALPIQMLAYYTAVFMGTDVDQPRNLAKSVTVE-MASDGNTLIWRLMEAGDLASVSAVAAVAHPDFPEEDAVFVNRLALYGQGAHVLEN-GTSIVGYAITHPWKSFDVPALNTVLPALLGY-DTYYIHDIALLDAARGSGAAGRIVAILATHAAAAGFQTMSLVAVNGSSGFWQKHGFDIVN--RQVLQQKLRTYSDDACFMLRQLR--------------MAESVQGPFETENEEFQPAAGH--WNAALPVSEALRMVEAILFASAEPVTEKALTERLPSGIDVPALLGELQEIYSKRGINLRQVGGAWAFRTAADLAFLMNREAVQQKKLSRAALEVLAIIAYHQPVTRAELEDIRGVETSKGTLDVLMETGWIKFRGRRRTPGRPITYGTTASFLDHFGLQEVRDLPGIDELRGAGLLSARMPSNFSIPIPANDPDELTEDEDPLTDIDLEEL----------GLLTPRVEHD--MTTDQR-S---WLKLASVLLPILFSVPLVLGFFGSFHPALDAFAHFRLHLAVLMVVSAIPALFFGLWREGLMAIVLALTALATILVPSPAPSAEAGAAA----AVPASHPEYKLLQLNLRYDNRSPGEVIRLIARQAPDVMTLQEVSNDWRPRLKAIEARYPYTLYCPNRSHIGGVAILSRRPFALGTTPQCVG-SLIGLARIDFGGRSAIITALHLDWPWPYSQPRNVKMIVPYFERLQGPIIIAGDFNAASWSQTVRNIAAASKTQSVEGLRPSWFAIGMPDFVTQWIGLPLDHILTSDKIVNPRVETLPKAGSDHLPMLLRFSIGGVDN-AGPDQQTVMLHD--------------------MFEVGWSEILVIVIVLIVVVGPKDLPKMLRAFGKATTKFRATAGEFRRQFDEALKEAELDDVRNVINDAKSLDPRNDIRKVFDPVRTIGEEIRSSLKDATAIAKENVAVPDPEAPGATQLQPQVMEPLSAAAPEKVTTAEAGASKPKATVSK-------------KPAAPKKAGT---------------AEAVAEAKPAKP-PATRTAKPRT--KTGDTGIESMSFFRTALVVAAVSSLSF-ATLNQGWADDANTMTIKLKDGDVKIELLPDLAPKHVEQIKALAKEGAYDNVVFHRVIPGFMAQTGDVQYGNTEKSYNPQAAGTGGSTRPDLPAEFSKEPFVRGVVGMARAQNPNSANSQFFIMFKEYPSLNGEYTVVGKVTSGMDVVDKIKKGSEADNGAVENPDKMIKVTVGK---------------MNEPTKTPRISQAMVEAYDEYTHLTLDRRSFMDKLTKLAGSGAAAAIIAPMLAANSARAAIVAADDSRLRTKDIVFPGPSG-DIKGYRVNPADASGKLPAVIVIHENRGLNDHIRDVGRRLALEGFIALAPDLLSSAGGTPSDEEKAREMIGALDSKTAIAEGVATIEYLKKDKSTNGKVGAVGFCWGGGMVNDLAVNAPDLGAGVAYYGRQAKPEDVAKIKAPLLLQYAGLDTRINAGIDAYKKALEENDKTFEIFVYEGANHAFNNDTSEARYDKNAADLAWGRTVEFFKKNLSMSE----TPEMTVETSLEYGAESIKVLKGLDAVRKRPGMYIGDTDDGSGLHHMVYEVVDNAIDEALAGHATRVTVTLNPDGSCTVTDNGRGIPTDMHSEGVSAAEVIMTQLHAGGKFDQNSYKVSGGLHGVGVSVVNALSVWLRLKIRRQGKIHEMSFTHGVADAPLKITGDAGEETGTEVSFLPSTDTFTMVEFDFKTLEHRLRELAFLNSGVRILLADRRHADPVELELFYEGGLTAYVRYLDRAKKELVSEPIYIRGEKDDTTVEVAMWWNDSYHENVLCFTNNIPQRDGGTHLAGFRGALTRQMTGYAETSGQAKKAKVTLTGDDCREGLTAVLSVKVPDPKFSSQTKDKLVSSEVRAVVESLVNEALSTWLEEHPAEGKVLVEKVIQAASAREAARKARDIT-RKNSLSISSLPGKLADCQERDPAKSEIFIVEGDSAGGSAKGGRSRQNQAILPLRGKILNVERVKLDRMLSSDQIGTLILALGTSIGKDE---FNPDKLRYHKIIIMTDADVDGAHIRTLLLTFFFRQMPELIERGHIYIAQPPLYKVSRGKSSQYIKNETAFEEFLIESGLEEASLELSTGEVRAGVDLRSVINDALAVRHLLQGLNTRYDRAVVEQATIAGALNPEAISDTARAEALVTEVAGRLDIIAEETEKGWTGRLATPDDGVDGYIFERSLRGVKETVTLDMALLGSADARQLDRYSARLHDIYSKPPVLRRKEKSDSVAGPIALLEAVFAAGRKGLSLQRYKGLGEMNADQLWETTLDPNARSLLQVKVNDATDADSLFSRLMGDDVEPRREFIQDNALSVANLDVMRKLYGFLRRVAVDALGHFLTDDGWALASHVALSGMMALFPFLIFATALASFLGASAFTDTAVHVIFDTWPEVIATPIAHEVRNVLNVQRGGLLTISVIAAAYFASNGIEALRVALNRAYRVIDTRSIIYCRLQSLGYVVVATIGIMAISFLLVLAPLAVRIAERWIPEITLVSGTIGFWRYCIATAVLIIGLLVVHLWLPAGRRRFVDILPGIVLTLIAWTAGAAIFASYLETFASYVSTYAGLASIMAAIVFLYIIAAIFIIGAEINAAIIRYREG---KAAERLMSKRIAGPEIERLIQLLAKVPGLGPRSARRAALHLIKKKEALLIPLGSAMQEAADKVRVCSTCGNVDTSDPCMICTDSRRERATLIVVEDVSDLWALERAGSMNVRYHVLGGRLSPLDGIGPDDLNIATLVSRVTEGDIKEVILAVNATVEGQTTAHYITDQLAGLDVRITRLAHGVPVGGELDYLDEGTLAAALRARTSF------------------MTNDEPRKVLVLTGASRGIGHATVKRFSRAGWRVITCSRQSFDDNCPWPAGPEDHIKVDLADPADVDHAVSEIQQRLAAEGGKLNALVNNAGISPKNSTGDRLDSIETPMQTWMTVFQVNFFAPIMLARGLFKELEAAQGSVVNVTSIAGSRVHPFAGTAYATSKAALASLTREMASDFGPHGIRVNAIAPGEIDTAILSPGTDKLVEHLPLRRLGKTAEVAETIYFLCTESSSYVTGSEIHINGGQHV-MDDGNEPVRLREKAYASFTEHLLARDINPGQFVSQRQLVAMTGMPLGAIRELVPRLEAEGLVKTIPQRGIQIAHIDLNLIREAFQFRLFMEKEAIALFCQSASDELLARLRKEHEETLEEALQGKETAELELRAQTIDWNLHETIIDSLGNEIIARAYRINAIKMRLINQERFRITGRVVPVMREHLAVLTAIETRDPQAAMDAIAVHINNARNLALKL---MRVAIIDYGSGNLRSATKAFERAARESGINATIDLTDDAARVRDADRIVLPGVGAYADCRRGLHAVEGMVEAIHKVAVEKARPFLGICVGMQLMSSRGLEKTISTGFDWIKGDVRKMVPSDPALKIPQIGWNTIRLNRPHPLFDGIETGPQGLHAYFVHSYFLDAEIENDVVAVTDYGGTVTAAVANDNMAGTQFHPEKSQALGLALIANFLRWNP-MASLLLS-LNRGF-------LLFLTIVSLIVAQAQTASA-QETYTMDEIVNSGQRFFGSTSGGLATAVEKVFQSYGLPNGYVLGEEGSGALIGGLTYGEGSLYTKNAGDHRTFWQGPSIGWDFGGQGSRVMMLVYNLDDIQNLYGRFVGVAGSAYLFAGVGFNVLKRNQVLLVPIRTGVGARLGVNVGYLKLTQSPTWNPFMANTADAIIDRRRLRRKLTFWRAFTLLLIALALISFYFLSTGEDSFSSKSTPHIAKVRVEGTIFENEELLKRLKDVEESAAVKGVIITVDSPGGTTAGGEAIYEAVRKLAMKKPAVAQVGTLAASAGYMIASATDHIVARQSSIIGSIGVLFQYPDISELLTKLGVKVETIKSSPLKAEPNFFNPASDEAKAMIHRMIIDSYDWFVGLVQERRKFSHEQALALADGSVFTGRQAVANKLIDELGGEDKAIGWLATKGVDSKLPVVEWKPVQKSS-LGRLFSESALNMLAQYLGIPQESAG-ILRELTGERIFLDGLVSVWHVDGKPVGAQ--------MSCGARLIS------ISTVALGILASSMIV-PAVAQDAGAAK----GIKLS-GDQPIQIDADKLVVHDNEGTATFTGNVTVVQGATLLKAGSMIVYYVKNDKKETAGA------APTANAPKDGGLAAPGPAAQDIDHLEVNDKVYVKSEDQVATGDHGTFDMKTEVLVLTGNKVVLTQGDNVAIGCKLTAQLKTGEAQLESCKSGQTGRVSIVVAPKNA-----------PKN--------MVSDETTLSDDAPHLLIVDDDTRIRNLLSQYLTGSGFRITVAANADEARRKLAGIDYDLLILDVMMPGESGVALTQSLRQEKNVPILMLTALSETDSRIAGLEAGADDYLPKPFDPRELILRINNILRRGAPAAQAKIEQIVFGPYTFVIAKRELKRSGEMIRLTDREQDIMAIFAARAGETVPRHELTGQEGDVGERTIDVQINRLRRKIENDPANPVWLQTVRGVGYKLSVEMIGLVLVTHGRLAEEFHHAVEHVVGQQEYLETVSIGADDDMEQRRRDIVDAVARADNGSGVIILTDMFGGTPSNLAISVMESGRIEVIAGVNLPMLIKLSSVRVTNDMAASLREGQDAGRKYINVASQVLTAK-----MAKITFVTFDGA-RIEANAENGSTVMENAIRNAVPGIDAECGGACACATCHVYVDEDWTSVAGDPEAMEEDMLDFAYDVRPNSRLSCQIRVSDDLDGLIVHVPERQAMTLKARVIPCLDVKDGRVVKGVNFVDLIDAGDPVEAAKAYDAAGADELCFLDITASSDNRETIFDVVARTAEQCFMPLTVGGGVRTVADIRKLLLAGADKVSINTAAVNNPEFIAEAADKFGNQCIVVAIDAKKVSATGETDRWEIFTHGGRHATGIDAIEFARRVVDLGAGEILLTSMDRDGSKIGYDIPVTRAIADAVRVPVIASGGVGNLDHMVEGIIEGHASAVLAASIFHFGTYTIAQAKAHMAAAGIPMRLDPVR-------MKFLDQAKVYIRSGDGGAGSVSFRREKFLEFGGPDGGDGGRGGDAWAEAVDGLNTLIDYRYQQHFRAKTGMHGMGRNMTGGKGADVVLKVPVGTQIFEEDNETLICDFTVVGQRYRLAKGGNGGFGNLHFKTSTNQAPRRANPGLEGEERNLWLRLKLIADAGLLGKPNAGKSTFLASVTAAKPKIADYPFTTLHPNLGVARVDAREFVIADIPGLIEGAHEGVGIGDRFLGHVERTRVLLHLVSAQEENVAEAYKTVRAELEAYGHGLADKLEIVALSQIDTLDAASRKKKLAALKKAAGREPMLLSAVSREGVEQVLRALSAIILQSRESEAPVEVDTRFKY--MYLGLDLGTSGVKALLIDEKQSVIASGTGILDISRPQSGWSEQDPSHWIKATEGAVAAVRATHGKELAAVKGIGLSGQMHGATLLDAGDQVLRPCILWNDTRSHKEAAEFDSNPIFRALSGNIVFPGFTAPKIAWVKNNEPDVFAKLSRVLLPKDYLRLWLTGEHVSEMSDAAGTSWLDVGKRAWSPELLAATGLDASFMPSLVEGTEVSGKLKADLAARWGMGTSVVVAGGAGDNAASACGMGTVAEGHAFVSLGTSGVLFAANSAYLPNPQSAVHTFCHALPNTWHQMGVILSATDALNWYAGIASRQPAELTGELGDQLRAPSGVTFLPYLSGERTPLNDSAIRGSFHGLEHASDRAVLTQAVLEGVAFAFRDCLHALAAAGTKLERVTAVGGGSRSTYWLKAIATALNIPVDVPADGDFGAAFGAARLGLIAAENGDPRAICAAPSTDYTIEPETALTDAFDAAYNRYHNLYPALKGVQS------------MSLFAVAPPASGRSIADCDKLQTAKAEVLSDTVDPIPDVDKNDKADACSPQNKAVSQQPVKVVPINIV-ATGSANILKKAIKGDILPQGEGSLHENLAFKYRIYRPEAPNGDTMVLLHGSGQDETSLVSFGSKIAPNALLLAVRGRVVQDGSNRWYRRLTPVSFDQKDIRSEAKAFAEFLKQVTREYKIDSNRTTFLGYSNGANLVNAVMMLYPDLVKQAVLLRSMPVLTGISEANLANARVLTVSGASDQLYAPYAPALEDLLRSHGARVEARSIKSDHGLGKDDVKVVSEWLS------GATAELKKN--------------MTASD-PVLEDR----LAGIVLNSIRHPVIMLDDAGHISYANADAEAFFRSSASILSRNTLDMLLPFGSPLLALVKQVRENPSPVNEYRVDVSSPRLGQDRIVDLYVTPVSESPGSIVILFKERSMAEKLDRQMTHRGAARSVTGLASMLAHEIKNPLSGIRGAAQLLETGLNDDDRALTRLITDETDRIVSLVDRMEVFSDERPIEREAVNIHVVLDHVKAIAKNGFASHIKFHEDYDPSLPYVFANRDQLVQIFLNLVKNAAESIGQNEPGEITLSTAFRPGIRLSVPGMRTRVSLPLEFTVQDTGPGVAPDIMPHLFDPFITTKPNGSGLGLALVAKIVGDHGGVIECDSVPRKTTFRILMPAWQS-GPLDDEHADEKPYSKKAGSMQRLVLAFINSMRALRHLAKHEKAVQQELILFLLSIPLAAVVAPTVLSFLLLTGSVLFLIMIEVLNTGIEAACDAVSLDFHREIQIAKDCGSLAVLISIILVATVWFYTVWTVVFS----MGSLKIENIRKSFGHVDVLKGIDLEVKDGEFVIFVGPSGCGKSTLLRVIAGLEDATSGDILIDGKKVNNTPPAKRGIAMVFQTYALYPHLSVRDNMGLGLKQAGKPADEIKERTGIASSMLSLDEYLERRPAELSGGQRQRVAIGRAIVREPQLFLFDEPLSNLDAALRVNTRLEIARLHRQLKATMIYVTHDQVEAMTLADKIVVLNKGKIEQVGSPMELYNAPQTIFVAGFIGSPQMNLIDAAKL-----GQSGAKTIGIRPEHIQVSKTSGEWKGSVIHVEHLGADTIIYLQTD-FGPLTVRLFGEHNYDVDGVLYATPDAGRTYRFDNDGQVIKG---MREVGHFIGGKHVAGTSGRTTEIFQPMDGTVQGTVALATPAEVRAAVENAKAAQPAWAATNPQRRIRVMRKFLELVEAEYDSLAELLAREHGKTIPDAKGDIQRGLEVVEVCLGAAHMLKGEFSDNAGTGIDVYSMRQPLGVVAGITPFNFPAMIPLWKAGPAIVSGNAFILKPSERDPGVPMRLAELFIEAGLPAGIFNVVNGDKSAVDAILDDPDIKAVGFVGSTPIAQYIYSRATANGKRAQCFGGAKNHMIIMPDADMDQTVDALIGAGYGSAGERCMAISVAVPVGKDTADRLVERLIPRVESLKVGPSTDMSADYGPVVTRQALERIKGYVDLGIQEGAKLVVDGRGFKMQGYENGYYMGGCLFDHVTPDMRIYKEEIFGPVLGIVRAETYEDAIRLPNEHEYGNGVAIFTRDGDAARDFASRVQVGMVGVNVPIPVPIAYYTFGGWKGSGFGDLNQHGPDAFRFYTKTKTVTSRWPSGVKDGAEFVIPTMK----------------------MKPDHFEVTPACTAVHHLLARVGDKWTVMVVKYLGNGSMRFNELKRTIDGISQKMLTSTLRGLERDGFVTRTVFPTIPPRVDYELTDLGRDLLVPVKALGDWAIKNEQRVREARARFDAARAGKE----HHSPFADAAEMANNYDVIIIGSGPGGYVTAIRSAQLGFKTAIVEREHLGGICLNWGCIPTKALLRSAEILHYGQHAKDYGLTIEGKISADVKAVVQRSRGVSARLNGGVGFLMKKNKVDVIWGEAKLSK------PGEIVVSKTSKKPMEPQNPIPKSALGEGTYTAKHIIVATGARPRALPGIEPDGKLIWTYFEAMVPPEMPKSLVVMGSGAIGIEFASFYHTMGAEVTVVELLPTVMPVEDAEISAFARKQFEKQGMKIITDAKVTKVEKAANSITAHVETKDGKVEKITAERLISAVGVQGNIENLGLEALGVKTDRGCIVTDGYSKTNVPGIYAIGDVAGPPMLAHKAEHEGVICVEKIANVPGVHALEKDKIPGCTYCNPQVASVGLTEAKAKENGREIRVGRFQFAANGKAIALGEDQGLIKTIFDKKTGQLIGAHMVGAEVTELIQGFVVAMNLETTEEELMHTVFPHPTLSEMMKESVLDAYGRVLNAMAKTKFRPLHDRVVVRRVESEAKTAGGIIIPDTAKEKPQEGEVIAVGTGARDEAGKLVPLDVKAGDLILFGKWSGTEVKIGGEDLLIMKESDILGILGMKLKDYVWPVIGLAAVGISVWLLYKELRSISLDDVIHSLYAIPTHRWILAGLSTLAAYAALAGYDRIALLHLRRKISWLFIALCSFTTYALSHNIGASVLSGAVVRYRAYSSQGMSTPEIALLIAFCSFTFMLGILTLSAIVLLVEPGLLRRFNSELPLTLSYILGFGSLMIVLLYIFGSWLHFRPLRVRRFTLEYPRLSVVAQQLIVGPLELVGAAAIIYFTLPVAGNPGFLVILGIFLVSFSAALISHAPGGLGVLELVFLTGLPDMDQADVLAALIIFRLFYLLIPFALSLLVVLYFERSQLLLRWYKKDGET------MIQSVLYFAFGFLSAVLLALLVAPPIWRRATLLTRKRVEAETPLTLNEIQAQRDGLRAEHAIAERKLELTLENVSEKAARQLAELSEKDRLARNLAGDLAARDATVAELRSALADRDKELEKAAKVIVGHERSLEQRSSELELLHRRMASLAMNADSLQIEAAAQSTRIENLADDLREARQDKRDSDERKRKAETDLKAIQHALEQEVKRSAELEKRANALLRQLSDSEARLSRREKEIERINERLRKVLADGRRQ-GSSVLEPPRGKEALVQTQETLRLREEMNTLASQVVAMVARLEGPSSAVDELLAKAGSETSPVYDENGEVIVSLADRIRALQVAA----SDAGKAAEPR------MKGSKMTIAQEVKSLLTKLEVNEAAYTGGTMPSFSPVTGEKIADIATHSSDDTAKTIEKADAAFRQWRLVPAPKRGELVRLFGEELRARKSELGRLVSIEAGKIPSEGLGEVQEMIDICDFAVGLSRQLYGLTIATERPGHRMMETWHPLGVVGVISAFNFPVAVWSWNTALALVCGNAVVWKPSEKTPLTALACQAIFDRAVARFGDVPAGLAQTLMGDRTIGEALVDHPKVALVSATGSTRMGREVGPRLAKRFARSILELGGNNAGIVCPSADLDMALRAIAFGAMGTAGQRCTTLRRLFVHESVYDTLVPRLKKAYESVTVGSPLDTAALVGPLIDKVAFDNMQKSLHEAAEHGGKVTGGARVDTGHAEAYYVRPALVEMPKQDGPVLEETFAPILYVMKYSDFDQAIADHNAVGAGLSSSIFTLNLQEAERFLSPDGSDCGIANVNIGTSGAEIGGAFGGEKETGGGRESGSDAWKAYMRRATNTVNYSSALPLAQGVSFDIE----------MRKAKGAG---------------TKG-----SAPKSTPHRQSATKPRPQFAKPV------RAADSEAQAKPTPRSE-PTPGRLLTRRSGARPHEQTGLILETLPSEDYALLDSGNGLKLEQYGPYRIIRPEGQAIWLPAWDRSEWDRADAVFTGNTDEEGVGRWHFPKVPLGETWPLAFDGLPFFGRFTSFRHVGVFPEQGTHWSHMDSLIRGAKRPVKVLNLFGYTGVASLVAARAGAEVTHVDASKKAIVWARENQEMAGLSGKPIRWICEDAMKFVAREERRGSSYDIILLDPPAYGRGPNGEVWQLFDNLPDMVDLCRAILNPNPLAVVLTAYSIRASFFAIHELMRDAFTGLGGEVQSGELILRERSSGRALSTSLFSRWGAKMKLTRRLIPDIDILQTFECAARHGSFTQAARELNLTQSAVSRQMSELEGQIGVLLFERVRQRVVLSEAGQKFLPEVRRLLGLTEETMLRAMAASQSASSLSIATLPTFGSRWLMPRLPDFLGRHPEMALSVASRSQPFDFEEEPFDLAIHYGQPVWAHATCTYLCSELIVPVGSPELCASNPATTPPELANAAPLLHLSTRPKAWASWFETVGADVASPYKGHRFEHFSMVIEAALAGLGFALLPRYLIEHELASGRLKVVFDQPLQTENSYYLVVPDHKKENPLAQSFFTWIADQVT-----------------MDIIFKFLAVIAIAAVTIVLLVGLRNMMKGGDANFSNKMMQLRIFLQLIAIILIVGALYFHRAAG-----------------------MTDEKKSTGSVTPLRPTRPCPECKRPSQREHYPFCSARCRNIDLNRWLTGAYVLPAVESADDSDQDADN----------------MAPRIAVLGCGYWGSNHIRTLKSLGALYAVSDENSDRAAGFAAEQGVEHIEVDDLFTHPDVDAIVMALPPQFHAETAIRAVTNGKDVLVEKPIALTVPDAERTVKAAHDNGRIFMVGHVLRFHPAFEKLLELVKAGDLGKVRYIHSHRLGLGKFHTENDALWDLAPHDLSMILAITGTEPLEVRGEGAAVLDHLSDFAHLHLSFPNNVRGHLFASRLNPYRERRLAVVGTKAMAVFDDMETWDRKLAVYKHSIWQDNGQWASTTEEPTYIKIAQGMPLTRELQNFIESIQTRVQPKTDGEEAIRVLRILTAGTVTHNK---------MHLDIIDLRTFYASTLGHLTERTITMALSPIWPKLPGERLVGLGYAVPYLDRFRGDTERTFAFMPAGQGAVSWPPAEPSATALIFEEDLPLPDSAVDRVLMVHALEHAEDPRETLKEMWRVLAPNGRLVIVVPNRRGLWARFEHTPFGSGRPYSRGQLTRLLREANFTPGPWGEALFFPPSSRRWMMRPSGLFERMGRRFWPMFSGVLIVEAQKRIYQGLPVAKRSSRRVFVPVLSPQGTAS-ASSR-------------------------------------------------------------------MARRIAERVWAPGALIPGEEVLAVEFDCARATVNRALQELARGGVLVRKRKAGTRVALHPMREARFVIPIVRQEIENKGGIYQFRLLSNQVEKAPQSIAERLGLAKGKAMLHVRSLHLSNSKPYQYEDRWVNLDAVPSIRHETFETISPNEWLVTHSPFSQAEIVFHAALADPEEAAILDVRERDSVFIIERLTYLPQKPITYVRMIHPG-SHRMVTQL--------------MPEVIFNGPAGRLEGRYQPSTEKNAPIAIVLHPHPQFGGTMNNKIVYDLFYMFQQRGFTTLRFNFRGIGRSQGEFDHGSGELSDAAGALDWVQSLHPDSKTCWVAGYSFGAWIGMQLLMRRPEIEGFISVAPQPNTYDFSFLAPCPSSGLILHGDQDKVAPPKDVQGLVDKLKTQKGITITQKTMVGANHFFSGMTDEVIAECSDYLDRRLNGELVEAKPKRLR------MTQWLTVIGIGEDGYDGLGQNARQVLAGARTVFGGKRHLELLPAEVVARRVTWPTPFSDAYAMLLALRGEPVAVLASGDPMHFGMGAALTRYVEAEEMRILPAPSAFSLAAAAMGWPIQDIQLLSVHGRPVETLFKAFVPGARLLILSNDGGTPEQVARMLSQSGFQSARLTVLEHMGSDAERHIEGMASAWGHPRCADLNVLAVDCGFTASSARAYSTLAGLPDDAFEHDGQLTKRDIRAVTLAHLAPLPGQLLWDVGAGCGSIGIEWMRAHPACRTLAIEADSKRQELILRNSHALGVPDLKLVRGKAPAALAGLMAPDAIFIGGGVTDDGVMQTCWECLKPGGRLVANAVTIQSEMTLIRWRDTYGGDLTKLGVSHAQPLGSFDTWRAVLPVTVYSVNKPD-MEESRGRIKITNRISIDEDDLEESFIRSAGPGGQNVNKVSTAVQLRFLGARAGLPDEVFARLVKLAGQRATKEGDILIEANRFRTQERNRQDARERMVALIAKAAEPPPPPRKKTRPTKGSIERRLKAKSGRSDVKRMRGKVKGDMASKKKTDVKAATKSKPPKAAKEKKAEIGL--FEELLFARAPEEDLADYDAGAVSTSAGFAEAALARFTPGQSVVDVSENNLVYRDGRPVTVISLINDNMPFLLDSVLGEISERVSTVHLVLHPVMDIDAKSN--KVIGAA--GPEKPGKDVSRVSVMQIHVSALDAASREIMTAALTLVLKHVREAVRDWKPMLSRLSDAIVAYKTGKLPMKKAAAEEAVAFLEWLRDDNFTFLGVREYDYLGSEGAGELERSGRPGLGILADPDVRVLKRGSQGVTTTPQIIAFLTGPEPLIVTKSNTKSLVHRRGYMDYIGVKTFDSDGNVTGEIRFVGLFTSGAYTRSVLKIPYLRSKTEAVINRLGFNREDHSGKALLNVLESYPRDDLFQIDVKTLTRYAEAILALGERPRVRVLPRVDPFGRFASIIVFVPRERYDSVVREKMGSYFASTYEGHVSAYYPAFPEGNLARVHFIIGHAEDEFPKVKRETLEEAVRAIVRTWDDAVAEVGDR------TGLSEFTALAAAFPDSYRENFSADEALVDASRIAGLSRNNPLFVDFYRHRTDGPNAASLKIYHYGSAVALSQRVPLLENMGFRVISEQTFALP----AGDGSPLYVHDMELVSASGSPIDLSDNGNLYEDVFRSIWGGASDNDGYNALALTGQLTARQIIILRAYGRYLQQAGIPYSQ---DFIAATLNRYPLIAKQLFELFEQGLNPAK--AGAAQSQRKQTVIKTAIEEALQGVPSLDDDRILRRFLNLIESTLRTNAFAPEEDGSERVTLAFKLDPRLLDDLPEPRPYREIFVYGPEVEGVHLRFGPVARGGLRWSDRAQDYRTEVLGLVKAQQVKNAVIVPVGAKG--------------GFYPKHLPTGG-----DRNAIFEAGRAAYITFISTLLSVTDNLDGEKVVPPVNIIRLDGDDPYFVVAADKGTATFSDTANAISQAHDFWLDDAFASGGSAGYDHKKMGITARGAWEAVKRHFREIGTDIQKTPFTVVGVGDMSGDVFGNGMLLSPQTKLVAAFDHRDIFIDPEPDVKASLKERQRLFNLPRSSWQDYDKTKISAGGGVFSRSQKSITLSKEAAKVIGL--AKTTATPFEIMTAILKAPVDLLWFGGIGTYVRSTLESDAEAGDRSNDAIRITGSDVRAKVIGEGANLGVTQKARIEFGLLGGRCNSDAIDNSAGVNTSDVEVNIKIALSQAMRTGRLQRAKRDTILESMTDEVSDLVLRNNYLQTLALSLAQRRGVADLAYQARFISDLETKKLLNRKVETLPDDKALAERQVDGIPLTRAELGVLLAYAKIVLSDQLLASNLPDDPYLEQELMDYFPSQMKAGFSEELRTHRLRREIIATQLANDVINRGGPTFVSRLSDLTGQSGPEIVRAYVIVRDGFELDGVFAGIDALDNAISGDTQNGFYAKVAQMLNWTTAWVLRNGTATVGLRAAIDEIRQARNALQPRLDKLLPMSMTGLIREDTASNAALGASPALAAQLARLEIAPIIPDIAAVAQQANIDIASAGKAYFDISEAFRIGRIEDAARGVSVADYYDGLALARATDLIGQARRGIAVAALSKYGK-QTDPAKDWLVSRGARVDEVRQRMANIVETGDLTVSRLAVAAGLLSDLS--MAARIYRPAKTAMQSGKAKSDTWTLDFEPEQPRKVEPLMGYTSSRDMKSQIRLTFETKEGAIAYAEKNGIAYTVQEPKETRRRVVTYSENFRFDRKQPWTHMAGTIKTVGIIGAGQMGSGIAHVCALAGYNVLMHDQSAEQIEKGIATVNGNMARQVSHGKLEDAQRAKAMTHIRPALRIEDLAGVDLAIEAATEDETIKRKIFSQLCPNLNPEAILATNTSSISITRLAATTDRPERFIGIHFMNPVPVMKLVELIRGIATENVTFETAREFVSSLDKTITVAEDFPAFIVNRILLPMINEAIYVLYEGVGSVEAIDTAMKLGANHPMGPLQLADFIGLDTCLSIMQVLYDGLADSKYRPCPLLVKYVEAGWLGRKAGRGFYDYRGEHPVPTRMNWLKSLLVVG---VLAIVP--AQAQAASNLEQVKAAGVLKIGTEGTYAPFTYHDASGKLVGFDVEIGEAIAKNLGVKPEFLEGKWDGLIAGLDANRYDTVINQVGITEARKQKYDFSEPYIASKAVLIVKGDNDEVKSFADLKGKKSAQSLSSNFGKIAQEAGAELVGTDGFDQSIQLVLNGRADATINDSLSFLDFKKHKPDANVKIAAEQENADYSGIIIRKGEPDLLAAINKALETIKADGTYQKIADKYFGQDVSK----MQRMGMMIGLNAGKVAEYKRLHAAVWPEILELISSCNIRNYTIFLREPENVLFGTWEYHGTDFKADMEKMAANPKNKEWWAVCMPCQVPLATRAEGEWWAMMEEVFHLD-----MSKNKARAPHLLIVEARFYDDLADALLNGAKLALDEANTTYDVVTVPGALEVPAVISFALDAAVEGGKEYDGYVALGTVIRGETYHFDIVANESCRALMDLAVNESIAIGNGILTVENEDQAWVRARREDGDKGGFAARAALTMIALKEKLGAMKIVMAIIKPFKLDEVREALTAVGIQGLTVTEVKGYGRQKGHTEIYRGAEYAVSFLPKLKIEVAVASELVDTAVEAITTAAKTGQIGDGKIFVLSIEQAVRIRTGETDTDAL------------MKHTFNQKPDYEKELRNAGVRITRPRRIILDILTSTEDHPDAMKIFHRAIEIDDSISLSTVYRTMNLLEEMGAIHRHAFNGGPSRFEQAHGEHHDHLIDVDTGAVIEFKSDKIEKLQEEIAKSLGYDIIHHRLELYGRKIDRSS---MNHHMLSGAFVPLKLADRSLAYKTLAVLIGTVFLAVSSWIEVPMYPVPVTMQTFVVTMVGALYGWRLGGLTVMAWLAEALIGFPVLAGGAGGLAHFAGPTAGYLFAFPVVAAFVGFLAERGLT-RNPFVSFAVMLLGNVLCLALGASWLANMIGFEKAWTFGVAPFIIGGVLKSALAAATIELLRRSGIMVRLS-MNTVEDIPGASQIPEIAKSIRERLGHKSIVLVGLMGAGKSTVGKKLATLIELPFFDADHEIEKVSTMTIPELFEAYGEAEFRDLERRVIARMLEDGPIVLATGGGAYMNDQTRRTIASEGVSLWLKAELDVLMGRVVRKQNRPLLKNDNPRGVMERLMTERHPVYALADLTINSREEKKEVIAFEAMQAIAHHLDGIA----V-TNEETGS---MNEQKPLRLRKRAEFLAVRAGEKRRGAYFLIEVNGRADQDGP-------PRVGFTVTKKNGNAVVRNRIKRRLREAVRVNVADDMHPGTDYVIVARRDALNAPFADLTRELSKRVSKKTQTRRSSKP-------MAQTGTVKFFNSEKGFGFIKPDDGAADIFVHISAVQASGLTGLADNQKVSFDTEPDRRGKGPKAVNISVTEMQGQDMQFFDNDGLKIAYLDEGDGEPLLLIHGFASSAFYNWVQPGWVPTLTAAGYRTIAIDNRGHGQSDKPHDMSVYTPTLMAGDAAALLDHLGIAQAHVMGYSMGARISAFLALQHPKRVHDLVFGGLGIGMVEGAGDWSPIAEALLADDAESISHPRGKMFRMFADKTKSDKIALAACVITSKEEISAENMARITQPALVAVGTKDDIGGSPHRLAELMPRGKAIDIPNRDHMLAVGDKVFKQAVLEFLKRHPL---------MTGSEQNALDRRFMAAAIRLSRTHQGLTGTNPSVATLIVR-GG---VIVGRGVTAIGGRPHAETQALMEAGEKAKGATAYVTLEPCAHHGRTPPCADALVTAGVTRVVSAANDPDDRVSGKGYAILHDGGIDVEAHVLGAEAADNLAGYLIRSAKKRPEVTLKLALSSDGYIGREGEGQVSITGSVSRSQVHMMRAESDAILVGIGTVLADDPQLTCRLPGLENRSPIRVVLDSRLRLPMNSALVNSASTVPVWVAASAAAEPARRQALVAAGCRILATETDCGATALPELLDDLAAQGITTVMVEGGATVASSFLQEGLVDRIALFQGSVAIGSSSGVAVPELHSHIAREFHLLREYRFGDDRYSEYVRPL------------------MGIKSVLLTATLA---LGLASS-AWAQE--RVVNVYNWSDYIDPSILEEFTKETGIKVVYDTFDSNEILETKLLAGGSGYDVVVPTASFLQRQIAAGVFQKLDKSKLPNLSNTWDVINQRIATYDPDNEYAVDYMWGTTGLGYNVAKMKEITGSDEKPTWDVLFKPEVAAKFKDCGIHILDSASDVLPSVLAYLGLNPDSKDPDELAKAGALLETIRPYVRKFHSSEYINALANGDICLALGYSGDILQAKGRAEEAKAGVNVDYAIPAQGAQMWFDVMAIPADAPHVAEAHEFINYILKPEVAAKATDFVSYANGNKASQASIDKAILNNPAVYPTPEVLAKLFTVTPYDTKTQRVVTRLWTKVVTGQ-MNMQGNFEDLPKQKLTIIGAPIEEGAGRLGALMGPAALRTAGLIRTLEELGHPIEDRGDLCLPKDLPVVPPVAGLAHHIEKIAAWSRMLSNETYDAMKNGSFPVILGGDHSLSMGSVSGVARYCKEHNRELFVLWLDAHSDFNTPATSPSGNMHGMSAALLCGEPGLEGVFGDEPRGFVKPENLHLFGIRSIDSTERVLLRERGIDVIDMRKIDEFGISRLMRGIIDQVQKANGVLHVSFDVDFIDPSLAPGVGTTVPGGATYREAHLVMEMLHDSGVVGSLDVVELNPFLDERGKSALILVDLVASIFGRQIFDKPTQADQSAAALNV----------MNNTQRKSGASQHSTPSDTALRTLYLEALQLVERLHRRLLDVVKDEFDRNGRSDINAIQALLLFNIGNAELTAGELRSRGYYLGSNVSYNLKKLVELGFISHERSRTDRRSVRVSLTEKGTEVAELVAGLYERHVASIEHVGGIKAEEFQSMNKALQRLDRFWNDSIAYRMMSFFRQSRRPDLGTNIRVSFEFFPPKTEVMEERLWETVTRLAPLNPEFVSVTYGAGGSTRERTARTIKRILTETDVNAAAHLTCVDATKEEVDTVVREFAAMGVKRFVALRGDPASGVGAHYKPTPGGYENGADLVAGLKAFADFDISVSAYPEKHPESPDFATDIDMLKRKVDNGATRAITQFFFENDLYERYVEKVRRAGIYIPILPGILPIHNFTQVTNFCSKAGTHIPGWLTERFDGLENDPQTHALVASAVAAEQVLDLVERGIQDFHFYTMNRADLAFAICHLIGIRP-------GGT-ALEANLAGAAAMSRTFLFVLDSFGIGGAPDAKTFGDEGSNTFGHIAEACAAGRGDKTGLRSGPLKLPNMASLGLFHAANLASNVPLQPQM---PEPVGLWGAAEEVSNGKDTPSGHWEIAGVPVTFNWGYFPQTIPTFPDSLVADAARRAKLPGILGNKHASGTDVIDEYGEEHIRTGKPIFYTSTDSVIQIAAHETHFGLDRLYELCGIVRELVDPLNIGRVIARPFVGETRETFTRTGNRRDYSVPPPEPTLLDRLTQAGRSVIAIGKIGDIYAHQGISQVRKANGNMALFDETLIAMDDAQEGDLVFTNFVDFDMLYGHRRDVPGYAAALEAFDRRLPEAIAKLRQGDLMVLTADHGCDPSYKGTDH----------------------------TRERVPVL----------CFG-----PDLP-----------------------------------------------------------------------------------------------------------------------------------------H---GSFGIRPT------------FAD---------IGETVAAHLG-----------------------------------------------IEAG-----------------PHGKSFLKGTGVRTPDA------------------------MLGRVDAEQPIADLMADVGRKARAASQPLSIASPNKKAAALLAMADAVDQRKAEILRANEQDMANGEKTGMAASLLDRLKLDEQRIASISESIRTIAGLGDPVGEIIAEWDRPNGLHIERVRTPLGVIGVIYESRPNVTADAAALCLKSGNAVILRGGSDSAHSSKAIHEALLDGLEAAGLPKDAIQLVPTTDRAAVGEMLKGLDGNLDVIIPRGGKSLVARVQSEARVPVFAHLEGLCHLYIDASADLAMARKIAVNAKMRRTGVCGSAETLLVDRLIAPSHLVPLLEDLRAAGCEIRGDEGVVELFAGAKPATDEDWSTEYLDAIISVKLVNGVDGAIQHINQYSSHHTEAVIAEDVLVVERFFNEIDSAILLHNASTQFADGGEFGMGAEIGIATGKMHARGPVGVEQLTSFKYRVRGNGQTRPMAKKVAGQLKLQVSAGSATPSPPIGPALGQRGINIMEFCKAFNAATQELEKGSPIPVVITYYQDKSFTFAMKTPPVSYFLKKAANLKSGSKEPGKVVAGKISRDKVREIAEAKMKDLNAADVEAAMRMVEGSARSMGLEVVG-------MAAQTISEGSIALVQNREARNRQYIRIWLYLALIVLATIVIVGGATRMTGSGLSITEWKPIHGIIPPIGQVQWMEEFDKYRQIPQYEKLNKGMSLAEFKQIFWWEWAHRFLARGVGFLVALPLAFFWLTGRLERRLKPRMVGILALGGLQGAVGWWMVASGLVDRVDVSQYRLATHLTLACIIFAAVMYVARGLAVYSEAPANRQIQRFAGWFVLLVFIQIYLGALVAGLDAGLSYNTWPLMDGAVIPGDLFPIQPIWHNFFENPKTVQFVHRFFAYFVFAVAIWQAIATMRQAPGTTHARRAVLLAVLVALQAAIGITTLLMQVPISLGLLHQGFAILILGFAVAHWRATKGAYPLETEVVAGR------------------------------------------------MAIEFALIALPFFLLIFAIIEVSLSFTAQQVMSNAADDIARQLRTGEITAAAISAPNRLKQTICGRL-FMPAAGCPDLFVDLQTYTTFANVPRTLPLTTAGDVDVSQL-----RVTPGGASTINQLRIFYRWPILTDLMKHRIESVDGQGKMMLFSTATWRNEPYL--MSQEVVTRRITAPEIRGRKGGEPIVALTSYHANTARIVDNHADILLVGDSLGMVLYAMDSTLGVSLDLMITHGRAVVRGSRRAMVVVDMPFGSYEESPAVAFRNAARVMQETGCGAVKLEGGIRMADTIRFLTERGIPVMAHIGLTPQSINTLGGFKTQGRDTALWPLIKADAQAVSEAGAFSVVLEGIVEPLAAEISRQSVIPTIGIGGSRECDGQILVLEDMLGYNPKPPKFVKVYGAVGTAIDDAVKAYAEDVRSRAFPGDENIYPLK-------MEADFVIVGSGSAGSAMAYRLSEDGKYSVIVIEFGGTDIGPFIQMPAALSFPMNMSTYDWGFISEPEPHLGGRTLVTPRGKVIGGSSSINGMVYVRGHAHDFDHWAESGATGWSYADVLPYFKRMENSH------GGESGWRGTDGPLNVQRGRRDNPLFHAFVDAGQQAGFEVTDDYNGSKQEGFGAMEQTIHKGRRWSTANAYLRPALRRKNVSLVNGFARRVIIENQRTIGIEITRRGKTEIIRARREVIIAASSINSPKLLMLSGIGPAAHLKDNGIKVVADRPGVGQNLQDHMEVYIQQESLKPITLNSKLGLFSKAMIGAEWLFFKSGHGATNHFESAAFVRSKPGVDYPDIQYHFLPAAIRYDGKAAAKGHGFQAHVGPMRSKSRGSVTLRSSDPFEKPKILFNYMSHADDWTDFRHAVRLTREIFGQAALDPYRGREISPGAQVQSDEEIDDFLREHAESAFHPCGTCKMGATTDPMAVVDPQCRVIGVEGLRVADSSIFPRVTNGNLNGPSIMTGEKASDHILGRDPLPRSNQEPWINPRWQV-SDRMGQLQAGIIPVTAFQQNCTILFDSDDKHGVLIDPGGDNDQVLNAIKSNGIVIEAIWITHGHIDHAAGAMDMKDALDVEIIGPHEADRFLLDSIEAKAMQYGITG-KVRNCAPDRWLNDGDQVSFGSHIFEVLHCPGHAPGHVVYFNRAAKFAHVGDVLFNGSVGRTDLPGGDHATLINSIKTKLLPLGDDIGFICGHGPGGRFGEERRTNPFLTPE--MTSQIIPVDPFDCIVFGGTGDLAERKLIPALYQRQRDGQLSEPTRIIGASRSPMSTADYRTFAEAAIKEHVKAAEIDGKQLDKFLERLSYVAVDATSDNGWEDLKAEVGNDSKRIRAFYLAVSPSLFGDITSRLKSYKLVHDHTRIIVEKPIGRDLESAIALNDTIGHVFHEQQIFRIDHYLGKETVQNLMALRFANALYEPLWNSAHIDHVQITVAEAVGLENRAGYYDKAGALRDMVQNHMLQLLCLVAMEPPSSMDADAVRDEKLKVLRSLSPIDSKNYEELTVRGQYRAGASSGGAVKGYLDEL-ESKSNTETFVAIKAEIANWRWAGVPFYLRTGKRLATRASEIVVSFKPIPHSIFGESAGRVVANQLVIRLQPDEGVKQWMMIKDPGPGGMRLRHVPLDMSFAESFQARSPDAYERLIMDVVRGNQTLFMRRDEVVAAWQWIDPILKAWADNEQPPYGYTAGTWGPSASIALIERDGRTWHEAEAMPKVGGKNIEVLFSAQTIADRNLELAKGIVARNFENLLVISILKGSFIFAADLIRAMHDAGAEPDVEFITVSSYGKGTESGEVRLLRDIDSDVHGRDVLLIDDILESGKTLKFTRELMLERGARSVSIAVLLDKSVKRKTDIEADFKGFECPDYFVVGYGMDVGHSFRQLPYVGVVTE--MDELIARISANVGTDPATATKAVGLILAFLQKEGPADKVGQLIAGFPGAEAAI--AEAQG-SGGLLSGLMPGVMGLGSKLMGIGLGMGEITGISKETIAFAREKAGSGPVDEVVNSIPGLSQFVMSGVNEIRSTFLNYFKKNGHEIVASSPLVPRNDPTLMFTNAGMVQFKNVFTGIEQRPYNRATTSQKCVRAGGKHNDLDNVGYTARHHTFFEMLGNFSFGDYFKEEAISLAWNLITKEFGLPKDKLTVTVYHTDDAAAGFWKKIAGLPEERIIRIATSDNFWAMGDTGPCGPCSEIFYDHGEGIWGGPPGSADEDGDRFIEIWNLVFMQFEQVTKEERIDLPRPSIDTGMGLERLAAVLQGKHDNYDIDLFQTLIHASEEATGVKAEGKFRASHRVIADHLRSSSFLIADGVLPSNEGRGYVLRRIMRRAMRHAQLLGAKEPLMWRLLPALVREMGQAYPELIRAESLISETLKLEETRFRKTLERGLGLLGDATENLSDGDKLDGETAFKLYDTYGFPLDLTQDALRQRGIAVDTDGFDAAMERQKAEARASWAGSGDAATETVWFSVRDKLGATDFLGYETEKTEGIITALVQNGKLVTSAEEGTDVSIVVNQTPFYGESGGQQGDTGIISGDGFSVTITDTQKKNDGVFVHIGKVTKGTVKVDAAVELTVDSTRRTRIRSNHSATHLLHEALRETLGTHVAQKGSLVAPDRLRFDFSHPKPISDKELAVIEDLANEIVLQNAPVTTRLMAVDDAIAEGAMALFGEKYGDEVRVVSMGTALHGNKEGKSYSTELCGGTHVRATGDIGLIRVVSEGAVAAGVRRLEALTGEAARRYLEEQDDRVKAIAGALKASPADALSRVTALIDERRKLERELTEARKQLALGGG-SAAGGAQSETVNGVGFIGKVVSGVNPRDLKPLADDAKKTVGSGVVTFIGVSDDGKASAVVGVTDDLTAKFSAVDLVRVASAALGGAGGGGRPDMAQAGGPDGSKAEEAVSAVREALAGMPSYRAPVEDTLFILNDVLGFERYNNLPGFGDASPDIVEAILGEGAKLAENTLFPTNQIGDHEGCVRHDDGSVTTPTGFKEAFDQYREGGWVGLAVPQEFGGQGLPYALHVAAGEYMASANMALVMYPGLTQGAIAAILTHGTDEQKQAYLPKMTDGTWTGTMNLTEPHCGTDLGMLRSKAVPNADGSYRISGQKIFISAGEHDMAENIIHLVLARIEGAPEGTKGISLFIVPKFNLDENGNPGTRNGVSCGSIEEKMGIHGNSTCVMNYDEATGYLIGGENKGLRAMFTMMNEARLGVALQGLSIGEIAYQNAAIYARDRIQGRSLTGPKQPDKKADPIIVHPDIRRILLTIKSFNEAGRAFILWSALKSDIAHRSGDEAERQLADDILGLMTPVLKGVLTDKGFEHACMAQQVFGGHGYIEEHGMSQYVRDARIAMIYEGANGIQALDLVGRKLGANGGRAVMAFFKEVGDFCEENRGDEKLSFFTKHLKKGLNDLQAASMWLMQNGMANPDNAGAASTDYMHLFGLVSLGYMWARMAKASHAKLDEGAGNKT-FHETKLVTGKFFMERVMPETSAHLARIQTGADTMMALAAEAFMRILVVEDDRDLNRQLVEAMTDAGYVVDKAFDGEEGHFLGDTEPYDAVVLDIGLPQMDGLTVLEKWRRDGRIMPVLLLTARDRWSDKVAGIDAGADDYVAKPFHIEEVLARLRALIRRAAGHASSEISCGRLRLDTKTSKATVDGTALKLTSHEFRLLSYLMHHMDEVVSRTELVEHLYDQDFDRDSNTIEVFVGRLRKKM-GMDLIETIRGMGYRIK-------SQDSKVMNLIRSYNNWRRYRDTVTELSRLSARELNDLGISRGDIAFVAKKAASAQMRRIIKGLLWIVFSLIPIAIVASILGYVWLARSVAPATGEISLADLSGPVTITRDKNAVPHISGNSIDDVLMALGFVHAQERLWQMEMNRMAGQGRLSEIFGDKTISTDRFIRSIGLYESAESSLASLGDPDRQKIEAYVRGINAFIASPGPVFGAKYSPEFVILGDSPEKWIAADVIVTLKLMSVTLAANIDDEVLRLKFARLGMSDAEITDLLPPVPGDAPPPLPDLRQLLGLSSGTLKAGAIEAEKQFASLNEIMGSGASNNWVVGGARTESGKPILANDPHLGLTSPGIWYLAHLQVKNSDGTLKNLVGVTLPGAPLVLLGRNDKVAWGFTNTGADVQDIFIEKTNPDDPNQYQAPEGFRPFEKKGVTIKVKGGDAVKFDRLVTRHGPVLPADYRGLDHYLPEGTVASLSWTALAGDDKTISAGLKLWEFASVTEFQNGMRDFVTPMQSIVIADTNGDIGLIAPGRVPVRDPANQIMGRAPSPGWDATYDWKGFIPYEELPRVYNPADNVLATANTKMVDASYPHFLTFDWDEPWRFERIKTLVYGAN-QQTLETNRKVQGDAHSNAYAALAPIMLNLVERHNDVDLDVLKQLNAWDFVEDRARIEPLFFNAWLRMATKRIFEDDLGDVFPSFWQGHVGAMLRWLGPNPARDWCDDRRTPEKESCGDVLALALGDAVSDLDMRLGSDRSKWNWGALHYAYGAHRPFSQVSPLDRLFDVTVPASGGAYTLDRGKSSFTDDSNPFRVTHGTSYRGLFDLAD--LDRSTYIQTSGQSGNVFSAN----YRDFAQKWANTEAITIPIDEKRYEEGL-LGVWRLKPKNMSFALS-SARRLRRMSTIMVLCSCTALNACGMGDVRRPMADVGSSTDTAVAEQSLASEENQQVATEDTQRLVVPESEQMASEELQPLSTPDDQQIANQD--------------TQLPSTPEPDPVINEENQ-----DDSHG------YPGG-ISDPIARGENLYAMSSSEVACRTQLKRLGVVFQEREPINDGGVCRIDHPLRVSGFSSGRIKLKPSATLNCQMTLAFARWVKGDLSPSTRLRYLSGINTIHQASSYSCRTMSNQRGRSMSEHSKGNALDIAKITLNNGRDIKVQKPGFFAFRQRGLLNSVRSDACDYFTTVLGPGYDRFHRDHFHFDLMQRRTGRRACR--------------MTYLLGIDGGGTGCRAALADMNGQVLGTGKSGSANIMTDMNTARLNILDATEAALADARIDHSAIPSLSAVLGLAGANVGSNAAQLQAMLPFRNSLVYSDGVIALQGALGDHDGTVVILGTGSAYVTRVGSDIRFAGGWGFKVSDLGGGARLGRDLLEETLLAYDKFHPSSPLTEAIMERFGGNPHRIVQFAHSARPSDFGSFAPMVFEYASQKDDVALALLRKTIGQIEEGLDAITANNPTRLSLLGGLGALYGPLLSPRYRAQLQTPLNDALTGAVQLAAQNFASKVTE--TVHG-----MSFLKIKDLHKSYGNVPILKDINIEIDEGGFLVLVGPSGCGKSTLLNTIAGLEPITSGDISIKGKSISGLHPSKRDIAMVFQSYALYPNMTVGGNIAFGMEIRGVPKPERDKAIKQVADMLQIGHLLDRKPSQLSGGQRQRVAMGRALVRNPQVFLFDEPLSNLDAKLRVDMRTEIKRLHQRMKTTIVYVTHDQIEAMTLASKIAVLKDGVLQQFGTPAEIYNNPTNLFVADFMGSPAMNLLKAKIDGSASDLHVVLDRPDAAPLRLPIKKT-NNA---LVGYAGKDIIFGIRPEALTDPDGADRNAGAIVEGECLIEVVEPAGSDTFAVTKLGGKEVVARLRADASIIAGQKTQLAFNLDKAVFFDPQSQLRIGAMTEAYIYDHVRTPRGRGKKDGSLHEVPAVRLGAHVLEALRDRNGLDTGLVDDIIYGCVDPVGEAGAVIPRSSAFEAGYDFKAPGMQISRFCASGLDAVNFAAAKIVQGADDIVIAGGVESMSRVGMGMSGGAWYMDPSVGLPGYFMPQGVSADLIATKYGFSRDDVDAYAVESQKRAGEAWKKGYFKNSVLEIKDQNGLTILDHDEHMRPTTDMQALASLNPSFVMPGEMGGFNAVGIQAHPEVETINHVHHAGNSSGIVDGAAGVLLGSKSAGKAIGTKPRARIRAFANIGSDPALMLTGPVDVTEKLLKRAKMKISDIDLFELNEAFAAVVLRYMQAFDIPRDKINVNGGAIAMGHPLGATGAMILGTVLDELERRDLNVALVTLCIGAGMGTATIIERVMAVYTDINEIDLAHFLKDYDIGELLSYKGIAEGVENSNYLLHTSTGSFILTLYEKRVNSNDLPFFLGLMRHLAGKGISCPLPVNQKSGSSIGELAGRPAAIVTFLEGMWMRRPTVQHCYAVGEALARMHVAGEDFPMRRRNTLSVDGWRPLWDNSKSGADRVEPGLAKETEADLAFFEANWPSHLPHGVIHADLFPDNVFFLGSKLSGLIDFYFACTDLLAYDVAVCLNAWCFEKDHAFNLTKGTALLRGYSTVRPLSVDEAACLPILARGSALRFMLTRLYDWLNTPEGSLVVKKDPMEYVRRMRFHRQIQSAVEYGLVLEGQ-----------KT---------------------------------MTDLSYQLYSSRNFPPLDQTLEMLKRNGYAQVEGYGAVYSDPIATRKSLDAHGVTMPTGHFSIDLLEKEQQKVLDIAGILGMKAIYCPHLAVDLRPTDAAGWSAFGERLEAAYATYSKAGYVFGWHNHDFEFKALPDGSIPQKLIFDAAPSISWEADIAWIIRGGADPFQWIENHGSRITAVHVKDIAPSGQNTNEDGWADVGYGTVDWKALMKILKGTPAGYFIMEHDNPSDDERFARRSIVTLKAF-------------------MATFVGFLAIIMWAMLALFTDVSGKVPPFQLTAMTFAIGASLGLVSWLWRPGAARHLRQPARVWLLGVVGLFGYHFLYFTALRNAPAVDASLIAYLWPLFIVVGSALMPGEHLRWFHIVGTLLGLSGTVLIITKSGGVGFDSQYSFGYAMAGLCAISWAAYSLLSRRFAHVPTDAVTGFCAVTSILSLISHLLLEQTVWPEGIIQWLAVLGLGLFPVGAAFYVWDYGVKHGNIQVLGASSYAAPLLSTLVLIIAGVTEPTARVMLACLLITLGAVLAAKDMLLKRR-----------MSILNGKANSAGAASAKRTRSLTVLDIGSSKVCCIIARLRPREESAHLPGRTHRIEVLGIGHQKSRGIKSGVIVNLDAAEQAIRLAVDAAERMAGLTVDSMIVNISSGRLKSESFSASINLGGHEVDQADIRRVLAAGAKQALATERHLVHSLPIAYGLDGERGIRDPLGMIGDTLGVEMHVLTADAAPLRNLELCVNRCHLSVEAMIATPYASGLSALVDDEAEMGAACIDMGGGTTTISVFSEGKFIHADAIAIGGNHVTMDVARGLSAHMDDAERLKVMHGSALPSAADDRDLISVSPLGNDGRDVPNQYPRAVLTRIIRARVEETLELARDRLNQSGYGQIVGKRVVLTGGASQLAGLPEVARRILARNVRIGRPLGVTGLPEAAKGPAFSAAVGLLIYPQVAGIEERSVKAGSSSLMTGTGGRFQRIGQWLRESFMFVELSFLKQIADVAAEQTLPRFRNISLVDNKYQTGFDPVTEADREAERAIRRLINASFPEHGILGEEFGGENLDQSHVWVIDPIDGTRAFISGIPVWGTLAGLTIDGDAVAGLMAQPFTKELFMCDGEASWYE--GPGG---TRRLETRKTIELSEATLFTTTPALFKDEKREAYDRVEQTVRLARYGTDCYAYAMLAAGFVDLVVETGLHPYDIVALIPIIEKAGGVITMWDGGAAEKAGNILAAATPELHARALDLLNR-MKINGNEIRPGNVIEHDGSLWVAVKTNAVKPGKGGAYNQVELKNLLNGTKLNERFRAAETVEKVRLEQKDFSFLYEQGEALIFMDTDSYEQLELQKEFVGDRAAFLQDGMMVTVELYDERPIGISLPDQVTLAITEADPVVKGQTAASSYKPAVLE-NGIRVLVPPFISSGERIIVDTNELTYIRRADMTAPRTLYDKIWDDHLVDSQADGTCLLYIDRHLVHEVTSPQAFEGLRVAGRKVRHPEKTLAVVDHNVPTSPDRIHGIKNEESRIQVEALARNAADFGVEYYSEKDIRQGIVHIIGPEQGFTLPGMTIVCGDSHTSTHGAFGALAHGIGTSEVEHVLATQTLIQSKAKNMLVRVDGVLPAGVTAKDIILAIIGEIGTAGGTGYVIEYAGEAIRALSMEGRMTICNMSIEGGARAGLIAPDETTFAYVKDKPRAPKGAAFEQAVEYWKTLKSDEGAHFDKVVVLNAADLPPIVSWGSSPEDVISVTGAVPNPDEIADETKRSSKWRALDYMGLKPGTPITDIKLDRVFIGSCTNGRIEDLREVAKVVEGRRVSETVDAMIVPGSGLVKEQAEAEGLDKIFKAAGFDWREPGCSMCLAMNDDRLKPGERCASTSNRNFEGRQGFKGRTHLVSPAMAAAAAIAGHFVDIREWK---------------------MLSLSWPMILTNLAQTAMTATDVMMMGWVGASTLAAGALGTNFYYLLMIFGLGLMLATSPMMARELGRNRFAVREIRRTVRQGLWVAVAVSIPIWLVLWNTESILVAMGQDAGLAGQAGNYMRALQWAVLPFYGYIVLRSFIAALERPGWALVIAIVSVAFNALANWVLIFGHFGFPKLGIVGSGIATTLSSMMMFAGLVVVISMDPKFRRYRLFGRFWRADWPRFADLLRLGLPIAAILSFEVTIFNAAAFLMGLIGADSLAAHAIAIQIASIAFMVPMGFGQAATVRIGRAYGAQDRDGIKRAGWVAFAAGTGFMVLTALLMLLAPHQLISAFIDINDPANADVISLAVTFLAFAALFQIFDGGQAVASGMLRGLHDTTIPMIYAAIAYWGIGLPLSVGFGFWLDMQGAGIWVGLLCGLAAAAVLLISRWLRREKLGIEIIR------MFSKQILLVTISVVSMTLGGCASYRPAPAAFNEAINKPYMLDAGDRIRLTVFEQEGITNTYSVDQAGYISVPLIGSVPARGKTIQQIEADVAARLRKGYLRDPDVAVEIDRYRPVFVMGEVGAAGQYSYVPGMTAQKAIAAAGGFTPRANQGDVDITRQFNGESLTGRVIISDQVLPGDTIYVRERFFMSATVLHNARIVDPSRNLDELGSLIIKNDKIVASGADTRNQGVPEGATVIDCQGKAVLPGLVDARVFIGEPGAEHRETIASASHAAAAGGVTSMIMMPDTDPVIDNVALVEFVMRTARDTAIVNVYPAAAVTKGLHGEEMTEIGLLREAGAVAISEGRNSIANTQLMRRVLTYARDFGVVLAHEARDPHLGITGVMNEGLYASWLGLSGTPREAEVIPLERDMRLAALTQGAYHAAQISTAMSAEVISRAKQNGLNVTSAVSINHLSLNENDIGEFRTFFRLEPPLRLEEDRLAMVEALRDGTIDIIVSAHDPQDVDTKRLPFADAAAGAIGLETLLGAALRLYHNGDVPLLRIIEAMSSAPARIFG-------LDAGSLRPGSTADVTIVDLDEPWVVKEEDLRSRSKNSCFEGARFQGRVVQTFVAGKSVFTI--------------------------------------------------------------------------------------------------------------------------------------------------------------------------------------------------------------------------------------------------------------------------------------------MVDPGRSPLGRVARTLILAGIILVMLPIILLSLYRLPFIHPVSTLMVKDLVTFSGYDRRWVSIDQISPTLVNSVMMSEDAKFCFHSGVDWDALNSVVSDAIDGEQTRGASTITMQTAKNLFLWTSRSFIRKGLEVPLAVITDLILPKRRLMEIYLNIAEWGPGIYGIEAAAQHHFNVSAARLSPRQAAYLAVTLPNPIVRNPAKPGRGLQTLARVNERRARGAGAYIGCVN-----MIRTATFGLCLLF---CSVAYAEDKIGVIAPLSGSFARLGTQLLDGATIAVATNKN-GQTISIVSADDKCDAAGGAAAATQMVQANVMIVVGFLCTESLEAALPILSQKGIPVITPAIRSRTLTELRTEQPYPVFRIAPSDRNEAEETGDILAERWRSVPFAIIDDGTIFGRELASGVRARLEEKGLKPVFADTYRPGLDNQNALVGRLKRADATQVFVGGERDDVAAIGRSAVALGYPLVIASGEALNATGNG--LAIGTLMIAPREPQLLESARNAKNAIELAGKVPEGYTLPAYAAVEVAIQVL-TMAQSQAGSLSELLRKN-VFDTALGSIQFNTTGERVADTYRLQKYDGSQFVLEI--NMTE--PRKPAAFRLETPQPA----AHKTGSAS-------PQSRRPAAIKDAAVITPAAIDVFDLDPAAADELDALTPPPALAQKRRFSFASLLTGALGILLSLALGLWADNLIRTLFERAPWLGWLALGVTILAFFALVAIVIREGLALRRLASVQNLRDEAAKAAITNDARTAKVAVGKLVAIAETLPATAKGRALLHDLRDDVIDGRDLIRLSETELLRPLDRQARDMILAASKRVSIVTAVSPRALVDIGYVVFESARLIRRLSELYGGRPGTLGFLRLTRNVIAHLAVTGTVAMGDSIIQQLVGHGLAAKLSARLGEGVINGLMTARIGISAMDMVRPFPFDAEKRPGIGDFIGDLGRIGGVKKE--------MIQKNWQELIKPNKVEFQTSGSKTKATVVAEPLERGYGLTLGNALRRVLLSSLRGAAVTAVQIDGVLHEFSSIPGVREDVTDIVLNIKEIAIHMESDGPKRMVVRKEGPGVVTAGDIQTVGDIEILNPEHVICTLDEGAEIRMEFTVNTGKGYVPADRNRAEDA---PIGLIPVDSLYSPVRKVSYKIENTREGQVLDYDKLILQIETDGSISGEDAVAYAARILQDQLAIFVNFDEPQK---EVQQEQVTELAFNPALLKKVDELELSVRSANCLKNDNIVYIGDLIQKTEAEMLRTPNFGRKSLNEIKEVLASMGLHLGMEVPSWPPENIEDLAKRYEDQYMKRKLLLACTGFVLFAGPALAEEPIVGSWKRPNGTIIQYTSCGSDTYCGTVMTGEYKGKSIGSMSGKDGAYKGKVNKLDEGKTYNGKASVKGSTLSLSGCVMGGLICKSESLTRQ-----MELGIYTFAGMSPDPATGVAISAQQRMQNLIEEMMLADEVGLDVFGIGEHHRPDFVISTPAVALAAGASRTKNIRLTSAVTVLSSDDPVRVFQEYATLDLISSGRAEIMAGRGSFIESFPLFGYDLNDYDELFAEKLDLLLKLRTSERVTWSGDFRAPIDNLAIYPRPLQDPLPVWIAVGGTPASVVRAGTLGLPLAVAIIGGEPHRFAPLLELYRKSGAKAGHAPETLKVSINSHGYIAENSQAASDEYWPSYEVVMNKIGRERGWPPSNRAQFEAGRSMRGALLVGSPQEIIDKIMLQHSYFQHDRFMLQIDLGSLPHANTLKAIELYGTNVAPVIRKELGGATGS-KTGKDVAIAGIDAVRI--MSKKTADEHAIDISLLHLSDAHEFAPLLASYAQALKRGAPRRPDDYYAENLLRDRAAEIAGARLDGHLVGFVIFYDLPEPVSGMRAGQVDHIYVHHDHRGKGIAKALIDVLADKAEERGWTKLSLNAPRVPEDGRKLYEQVAAPADWTSFIIRFGNQMDKRIGTKKPVTISMPRHVEPEEFTDAKAAVEALSALYERNTQFLRDAFDKVAKGEALDTTHFRAFYPEIRLSTSSYAQIDSRLAFGHVSSPGQYAATITRPDLFGNYLEEQIRLLIRNHGHPITVQESTTPIPIHFAFLEGTYVESSVADAFTRPLRDLFDVPDLNATDDRIVNGEYETDDIDGIMPLSPFTAQRVDYSLHRLSHYTATRPMHFQNYVLFTNYQFYIDEFCALARKLMAEGGGGYERFIEPGNLVTEAGDDKPSSGVPLGRLPQMPAYHLARSDSSGITMVNIGVGPSNAKTITDHIAVLRPHAWLMLGHCAGLRNTQALGDYVLAHAYVREDHVLDDDLPVWVPIPALAEVQVALELAVAEITGLEGYDLKRIMRTGTVATIDNRNWELRDQRGPVKRLSQSRAIALDMESATIAANGFRFRVPYGTLLCVSDKPLHGELKLPGMATEFYKRQVAQHLQIGIRALEKLAEMPPEKLHSRKLRSFFEMAFQ-MSAA-LIDLTTERNRRSPNPEQIIIAGAGIAGLTLALALSDKGFQVRIFEKSATLAEIGAGLQLSPNATRLLDRLGVLERLKHSSVEPQAICLQDGTSGEKLLHLPIGGQAKERWGAPYIVCHRADLQNALLTEVRSRSNIIVQLQSAVTHHKADAETILVRAQYHDRIEEHRGALLVGADGVWSNTRTAMSDTDRARYTGTIAWRSSVAVDELPESFNALMPPGTNVIAWTGANAHLIAYPIRSGQMINFVAVAPDRQNGRRWDLQINPEGVSQTFVREFAGWHQGIRDVLR-AGKPWTPWRLFEMKKCRFRLEDRLVLIGDAAHAMTPFAAQGAAMAIEDACALAGALGADNTKWDAALGAFASARTQRITAVVRRGALNQLAYHARGPVAVARNILLRNRPVEKFVKGFDWLYGFDAEGPLDQK---------------------MASRDRGRQRIEPGFSSDRRSG---DDLRADPEDRPV---AKRKTVRKS-TKSKRGKSRRGSGSGGFFGLLRRAVYWSFVLCIWGGIALAGTVIYFAAKMPQTTTWSIPDRPPNVKIVSVEGDLIANRGASGGEAISLHDMSPYLPEAVVAIEDRRFYSHFGVDPIGFARAMAANVMSGRLVQGGSTITQQLAKNLFLTPDRTIERKVQEVLLALWLEQKYTKDQILEMYLNRVYLGSGSYGVAAASRRYFDKSAKEVTLPEAALIAGLLKAPSRLSPARDPKAASERAQLVLAAMREQGMIGDKELTFAMKQPATRAASYWSGSEQYVADRVMEELPGLIGDVRSDVIVDTTVDLGLQKLGEASIRDLISKNGEKLNVSQGAMVSIDGTGAVRALIGGYDYANSQFDRASEAKRQPGSAFKPFVYLSALEQGFTPESVRNDAPIRIGNWTPGNYNGKYFGKVTLAEALARSLNSVSAQLVMEVGPKTVVSTAHRLGIESNLTSNASLALGTSEVTLLELTDSFVPFANGGYKAPLHIIRRV-TTNEGKVLYEYKSPAPNRVIDLRNVGMINAMLRQTVESGTATKAKF-GWPAAGKTGTSQNFRDAWFIGYTSNLATGVWFGNDDGRPTKKITGGSLPAIAWKDFMVAAHKGVPVASLP-GEYQLDQQVNDGNDILPSSGV----DPA-----APASEG-----VPSAQLNPAGPMPPVDVG-NQTGSTRPVP------PANVGSNG-QKGRKTTTLFDLIMGN----MSLDFQPLVSSTLLALLLVPLVLLTLAGIYFRQRGSLIRLLAVLAFAMALFNPIVVNEEREPLKSVVAVIADRSQSQDIGNRSADTDAALKEVQTTLARLPQFEVRTVETGRVSENDDATSTRLFQALNGAFRDVPPARIGGAIMITDGQIHDIPANPTGLGFNAPVHGLITGTPGEIDRRIQFVRAPRFGLTGKPQQMTYKVTGAGE--PQGGRAHVRVRVNGNEVNSEDAIVGEEMPLEVTLPRAGVNIVEIVVDSVPDELTEVNNRAVAMVDGIRENLRVLLVSGEPHNGERTWRNLLKSDASVDLVHFTILRPPEKQDSTPINELSLIAFPTRELFVDKIEEFDLIIFDRYQHRDVLPLLYYDYINDYVQKGGALLIAAGPEFAGNMSIANTPLLSVLPATPTGNIDETAFYPRLSDQGKRHPVTRGLEGSAQEPPNWSRWFRVIDVNPPEGNVVMNADD-RPLLVLNRMGEGRVGMFLSDQGWLWARGFEGGGPHAALYRRIAHWLMKEPELEEEALTATGSGRNLEIQRQTMKKTADPASIITPSGKTLSVPLTETEPGIFTAALPTDEIGLYQVANGDLTTLAHVGPVDAPEFADNVSTTAKLDPLARETGGGTRRLRADADQSTIEVPNIVASRGIASASGDNWIGLRPTNETVLKSVDRLPLFSGFLGLAVMILVLGSMWYREGKMTTAKDILKQIKDNDVKFLDLRFTDPKGKLQHVTMDVAEVDEDMFADGVMFDGSSIAGWKAINESDMVLMPDTDTVHMDPFFAQSTMVILCDILDPISGEAYNRDPRGTAKKAEAYMKAEGIGDTIFVGPEAEFFVFDDVKYKADPYNTGFKLDSTELPSNDDTDYETGNLGHRPRVKGGYFPVPPVDSAQDMRSEMLTVLTEMGVRVEKHHHEVAAAQHELGIKFDALTRNADKMLIYKYVVHQVANAYGKTATFMPKPIFGDNGSGMHVHMSIWKEGKPTFAGNEYAGLSENCLFFIGGIIKHAKAINAFTNPSTNSYKRLVPGYEAPVLLAYSARNRSASCRIP--FGSSPKSKRVEIRFPDPTANPYLGFAAMLMAGLDGIKNKIHPGQPMDKDLYDLPAKELKKIPTVCGSLREALQSLDKDRGFLKAGGVFDDDQIDSFIELKMAEVMRFEMTPHPVEYDMYYSV---------------------MASKTGD-IVIKKYANRRLYNTGTSTYVTLEDLADMVKRNEDFTVQDAKTGEDITHSVLTQIIFELENKDGQNMLPIPFLRQLIAYYGDQMQVVLPTFLEQSMSAFAKEQERMREQLTAAFGKTPMDMMNVSAPLKLVEEQVRRNTEMLQNAMRMFTPFPIGKTG--AEPDETA--APEKPTRDT--GLDELKEQIAAMQRKLDSLDKMVSTGEKTRVDWVDIAKGICIIFVVMMHSVLGVENEAGARGWMHPVVAFAQPFRMPDFFLISGLFLGLVIDRPWLRYTDRKIIHFAYFYVLWLTIQFLFKAPGIIAESGIGGAITAYLLAFVQPFGTLWFIYLLPVFFVFTRLLKGVNVWFVLGVAAALETLPIHTGWLVIDEFCSRFVYFFAGYAFAPAIFRLADWLRQRPLVALTILGFWALTNGWLVFHPASEPFSAWIPEESYTSGGLGGWSSVPVISLTAGFAGALAIVSVSALIA--GSLSSWERVVTPLRWLGAHSIVVYLAFFLPMAIARTILLKTGIITDIGTISLLTVASGVLSPVILYGLIQWSGYGQFLFKRPQWAHIDRAPAR---REALASAE---------MHKAWLGLGGNVGDPVASMGKALRALHRRSDTRVRAVSAVYKTPPWGKTDQAWFHNACAEVETLLAPEALLATCLDIEKRMKRQRIERWGPRIIDIDVLAYEGEETFGSPTLVLPHPRMTERAFVMVPLSDIAPQLAVSGRSVAEWSRLCDRSGIEKARSDAGWWRQTP----------MHLLLAQKGTIAEGTEAIDLGQTPGDVLFLSAADTELASLASAQRQRTGEVSLRLANMMALTHPMSVDAYVERTARHAKLIVVRVIGGESYWPYGLEALHACAINHKVQLAVLP----GDDKPDPGLDRFSTIARQDRDALWHYLLEGGAQNAQRFLGYCAALIDGEEKPQDAA--PLLKAGLWWPGSA--APSLNEMRGH----W--------------------------------------------------------LDN---APAIAICFYRALVQSGQTQPVEALIEALKAKGLNPLPVFVSSLKDPVSVATLEAIFGEAPPSVVLNATGFAVSAPGAARKPTVLEHDGAIVLQVIFSGSSADQWRNSDQGLSARDLAMNVALPEVDGRVLSRAVSFKSARQYDEAVETNIVTHEPQPDRVEFVAALAANWARLRGTKPETRRVALVMANYPNRDGRLGNGVGLDTPAGTWHVLNTMREHGYAVT-----NVPSDGDALINHMMAGPTNAAS----DGREVRETISLNQYKNFFQSLPKPIQQAITERWGAPEADPFF--VKGTQTFALPLARFGETLVGIQPARGYNIDPKDTYHSPDLVPPHGYLAFYAYLRVVYGAHAIVHVGKHGNLEWLPGKSLALSENCYPEAVLGPVPHIYPFIVNDPGEGTQAKRRSSAVIIDHLTPPLTRAESYGPLKDLEALVDEYYEASGVDPRRLVRLKVQILDLVRDIGLDHDAGI-HDHDPDDLA--LQKLDAYLCDLKEMQIRDGLHIFGRSPE-GHLLTDLVVALARVPRGAGHGADQSLQRGIALDLGLAG------------------------------------------------------------FDPLDCDMAAPWNGVHPEMLQDVSDSPWRITGDTVERVELLAAKLVAGEAECPSEWENTTLVLEGIRTNLHPAVTACGDAEMSALLTALDGRFVAPGPSGAPTRGRPDVLPTGRNFFSVDSRAVPTPAAWELGQKSAELLIRRYTQDH-GEWPTSFGLTAWGTSNMRTGGDDIAQALALIGVKPVWDMASRRVTGYEIIPTALLGRPRVDVTLRISGFFRDAFPDQIALFDTAVRAAGALDEDESDNPIAARMRSELARLVAEGSDEKLARQRAGFRVFGSKPGAYGAGLQAL-IDEKDWAKRDDLAEAWLVWGGYAYGAGEEGTAER--GLLEERLRTVNAVVQNQDNREHDLLDSDDYYQFEGGMSAAVELVSGEQPTIYHNDHSRPEKPVIRTLEEEIARTVRGRVTNPKWIAGVMRHGYKGAFEMAATVDYLFAF-AATTGAVGDHHFEGVYQAFLVDE---EVRSFLKDKNPHALRDIAQKLEEAVTRGFW-----TPRSNSARYE------------------LSSLAA-------------------------------------------------------------------------------------------------MSSGAAT--DAPF-------FAQALRDLEPLDKQNAETLLHEFLKRAAEKECSGTGTLTANKPAAAFVAAVLDLSPYLRAILLRRPQIIEPLFEIPLSRRLEALMLEIAATSEGDDVTEAGLMTALRQSKLKGHVLIALGDLSGQFVTSEATFWLSRLAEECLGAAVRFLLRDAHEAGKLKLPDLKHPDKNSGWIILAMGKFGAFELNYSSDIDLIVFIDEHSPAITDPYECVETFSRLTRRLVRILQDRTADGYVFRTDLRLRPDPGSTPLAIPVGAAINYYEGRGQNWERAAMIKARPVAGDIDAGKRVVAELAPYVWRKYLDYAAIADVHSIKRQIHVHKGHGEIAVRGHNVKLGRGGIREIEFFVQTQQLIAGGRFPKLRGSRTVEMLGALHGLGWISEEARDTLAEKYGFLRDVEHRIQMIADEQTHMLPEDDENFLRVAHMMGYRDGETFADDFRAALKTVETHYAALFEQAQDLTGEAGNLVFTGDVDDPDTLKTLAKFGFERPSDICRVIRTWHFGRYRATQSAEARERLTELTPVLLKAFGATSRADEALMRFDGMIKGLPAGIQLFSLLQSNPRLLDLLVLIMGAAPRLADIITRKPHIFDGMLDPAIFADVPTRAYLAERLEGFLGPCKVYEDILDRLRIFAAEHRFLIGIRLLTGAISGGRAGKAFSHLADLVIDRALKAVMDEFASKHGRISGGRIAILGMGKLGSRELTAGSDIDLILLYDHDEHAEESDGQKGLAPSQYYMRLTQRLIAALSAPTSEGVLYEVDFRLRPSGNKGPVATHIDAFRKYQRTDAWTWEHMALTRARPVAGDGTFFDEIEKDVSDILGLPRDPAKIAKDVAEMRAMIEAEKPPSDAWDLKLVAGGIIDIEFIAQFAVLTGNVDGPGNGQPTAEVLAKLKPDFADPAVTDSLVEAAHLYTGITQIIRLCLNGDVKREDFPPGLSELLCRACDLPDLERVESQLAETAQWVRKTFDTLLKDARNQAKSSKMNWFSLTNEEWTAVHLTLRIASVAMLVSLPFGIAVGWLLARGRFWGKSILNGLVHLPLILPPVVTGFILLLLFGRRGPIGAFLAEHLGI-VLSFRWTGAALACGVMGFPLMVRSIRLSIEAVDRKLEDAAGTLGASPLWVFTTVTLPLILPGIIAGMILSFAKAMGEFGATITFVSNIPGETQTLSAAIYTFTQVPG-GDAGAFRLTI-ISVIISMIALMASEFMAY-VVGRRVDIE--MSVPHGTKRAFGKN--QKPANFIDAEDRPGLAARQCATRLLGAVIEKHTSLDGLTDNTGGHPQYMALDDRDRSLVRAILGAALRNRGTIEAAIGQLIDRPLPENAVALRHLLHIAAAQILYLDVPDRAAVDLAVTAANNDPRNRRFASMVNAVLRRLSREKE------NLVS----EASNVPAWFEESLIAAYGKEKTRAILAIQSKEPPIDLTVKGDAQEWALKLGGIALPTGSVRLG-----SVDGALTALPGFAEGDWWVQDTAASLPAKLFGDIRDKRVADLCAAPGGKTAQLALAGANVTALDLSANRLKRLRSNLERLELSAETVEANLSAYQPAELFDAILLDAPCSSTGTVRRHPDVPWTKTLGDIQKLAHLQAKLLDHAVTIVRPGGIVVFSNCSLDPLEGEKVANDLLAKNPAVELVPVTKDEVGDLAELITPEGFV----RSTPADLRHENPALSGMDGFFAARFKRKY---------------------MTRSSYLFTSESVSEGHPDKVCDRISDEIVDMIYKEAKKTGVDPWTVRIACETLATTNRVVIAGEVRVPDTLLKKDKNGVVVKDAKGHPIINPSRFRAAARRAIRDIGYEQDGFNWKTAKIDVLLHPQSADIAQGVDNAADRQGEEGAGDQGIMFGYACRETPDLMPAPIYYSHKILELLAGARHKGEGDAGKLGPDAKSQVTVRYVDGVAAEATQIVLSTQHMDASWDSKKVRNVVEPYIREALGNLKIADDCVWYINPTGKFVIGGPDGDAGLTGRKIIVDTYGGAAPHGGGAFSGKDTTKVDRSAAYAARYLAKNVVAAGFADRCTIQLSYAIGVAQPLSVYVDLHGTGSVKEDAVEAALRKVMDLSPSGIRKHLDLNKPIYAKTSAYGHFGRKPGRDGSFSWEKTDLAKQLKTALAA--MAERSQNLQDLFLNSVRKQKISLTIFLINGVKLTGIVTSFDNFCVLLRRDGHSQLVYKHAISTIMPSQPVQMF--EVEETD--MPALEPVLNTLDNNLDKSLERLFDLLKIKSISTDPGFKAECRKAAEWLVADLKSVGFEASVRDTPGHPMVVAHHDGPSADSPHVLFYGHYDVQPVDPLELWENDPFAPAIKDVGNGRKILTGRGTSDDKGQLMTFVEACRAYKEVHGNLPVRVSILFEGEEESGSPSLKPFLQANRDELKADFALVCDTGMWNAETPAISVGLRGLVGEEIVVKAADRDLHSGHFGGAAANPIRILAKVLADLHDENGAVTIPGFYDGVEETPTQILQMWDGLGTTPESFLGPIGLSVPSGERGRSILELIWARPTAEINGITGGYAGQGFKTVIAAQASAKVSFRLVHKQDPVKVREAFRAFVRERIPADCSVEFHEHGGSPAIQLSYESPLLTKAKDALSDEWSNPAVLIAMGGSIPIVGDFQKMLGMESLLVGFGLEDDRIHSPNEKYELNSFHKGQRSWARILAALAK---------------------MASVLRQSDPDFEQRFSAFLATKREVSEDVDRAVADIIHRVRSEGDKALIDYSKAFDRVDLVQHGIAITPAEIDAAVASAPAATVEALKLARDRIHSHHARQVPQDDRYTDALGVELGSHWTAVESVGLYVPGGTASYPSSVLMNAVPAQVAGVERIVMVVPSPDGRLNPLVLVAARLAGVSEIYRVGGAQAIAALAYGTETIAPVAKIVGPGNAYVAAAKRRVFGTVGIDMIAGPSEVLIVADSDNDPDWIAADLLAQAEHDSAAQSILITDDEGFGNAVVSAVERQLQTLPRTATASASWRDYGAVILVQDLDASLPLVNRIAAEHLEIATADPERLLAGVRNAGAIFLGRYTPEVIGDYVGGSNHVLPTARSARFSSGLSVLDYVKRTSILKLGVEQLRALGPAAIELARAEGLDAHGNSVAIRLNLMSNASASVA----DTFFNASLEEIDPDIFGAIRKELGRQRHEIELIASENIVSRAVLEAQGSIMTNKYAEGYPGKRYYGGCEFVDIAEELAIERAKQLFGCEFANVQPNSGSQMNQAVFLALLQPGDTFMGLDLNSGGHLTHGSPVNMSGKWFNVVSYGVRQDDHLLDMNEIERQAHQHKPKLIIAGGTAYSRIWDFERFRQIADAVGAYLMVDMAHIAGLVAAGVHPSPLPHAHVVTTTTHKSLRGPRGGMILTNDAEIAKKMNSAVFPGLQGGPLMHVIAAKAVAFGEALKPEFKTYAQNVAANAKTLADALKKNGLDIVSGGTDNHLMLVDLRPKNATGKRAEAALGRANITCNKNGIPFDPEKPFVTSGVRLGTPAGTTRGFGAAEFTEIGNLISEVLDGLKVANSDEGNAAVERAVQQKVINLTDRFPLYPYLG----MSISTFKKSLYVSGIVLALAAVGGNHFFEL-TQSHAEGEKQQAAPAQAMPVSVAVVQPKLVTQWSEFSGRLEAIDAVEVRSRVVGAVQSIAFKEGAIVKQGDLLVKIDPAPYEAEVARARAQVSAAEAKLAFAKNELERGRRLVDSRTVSQSDYDQRLNVQTSAEADLLAAKAVLQTATLNLGYTDIRAPITGRVGRIEITPGNLIAAGPSSPLLTSLVSISPIYASFEADENVVANALADLPEGLNSRDFVDRIPVQMDVQGKYG--VTGKLQLINNSVDVASGTVKVRAVFDNANGSLMPGQFAKVRMGRATERQELLVDEKAVGTDQNKKFVMVVNPQNIVEYREISLGARADGLRIVTAGLQADEKIVVNGLQRVRPGSLVAPEMVAMGGQ-APNQQALA-DQAKAKQ--MSSSVVTRFAPSPTGYLHIGGARTALFNWLYAKHTGGKMLLRIEDTDRERSSEAAVAAILDGLRWLGLDWD-----GDAVSQFERAPR---HREVAEELVAKGKAYYCYASPAELEEMREKARAEGRPPRYDGRWRDRDPKEAPA----GIKPVIRIKAPQEGETLVRDQVQGDVRF-PNKDLDDFIILRSDGNPTYMHAVVVDDHEMGVTHIIRGDDHLTNAARQTVIYNAMGWDVPVMGHIPLIHGADGAKLSKRHGALGVEAYRAMGYLPAALRNYLVRLGWSHG-DDEIMSDEQMIEWFEISDINRGASRFDFQKLEAINGHYMRFSDDADLVKAMIDVLPEIESGAEIMARLNDTTKGQLLAAMPGLKERAKTLVELADSAKYLFAQRPLTLDEKAAGLLNEEGLSVLSGVLPALSAVDDWTAEALDAAVRVHAEQTGLKLGKIAQPLRAALTGRATSPGVFDVLAVLGREESLGRIEDRIKG-MAVAAKSFTPPNLHPVRRALLSVSDKTGLTALAHALHRHGIEILSTGGTSKAIAAEGIPVRDVSDITGFPEIMDGRVKTLHPKVHGGLLAVRDDPAHTAAMETHGIGAIDLVVINLYPFEEVRHSGADYANIVENIDIGGPAMIRAAAKNHAYVGVVTDTGDYDMVIAMLEKHEGSLPYSFRQNLAAKAYARTATYDAAISGWFTEALMTETPSRRAIGGHLHSVMRYGENPHQSAGFYLTGEKRPGVATATQLQGKQLSYNNINDTDAAFELVAEFDPARTSAVAIIKHANPCGVAEGRTLKEAYLKALACDPVSAFGGIVALNGTLDAEAATEIVKTFTEVIIAPDATPEAQAIVAAKKNLRLLVTGGLPDPRASGISAKTVAGGILVQSRDSGVVDDLELKVVTKRAPTEAEMSDLKLAFRIAKHVKSNAIVYVKDGAAVGIGAGQMSRVDSARIAARKAEDAAEAAGNATPLTKGSVVASDAFFPFADGLLSAVAAGATAVIQPGGSMRDDEVIAAADEHGIAMVFTGMRHFRHMANADAYHAPTGKTYAKNRGDARGRNRLSGHVKLLADPAYGKLLAAEPYLRRAIPPLIIVFLLVLAVVRSMSLLAWRDDTERTARATLSMAASHVASVIDNRLNSLAPNGGQK------LDTGDLQNIISEMRSSELIPEGMWFAVADGDSDIIVSSDGLDRWRTRNLETFVTEGQPLFLFGTRAGAMPVKVDGTAALAAFSRTSGDAGQGTYAVFVTQPVETIFADWRRVVSINVTMFAGTATIMLIVLCAYFSQAARAQDADELYQHTQARVDTALARGRCGLWDWDMARGRVYWSRSMYEMLGYEAHDAILSLGDISSIIHPDDDKLYSVAAQVAAGDITQMDRVFRMRHAEGHWVWMRVRAQVADA-NMGDLHLVGVAVDVSEQHRFVQATAQADQRIREAIESISETFVLWDADNRLVMSNSKFNEYSGLANECLLPGISREELEPRIRAVAFEKRMANEHGRNGALTFERQLADGRWLQVNERRTQDGGLVSVGTDITQLKVHEERLVDSERRLMATIHDLSLARKSEIERTLELTELNSKYAVEKERAEAANRAKSEFLANMSHELRTPLNAIIGFSEIMHSGTFGALGSDRYVEYVHDIHTSGNYLLNVINDILDMSKIEAGHFSLDREEIDLCPLIHETVRVVSLQAQEKEVKVETRIAESVTLNADRRAIKQILLNLLSNAVKFTDTGGRISVRARKVSGALVFTIEDTGCGIPKSALKKIGQPFEQVANQFTKSHAGSGLGLAISRSLTELHGGALKIRSQEGVGTIVSVRIPTRATENV------MRRFEMIARSPVVVALFMSLAIAGCASKKNI-NSAGDLG--L-GAGA--ATP-----GSTQDFTVNVG-DRIFFDVDSSVIRADAQQTLAKQAQWLQRYPNYAITVEGHADERGTREYNLALGQRRAAATRNYLASRGVPAARIKTLSYGNERPVAVCDSESCWSQNRRAITVLGGAGS-------------MNRIVPLVLAVALFMEQMDSTVISTSLPAIAADIGTSPIALKLALTAYLVSLAVFIPVSGWMADRFGAKNVFRAAIVVFVLGSIACAVSNSLLAFVMSRFFQGVGGAMMTPVGRLVLVRSTPRNELVGAMAWLTMPALIGPLLGPPVGGFLTTYFSWHWIFLINVPIGIIGIWFATRYLPAIETLVQRPLDVPGFFLSGIAASGIVFGLSVVSLPALPTWVGLSTLALGIVSTILYLLHARRTAEPLLALDLFNNQVFRASIVGGSLFRIGVGAVPFLLPLMFQIGFGMTPFESGLITFVSALGAMSMKLVTKWFYQKTGFRNSLMYGSVVAAAFIAINGFFTPETPYWLMVVLLLAGGFFRSLFFTGTNALAYADIPNEQTSLATPISSVAQQISIALGVAVAGGILEVSTKIHGGPLQLSDFHIGFFIVAAISALACLSFRGLKPDAGAEVSGHRSLVKSAAPAE-------MKNRYEPAL------------RA-T----GHTQRGQCAKRLSILDAAAHVFCREGFSGASIDEIAVAACVSRQTIYNHYREKETLFTAVVEDVMDRANAMLYSILATFPDKSDNIEDDLTAFAVRLSKNCLCNQDGKFLRKLVQSEGERYPHLFEAWRQHGPGKISSALGALFSRLCHKGVLQIDDFDLAARQFLALVNADLQMISLFGGTPTDEELESAARNAVQTFLRAYGKPA-GTSIAQPP---SLAVVS--------------------------------MAFDDVETVKTATGATLAMRFSPADGPQRGIVQINHGVSEHSRRYSRFARFLNEKGFHVYAHDHRGHGLTKAPGAPAGRFAPSGGLLHVLDDVDAINDLARSRHPGLPLILFGHSMGGLVALNYVMKHPDKVDAAAIWNANFSAGILGRLAQGILRAERMFLGSDVPSMVLPKLTFREWGRSMKNGRTASDWLSRDPDEVDAYLADPLSGWNPTVSMWRDIFDFIFAGSDDRLLAKVPRDMAFNLVGGAEDPATAKGKAVKDLENRMRKHGFIHVTTKIYEDTRHEGLNEINRELIMQDFADWARQVAERQM----MKLKSA-------------MRFTVKHHSPIKELKKNLRLEALGRRDILDVGYRIEASMRIADIGLSDFELERGAIVSGFWPIRSEVDLRPLLFGFRERGARLCLPVIIDKQTILFRELVRGVPMVETGFGTAGPPEDAVVLDPTFMLVPLAAFDARGHRIGYGAGYYDRAIARLQEIGVNPRLIGVAFDCQEVEIVPDEPHDMPLEAILTESGLRRFGRTDKAS-MSRRQIIDLQVRLPNVT-------PMRSDLSKP---VKQDIRVFGSDILFDGSNEVGIEHAGSLYRLKITRQGKLILNK---------------------------------MTKPVNYFHLHMISDATGETLIAAGRAAAAQYAHARAIEHVYPLIRTEKQMKRVLDGIDAEPGIVLYTIIDQRLAAMINDGCAAIGVPCVSVLEPVLTIFQSYLGAPAGRRVGAQHVLNAEYFRRIDALNFTMEHDDGQLPPDIEEADVILIGISRTSKTPTSIYLANRGIKTVNVPIVIGVPIPDVLVQSERPLIVGLVASAERISQVRQHRVLGATHGFDADQYLDRANIIEELTYARQICHRHNWPMIDVSRRSIEETAAAVLALRAEGR----MAKEEVLEFPGLVTELLPNAMFRVKLENDHEIIAHTAGRMRKNRIRVLAGDKVLVEMTPYDLTKGRITYRFKMIKKTILIGAALLLTCMTALANPSLSTAAIMKTGARTSQPIGHYEFCQTYKNECNIKN-DARPTPLTSKIWTLLVQVNGSVNAKVQPATDMDIWGKAEVWSYPTTV---GDCEDYVLLKRKMLNESGIPLGDLLITVVRQMNGEGHAVLTVRTDRGDYVLDNLEPRIKPWNETNYDYLKRQAANNTGAWVSINDDRQVLVGSIETQ---MRHDATSELFGYWNRLRGTRAAPERKEITPAPMRSYLADTFILQASGTSEPRFRLAGTRICSIYGRELKGLSFASLWHTRDKNTISRLVKNSMTSKSVVQLNYEGRSARGRKVLLKLLLLPLASEANEQHLMGMITAIGRPFWLESDAIVENRIQSVSIIDPRN--QVVPGID--PIHARMEAAASVPLPSSRTI---VSRKVGHLRVFDGGKITE----------MQNNTVVITGIGLISSLGEGVEAHWQAFNDKVEPRVDSETFKPYTIHPLVEVDWNNQIPKRGDQRQMETWQRIGTYTAGLALQDAGIKDNEELCATMDMVVAAGGGERDVTVDKQILTEGRVRNDLGTMLNEKLTTELRPTLFLAQLSNLLAGNISIVHKVTGSSRTFMGEEGSGVSAIDTAVARIRSGQSTHALVGGAYNSEHPDMLLGYELNGLLKTDGWAPLWQRERGAGGGVVTGSGGAFLVLESLDHAKKRGARAYAAIDAVHGSQIRRTREDLTETIAKMVGAASADTAANLIISGACGFKGVTAAEKAALDKFPSSAVRGIAGKLGHLREAQFSLVVALAALNVFKGTAFPPIDADNEKDGPSQINTALATAIGGCRSEGVARLSRVMAILLFAEHDNETLSDQTAKALTAALAIGPDVHVLVAGKGAKGVAEQAAKLAGVKKVLLADSPDLENRLAEASAALIVSLAGNYDTIIAAATTNGKNILPRVAALLDVMQVSDIIEVISPDTFKRPIYAGNAIQTVQATDAKKVITVRTATFAAAGEGGSASVENVSAAADPGLSKFVEAKLSGGDRPELTSARIIISGGRALGSNEKFKEVILPVADKLGAAVGASRAAVDAGYAPNDWQVGQTGKVVAPDLYIAVGISGAIQHLAGMKDSKVIVAINKDEEAPIFQVADYGLVGDLFVILPELEKAL----MLQRIFKTDRFREFLLAG-TLLLAAGLAGCNT-ADLN--PSETLTQGYVLDEKALEFVPVGSSREQVQLSLGSPSTTATFDNEVYYYISQKRHRTVAFMNPRVTERRILAVYFDKDGKVASISNYGLQDGKVFDFISRTTPTGGKDLSFLGQMLAGVGKAP-SL-GAGGPPSQ------MADLFSVNDDPASDRSRPLADRLRPRTLKDVTGQEHLTGTEGALTRMIDAGSIGSMIFWGPPGTGKTTVARLLANETNLAFEQISAIFSGVADLKKVFDMARARKMSGRQTLLFVDEIHRFNRAQQDSFLPVMEDGTIVLVGATTENPSFELNAALLSRARVLVFHPHDETSLKALMERAESHEEKTLPLNEEARASLVRMADGDGRAILTLAEEVWRAARDGEVFDAERVQQIVQRRAPVYDKGQDGHYNLISALHKSVRGSDPDAALYYLCRMFDAGEDPLYIGRRMVRMASEDIGLADPQALVICNAAKDAYDYLGSPEGELALAQACVYLATAPKSNAVYTAYKAAMQTAKANGSLVPPKHILNAPTKMMKGEGYGKGYAYDHDQPDAFSGQNYFPEALGRQTFYDPPERGFEREIRKRIDYWKKLRQER-GEK-MRIEIVKRPQHSALFSALSPFIALFLTLIAGAILFSILGKNPISALYSYFVEPLTEVWSIHELLVKAAPLILIAVGLSVCFLSNNWNIGAEGQLIAGGIAGSILPVMFPDLQGWYVLPIMLLLGMAGGMAYATIPAFLKVRFNTNEILTSLMLVYVAQLFLDWLVRGHWRNPQGYNFPETVQFNPSAILPEIWMASGRAHWGFILALVAAVLVWFMLSRTLNGYEIKVLGRSSRAGRFAGFSAGRLTFFAFLISGALAGLAGIAEVSGAVGQLRTSISPGYGFTAIIVAFLGRLNPLGIIAAGLVLALSYLGGEAAQISLGISEKSARIFQGIILFFVLASDTLIYYRIRIVSRA-----GDAVAKGA---MRYLHTMVRVRDVNESLDFYCNKFGLEEIRRMENEKGRFTLIFLAAPEDKGRSLAERAPELELTYNWDPEEYAGGRNFGHLAYVVDNIYDTCQNLQDKGVVINRPPRDGNMAFVRSPDGISIELLQKGSPLPPQEPWLSMPNTGTWMTTYDYDLFVIGGGSGGVRAGRLAGAMGKKVALAEEYRMGGTCVIRGCVPKKLFVYASQFPEHFEASAGYGWSVGETKFDWPTLIANKDREIARLEGLYRSGLDNSNVEIIDSRAILIDDHTIEILKTGKRVTAGQILIATGGHPNPHVALPGHELCISSNEAFHLKELPKAIVIEGGGYIAVEFANIFHGLGVETTLVYRGKEILSRFDHDLRQLLHASMVEKGIRVRCVEVIKEVKRQDSGRLAIQLSSGGELVADQVMLAIGRVPNTKSLGLEEARVEVDEIGAIKVDEYSRTSKANIWAVGDVTNRVQLTPVAIHEAMCFLETAFKNNPTKPDHRQIPTAVFSQPEIGTVGLNEEDAAKEFKELEIYRALFRPMRNTLSGAKDKMLTKLIVDSASRRVVGAHILGPDAGEMAQLLGIPIKAGCTKDDFDRTMAVHPTASEELVTMYKPNYRIVNGERID-----MPSAKDGQSP-AVKTANKRGVARLAAVQALYQMDVAGTGVLEVVAEYEAHRLGKEVDGTQYLDADPQWFRAIVSGVVADQTSLDPMIRQALMEDWPLSRLDSTLRAILRAGIWELKDRVDVPTAVIVSEYVDIAKAFYTDEEPKLVNAVLDRLAFEIRGESKGVKGPRR---------MAEKQDYIVKDLELAAWGRKEIEIAETEMPGLMASREEFGSSQPLKGARITGSLHMTIQTAVLIETLQALGAKVRWASCNIFSTQDHAAAAIAATGTPVFAVKGETLEEYWTYTDQIFQWPDGQPSNMILDDGGDATMYILIGARAEAGEDVLSNPGSEEEEILFAQIKKRMQETPGFFTRQRDAIKGVTEETTTGVNRLYQLQKKGLLPFPAINVNDSVTKSKFDNKYGCKESLVDGIRRATDVMMAGKVAVVCGYGDVGKGSAQSLNGAGARVKVTEVDPICALQAAMDGFEVVTLEDAAPTADIIITTTGNKDVITIDTMRKMKDMVIVGNIGHFDNEIQVVALRNLKWNNIKPQVDMITFPDGKRLLLLSEGRLLNLGNATGHPSFVMSASFTNQVLAQIELYSRGEQYKNEVYVLPKHLDEKVARLHLDKLGAKLSVLSEEQAAYIGVTPSGPFKSDHYRYMADGLIDILPSLNDGSAAGGPLYVKLQRLIENAVRDGMLHPGDALPPERELAAMADISRVTVRKAVQGLVNSGLLVQRHGSGTFVAPRSERVEQSLSHLTSFTEDMARRGMIVRSTWLDRGIYAPSPEEMVTLGLSSGEKVARISRLRVANETPMAIERAALSIHVLPNPELVTLSLYAVLAESGNRPVRAIQRISAAILKENDAKLLQVPAGSASLNIERISYLESGKVIEFTKSIYRADAYEFVAELKL----GDSSEISGAVLMIEAAILTVFPFAMAFAAVSDLLSMTIQNRVSLILIISFAVLAPLTGMPWELYGMHFVAGAAVLATTFALFATGTMGGGDAKLMSATAVWLGWNLGLVEYLLTMSMLGGLLTIAILRYRSSQFAVAYTDRFEFMRRLARKDVGVPYGIALGMAGLLTFPGSPLCQWVINRLAQIMRKLLIGLAGAVLASTLTFAYAQDKE-VNIAVSIPAADHGWTGGVVYHAERAAKLLEAAHPGLKITIKTSPDGASQANALEDLTTQGIDALVTLPHNSDELTDPIRAVKEKGIFVTVVDRALSDPSIQDLYVAGNNPELGRVAGEYLKNTM-KTGDVVVIRGLPIVIDEERQKGFDDAIAGSGIKVLDKQFGNWSRDDAFKIMQDYLTKFPKIDAVWCQDDDMAVGVLEAIDQAKRTDIKLVIGGAGMKDMIKRVADGDQLTPINVLYPPSMVATAMELTVANFYDQVPVRGKYILDATLVTKDNAKEFYFPDSPF----------------MSK-TKKFRTKPVLALLAVSL-LAGCAQ-RVMT-GSIPDDYRTNHPIVISEKEQVADIPVGHADSKLSLTQRSIVQNTIANYRANGSGVIHILLPTGSPNERAATRLRKDVAATLRRGGVKPFNISSEPYPAG-GSESPPIRLVYSAITASTHPCGQWPKDLLETADNRHYANFGCASQTNLAAQIANPADLLGPRATSPIDAERRGVVIDDYRNAET-----PKPVTTQEVNYDFN---MTDKKFTIDVVSDVVCPWCFLGRKRLEKALALNPDLDVSVNWRPFQLDPTIPRQGKDRNRYMQEKFGSSDRIFEIHQQLIELGKEEDIEFDFEAIEVSPNTLDAHRLIRWASQANPNVQDAVVGILFSYYFEQGKNIGDHQVLLEAADAAGMDVAIVASLLPTDADAVGVQQEIDTANQIGVRGVPCFILDQKYAVMGAQSADALADAIRQTADGFEPRPV--------------------MSLPPLKAKIISGP-EGGAAWNGLGD--DSLSGPSQTADWFSHWHSNANADCLVAALYAVDKPVFILPLEVVKKGPVRVAIYAGGPHANCNFPALSRTQ--KFGRNELANLFDDLHKVRPDIDLVWLSRQLDELGGAKNPLLQLPARENANVSLAITLDRNFDTVLGRNNAKRKRKNHRQHTRRFEEAGGYRIVTATTVSETEAMLSNYFVWKADRVAKAGIKNTYEPAGIKGFFHQLFAAETHAAAPRFQLKALEVAGTYRAVLGKSHAKGQTFIDFIGIADDELASASPGEFLFFEDIQDSCNTDLSIYSFGIGDEPYKRSWCDIEIPTYDTNISLTTKGRMYAGYLAGRERLVQKVKQNDVVWARVKKVRSRLFGKA----MEAVMQNSVHKNLLSELEFARLGAGQVAYMRKVKTDDLAHSFPALPPMTPGIELWALFAANGDPIVLSDERDNVLVGAQEHELRTVMLH---MNTQADHQDIAIVGAGIIGIAIAACLSESGRKVLVVDRQGICEGTSSGNAGALAFSDILPLASKGILAKVPGWLMDPLGPFTIRPSYLPKLTPWLYRFWRASRADMLERTIEAQGAMMRLAEPEMLGLMQRAGIRDMIREDGSLELYESEEELNAALPGWTARRRAGIAFEHLRGEQLAACQPGLAPRFVAGTFVPGWKTVSDPQQVGKRLWNYAERLGARFVRARVDLVAAGENGATAHLCDGRTIKASKLIVAAGAWSHRLAKGLGDTIPLETERGYNTTLPAGAFDLKRQLIFSGHGFVVTPLETGVRIGGAVELGGLDLPPNYRRSEAMLIKAQKFMPGLETAAGRQWMGYRPSLPDSLPAIGYAGTSKSVLYAFGHGHLGLTQAAATGRLITDLVSGKEPAISIEPFNPHRF------------MAKIVESATGALALTFDDVLLQPGHSEVMPGQTDIRTRIAPDIELNLPLLSAAMDTVTESRLAIAMAQAGGMGVIHRNLSPELQAEEVRQVKKFESGMVVNPVTIGPDATLADAQALMRSHGISGIPVVENAGEGSRKPGRLVGILTNRDVRFASDPAQKIYELMTRENLITVRE-NVQQDEAKRLLHQHRIEKLLVVDDKGYCVGLITVKDMEKSQLNPNAAKDAQGRLRVAAASSVGEDGFERAERLIAAGVDVLVIDTAHGHSQRVLDAVTRAKKMSNAVRIIAGNVATAAGTQALIDAGADAVKVGIGPGSICTTRIVAGVGVPQLSAIMSAVEAAHKSDIPVIADGGIKYSGDFAKALAAGAAAAMAGSALAGTEESPGEVYLYQGRSFKAYRGMGSVGAMARGSADRYFQAEVRDELKLVPEGIEGQVAYKGPVSGVLHQLAGGLRASMGYVGAKNLEEFREKATFVRITNAGLRESHTHDVTITRESPNYPGGNMTSRSFLTTILMAVAIAGAVSACGRKGPLEPPPASVTS-TPGEPNQATPPAPDKRFILDPLI-MYRAPVEEIAHTLKIVAGLDDAIAQGRLGDLSDDLVDAILEEAGKFASTRVAPLLQIGDEHGTPLKDADVTMPPGWKEVYREWISGGWNALTGKEEFGGQSLPMMLAIATFEMWNSGSMAFGIGPTLTLGAIEALEAHATDELKQTFLPKLVSGEWMGTMNLTEPQAGSDVGALRTRAERAEDGTYRIFGSKIFITYGEHDLTENIIHLVLARLPDAPAGTKGISLFLVPKFLVNEDGSLGARNDVFCGGIEHKMGIHGSPTCTMIYGDGFARAKKPGAVGWLVGEENRGLACMFTMMNNARLAVGIQGVAVAEAAYQKALAYAEERRQMRAP----GWT-GEGMSPIIEHPDVQRNLLTMKGLTAAARSIAYACAHAIDMAKSGTGDDARHWADRANLLTPVAKAFSTDVGVDVASIGIQVHGGMGFIEETGAAQLLRDARIAPIYEGTNGIQAIDLVQRKLPLGGGEHIKTYVAELRAVAKSVAASNSADFGETGQRLLASLEDLETTSLWLQQALAKGKISAAFAGATPYLRLFGLAAGATYLAKSALGSEQAGRTALARFFAENLLSETSALKDRVIHGAASLFGARSALAS-MATFSQKPADVTKKWILIDAEGLVVGRLASIVANILRGKHKATFTPHVDDGDNVIIINADKVAFTGKKLTDKVYYWHTGHPGGIKERTARQLLEGRFPERVVEKAVERMVPRGPLGRRQMKNLRVYAGSNHPHEAQSPEVLDVGALNAKNKRSA-MITNTLYPENQPYSEQMLEVSELHSIHLEQCGNPDGKPVIMIHGGPGGGINPIMRRLHDPERYRIILFDQRGCGKSTPHAELEENTTWDLVADMERIRNHLGIEKWQVFGGSWGSTLGLAYAQTHPAQVTELILRGIFMIRRFEIEWFYSNGASIIYPDRFEAYQEHIPEEERGDMIAAYYKRLTDPDPNVRMAAAKLWARWEGSALSLLPDPAREEAFGSDHFAIAFARIECHYFQNRGFFDTDDQLLRNAHLIRDIPGIIVHGRYDMCTPLLNAWHLKKVWPEADLRIVEDAGHAVSEPGIVHELVAATKRFAA----------------------MLSRRTLLVGMSVLALSGPASAEPILKRILNPFGGEPSQP-------AVVPDAEVKPIEVAEVRKP-------ANQTKAAKAKTKYGIDPKYMPQDVAFSGYKQGTIVIDPKAKFLYLVESPFSARRYGIAVGKEGLEFKGTATIQTKREWPRWIPTKEMIEREPAHYAKYENGMDGGPGNPLGARALYLSQGNKDTHVRIHGTIQPWTIGSSASNGCFRMVNDHVIDLYNRVSVGTEVIVLMGELMKGKRGLIMGVANSHSIAWGIAKELAENGAELAFTYQGEAFGKRVAPLAEQLGSKLLLPCDVEDIASVDAVFETLEQQWGTIDFVVHAIGFSDKSQLKGRYADVTTRDNFSRTMVISAYSFTEIAQRASKLMPEGGSILTLTYGGSTRVMPNYNVMGVAKAALEAMVRYLAADYGPEGIRVNAISAGPVRTLAGAGIGDARAMFSYQKRNAPLRRTVDIEDVGRSALYLLSNLSSGVTGEIHYVDSGYNIVSMPTLEELKKSDD-GKE--------------MPSKSIDHAFTARNLTSAATDPTHAGALSFMRRRYTKNLNGAEAVVWGIPFDAAVSNRPGARFGPQAIRRASAIFDNDPQYPFQRDLFADLAVVDYGDCLLDYGNHQKTPATIEREAAKIIKAGAFLLSLGGDHFVTWPILKAHAAKHGPVALVQFDAHQDTWYDDGKRIDHGSFVGRAAHDGVIDPAHSIQVGIRT-HAPDDCGLKIVYGYEVEEMSAADIAKQIIARTAGKKVYLTFDIDCLDPAFAPGTGTPVAGGPSSAKILSVLRHL-TKLNIVGADVVEVAPAYDHADVTAIAGATIAMYYLGILAEKKARRS-MGSKTLKTLLMATSILATAGFAHAADTTLTIESWRTDDLAIWQEKLIPAFEAKNPGIKVKFSPTPPTEYDAALGARFDAGSAGDIITCRPFDKSLEQFKRGNLASLNDLSGMKNFSDVAKAAWTTDDGKDTFCVPMASVIHGFIYNKDAFDKLGIAIPTTEAEFFAALDKIKADGTYIPMAMGTKDLWEAATMGYQNIGPTYWKGEEGRAKLIKGESKLTDPEWVEPYKVLLKWKDYLGDGFEAQTYPDSQNLFTLGRAAIYPAGSWEIGLFNTQAQFKMGAFPPPVKNAGDACYISDHNDIGVGLNSKSGHPEEAKKFLDWVASPEFADIYANALPGFFSLNSTAVKMQDPLAQEFVSWREKCKPTIRSTYQILSRGTPNLENETWVESANVINGTDTPEAAAKKLQDGLDSWFKPAKMTRMTPFSSPLLLGFDTMEKTLERIAKSGDGYPPYNIERIRGE-NGCEKLRITLAVAGFGNDDLEVTTEDNQLVIRGRQA--EEGEREYLHRGIAARQFQRIFVLADGMRVSVATLKNGLLSIDLDRPEPERLVKRINIAVQDMGKVTGFLEIDRQVGKYQPASDRIRHFREFTIPMSDSEVKKQAARCMDCGIPFCHGPTGCPIHNQIPDWNDLVYNDNWDEAIRNLHSTNNFPEFTGRICPAPCEEACTLNLEDVPVSIKTVEQAIADKAYEKGFIIPQPAASKTGKNVAIIGSGPAGLAAAQQLGRAGHEVHVYERESRPGGLLRYGIPDFKMEKHFIDRRVAQMEGEGVTFFCGVNVGIDKPVQELIDSYDAVLYCGGAETPRPAGIPGGEFEGVHDAMHYLVQQNRRIGRENIESVAWPSDPIVASGKHVVVVGGGDTASDCVGTAFRQGAVKVTQLDIRPQPPEKEDKLSVWPYWATKMRTSSSQAEGADREFQVATLEFIGENGTLTHVKCCEVDEARKPVAGTEFFIKADLAFIAIGFSGPFENSIVKELSGSLDMRADRRGNRSVAADDRNYRTSVDKLYTAGDVRRGQSLVVWAIREGRQAAQAIDTALMGTSVLPR-MTQLIYKIAPRALWQAAEEKGVFDGASVDHADGYIHFSTAVQAMETAAKHFADQDDLLLIAVETDRLGDALKYEVSRGGDLFPHLYASLPLAAVRWVRPLPLGSDGKHVFPEMTA-------------------MAG--NARAETKMDKISLAATAPTHVSKVGIKAKDADTLSKYYQDVVGLREISRKGQSVILGAGATPLLEIEQASAIRADDPHSAGLYHTAFLLPARADLARWARRAIDRRTAITGASDHLVSEAIYLTDPEGNGIEIYADRPHESWKWNGALVQMATEALDVGNLLGEPGGDVP-WTAAPDGTMVGHLHLRVGNAKEAESWWQNELGLQTVAGYGGSAVFMSTGGYHHHVAANSWQSRGAGRRDNDRSGLAWAEFSSADAKGERELVDPWGNVIRIVPAK--G---------MSFALSRNVAAIFLAGIVVALPP-ALP---ARAETASQAP--------------------------------AAASTTDKDDVSSFNLENGLEVVVIPDHRAPVVTHMLWYHVGSADEEPGKSGIAHFFEHLMFKATKTYPAGEFSRKVAEIGGQENAFTSYDYTAFYQQVAPQALEMVMTYEADRMENLILNDDVVKTERDVVLEERRSRVDGDPGALLAEEVNATLYQNHPYRMPVIGWLHEMQQLNLKDALAFYEKYYTPNNATLVVSGDVDLETVKALAEKTYGKLPRRAEPGNRVRPQEPEQNTKRTVSMADPRVSQPSFQKMWLVPSYTSAKPGEAEALDLLSEILGGSTRSRIYQALVVRDGSAASAGAYYQGGSLDDGSFGIYGSPRGSATLEQVEAGVDAQIAKIIKDGVTADELEKARNRFLKSMIFARDSQSSMARIYGSTLSTGQTIKDIQEWPDRIRAVKVEDIQDVAKRYLVDSRSVTSYLMPEDGKPDQKVEEE-TPASEET-QQPEEPAQ------GVVQMTSSNDNGKG----GKRSFEKRGFDK--KGGDAGRSAHKGPRKSDA------GA--PAKRSF---KSSHVDVPVEEGERIAKRLARAGIASRREAETMIAAGRVSVNGKRLESPAINVMRTDRIEVDGKPLPEKERTRLWLYHKPAGLVTTNRDPEGRQTVFESLPRDMPRVLSVGRLDINTEGLLLLTNDGGLSRALELPSTGWLRRYRVRAHGSVTQEKLNTLKDGIAVDGVFYGAIEATLEREQGSNVWIMVGLREGKNREVKNVLGALGLSVNRLIRISYGPFQLGDLPEGAVREMNGRTLRDQLGERLIEESGADFDAPIINVFSNDAVKGEA-VQPAE--ARERPVR-GSSEWISSAPV-----AKSRGPRKSKDEKREETLSRWTTDRNDRPAAA---RGARDDRPARA---------PRDDDRKPRA----PRDAAPKAK-RGKAEEEISERKSRSANVWMAPGARPVGKPRAKA-EAERGERTRSDSPRTDKPRRSGEGSSRP--------AGDRPY--TGRPAGERS------SAGYGGDKPR-SYST----RPPRSED--ERSERPKRSFGSDREDGS-----RPARPPRVGKRERAALKEQESGGA---------KP-FQSERPNRSE--RPYG-ASGKPSRGPGRQDGR-SGDR-----PYGG--PK------GA------PRGSS-G--GAGRPPRGNKPKSMPWSNQNGGGGPWGGGGNNS-GGGGPWGQKPQG--G-G-GG--NPPDLEDILRRGQDRLRQALP---GGSG-GSPAIWGLVGLALVAFWLFQSIYTVQPDERAVELRFGKPKPDISEPGLHFHLWPIESYEKVEIVEKQKNIGGQGARGAKEGLMLSGDQNIVDVQFSVLYRVSDPVAYLFHVENPENLVQQVSESAMREIVGRSPAQDIFRDNRAGIANDVRGIIQKTLDDYGAGVAINALSIEDAAPPREVADAFDEVQRAEQDEDRFVEESNQYSNQKLGQARGEGAQIREDAAAYKNRIVQEAAGEAQRFTSVYDEYVKAPEVTRKRLFLETMERVLKDSNKVIIDQ-SGQGVVPYLPLQEISR-PRPATV---------------------EGTKQ--------------MQTTKP---PHGELVEPRT----TFSLARLSIVLGLAGSIVVVDTAAA--R--TILDMLFGP-RQVYREPTDRLIDDG-RRRYR----APTKNR-AIKRASRPRPTAP-NRAPSGP--ASVAQPAIPEKLPDAKTVLVVGDFMAGNLAEGLDAAFVDSPGVRVVDKADGSSGFVRTDHHDWPASIGGLIEAEKPAVVVIMVGANDRQQM-G------GQTLLTDEWTREYQTRINAFLEAVKKTGNPVVWVGQPAFKSKNITNDVLAFNELYRNATEKADGKFVDVWDGFVDQSGNFTLSGFDISGQTARLRNNDGIGLTMAGKRKLAFYVEKPLRQMLGNATSPEIAAIKPGAPVQALPGTPQPPVKVDRLAPISLNDPELDGGTDLLGGAPRPVSATEKSARDRLVIDGIAPDSQPGRVNDFSWPKKPEET----------PTTGAVRMFTGIVTDIGKVGQVKPLNEGVLLRVETNYDPETIEIGASISCSGVCLTVTTLPEKGSNARWFEVEAWEEALRLTSIAGWKSGTRINLERSLKLGDEMGGHLVSGHIDAMAEIVKREDEGDAVRFTLRVPEHLARFIAQKGSVALDGTSLTVNRVTGNEFDVLLIRHSLEVTTWGDRKVGDKVNIEIDQMARYAARLAEYPKSAMIALPFLRS-ASPPM--QGERLYLRPPQLSDYRAWATLRAQSREFLAPWEPLWAVDDLERGAFRHRVRHYDEEAAAGTAHPFFLFRNPDSRLLGGITLGNIRRGVGQNGMIGYWIGEPFAGQGYMSEALDLLIPYAFTKVRLHRLEAACIPSNERSIRLLEKAGFQREGLLRSYLKINGMWQDHLLFSLIEDDGR-----RQLR-----MNFKVAPSARALFLCTALLSGMVAPLHAQ-----------------------------TATDTTKQLPETHEQMQLSFAPLVKQTAGAVVNVYAAQAVRTRSPFAGDPFFEQFFGG-QF-NGPPRVQSSLGSGVIADASGIIVTNNHVIRNADSVKVALSDGREFESKVLLKDESTDLAILKIDAGEPLPALQLADSDKVEVGDLVLAIGNPFGVGQTVTSGIVSAQARTRVGISDFDFFIQTDAAINPGNSGGALIDLSGKLIGINTAIFSRSGGSIGIGFAIPSNMVRAVVETAKGGGDTFERPYIGATFQNVTPDVAEGLGMKQPYGALVTNIGKGGPAEKGGLLVGDVVLSVDGVRIENPDGLGYRLSTAGIGKTVQLQVLSRSKEKAITVVLEKPGPDSLDNQMLIQGRNPLSGAHVLMLTPSSAARLNLPSDTE-GVAVDKVFPNTPAARIGLQPGDIVRGINGAEIETMADLTKAMSSKPVL-WRFDFERGGDIIRQIIR-MMAAMIERALNSASAPLRNGAFIAVVGPSGAGKDTIIDYARAALGDQPGYHFVRRVVTRPSSADAEDHDTLSEEQFLAAKQAGAFCHSWEAHGLHYGLPRSVDDEIERGAVAIANVSRGVLPALRQAYSNLLVVHITASLDVLAQRLASRGRENADEIRRRLIRAAPNACDPVDAVMIDNSGDVSNAGDEFIAVLRKSAAFAAISEQIMAEAHQKNHDYHIIDPSPWPFLGSVGAFVLAIGGIAFMRYNSGGELVLFNANLTSPWIFAIGLVIILYTMYGWWSDTIKESKEGHHTRVVSLHLRYGMIMFIASEVMFFVAWFWAFFDASLFPGEVQQVARTAFTGGVWPPKGIEVLDPLHFPLYNTIILLLSGTTVTWAHHALIHNDRKGLITGLTLTVLLGLLFSFVQGYEYVHAPFAFKDSIYGATFFMATGFHGFHVIIGTIFLAVCLLRSLRGDFTPQKHFGFEAAAWYWHFVDVVWLFLFFSIYVWAGWGAPIAVE-MSQDSFIP-------------------QKIVPVLVPMPAERPYSYAVPDGMEVKAGSIVRVPLGPREVAGIVTDGSTDSIDAKKLRPISELFDCPPVDGDMLRFMRWAADYTISPPGMVARMVLRVPAAFDPEPPVPGLRFAGREPDRMTGARARVLELAEDGMAWTRTGLAHAAGVSATVVDGLVTQGVFEHVMIPPRPVVAAPDVDYAKAALSGEQEGAAAQLVDAVSAGGFSVSLLDGVTGSGKTEVYFEAVAKAIEKGKQVLILLPEIALTQQFLDRFHDRFGSKPGEWHSDLAPRMRERVWRQVMEGQIRVVAGARSALFLPFQELGLIVVDEEHDPAYKQEDRVYYNARDMAVVRGHIGNFPVVLASATPSIESQVNAGQGRYNRIKLTGRYAEAAMPDLKSIDMRRSPPRPGGFLSPALVEAINRTLHRQEQSLLFLNRRGYAPLTLCRVCGHRFQCPDCSSWLVEHRFRGQLVCHQCGHHEARPEACPSCGTLDHLVACGPGVERIAEEAEKTFPDARIIVLSSDLM-GVKRLRLELDAIARGEADIIIGTQLVAKGHNFPNMTLVGVVDADLGLANGDPRAAERTFQLLSQVTGRAGRTGKKSLGLLQTYQPDHPVMRAIVSGDSQAFYEREIEERERSGLPPFGRLGSLIISAATRVEAENHARALRRAAPVSPQISVLGPAEAPLALVRGRHRFRLLIHGTRKADIQGFIRAMLAAGPKERGSVRVQVDIDPQSFLMSQTAIFWPMIALTFLICCVYAVLFLRRQQALAIGQATPHDFKLPLKEPEASVTAIRNLMNLYELPTLFYVVCLSLYAVNGATLLAVILAWLFVAARAVHTIVHITSNRLRLRQPLFLVGFILNGALWVLLALHLALPPVA-----MLLETNEKTVADEARALQARYGNLEPLDVIELAIDHLFNEEIAVVSSFGAESSVLLHMIAQVDRATPVLFLETGKHFPATLRYRDQLVGDLGLTDVHDIHPLKQSLKDEDPFGALSMTNKDRCCYIRKVEPMARAVAPYRAWMTGRKQFQASTRTALPVFESVGSRVRINPLARWTSADLKDYMAAHELPPHPLVAEGYRSIGCMPCTQPVKDGEDDRAGRWAGTDKTECGIHLTGLTDQLSNISLTDSNLMNKWKDVKKVVLAYSGGLDTSIILKWLQTEIGAEVVTFTADLGQGEELEPARKKAEMLGIK--EIYIEDVREEFVRDFVFPMFRANAVYEGVYLLGTSIARPLISKHLVEIAARTGADAIAHGATGKGNDQVRFELSAYALNPDIKVIAPWRDWTFKSRTDLLEFAREHQIPIAKDKLGEAPFSVDANLLHSSSEGKVLEDPWQEAPEYVHQRTISPMDAPDIVTEIEVSFEKGDAVAINGKPLSPATLLKTLNDLGRDNGIGRLDLVENRFVGMKSRGVYETPGGTILLAAHRAMESITLDRGAAHLKDEFMPRYAELIYNGFWFSPEREMLQAMIDKSQEDVEGSVRLKLYKGNVMVSGRKSKKSLYSDALVTFEDDRGAYDQKDAAGFIRLNALRLRTLAARKRSSMSRIAYVNGRYVPHSEAGIHIEDRGYQFADGVYEVCEIARGNIMDMTRHLDRLGRSLGELKILWPMDRKALIAVIKEVVRRNRVHNGLVYLQVTRGVAKRDHVFPSVDTPPSIVITAKRTDPHASAARAAKGIKVITVPENRWERVDIKSIGLLPNVLARQEAKEQGAQEAWFVDPDGTVKEGAATNAWIVTKDGVLVTRPAESGILRGITRTTIFDVARKMGLKIEERGFSVDEARKAKEVFMTAATTVVMPIVAIDGESVANGHPGATTLSLREAFFDIAEKTPSC--MRKYSVFALAREALRAHKGWEQQWTSPEPKAEYDVIIVGAGGHGLATAYYLAKEHGITNVAVLEKGWLGGGNTGRNTTIIRSNYLYDESAGIYEHAVKLWEGLSQDLNYNVMYSARGVMMLAHNIHDVQVFKRHIHANRLNGIDNEWLTPEQVKEFCPPINLSADARYPIIGAALQRRGGTARHDAVAWGYARAAADRGVHIIQNCEVTGIRRAPNGAVIGVDTNRGPINAKKVGVVAAGHTSVIMNMAGVRMPLESYPLQALVSEPVKPLIPCVIMSNTVHAYISQSDKGEMVIGAGTDQYTSYSQTGGLHIINHTLDAICEMFPVLTRLKMLRSWGGIVDVTPDRSPILAKTPVPGLYVNCGWGTGGFKATPGSGNVFAHTIARDEPHRINAPFTIERFTTGRLIDEAAAAAVAH-MSDALDFNDLKRRMDGAIQALRHELNGLRTGRASASLLEPITVEAYGSQMPLNQVANITVPESRMLAVSVWDKSMVGKVERAIRD-SGLGLNPITDGNNLRIPLPELNEQRRKELVKIAHQYVEHAKVAARHVRRDGMDELKKLEKDGQISQDDNRVLSEKVQKLTDDTIAEMDKVVAVKEAEIMQVMTSEAPPFWWEKPDWRSRALSPLSKIYGTVAGRRIRRAKSPAVPLPVLCVGNFTVGGAGKTPTSIAFAKAATSAGLKPGIVSRGYGGVFSGQHRVDPDYDSARHVGDEPMLLARHAPVVVSADRLAGALALQEAGCDFIIMDDGFQSARLHFDYALMVVDAGRGIGNGHVIPGGPLRAPLTDQLVRTDALLKIGTANGADQLVRSAARAAKPIYEAVLNPKSTADILGKQLLAFAGIGDPSKFFRTLQEAGAAVAQSRSFPDHHRYTDEELNDLVNLAEKDGLTLITTAKDHVRLLDGGTFAKEFAARTLVLEIELAFSHPQTASRIIRETRDRARTRAINCS-----------------------------------------MPKNFETPLLDRVNAPADMRQLPESDLSQLALELRTELIDAVSTTGGHLGAGLGVVELTVALHHVFDTPHDRIIWDVGHQAYPHKILTGRRDRIRTLRQEGGLSGFTKRSESEYDPFGAAHSSTSISAGLGMAIASDLSGTRRNVISIIGDGAMSAGMAYEAMNNAGALDARLIVILNDNDMSIAPPTGAMSAYLARLVSGRAYRSFRETAKQLAKKLPKFLQEKARLSEEYARGFWTGGTMFEELGFYYVGPIDGHNLDHLLPVLKNVRDTMDGPVLIHVVTQKGKGYAPAEAAADKYHGVNKFDVITGAQAKPPANAPSYTKVFATSLIEEARHDDRIVAVTAAMPGGTGLDLFGDVFPSRTFDVGIAEQHAVTFAAGLASEGFKPFAAIYSTFLQRGYDQVVHDVSLQNLPVRFPIDRAGLVGADGATHAGSFDTGFLAALPGFVVMAASDEAELRHMVRTAAEYDEGPISFRYPRGDGVGVDMPERGQVLQIGKGRIVREGSKIALLSFGTRLHECLAAAEELDAAGLFTTVVDARFAKPLDEELVRRLAREHEVLITVEEGAIGGFASHVLHFLSRDGLLDNGLRVRTLTLPDLYLDHAKPEAMYARSGLDSAGIVQAVFGALGREHIVSPARA------------------------MPKKVTAPQAKAFSVHLLTASGSFLAFLSVVAASEESWTAMFWWLGLALFVDGIDGPIARKLEVKYVLPNWSGELLDNIIDYMTYVLIPAFALYQRGFMGEGLSFLAAAIIVVSSAIYYADTGMKTKENFFKGFPVVWNMIVFTLFIVRPGEWVAFVIVLASAIVSFLPVYFLHPVRVQRLRPLNLAIFFLWCGFGAISLFQELDSPLWVRIGISVTGIYLFCIGAVMQAFPNLGRTEKV-------------MSVKVLTANRLTDGEAVWLGANGEWLEHIDGALIARHAEAVTALEEAGKAAIKSNLVIDVNVIDVEERGTNLYPLRLRERIRQLGPTIRLDLGKQAEKPKATAA---MIENSSATAK-ATRRKGGALKRFWRSVRGPLANSPMVRTMIVWLITQFFRLVHLTNPRLAGSSDLEDMKRGGEPFIAVAWHGQHLMAPFLMPRGVRFVAMFSKSKDAELNARVAERFGVEIVRGSGGRERARGTEKGGARALLVLKKALNEGKTAAMIADIPHGTPRDSGFGVILLAKLSGRPILPICYLTTRRKVLEKSWDKTTIPLPFGKSGLIVGDPIFVPENADDAMLEAKRIELTNSLNENTRRVHQLVDGLA-----MSVDISTVKRVAHLARIAVSEDDATRMTGQLNAILGFVEQLNEVNVEGVEPMT----SVTPMAMKKRQDQVNDGDKAADIVANAPIHEENFFVVPKVVEMPANSKNRSDLELIREAAEGAGRIALSYFKQAPQIWMKTGNSPVTEADLAADKYLKDMLLTARPDYGWISEETHDERVA-SRRQRFFVVDPIDGTRAFIEGSDVWCVSVAVIENGRPVAGVLDCPARGEILTALPGLGAEQNGNAIHV--QAETRPFKVAGPGAFLKQLPEHFLQSIEHAPYVASLAYRIAMVARGDIAGTFVRPNSHDWDLAAADLILSEAGGQVLTADGKALTYGVRNTNKGDYRHGALVAASGLLMDNMLDVVAETAFGMTAKTGLMQGKRGLIMGVANNRSIAWGIAKAARDAGAELAFTYQGDALKKRVEPLAQELGAFLCGHCDVSDASTIDACFAALEKEWGKLDFVVHAIGFSDKDELTGRYVDTSEGNFTKTMLISVYSLTAVAKRAEKLMTDGGSILTLTYYGAEKVMPNYNVMGVAKAALEASVKYLAVDLGPDNIRVNAISAGPIKTLAASGIGDFRYILKWNEYNAPLRRTVTIEEVGDAGLYFLSDLSRSVTGEIHHADSGYHVVGMKAVDAPDISVVKD--MADIPVEDLSEAEAAEELGRLAAEIASHDYRYNTEDAPIISDAAYDALRRRNLAIEQRFPHLKREDSPSNKVGAVASEKFGKVTHSIPMLSLDNAFHDSDVEDFVGRIRRFLKLGPDAKIGITAEPKIDGLSLSLRYELGRLVTAATRGDGSTGENVTLNARTVASIPNVLNGKPPEVLEVRGEVYMSHADFAALNERQAKEGKQIFANPRNAAAGSLRQLDATITASRPLQFFAYAWGEVSEMPAATQMGMVDAFRSYGFRVNPLMQRFVSVEGLVKHYHQIEEQRALLGYDIDGVVYKVDDLALQQRLGFVSRSPRWAIAHKFPAEQAMTILKGIDIQVGRTGALTPVARLEPVTVGGVVVTNVTLHNEDYIKGIGQNGQPIREGRDLRVGDTVVVQRAGDVIPQIVDIVAD-KPRGPSPYVFPDTCPACGSHAVREEGEAVRRCAGGLICPAQAVERIRHFVSRNAFDIEGLGEKQVEFFFKSEDPALHIGTPADIFTLKKRQAASLAKLENIDGFGATSVKKLYDAIDDRREVTLSRFIYGLGIRHVGEVNAKRLAKAYRSYKALANAATAAVP---PKDRADRGNDAWRELNEVEGIGSIVAEALVDFYAEEHNREALAALLAEVTPLDEEVKTVSGSPVVGKTIVFTGSLERMSRDEAKAMAERYGAKTAGSVSKKTDLVVAGPGAGSKLAQAQSLGIEVIDENAWFTLVGA--MTLIKPEVQSDPKAMIAEAERALADIAKIKAGIGTVIFGQESVIERTLVALLAGGHALLVGVPGLAKTKLVETLGTVLGLDERRIQFTPDLMPSDILGSEVMEQDDNGKRYFRYVKGPVFAQLLMADEINRASPRTQSALLQAMQEYHVTIAGERHDLPQPFHVLATQNPLEQEGTYPLPEAQLDRFLMQIDILYPELEAERRVLLETTGISEATAETIIDAERLRDMQQLIRRMPVPEGVVDAILTLVRSARPGADNEIAKKFISWGPGPRASQALMLCSRARALYDGRLAPSVDDVKALAEPVLQHRMALTFAARADGMNVRDVIANLTKAAL-----MKLTTDDFTEKSEPLALFDEWLAEAAKAEPNDPNAVALATVDSDGLPDVRMVLLKGFDSQGFVFYTNFESQKGQEILGSMKAAMCFHWKSLRRQVRIRGPVEIVSKEEADAYYATRPRGSRIGAWASKQSRPLESRFALEKAVAEYTARYAIGEIPRPDYWSGFRIRPTSIEFWQDRPFRLHDRMVFSRDTPEGDWG-KTRLYP-MSALPLAFGAPAILLSLIILPLIWWLLRVTPPRPIREVFPPLKILAQLLKKEETPNKSPWWLTLLRLLLAALVILALSEPVWNPRPETLTGKEPVAIVLDNGWSSNEEWNARKETAERLIADAAQSGALIYILGTAEKANADIGPFEANAALERLRALTVRPIPVDRQAAFARLRTALANISGTRIAYLNDGVDTPQSAEALKSLEGTGIASMIWYEPSISSLVAIRQAVNNANGLTVRAVRPVNDRGQSGVTIGAFDEKGRRIAETGLVFTAGEATAEAVISAPYELRNDFHTLRVDGTAQAGGTYLIDDNNKRRRVALLSGSEADI--SQPLLSPLYYISRALEPFADLLRPRNAELVRAVPELLEQKPSVVIMADIGKLPAETEQILSDWINKGGTLVRFAGPRLAGNSELDPLLPVTLRRGERSLGGTLSWKESQPVAAFPENGPFGGLPTPQDVTVTRQVLAEPSADLYDKSWANLADGTPLVTGEARQRGQLVLFHVTPEATWSNLPISGSFVDMLHRIISLSNSSGPTSNDTRESGSAVLPPYLTLSAEGTLVPPNGDTKPLLIRSGTSPEVSFDNPPGFYGVEDAMMAFNLFKPDGEIKPLIRPDLTIPVMTARYAVDESVPLRGPLFALASLLLALDAIAILWLGGHLR-------RRF---RPTATAASLVILTLAAIAGFGPSPALAQQ-----------------------------NDSKPGDQFVLEAVNATHLAYVITGDRAVDDVSKAGLSGLSRALTDSTALEPGEPIGVNPATDELAFFPLIYWPIDAAARMPSPEAIAKIDAYMQQGGTVLFDTRDQDAAAINFENSTTPANQRLRDILAGLNIPPLEPVPSDHVLTKSFYILQDFPGRYRGSPLWVQASLSTETRAD---RPVRAGDGVTPIMITGNDLAGAWAINADGSPILPTIPNDPMQRTYAYRTGINIVMYMLTGNYKSDQVHVPDILERLGN--------MMSTRVTIERMGAGGDGIASTSAGQAYVPFSLPGEVANVALEHGRATLMALLEPSSERIEPACRHFEECGGCALQHWQEEAYRNWKRSLVHDALAGRGLTFALDTLVPCAPQTRRRAVFAVRTSDHGAVLGFNRHLSHELIDISECPITVPEIVLRLDDLRDLVAVVSGGMKPFRLTVTATASGLDIMVSGCAAPDAAKRQALTALVLKKDFARLSFEGEIIVEPRKPLVHFGKVPVFLPAGGFLQATADAEQAMSALVTGHLAKAKKTLDLFSGSGTFALRIAEKSAVHAVEGDQAAIAALDRSVRHVQGLKPVTVERRDLFRRPFLARELTPFTGLVFDPPRAGAEGQAIEIAKSSVPKVAAVSCNPVTLARDLSILVKGGYKIDRVVPIDQFLWSSHVEAVALLSKK-MNVLLIGAGGREHALAWKIAASPVLAKLYCIPGNPGIANVAECRPLDPANHQEIVAFCKEKSIDFVVVGPEAPLVAGIADDLRAAGISVFGPSKAAARLEGSKGFTKDLCARFNIPTGAYQRFNNAEAAKSYVREQGAPIVVKADGLAAGKGVVVAFEIQEALDAVDACFEGAFGGAGAEVVVEEYLDGEEASFFCLCDGKTALPFGTAQDHKRVGDGDTGANTGGMGAYSPAPVMTLDMVDRTVRELIEPTMRGMAEIGSPFSGVLFAGLMITRDGPKLIEYNTRFGDPETQVLMLRLKDDLLLLLKAAADGVLDQMSVRWNDDPALTVVMAARGYPASPEKGSVIKGVEAASNNDAVEIFHAGTAEKNGELVANGGRVLNVTASGKTVRQAQDAAYAAIAKIDWPGGFYRHDIGWRAIAREKGDALMARFDLQHIDSGHVGGSAQTDILFEMGIMYATGRDCAVDLVAAHKWFNIAAIKGSDRAAELRAELAQSMSKSDLATALREAREWMTSHMTDFSSTRQGMD---GDDYDPYRLSSPRFVLLSMAIFLAIVAFLAAILFRQIHGFFVTNPGLNSLIIGVLVVGILLGFGQVIRLFPEVRWVNSFR-QGNNDPNAR-PIMLAPMRALIGRRQSMALSTSAMRSMLDSIASRLDETRDISRYLIGLLVFLGLLGTFWGLLETIGSIGQTIQSLDPRAGDANSVLDSLKAGLQSPLRGMGTAFSSSLFGLSGSLILGFLDLQAGRAQNRFYTELENWLSSVTDLGSDLHALDAASSGTSDELKALSERLQNIQETGGSSQRTTAALASLAEGIQGLVKNMRTEQQMMRDWVEKQADEQKSIRQTLAKLGDAISRDKAN-----MGLIQTIIVVFGVIAIAYAAAHFKLLDERVGDGLSDFVFMIAAPLLLFRTMVTADFHGTAPWQLWIAYFAGVVVAWAASDLAIKMIFGRDNRAGVVAGVSGAFSNLVFLGMPLMLGVFGQEGFAILSLIVAIHMPIMMAASITLHEIAMRRDGVFAGEGHFGLVVLAFFKSLLRNPFVLGILAGWIWRLTGLQMPVIADSLIQTLGSVAGPVALFAMGMGLKKFGVTGHIAASLVVSAIKLFVMPAVVLAAAWFIGLPPFTAKIAVASAALPAGVNSYLIATRFGTGQALASNSMVMATALSAITMAFWISVVTHIFG-MFVTPAYAQAT-GAGGAPEMLMSILPFILIFVIMYFLIIRPQRTAAKKREEQLKNIRRGDTIITGGGFIAKVTKVYEDTGELDVEIGEGVKARVVRGMVADVRVKGEPVADNKKMVSKAPRSQFPKPPLDGLRKRLKESRE-GTNQQRGAFVRESFRLPRLAAREKAREWFEQYPKAAYWTEIESWYERPGDIIEFTMRRLPNADMAKAATPKTPAAAKA--APKAAAAKTA---AVKAEA-AAK------PAAKAAAPKAPAKAAPAAK---------AATPKATLKATGVAGRVSQVIGAVVDVAFDGGVLPQILNALETDNLGNRLVLEVAQHLGENTVRTIAMDSTEGLVRGQAVYDTGSPIMVPVGEETLGRIMNVIGEPVDEAGPLNTKLTRAIHQPAPEYVEQSTEAQILVTGIKVVDLLAPYARGGKIGLFGGAGVGKTVLIMELINNVAKAHGGYSVFAGVGERTREGNDLYHEMIESGVNKAGGGEGSKAALVYGQMNEPPGARARVALSGLTVAEHFRDQGQDVLFFVDNIFRFTQAGSEVSALLGRIPSAVGYQPTLATDMGQMQERITTTHKGSITSVQAIYVPADDLTDPAPATSFAHLDATTVLNRAISEKGIYPAVDPLDSTSRMLDPLIVGEEHYQTARQVQSILQRYKSLQDIIAILGMDELSEEDKIAVARARKIERFLSQPFFVAEVFTGSPGKLVDLADTIKGFKGLCNGDYDHLPEAAFYMVGSIDEAIEKAQRLAAEAA----------------MGKTEFIAMMAMLMAINALAIDIMLPGLQEIGASLGVASENHRQYVISAYLMGFAVSQLFYGPISDRFGRRVPMFTGLVVYVVSALACVFVPSFTSLLLFRLLQGIGSAATRVITISIVRDVYGGRMMAEVMSLIMMVFMIVPVIAPGTGQAVMLFGNWHLIFIFMAAIASAVLVWVYLRLPETLDPADVRPFTLKSVAQGFRIVLTNRVALCYTLANSFIFGALFGFINSAQQIYVGIYDLGVWFPVAFAGVAVTMAFSSFVNSRFVGRFGMRRLSHGALLAFLGINGFWLLLTLFGPHPTPFVLYMLLFALAMFQFGWIGANFNALAMEPLGHVAGTASSVLGFMSTAGGGAIGALIGQAYDGTTLPLVAGYFVLSLIGLIFVLIAERGRLFQPHNQPV-------MFAVIKTGGKQYRVAANDLLKVEKVAGNAGDIVEFAEVLMVGVGADATIGAPVVGGALVTAEVVEQGRARKVIAFKKRRRQNSKRTRGHRQELTTIRISEILTDGAKPSKKAAEKP-AKKEAAPKAE--AAAADAVADSGETKAAPKAKAAPKKAAAKAEKPAKGEMVEKKKGKTVTVEQIGSPIRRPAEQRATLIGLGLNKMHRTRTLEDTPSVRGMIAKVQHLVRVVDEAMDIRAAEISAILKEQIKNFGQEAEVSEVGQVLSVGDGIARVYGLDNVQAGEMVEFPGGTRGMALNLESDNVGVVIFGSDRDIKEGDTVKRTGAIVDVPVGKELLGRVVDALGNPIDGKGPIKATQRSRVDVKAPGIIPRKSVHEPMSTGLKAIDALIPVGRGQRELVIGDRQTGKTAIILDTFLNQKPAHDAGNEADKLYCVYVAIGQKRSTVAQFVKILEERGALEYSIVIAATASDPAPMQYIAPFSACAMGEYFRDNGMHALIGYDDLSKQAVAYRQMSLLLRRPPGREAYPGDVFYLHSRLLERSAKLNDDNGAGSLTALPIIETQANDVSAYIPTNVISITDGQIFLETNLFYQGIRPAVNVGLSVSRVGSAAQIKAMKQVAGSIKGELAQYREMAAFAQFGSDLDASTQRLLNRGARLTELLKQPQFSPLKTEEQVAVIFAGVNGYLDKLAVNQVGKFEHGLLTWLRTEHKDVLDAIAKEKQLSDDVKGKLKAAVDAFAKSFA--MISAGSTIGIIGGGQLGRMLAMAAARLGYRTVILEPQGDCPAAQVANEHIVASYDDPNALEQLAELSDVITYEFENVPVDSAEFLNENKAVFPPPEALKVSQDRVAEKRFLNKAGIQTAQWRIVENEGDLKAALLAFDGKGILKTRRFGYDGKGQIKFSGTKGENAKSGFAAIGSVPSILEAFVDFTHEISVIAARDAAGNIKVYDIAENVHKDGILATSTVPVSVSSTIAGLAADAARKLLEGLNYVGVVGMEFFVLANGTLLANEFAPRVHNSGHWTEAACAVSQFEQHVRAIAGLPLGDTRRHSDCVMENLIGDDIDRVPELLQESGAVVHLYGKTEARPGRKMGHVTRITKRHMSFKVAVVGATGNVGREMLTILEERGFPVSEVVALASRRSLGTEVSFGDKTLKVKALDTYDFSDTDICLMSAGGNISKEWSPKIGAQGCVVIDNSSAWRYDSNVPLIVPEVNPDAISGFTKKNIIANPNCSTAQLVVALKPLHDKAKIKRVVVSTYQSVSGAGKEGMDELFEQSRAVFVADPVTAKKFTKRIAFNVIPHIDVFMEDGYTKEEWKMVAETKKMLDPKIKLTATCVRVPVFIGHSEAVNIEFESPMSAEDAREILSNAPGCLVVDRAEDGGYITPYDAAGEDATYISRIREDITVENGLSLWVVSDNLRKGAALNTIQIAELLVARGLIQPKRIAA---------MNIFADFDARIKKALQALELQTTDGAEMDLSRVGVEPPRDPSHGDIATNAAMVLSKAVGQNPRELAVRIGEALKNDPDVGSVDVAGPGFINLRLKDSYWHGQLAGMLSAGLDYGRSKLGYGHRVNVEYVSANPTGPMHVGHCRGAVVGDALANLLKFAGYDVAKEYYINDAGAQIDVLASSVMLRYREALGEQIGDIPAGLYPGDYLVPVGQALAADYGTKLLEMPEDEALAIVKDRTIDAMMAMIRDDLASLNVHHDVFFSERTLHANNAKLIRSAINDLTLKGHVYKGKLPPPKGQLPEDWEDREQTLFRSTDVGDDIDRPLVKSDGQFTYFAADVAYFKDKYDRHFDEMIYVLGADHGGYIKRLESLARAVSGGTAKLTVLICQLVKLYRNGEPVRMSKRSGEFVTLRDVVEEVGRDPVRFMMLYRKNDAPLDFDFAKVTEHSKDNPVFYVQYASARCHSVFRQAMEQLGIGEEALAGSAQHFGLLTDESEIALIRKLAEYPRLIETAALHQEPHRLAFYLYDLASSLHTQWNKGTENPDLRFIKVNDPNLSKARLGLVQVVSKVLASGLSIIGADAPTEMRMGTEHKLHSFADGNELAEALASTVADRLGAACDERGKAVLAVSGGTTPARFFKALSVKKLPWERITVTLVDERFVPPSSDRSNQKLVTGMLLQNDAASANFVGLYNDAPDVEEGAKIAAADIAELGQPFDVVTLGMGGDGHTASFFPGGDRLADAISPNQRALVLPMRAEGAGEPRLTLTLPVIVKARFAALHIEGAGKKAVLGQALEGGETSDMPIRAVLRHEPIELEIFWAP-----MTEVANVSVSDAAAKRISAILKSEPEKTALRISVEGGGCSGFSYKYDLVDVQDEDDIVIEKLGAKVLIDSISLPYIGGSEIDFVDDLMGQSFQIRNPNATASCGCGTSFSIMENTTSQQLRGVSTMAAAMFFLPMMDAIAKWLSTVDNLPPATVTLSRFVVQTTLTLLIVLAITGTGSLTPVKLWGNLFRGFLMGIGSLCFFAAIKYMPLADAMAVFFVEPLMLTLLSALILKEQVGWRRWVAVAIGFVGTLIVIQPSWELFGWVSLLPLGTATLFAIYLILNRRYGDADTPLVMQLYAGVGGTVTVAVALLLGQLFDVSDMTFDVPLGGLSWFLLFTIGAIATGGHLLVVQASRLAPASFLAPFQYLEIVMAVIIGLVVFNEFPSASKWFGIAIIISSGAYVVWRE-GKNRQEP-----EPA-----YN-------M-----NTGLAHRA----------RMALLLVPIASAIAISGCASKDDDIDLNAYVDTIEPADVLYNQALANLNSGRLAEAAKKFDAVDRQHPYSEFARKSLVMGAFANYRAGKYDDALAMTKRYIALYPTQPEAAYAYYITGLCYYRQIPDVTREQSMSRRSIAAFSEVVERFPDSEYVEDSKAKIRFGRDQLAGKEMQVGRYYLERKEYLAAIKRFRVVVEQYSNTRHVEEALARLVEANFALGLTNEAQAAAAVLGQNYPDSQWYKDSYKLLQSGGLEPRESAGSWLSKAATAITGG-KDGVMVSLASIRTVLSASILVLMFGAGSANAGSACGGASWYALHSKTASGERMNPSALTAAHRTLPFGSKVKVTNQRNGKSIVVRINDRGPFIKGRVIDLSKGAANRLGFVGAGHTNICMARV---------------------------------MSLLNIYWKSLQYLGAEKRATVFICVVNIALALTMLFEPLLMGLVIQAIADKADIWLPLTQLAAVGLFHIGANVLVAREADRLAHRRKLAVLTESYNRVISMPLSWHHQRGTSNAMHTLLRAIETLFGLWLEFMRTHLATTVALVGLIPMALVQDWRLASVLAVLGVVYVLVSRFVTAKTKDGQAQVERHHITAFSHVTDSMGNISVLQSYNRIDEETRALQSYAKQLLAAQYPVLNWWALAAGINRMASTISILVVLALGAHFVMQGQMQVGQVVSFIGFAGLLIGRLDQLSAFVNQIFAARAKLEDFFDMEASAAYAVEPKTVHDIDNVSGDVEFEDVSFAFANSTQGLKKVSFKAKAGQTVAIVGPTGAGKTTLINLLQRVYDPKEGRILVDGIDTRTISRRSLRNAIATVFQDAGMFNRSIEDNIRIGRPKASAEDVLAAAEAADASDFILQKSGGYGTVVGERGSQLSGGERQRVAIARAILKDAPILVLDEATSALDVETEARVKVAVDKLCQNRTTFIIAHRLSTVRDADIVLFMDQGEVVESGSFDELARQNGRFTSLLKAGGLKLDD-----DTANVTPF-----PRKPEAA-MFNQHKVEIEWGGRPLILETGKIARQADGAVLATYGETVVLATVVSAKEPKPGQDFFPLTVNYQEKTYAAGKIPGGYFKREGRPSENETLVSRLIDRPIRPLFADGYKNDTQVVITVIQHDLENNPDIVSMIGASAALTLSGVPFMGPIGGARVGYIGGDYKLNPTIDEMVESSLDLVVAGTGDAVLMVESEAKELAEDVMLGAVMFGHKGFQPVLEAIIKLAEVAAKEPRDFATDDLSAVEAAVLKVAEADLREAYKITDKQQRYAAVDAAKAKVKAHFFPEGVEEPEFSAEEVATVFKSVQAKIVRWNILDTGSRIDGRDLKTVRPIVSEVGLLPRTHGSALFTRGETQAIVVATLGTGEDEQYVDSLTGMYKETFMLHYNFPPYSVGETGRMGSPGRREIGHGKLAWRAIHPVLPAKEQFPYTLRTVSEITESNGSSSMATVCGTSLALMDAGVPLLRPVAGIAMGLILEGEKFAVLSDILGDEDHLGDMDFKVAGTDGGITALQMDIKIDGITEEIMKVALAQAKDGRLHILGEMAKAITEGRSELGEFAPRIEVMNIPTDKIRDVIGSGGKVIREIVEKTGAKINIEDDGTVKIASSSAKEIEAARKWIHSIVAEPEVGEIYEGTVVKTADFGAFVNFFGPRDGLVHISQLASERVAKTSDVVKEGQKVWVKLMGFDERGKVRLSMKVVNQETGKEIVQEKKPAEEDAE-----------------MNDDETSVTP---HADNDDQPPRPAFGQLPPAAQRALKEAEERRKA-ETKEADRPREIGGRGGKDPARFGDWEIKGRTIDFMGRL----KSFLAGLVFLIVTAIPVLALDQAPMHLPIDAAPLTINTAKGDVPFRVEVADTDEERERGLMFRTDLKDNSAMLFVFDRTRLVTMWMENTPSALDMLFLDNNGRITAIRENAVPFSRDTISSGDPARFVVEVKAGTAQRLGLTIGDKVRHPAIERIGR-----MTLPVILSATDLGALLSSRICHDIISPVGAINNGLELLEEGGADEDAMNLIKSSARSASARLQFARIAFGAAGSAGVQIDTGDAQNVANEYMKGEKAELTWEGPRVLLPKNKVKLLLNLVLIANAALPRGGTLSVVLETPDTQPRFVLTSRGPMLRVPPKFLELHSGKVPEEPIDAHAVQPYYALLLAQESGMTISIHATPEEIVFTAA--------MKARVTVTLKNGVLDPQGKAIVGALGSLGFDGVGSVRQGKVFDVELDTKDRAKAEESLKAMCEKLLANTVIEDYSVAID---------MKDFGIEKAVPDQATVTGSVAPAQSVDMKGS--SDQTTVRNIVSALNFTQWGSKPVPWSNPETGSQGTITAIAEKKDESGLCREFQTSRESFDGVMLYKGETCMQNGGQWTLKSFAPM----------------------------------MQAINDTELDKSAAVDSAKRTISTEKDGLAALSRSLEGPLAEAFCKAIEKIAAVRGRIIVTGVGKSGHIGSKIAATFASTGTPAFFVHPSEANHGDLGMIATDDAILAISWSGDTAELKGIVSYSRRFRIPLIAVTAGENSALATAADVVLLMPRVTEACPHGLAPTTSTLMQLAMGDALAVALLEARGFTAHDFKTFHPGGSLGASLTHVREIMHRGDRLPLVPLGTLIPDAMKVLSEKRFGCIAVLNDDETLAGIITDGDLSRNLHRNLVTVKVEEIMTRAPKTVPPTMLASAALALLNEHNIGALIVTENNFPIGIVHFHDLLRIGAA---MAETSSLISGVAQRYAGSLFGLALEAKSLAEVEQNLDAFEALINGSADLKRLIQSPVFSSDDQVKAISAILNKAKIGGLVGNFLRVVAQNRRLFSVPAMIQSFRQIAANHRGEVTADVTSAHALTAAQETELKATLKSVAGKDVTINVTVDPSILGGLIVKLGSRQIDTSLRTKLSSLKLVLKEVGMLDIKWIRENPQALDAALAKRGNEPASSLLIELDERRRQHIAKLQEAQERRNAASKEIGKAMGAKDQATAERLKAEVAEIKLFLQAAEEEERQLDRALNDALSVLPNLPLESVPVGKDEAANVETRRVGALKNFAFEPKEHFEIGEKLGYMDFERAAKLSGSRFTVLKSQLARLERALGQFMLDLHTRVHGYIEVSPPLLVNDDVLYGTGQLPKFAEDLFQT----------------------------------------TDGRYLIPTAEVPLTNLVREEILDIRDLPIRMTALTACFRSEAGSAGRDTRGMLRQHQFMKVELVSITDAERSIEEHERMTSCAEEVLKRLDLSFRTMTLCTGDMGFGAQKTYDLEVWLPGQKAYREISSCSVCGDFQARRMNARYRPEGEKQTRFVHTLNGSGVAVGRALIAVVENYQNEDGSVTVPDVLVPYMGGLKRIEKAAMQAKDDEGLILLNLGNIRSFLNAGTVAAAFALTIPLSLPVLGLAEAAAQ----VGPSGLPLPRFVSLKPGRVNLRVGPGRDYAVTWLFLKSGLPVEIIQEYDNWRRIRDSEGTEGWVYQSLLSGKRTAMTAPWQRDKDGTMLNVYRSADDKSGIVAKVEPGVLGTLKACNGSFCRVSFSGASGWIRQPDIWGAYPDEKFDD-------MAADSIFLGASRKPDNSYSQAESLLLKFGNRHGLITGATGTGKTVSLQVLAEGFSEAGVPVFCADIKGDLSGIGAKGEEKDFLLKRAAEVKLDPYEFGASPVIFWDIFGEQGHPIRSTISEMGPLLLSRLMNLTEAQEGVLNIAFKIADEEGLLLLDMKDLQALLVNIAERAEEISARYGNVTKPSVGAIQRSLLVLEQQGGTKFFGEPALKIADIMRTDTNGRGLVSVLAADKLMMNPRLYSTFLLWLLSELFEELPEIGDPDKPRLVFFFDEAHLLFNEAPKALLTRVEQVVRLIRSKGVGVFFISQNPLDVPETVLAQLGNRVQHALRAYTPREQNAVKTAADTFRPNPDFNTFETITNLATGEALVSTLADKGVPSMVQRTLMRPPAGRIGPLTSDERQGLINASPVAGLYDEMVDRESAYELLQKKAEQTKTEAEA-KQTQEADSTS-GWSLPDIL-GG-GQPGPRGG-APRGRQTVAEAAMKSVVRSVGNSLGRALVRGILGSLSRR--MPVRYFPALFVVLWATGFIGARYAMPYMEPFLFLTARFLIAGAILGVWVVLAGNHWPSKRGAMHAIIAGCLIHGVYLGAVFWAIHNGLPAGMSALVVGLQPLITALIAGLALGEKIQPRHWAGLAVGFYGVAMVLWPKLSISADGITPATVSASILSVIAISAGTVWQKRFVGAIDLKAGTTLQYLGAAVLTGVFSLLFETHVVIWSGSLVFAMLWLVFVLSIGAVLLLMILINQGAVSKVASLFYLVPGVTALMAYALFGETLTLFQLFGMLIATLGVALST--GQRRSAALPSR----------------METTTRTA-----FIPPAPIPRTGPVGTLRLIRTIYRNPLELWGEPSYNEPWVSIDWSFAHTIIANDPGLIRHVLVDNAKNYKMAAVRQRILRPILRDGLLTAEGEVWRRSRKAMAPVFTPRHIHGFAEAMKRTTDSFADRYEGLEGTTDVSRDMTMLTYDILAETLFSGEIAGDPDQFSHEVDRLFETMGRVDPLDLLGAPSWLPRFTQIRGRRALAFFRQIVANTIEMRKVRMDKEGDKVPNDFLTLLLRAEGPDGLSRAEIEDNIITFIGAGHETTARALGWTLYLLAQAYWERDLIEAEIDAFMDQADVP-PPHEWLDALPLTRAAFEEAMRLYPPAPSINREPIEDDQYKDLVLPKGAAVLVMPWTVHRHRLYWDNPDAFMPSRFHPENRDRIDRFQYLPFGAGPRVCIGASFALQEAIIALATLLHRYRFDMAGSIKPWPVQKLTTQPDGGLPMTVTARR----MKDREAESKLSRDVVRNLAPDAMHASSGKT-TLSPVAQTIASQYARDTNSPVMISGIMRIIEFLIVAFAGVAVFAVYVGFSAGAILHYALAILATATVTVLLLELTDSYQLAVMRDPFSKLGKLLLIWSGCFALMSLAAFFLKISEDYSRVWFGAWYISGVILFIAFRVIMSRLIRRWARNGKMERRAVIVGGGKPAEDLIRSIEQQPYNDIRICGVFDDRDAKRSPPIVAGYPKLGNISELIEFARIAHIDMLIVSLPITAEERVLSLLKKLWVLPVDIRLSAHNNHLQFRPRAYSYIGSIAMIDLFDKPINDWDSVAKRAFDIVFSLFGILVFSPIMIATAIAIKLESKGPVIFKQQRHGFNNEIIEVWKFRSMYTEMCDPTARNAVVKNDPRVTRVGRIIRKTSIDELPQFFNSLMGTLSLIGPRPHAIAAHTRNLLYNEVVDGYFARHRVKPGVTGWAQINGWRGEIDSDDKIKMRTEFDLYYIENWSLWFDLKILFLTPVRLLNTDNAYMTDLVRPRVKYVIGPDGSPLTIADLPPANTRRWVIRRKAEVVAAVRGGLLSLEEACQRYTLTVEEFLSWQSSISDHGLAGLRTTRIQQYRHMSSTESRLESRRSGA--WASSLLLATGRFILIGAITFLGLLAVTFFIGRVIPIDPALAIVGDRAPAHVVERVREELGLNLPLYEQFVIYVKDALQGNFGNSVLTTNPVMTDIRRVFPATMELATLGTLIGALIGVPLGVLAAVKRGSIIDQIVRIIGLIGYSVPIFWLGLLALLVFYAKLGWTSGPGRIDVVYEYTFTPITGFYLLDAVLQGNWEAFRNIFSFIILPASLLGYFSLAYISRMTRSFMLNELEQEYIVAARAKGLSETRIIWGHALRNAAVPLVTVIALSYAGLLEGSVLTETVFSWPGLGLYITNSLQNADMNAVLGGTIIIGTVFVAINLLSDILYRALDPRTRARMSSMLDVEDLRVQFPTRTGIVEAVRGVSFTLGRERLGIVGESGSGKSQTGRAIMGLTPPHARITAKTLNFDGIDLLGASVKERRALRGNRIAMILQDPKYSLNPVMSIGRQIVETLRTHEKVSKAEARRRALEMLTAVQIRDPERVYDLHPHEVSGGMGQRAMIAMMLITGPELLIADEPTSALDVTVQLDVLRILDNLVRDRGMGLIFISHDLRLVSSFCDRVIVMYAGRIVEEIAARDLHMAKHPYTQGLLNCMPQIGADRHPLPVLDRKPEWAAMTAAI-DLPLTGLLVVDMSQFLSGPYASLRLMDLGARVIKVERPDGGDLSRRLYLSDTEIGGDSTIFHAINRAKESLAIDLKDAADLATLKGLLTRADVLIQNFRPGVINRLGLDYEAVKAINPRLVYASISGYGEEGPWVTRPGQDLLAQSRSGLMWLNGDESQGPVPFGLAVGDMLAGAATVQGILAALVRRGISGEGSHIETSLMESLIDFQFEVLTTHLNDGGRLPKRSDFRSAHAYLSAPYGVYQTKDSYLALAMTPLPKLADLLELDELAPYRDMPATWFTARDAIKRIIAAKLATETTDHWLSILEPADIWCAKVLNWEELLQSDGFKVLDMLQTVNREDGVSILTTRSPIRVNGKRAKFDRAAPRIGEHSKAIRKEFGL---MVANKTVSAESGKTLQTIRNLWPYMWPDERPDLKMRVVWATVYLLISKVVLMLVPYFFKWATDALNGKLETSELVPVLLIGPLMLVAAYNVARIVQAGLNQLRDALFASVGQYAVRKLAYRTFVHMHDLSLRFHVERRTGGLSRIIERGTKGIETIVRFTILNTAPTIIEFLLTAVIFAVTYGLSYLAVVVATVWLYTWFTVRASDWRISIRRDMNASDTDANSKAIDSLLNFETVKYFGNEAMEAKRFDTAMARYEISATRIWTSLGWLNFGQAVIFGAGMAVMMIMSGREVLAGTQTIGDFVFINAMLMQLSIPLNFIGFIYREVRQGLTDIEQMFDLLDVEQEVMDKPGAKPLTIDRGSVRFDNVHFAYDPKRPILKGVSFDVPAGKTVAIVGPSGAGKSTLSRLLFRFYDIQGGSISIDGQDIRDVTQESVRSVIGMVPQDTVLFNDTIAYNISYGRISATDEEMRNAAELAQIGPFIESLPEGYDAMVGERGLKLSGGEKQRVAIARTILKAPPILILDEATSALDSATEHEIQSALDLVSRNRTTLVIAHRLSTIIGADEIIVLKDGVIAERGTHLKLLAKHGLYASMWNRQREATEAEERLRRV-------QAEDDLGVVIRGKPALPA----MTEEDDSDRYRAPALDKGLDILELLSGIDGGLTQAEIAKFLGRSPNEFYRMLDRLVRRGYVTRLEGDRYSLTLKLFGLAQLHAPVRRLASYATPLMRDLAVSSKQANQLSVFDRGAAVVIAQQEAPDYWGISIRVGSHISLFNTGSGHVLLAFRSDEERALMIAEYEKHADDIAQPPDFHERLNQIRERGYEVMPSAQTAGVYNVSAPVLGPDGRAIAALTCPYIQLLNMPDAPAVDDARKMLVATAQKLSELAGADVIHSSVVRMAMAQTFDTIFRSGIVVNQDGVGLRDIGISNGRIAAIGNLGGASAGEMIDATGLHILPGVVDSQVHFREPGLEHKEDLESGSRSAVLGGVTAVFEMPNTKPLTTSAETLEDKVRRGRHHMHCDFAFWVGGTRDNANDVAELERLPGAAGVKVFMGSSTGDLLVEDDEGVRSILRNTRRRAAFHSEDEFRLREREGLRVPNDPSSHPVWRDEIAALKCTERLVRIARETRARIHVLHISTAEEIEFLKDHKDVASCEATPHHLTLDASQYATLGTLLQMNPPVRDARHRDGVWKGIGQGIVDVLGSDHAPHTLEEKSKPYPASPSGMTGVQTLVPIMLDHVNAGRLSIERFVDLSSHGPNRLFGMASKGRIAVGYDADLTIVDMKRRDTITNAQVGSKAGWTPYDGKIVTGWPVGTIIRGRKVMWEGEIVTLSQGEPVLFGEALPASM--------------MLVSKKPLVVITRKLPDPIETRMRELFEARLNVDDRRLSKAELIEAVKQAHVLVPTITDHVSEEIINAAGPNLKLIANFGNGTDNIDVVAAAKRGITVTNTPNVLTEDTADMTMALMLAAPRRLVEGASILLDDGKWEGWGPTWMLGRRIWGKRLGIVGMGRIGTAVARRAKAFGLSIHYHNRKPVNPKTEEELEATYWESLDQMLARMDIISVNCPSTPATFHLLSARRLALMQPTAFIVNTARGQIIDEHALIEQLERGKLSGAALDVFEHEPAVNSKLRKLAKHGKVVILPHMGSATLEGRIDMGEKVIINIRTFIDGHRPPDRVLPRDW------MRPADPAAKD--RMKGTLVARGITKSYNGRQVVNGVSFGVRSGEAVGILGPNGAGKTTCFYMVTGLVPVDKGTIEIDGFDVTTMPMYRRSRLGIGYLPQEASIFRGLSVENNIKAVLEVVEKDRGARAQELDSLLEEFHIAHLRKAPAISLSGGERRRLEIARALASRPNFMLLDEPFAGVDPIAVADIQQLVRHLTGRGIGVLITDHNVRETLKLIDRAYIIHAGQVLTHGRPDEIIANQDVRRLYLGDQFKL-------------MDESVAINRETITLVERVMQAVRQRIAGRTLVPGAKLPSIRSFAETMGVSKSTIVEAYDRLAAEGVIQSRRGSGFYVAGHLPPLSLAEIGPRLDLAIDPLWVSRTSLEAGEAVAKPGCGWLPSSWMPEDGIRRALRALARGKDSSLADYAPPLGISALRQHLSRRMAEHGIEAATDQIILTESGTQAIDLLCRFLIEPGHTVIVDDPCYFNFHALLKAHRAKVIGVPYTPSGPDLEYFAKALTEHHPRLYITNSALHNPTGAVLSPVIAHRLLKLAEQYNVTIIEDDIFADFEHEPAPRLAAFDGLDRVVHIGSFSKSLSASVRCGFIAARRDWIDGLVDLRIATSFGGGRLAAELVLCMLTDGTYRKHMDGLRTRLARSMGETSSKLKSLGLTPWIEPAGGMFLWCRLPGDADAAAVAQHALAERVVFAPGNVFSQSQSANSYMRFNVAQCTDPRIFEVLQTALAST--------MKVKDADILIVPGYTNSGPDHWQTRWESKLSTARRVEQAEWTKPVREDWVAEVVKAINAAKRPVVVVAHSLGVPTFIHAIPEIGGKVKGAFLVTPPDVSNPKIRPKHLMTFGPYPREPFTFPSIVVASRNDHYCSYEVADDLAAAWGSMIVDAGESGHLNSESGHGPWPEGSMVFAQFLSRL---------MTTRQAILIAGPTASGKSALALRLATETGGVIVNTDSMQVYDVLNMLTARPSPEDLKAVPHYLYGHVSPITAYSTGRWHSDVECVLAKPELYEKTVIFVGGTGLYFRALLGGLSAMPDIPATVRSYWRRQDAEQGAERLHAILAEKDALGASTLRPTDSQRIVRALEVFDASGRSIVEWQRETGKPLIDTDDARKIVIEPDRQWLGERIASRFGMMMKGGAMDEVKALLSLGLSPDLPAMRAIGVREIAEVLAGRVLADEAAELATIATRQYAKRQMTWFRNQL--GDDWERL-------PYP---MEKVPMTQAGFDKLKEELRWRQQEERPRIIQAISEARAHGDLSENAEYHAAKEAQSHNEGRVGELEDYTARAEIIDISKLSGDKIKFGATIHLIDEDTEEKRIYQIVGDQEADPKAGRISISSPIARALIGKSEGETIEVNAPGGARSYEIIGFRFV------MNEPRRERSTEAFFGRRSGKTLRPMQRSIRDELLPLLKLDLTKPAPSDLRTLFSAEVEAVRMEIGFGGGEHLLHEAARCPTSGFIGIEPFINSMAKLVVDLHDNPLQNIRLYDDDATQVLDWLPKASLNGIDLFYPDPWPKKKHWKRRFVSQKNLDRFARVLKPGSIFRFASDIDTYVNWTLQHCRIHDEFDWQVATAADWHTPYEAWPGTRYEQKAHREGRTGAYLTFLRRMDLGDLIQPDAVLPALKVNSKKQLLQIMSEKAAALTGLSEREIFDTVLQRERLGSTGVGNGIAIPHGKINSVKRIAGVFAQLETSIDFEALDDQPVDIVFLLLAPENAGADHLKALSRIARMLRDPERIAKLRTAHDASLIYELLTTQPTSNAAMSD-NKKRPNPVDVHVGARIRLRRNMIGLSQEKLGESLGITFQQIQKYEKGMNRVGASRLQAIGNILNVPVTFFFDDMPGQSDKPKG-FDEESETTYVVGFLNSSEGIQLARAFAKITDAKIRRKILDLIRTLGDEEE--------------MKLRNIAIIAHVDHGKTTLVDELLKQSGNFRDNQRVAERMMDSNDIEKERGITILAKATSVVWKDTRINIVDTPGHADFGGEVERILSMVDGAIVLVDAAEGPMPQTKFVVGKALKVGLRPIVAINKIDRPDARHEEVVNEVFDLFAALDATDEQLDFPILYGSGRSGWMAKNPEGPQDQGLGPLFDLVLDHVPEPTVG--EGPFRMIGTILEADPFLGRIITGRIHSGTVKSNQQVKVLHGDGTQLETGRISKILAFRGLERQPVEEAHAGDIVAIAGLQKGTVADTFCAPEVTEPLQAQPIDPPTVTMSFIVNDSPLAGTEGDKVTSRVIRDRLLKEAEGNVALKIEESSDKDSFFVSGRGELQLAVLIENMRREGFELGVSRPRVVMQKDEDGNMLEPIEEVLIDVDEEYSGTVVQKMSERKAEMVELRPSGGNRVRMVFYAPTRGLIGYQSELLTDTRGTAIMNRLFRAYEPYKGEIAGRNNGVLISNDQGESVAFAMWNLEDRGPMVIDAGVKVYAGMIIGIHSRDNDLEVNVLKGKKLTNIRAAGKDEAVKLTPPIRMTLERALSWIQDDELVEVTPKSIRLRKLYLDSNERKRFEKGSKTAGAA-----------------------------------------------------------------------MEKQPSKTVKPGINTRLAHSGYDPRDYHGFVNPPVVHASTVLFPDAETMAGHKQKYTYATHGTPTTDALCRAIDELEGSVGTLLVPSGLVAVTLPLLAFLSPGDHLLMVDSCYGNTRHFCNTMLKRLGVETEFYDPLIGAGIEQLIRPNTRVVFTESPGSNTFEVQDIPAIVEKAHAAGAVVMMDNTWATPIYFKALDYGVDVTIHAATKYPSGHSDILMGTISANDKCFKTLDAAYTALGLCVSGDDAYQILRGLRTMGVRMERHAENALEIAKWLERQDGIVEVLHPGLESHAGHALWKRDFCGAGGVFSIVIDGG-VKQAHAFLNALRIFGLGYSWGGYESLALHVHLGGRVITGQNYAGPVIRLQIGLEDTADLIADIENGLAAAKAV---MDVRVSGVRKEFDRFPALHDVSLEINSGELIALLGPSGSGKTTLLRLIAGLESPTEGKVFFGDEDASLKSVQERNVGFVFQHYALFRHMTVIDNVGFGLKVRPNSSRPSSEEIRRRSSELLDFVQLSGLEKRYPAQLSGGQRQRVALARAMAIEPRILLLDEPFGALDAKVRKELRRWLREIHDKTKHTTVFVTHDQDEALELADRVVVMSQGRIEQIGTPDEVYDKPNSPFVYSFIGESSKIDVRVENGELWLADRRLGLKS-DLPDGDASLYFRPHDVALVSDS-DA---CIMGTVVASRRVGATRRVELEIGGASERIEIGISADHPAKEMSGIAFRPQHWRVFPKS----MLKKSFQILALMTAACFS-ANA-----AIAEPTYGIAMRGAPALPADYQYFPYVDPNAPKGGSITYGVVGTFDSLNPFLVKSLRTTARGIFNDPQFGNLVYETLMQRSADEPFTMYGLLAEKVETNDDRTWVEFTLNPKAKWSDGEPVTPDDIIFTFDILPEKGLPQYKQRKDRIASIEKVGERGIRFTFNDKADREFPLILA-LMPILPKHAINRDKFDASPLAVPVGSGPYIVSQVQPGQRIIYKRNPDYWAADVPSRRGFNNFDTITVEYFRTEQAQFEAFKKGVFDVFMEGDPNKWASSYNFPAFKDGRVVQETFETKSPANMFGFAFNTRREKFADRDVRGALAMMFDFEWTNRNLYADRYQRMGSYWQGSEISALGKPADATERALLAPYPDAVLPDVMNGTYEPAKTDGSGRDRKEMKRAYDVLMSKGYSIKDETMLDPEGTPLTFEILTRSVAEERLGLAYKRTLERLGIGVTIRTVDDAQYQKRLETFDYDMILGAYSGTLSPGFEQFRRWSSQSRDSNGSFNYAGVADPVVDALIGKMLSVREQDEFISTVRALDRVLISGNYFVPIYYQPAQWVARWSHIKHPEKTSINGYQFTSWW-----SDKR------------------------------------------------------MGKSIDELMEYLTGLGIEVSTVRHQAVFTVAEAQDLRGQVAGVHTKNLFLKDKKDNFFLVTVDEEAEVDLKTIHTKIGAASRVSFGKPEKLLELLGLTPGAVSVFGAINDTQLRVKVVLDEDLMAHDIINGHPLTNEATTSIRTGDLLAFLRATGHEPLILKLDG-STPN---MAMDARDIEKMIRDAIPDAKVTIRDLAGDGDHYAAEVVAESFRGKSRVQQHQMVYDALKGNMGGLLHALALQTSAPE----------MARKYFGTDGIRGQANAYPMTPDIAMKVGMAVGLAFKRGEHRNRVVIGKDTRLSGYMIENALVAGFTSTGMDVFLLGPIPTPAVAMLCRSLRADIGVMISASHNPFHDNGIKLFGPDGFKLSDDIELEIERLMDEDLADRLSSFDALGRAKRVDGDIYRYIEFAKRTMPKNISLAGLRIVVDCANGAAYKVAPAALWELGAEVVTINNEPNGTNINEDCGSTHPLGLIKKVHEVRADIGIALDGDADRVLIVDENGAIIDGDQLMAVIAQSWQESDRLAGGGVVATIMSNLGFERFLGDRGLTLARTQVGDRYVVEHMRANGYNIGGEQSGHIVLSDYSTTGDGLISALQILAVVQELGKPVSEVCRKFEPVPQLLKNVRTTGGKPLENKKVKSAIDEARDRLGNAGRLVIRPSGTEPLIRVMAEGDDRKLVESVVNDIISVIS---SAAMADA-----------------ITNWLHSVFIAQFDAWVVLGFFAQACFTMRFVLQWLASERAKRSVVPVTFWFFSLFGGALLFVYAIVRKDPVFIAGQGLGLVVYIRNLWLIANEKKAMTQDT-MPQKGRLFGLGVGPGDPELITLKALRLLQAAPVVAYHAAKGKKGNALTIVERYLDEKQLLVPLIYPVTTEILPAHMNYESIVSDFYGEITGLLREYLDAGNDVAVIAEGDPFFYGSFMYIHDRLAEDYETEVVPGVCSILGGAAVLGAPLVYRNQTLTVLSGVLPAEDLRTKLAATEAAAIMKLGKNLAKVRSVIGELGLMDRALYVERATMDAQRIVPLADVDPESSPYFSLILVPGDRWQGGGE--------------------MIDEIARPDDVRRQNRRLVLTGLRRHGSLSRTDIVAETGLSPSTVSAITTALIAEGIIGESRDSDVASNRRGRPQVALALNPEIGTIAAVELALNLVHAVLFDYKGQVISEELQRVPTLAAAADDLNQSICRMLDNQLGSLPPKAGRLMHISMGVQGVTDSAGGTMLWSPITPDSNIAFGETLSKRYGVTVAVANDCNMISEALRWSDPSHYAEDFAAILLSHGIGMGLYLKGKPFVGAQSSAAEFGHMLHVPNGALCRCGRHGCIEAYASDYAILRKTSGQEDNSQPAQDVDPAAFGEVADLARANDGSERAAFRKAGQAIGSGLRSLFSLIDPVPVALVGPGAGVFDLMEDELRGIVSGTSGWGATQDLNIRCYPDEHPLILQGCMMTSLLHLDTQIFAPGDVKEAPLKRAI--MTAFIKAVDRATEKMRALFPETPLQLNHYLSHKYGAQIWLKREDLSPVRSYKIRGAFNFISHYLNEHKSETSSFVCASAGNHAQGFAFICRYFAKKGVVFMPVTTPQQKIDKTRSFGGDFIEIRLVGDIFDQCYSAAQDYAAETGAVMVPPFDHMDIITGQATVAHEIAAQLPDKRKPDLMILPVGGGGLSGGVTRYLADLGWETRFRFVEPAGAPSLKRSLEAGKRIKLDAVDNFVDGAAVAEIGRENFKLLKGFTSDAVTLIPENRLSSTILEMLNVEGIVVEPAGALAIDALKDLKKSDIKGKRIILVISGSNFDFERLPELKERSLRYEGLKKYFIFRFPQRPGALRSFLDLLGPEDDVARFEYLKKSARNFGSVLIGIETKNKVNFDILFKRFDEAGWAYQDITENEAIAGLII---MSTRLTHLDETGAARMVDVGDKAETVRVAVAEGSVKMQSATLTLIKSGNAAKGDVIGTARIAGIMAAKKTHDLIPLCHPLLLSKISVDIEADEALPGLHVVATVKLTGKTGVEMEALTAVSVACLTIYDMAKAADKGMTIQDIRLLQKSGGKSGDWSVGASEN--MEGSIALLGFGEGGWGPSIASGILVTVSLALATLPLGLAVGFFVALGKQSSEPSVKLAANMYTTIFRGLPELLTLFLVYFGGQIGIQKLGQLFGYTGTIEINSFIAGMFAMGTVFSSYASEVFLSAFRAIPKGQYEGGYAIGLSNGQTMRKVILPQLIRIALPGLGNLWMILLKDTALVSAIGLADILRQTAVAARVTKHAFLFYGTACMIYLALAILSSFVIDGIDRWAAKSGVKR----MSIRLIGALSSALFFAGSTAVLADEYYPDNQ--PTSSTRGDHWWSGDWYLTLGAKGFVAPRYEGATEYLLRAAPVISLGRAGRSVRFSSLNDNASIGFVDTGVFRAGLTGKLITGRDDGDSDDLKGLNDVDWGFELGGFAEFYPTDNIRGRVEVRRGIGAHDGVVADVSVDAFKDLTETVRVSAGPRATFATEEYFQEYYGVNATESAASGLKQYSP-GGGLKSLGIGGQITWQTTDKVTTSAYAEYNRLMGPAADSSLVKERGSANQATFGLQATYRFDFSL---MIAQKPIRVLVAGLGNMGRSHALAYHNNPGFEIVGLVNRSIVPLPEELRSYTIHPSFDEALAELKPDLVSINTYSDSHADYAVAAMDAGADVFVEKPLATTVADAERVVAAARKHGRKLVIGYILRHHPSWVRLIEEARKLGGPYVFRMNLNQQSSGATWETHKSLMKTTSPIVDCGVHYLDVMLQITDAKPVQVRGMGVRLSGEIDADMYNYGHLQVLFDDKSVGWYEAGWGPMMSETAFFVKDVISPNGCVSIVMDQNAKSDDIDSHTKTSTIRLHSARTAADGRFAEPDELLNMAGEPGHQELCDLEQAFVLKAIRENLDLARHMLDAVRSLSVCLAADESVRTGRPVDL-MSFTLAIVGRPNVGKSTLFNRLVGRKIALVDDLPGVTRDRRVHAAKLYDLKFDVIDTAGLEEVDSASLEGRMRAQTEAAIGEADLILFLVDAKAGITPTDVTFADLARRSGKPVILVANKAEARGAESGMYDSYALGLGEPCPISSEHGQGFPDLRDAIVDAMGEERVFPDEHVKPSA--DDSVAVTIPATPRSMDDLVGDDIDDPDAEDVPAYDSSKPLRIAIVGRPNAGKSTLVNTMVGEERLLTGPEAGITRDSISVDWEWNGRKIKLFDTAGLRRKSRVQGKLEKLSVGDALRAIRFAEVVIIVLDATIPFEKQDLQIADLIIREGRAPIIAFNKWDLVENRQMVLADLREKTERLLPQIRGIRAVPISGERNQGIDKLMAAVATTDEVWNRRISTGRLNRWMEGVITHHPPPAVAGRRLKIKYITQVKTRPPGFVISCSRPEAFPTSYVRYLSNGLRETFDMPGVPIRVVLRTSDNPFAGRAKKKR-MSRFRAMMKMTAVRLSALYLLLFTVCALVLVFYMTSLSVRLLTTQTETAVNEEIESIGQAYERGGIAGLVRSIDRRGRQPGAYLYLVADPAGRIVAGNVQSIEIGVLQNEGWIERPITYERYGEG-NDPQMHTAIARVIVMPNGIRVMVGRDLGEPEQVRLVVRRALMAALGIMGVGAFLIWFFVGRRALQRIDEVSVASQRIMGGDLAGRLPVSGSGDEFDRLAENLNGMLARIQELNEGVRHVSDNIAHDLKTPLTRMHNRAESAL-SNAKTTKAWRAAMEGMISDSDQLIRTFNAILMISRLESGYSTETMEQIPLSTIVGDVFELYEPLAEDAGVKLVLGTMDQNQVKANRELVGQTISNLVDNAIKYAGGGENP-VEVTLSVEKEGGKVKVTVADNGPGIPEDQVTRATERFVRLEESRSQPGSGLGLSLAKAVMKLHGGTLELTDNAPGLRAVLVFPVGEENA---------MSGINEFIDNEVKSNDVVLFMKGTPGFPQCGFSGQVVQILDYIGVDYKGVNVLSSTELRQGIKDYSNWPTIPQLYVKGEFVGGCDIIREMFQAGELQTLLSEKGVATKAA-MQAGNVRYRTLDDIPESVPIFPLSGALLLPGGQLPLNIFELRYLAMVDAALAGKRIIGMIQPRFDPENEADA-------DLCEVGCLGRITSFAETGDGRVLITLQGICRFRVIKELSTRAPFRQCKIAPLRADLDEADDSD-VDRLALLRVFRAYLDANNLEADWDSISRAGNETLVNALAMMSPFGAAEKQALLEAADLKTRAETLIAITEIALAKDQDDFEGSLQMGWTPDSKRYDTMKYNRCGRSGLKLPAISLGLWHNFGNDTPHSLKQAICHQAFDLGITHFDLANNYGPPPGSAEEAFGEILRNDFKGYRDELIISSKAGYGMWQGPYGEWGSRKYVIASCDQSLKRLGLDYVDIFYSHRFDPDTPLEETMMALDHIVRSGRAQYVGLSSYNSKRTREAEAILEELGTPCLIHQPSYSMINRWIEEDGLIDTLEELGIGSIVFSPLAQGMLTDKYLKGVPEESRAAQGKSLNPNFLSEPNIANIRALNDIAGKRSQTLAQMAIAWVLRKGRITTALIGASKPQQVVDCVKALDNLEFTDAELAEIDKYANDANVNLWAASAERDGPQRVPPKLA---------MPINITMPALSPTMEEGNLAKWLVKEGDKVTSGDVIAEIETDKATMEVEAVDEGTVAKIVVPAGTEGVKVNALIAILAGEGEDAAAAAK-GAD--AAPAKVEAPK---QEAKAESAPAAPKAAEAA-----P------TPTPAVAPSAAPA--QAGN-RTFSSPLARRIAKDAGIDLAAVTGSGPHGRVVKKDVEAAIAGGGAKAAP-AGATPAAASAA-APKPMSDDAVLKLFEEGSYDLVPHDGMRKTIAKRLLEAKSTVPHFYLTLDCELDALLALRAQINAASPMRKTEKGDVPAYKLSVNDMIIKAMALALRDVPEANVSWTDANMVKHKHSDVGVAVSIPGGLITPIIRRAEEKTLSAISNEMKDLAKRARDRKLKPEEYQGGTTAVSNLGMFGVKDFAAIINPPHATIIAIGAGEERAVVKKGEVTVATVMSVTLSTDHRAVDGALGAELAQAFKRHIENPMGMLV-------MSGFAAIGECMIELSGANGDHWRMGFAGDTLNTTWYVRALTDPGFAVDYVTAFGDDPFSVKQRTFLRANGINTAHSPIVVGARPGLYAITLTGAERSFTYWRNDSAARHLADDPEALAKSLDGREVIYFSGITMGILSPKSRQNVLNAVSNARRHGSLIVFDANYRPRLWENAETAKQVLSNAFHITDIALPTFPDENALFSDVTPADTANRIASYGVREIVVKDGTEPALVVAGETRTEVPASRAKAVDTTGAGDSFNGGYIAARLAKLDPVAAAAHAHKVAARVVEFHGALAPMEEARKAFEA------------------MKLYRLLTGPDDASFCHKVTAAISKGWHIYGSPTYAFNSATGMMQCGQAVVKDVDGVDYDPG-LKLSDY-MNANGN--------SR-PLIIPALGSIYQNLGQLSETILRVIAGVALIVHGSGKIVNPFGAVGMVEGLGFYPGVFWSPLLSVTEFVGGVLLTIGLFTRPAAFATMIILAVTIYFHGIVQGQGWSGSEKSILWTAILFFFVVRGASSHSVDAKIGKEF------------------------MARDTTDETPITGTEELAAYLAQGSKPESAWRIGTEHEKFPFYTEDNSPVPYDGPRGIRAILEGMQSKLGWEPIIDGGNIIGLVEPTGQGAISLEPGGQFELSGAPLLSIHQTCRESNAHLAQVREIAEPLGIRFLGLGGSPKWTLAETPRMPKSRYNIMSNYMPKVGKDGLNMMYRTCTIQVNLDFASETDMRRKMQVSVKLQSIATALFAMSPFTEGKPNGLLSWRSNIWRDTDNNRSGVLPFIFSESFGFADYVEWALDVPMYFVMRHGRYHDCTHVTFRQFMNGALKDSVPEGTPNMGDWANHLSTLFPEVRLKRFLEMRGADGGPWRRICALPAFWVGLLYDAEALNAAEALTRDWTYPEVQALRDDVPANGLRATFRNLSAHDIARDALAISRLGLKNRARLNSEGFDETHYLSSLEEVVARGTTSAEQMLSQYNTAWGASVEPAFLEYAYMTNWLPELEPG------KGPLYLQLADRIETDIAEGVLAPGTKLPPQRNLAFDIGVTIGTVGRAYNLIRERGLVSGEVGRGTYVTDHKETSLVLPVA--HTEP-FGGTRSATIPS--GKIRMDSTAAIEVGQSAAMERLLAELTKEYPSEIASYSRTLPASWQQAGSKWLSAAGWVPEPSSVVPTLGAHSAILAVIAAITVPGDKIAFEDLTYCSAARSVNLMGRRSIIMQTQ-GSLTPDDFERHCAQQHPKLVFLMPSLHNPTAATMPEEHRRAIVEIARRYNVWIIEDEIYGSLMTHDHVKIADLAPERTFHIGGLSKSVAAGVRGGWVACPPHLAARVLTAHKMVSGGMPFILAELAAKIVNSGDADAIRAKVREEIVAREAIARSAFAGMEFTSQPLAPFLWMKLPDPWLSGTFKNAALNEGVLIDDEDEFKPGRTEKVFHRVRLGITVPTTRDEVRQGFAILRRLMETDNASYDSYSMDKFTKLTGVAAPLPIINVDTDMIIPKDYLKTIKRTGLGTGLFAEMRYNDDGSENPDFVLNKPAYRKAQILVTGDNFGCGSSREHAPWALLDFGIRCVISTSFADIFYNNCFKNGILPVRVSQEDLDKLMDDAQRGANATLSIDLEDLTIKGPDGGTIGFELDEFRRYCMLNGLDDIGLTMEKADKIDSFESANAEKHPWAMAELKRFIGIAAAGKALSREEAVEAFDIMMSGQATPAQIGALLMALRVRGENIDEIAGAVYAMRSKMLKVNAPSNAVDIVGTGGDQSGSYNVSTCAAFVVAGAGVPVAKHGNRALSSRSGAADTLAALGINIEIGPELIATCIEEAGIGFMFAPMHHPAMKHVGPARVELGTRTIFNLLGPLTNPAGVKRQLVGVFAPEWVVPVAHVLKNLGSESVWVVHGDGLDEITTAGVTKVAALENGTIRTFEIDPESFGLRLVDPKDLKGGTADENAVSLLSVLDGAHGAYRDITLFNAGAGLVIAGVASDLKTGIEMAAHSIDSSAARNVLKKLVHVSNSEASMLVPFLIMLREGIEAALIVGIVASYLKQTGRDAWMPAVWVGILLAVALSLFVGAGLQMVSAQFPQKAQEFFEAIVGLIAVAVLSSMVFWMRKAARSIKSELHASIDSALSHSTGQASALIALVFFAVAREGLESVFFLLAIFQQSTNNDALLGAVLGILVSVVLGYGIYAGGIRMNLRRFFFWTGLFILFVAAGILAGTLRHFHEAGVWNSLQTVVVDLSHVLPVSSPFGTLLSGMFGYQDMPTLGEVIVYVGFLAISIFMFLRPAGGRETAATASRPTN--------------------------------------MELVKETMRALAFLSRLPVPARWFDGYDGALYGTVRGFPLAGMIIALPASIVLLIAIAFDLPDIVTSLLTIVALIVTSGALHEDGLADVADGFYGGHSVERRLAIMKDSSIGSYGTLALIVSVVMRTALLTAVLDQVGPIHALIAVVGTEAASRGTLVKFWQSLPSARAGGVADRAGTPSEGEANSALIIGAVVLAAAYAAIGGLLPVVYAMLLTGLVFYGFSTLCREKIGGQTGDTLGAMQQLATISLMLGLVIAL-MSSTRTETDTFGPIEVAADKYWGAQTERSLHNFKIGGERMPIPLVRALGIAKRAAAETNMALGKLDEVIGRAITVAAEEVIEGKLDDHFPLVVWQTGSGTQSNMNANEVISNRAIEMLGGTKGSKKPVHPNDHVNMSQSSNDTFPTAIHIATAIEATERLYPALEHLTAALAKKEKAFANIIKIGRTHTQDATPVTLGQEFSGYRAALEFARTRVEQGLADIFLLAQGGTAVGTGLNAPVGFDTGFAEAVSDITGLPFKTAPNKFEALASHGALTNFHGSMNALATDLFKIANDIRFLGSGPRSGLGELKLPENEPGSSIMPGKVNPTQAEALTMVAAQVFGNNTTVTVAASQGHFELNVFKPVVALNVLQSIRILSDAMVSFADNCIEGIEADEVRIKDLLERSLMLVTALAPAIGYDNAAKIAKTAHKNGTTLREEALASGHVSAEDYDRLVRPERMIAPEMSAELILNNARIVLENEVIAGSVVIRDGKIADISEGSVA-SGEDFEGDYLIPGLIELHTDHLEGHYVPRPRVRWNPIAAVLAHDAQIATSGITTVLDALRVGLDEDADMSAPEIRKLADAIEDSVIQGRLRADHYFHLRCEVSAPDCLEGFHYFDGDDRVRLASLMDHAPGQRQFVNLETYAYYYQRKLKLSDHDFNLFCEKRMAESAKNSGPQRKAISDLCRERGIKLASHDDATTAHVDEAVEQGIDVAEFPTTLEAAAASKQAGLAVLMGAPNVVRGGSHSGNVSARELAEHGHLDILSSDYIPFSLIQSTFFLSEAVDGITLPDAVRLVTKNPAQAIGFEDRGVIEIGKRADLVRVRVDEHIPVVRTVWREGRRVV----MKADYYETLGVARSADEKELKSAFRKLAMQFHPDKNPGDAGAEHRFKEIGEAYETLKDPQKRAAYDRFGHAAFENGGMN-GGGGFSGAGGFADIFEDIFGEMMGGGRQRRSGGRERGADLRYNMEITLEEAYTGKTAQIRVPTSITCDECSGSGAKIGTQPVTCNMCSGSGRVRAAQGFFSIERTCPTCHGRGQIIKDPCSKCSGQGRTTEERALSVNIPAGIEDGTRIRLTGEGEAGLRGGPSGDLYIFLSLKPHEFFQRDGADLYCKVPISMTTAALGGQFEVATLDGTQTRVKIPDGTQNAKQFRLKGKGMPVLRQPAMGDLYIQIAIETPQNLNKRQRELLEEFERMSSQDNSPQSSGFFARMKDFFEGLSEMPIARQSRSASHPR----NEKLNKAVRQNRLLSRAGLSERLFAHVFKGLVYPQIWEDPEVDMEALQIRAGHRIVTIASGGCNAMSYLTADPASVEAVDLNTAHVAFNRLKLAAVANLPNYDAFYRFYGTADDKANLAAYERFIQPHLDTTSRAYWEKRMLSGRRRITIFSRDLYRHGLLGVFIGMGHRVARLYGIDPRDILKASTMEEQRAYFDRALAPLFDKRMIRWATKRKSSLFGLGIPPQQYDALATAGNGNMAQVLRGRLEKLACGFPLSENYFAWQAFGRGYSDNAETGPLPPYLSRNNFATVRERAGRMSVVNASLTEFLAAKPAASVDRFILLDAQDWMTDTQLNDLWREISRTAAPGARVIFRTAAEPTILPGRINDALLNRWAYQAEESLALHDRDRSSIYGGFHLYLLKD-------MSLVKKFATVASGTLMSRVFGFTREMLMAAALGTGPIADAFNAAFRFPNTFRRLFAEGAFNAAFVPLFAKEIEANGLHGARRFSEEVFGVLFTVLLGLTILMELTMPLIVRYVIAPGFVEDALKFDNTVSMAIIMFPYLACMSLAAMMSGMLNSLHRYFAAAIAPVFLNVILIAILGYAWLKGHDGTKVGYALSWGVMASGVVQLAIVWFAVRNAGISIGFRLPHLTPNVKRLLVLAFPAAVTGGITQINLLINTNIASAKSGAISSLAYADRIYQLPLGVVGIAVATVLLPELARALRSGNLKEAGSLQNRSVEFTLFLTLPAAAALLVMSEPIVRMLYERGNFSTASTHTVGNILAIYGLGLPAFVLIKAFLPGFFAREDTRTPMIFAIIAVIVNVTLALTLFPVMAETGIATAEVVAGWTNAILLFTTLVWRGHWGRDIPLLTRIPRLVLAAGLMGLALYYAIAWMGPALAPHAPLHTQAIAVMSLVGLSMILYFALAFGTGGASLGMIRRNVRRGAKTPEIPPEDTAS--MASSGKDRRIDYIEFNVSD--IARARQFYGEAFGWTFTDYGPSYCEFSDGGLKGGFTTEGKVGS-GGPLIILFADDLADTLRQVEKAGGQIVKPVFTFPGGRRFHFADPDGYELAVWSDK---------------------MKRILLAEDDNDMRRFLVKALEKAGYHVTHFDNGASAYDRLREEPFSLLLTDIVMPEMDGIELARRASEIDPDLKIMFITGFAAVALNSDSKAPKDAKVLSKPFHLRDLVNEIEKMLVAAMVSKRLSREAGHRRKFLAVIDETPECERAVAYASRRAKTTGGVLLLLFVIDSADFQAFLGVEQIMRAEAEERAHSTLSKFAASVRESIGIESELVVREGITSEEIQKLIEEDQDIAILVLAAGSGKEGPGPLVQSLAGRES-FSIPVTVVPYNLSDEDIDAIT--------------MRLLFLGDMVGRSGRTAVYEQLPGLISDLKLDFTIVNGENAAGGFGITEEIFHDTINAGADVVTTGNHVWDQREALVFAAREQRFLRPANFPLGTAGKGSGVFLAKNGARVLVSNIMGRVFMHPDLHDPFTIAEEILAACPLGEQADAVVFDFHAEATSEKQCFGHFVDGRASFVVGTHTHVPTADAQILNGGTAYMSDAGMCGDYDSSLGMDKEEPLNRFLSKVPKGRFEAASGKATICGVGVEISDRTGLAEKIAPLRLGPRLEETIPSFWKMPHWLTLMLESLPSLLWAGLIFTIPLTLLSFVLGLTVGLIAALIRLFAPVPFAAIIKFYVWIIRGTPLLVQLFLIFYGLPSVGIVLDAFTAALIGFTLNIGAYTSEILRAVISSVPKGQWEAAYSIGMSWGQAMRRTILPQAARVAVPPLSNTFISLVKDTSLAAAITVPELFQAAQRIVATTYEPLILYVEAALIYLALSSVLSSLQARLENRLNRYGGFLEARSMATRAKKSPA---RSAQTSAAKAPKPIQFTKEQELEAYRQMLLIRRFEEKAGQLYGMGFIGGFCHLYIGQEAVVTGMQMALREGDQVITGYRDHGHMLACGMSARGVMAELTGRRGGLSKGKGGSMHMFSKEKHFYGGHGIVGAQVSLGTGLAFANRYRDNGNVSLAYFGDGAANQGQVYESFNMASLWKLPVIYIIENNRYAMGTAVSRASAETDFSKRGISFNIPGIQVDGMDVRAVNAAGEEAAAWARSGKGPMILEMQTYRYRGHSMSDPAKYRSKDEVQKMRSDHDPIEQVKNRLIEKGWSTEDDLKKIDKDVRDIVADAADFAQSDPEPDASELYTDILL------MVAQLQSGSIT--RKSAVMRRKRSLALRRFFKPLNRVFGNYLFSSLTRRILFLNLAGLAVLLSGIMYLNQFRDGLIDARVESLMTQGEIIAGAIASSATVDTNSIAIDPEKLLELQAGQSITPTPDVLDNLDFPINPERVAPVLRRLISPTRTRARIYDRDSNLLLDSRHLYS--------RGQVLRYELPPIDGEEPSFFERLTNRMTRLLR-RSDLPIYQEQPGGNGSSYPEIVKALTGAPATVVRMTEKGEQIVSVAVPIQRFRAVLGVLLLSTEGGDIDKIVQGERFAIIRVFTVAALVMAILSLFLASTIANPLRRLAAAADRVRHGVKNREEIPDFSERQDEIGHLSTSIRDMTNALYARIESIESFAADVSHELKNPLTSLRSAVETLPLAKSEDSKKRLLDVIQHDVRRLDRLITDISDASRLDAELAREDSEHVDLRYLLGNLVTAAREVRRNKKDTRIEFVVGKLPNNKKGFFVVGHDLRLGQVVSNLIENARSFVPVEDGVITVTLQHMGDRIRILVEDNGPGIRVEQIDRIFERFYTDRPFGEAFGQNSGLGLSISRQIIEAHGGTLNAENIIDPTD-----PDNYLGARFIAILPVEVMTMFEAILLTIATAATPLLIAALGELVVERSGVLNLGVEGMMVMGAVAGFAVAQTTGSAWLGMVAAILAGAAFSLLFGFLTLTLVTNQVATGLALTLLGLGASAMIGESFVGLPGVKMASLYIPGFSDSPYVGKFLFGQDPIFYISILLTIGVVWFLFKTRAGLTLRAVGDNHTSAHSLGVPVVRIRYLAVLFGGACAGLAGGQLSLVYVPQWVENMTAGRGWIALALVVFASWRPWRILAGAYLFGAVSIGQLHAQALGIGIPSQFMSSLPYLATIVVLVLISRNKRLTLMNTPTSLGKPFVPDRMTAIVDIIGREILDSRGNPTVEVDVILEDGSMGRAAVPSGASTGAHEAVELRDGGARYLGKGVQKAVDAVNGELFDAIGGLEAENQIHIDQTMIDLDGTPNKSRLGANAILGVSLAVAKAAAEASGLPLYRYLGGPSAHVLPVPMMNIINGGAHADNPIDFQEFMILPVGASSIAEAVRYGSEVFHTLKKRLKDAGHNTNVGDEGGFAPNLKTAQAALDFIVESIEQAGFKPGEEIAIGLDCASTEFFKDGNYVYEGEKKSRDPKTQAKYLAKLVGDYPIITIEDGMAEDDWDGWKYLTDLVGNKCQLVGDDLFVTNSARLRDGIKMGVANSILVKVNQIGTLSETFDAVDTAHRAGYTAVMSHRSGETEDSTIADLAVATNCGQIKTGSLARSDRVAKYNQLIRIEEELGPQARYAGRGIFRNAMPVSERP---------ILILGGTAEAAKLAARLVSQGHRVISSLAGRTREPAILDGEVRIGGFGGARGLTGYIARENVSLLIDATHPFATQISDNALIAAASTKIPFVRLERPAWHARPGDNWTSVTSIEEAVSAIPARARVLLALGRQHIAPFSRRGDVHFVVRMIDPPEQPLDLVDFELELSKPGKVEDEAAFLAKRRLTHIVCRNSGGSASYTKIKAARELGLPVIIIDRPHRPVAHTLPDIESVGQFIDNTLGSFRA----MMSLNSTVFAGNVAFSNAAQLSLIAGPCQMESRQHAFDMAGALKELTGKLGIGLVYKSSFDKANRTSLIGKRGFGLENSLPVFADIHKELGLPTLTDVHTEEQCAILGEVVDVLQIPAFLCRQTDLLIAAAKTGKVVNVKKGQFLAPWDMKNVVAKVSESGNPNVMVTERGASFGYNTLVSDMRSLPIMAGIGAPVIFDATHSVQQPGGQGGSSGGQREFVETLARAAVAVGVAGVFIETHQDPDNAPSDGPNMVPIDQMPRLLETLLEFDLIAKRASLRPEKFQPKKMSRLFWTIIALIGVTLLLLMANNDAGSTLGMANDDFARFAYMGIWGIVLASALLGSGIPLSHFLRSMAIWIIIILALVAGYQYRYELQDVGHRVTAGLIPGSPISNRDGDGLVTVTLAKADNGHFEVRGEVNGASVPFMVDTGASSIVLSSEDARRAGLDPDKLTYGIPIMTANGTATAANVTLDSIKIGAIERRNIRAMVAKDGRMAGSLLGMNFLNTLSGFSVRGDRLILSD-------MMGVLSGLLSAGT-AYAAQPEPWQMTFQPAASAIMEQIIWFERYTLWFIIPITLFVMVLLAWVMIRYRASANPVPSKTSHNTAVEIVWTVGPVVILLFLAIPSFQLLTAQYSPEEPKMTIKATGYQWYWGYEYQVGDAPVSFDSVLLKDADRAALGKEDRKAFPRLLAVDNEMVVPVNTTVRLLVTGADVIHSFSMPAFGVKMDAITGRSNETWFKADKEGMYYGQCSQICGKDHAYMPIAIHVVSQEQYDTWFAAAGKDLPGAYKALTAKIDTDKNVNVAGN----------MTNGSSNRVLDELAKLVTDAAGAAQGVRREVETAFRGQAERLLNTMDVVQREDFEAVREMAIRARAENTALLARIEALEALVGQKD----EAGKPAKSGPAAG-RTKKE--MHIREIPWLEPVEAAERLRALGGLSFLDSAMRHDTLGRYSYVAGDPFGKLVVEKGAAHWNGAALDRHPLEAISHYLDLYRLDDQPGLPPFQGGAIGYFSYEFGRLLERLPQPEAPAQGAMPDAAWYFYDVVLAFDHQAEKVWLLSSGLPEADEAARGRKCLERSELFLRLLREEPKQETSK-VEQQRIARASWQSNFERDAYMQSVARVVEYILDGDIFQANIAQRFIADLPDGFAQWQFYKALRRRNAATFAAYLDHDDIVIASSSPERFVAVNGDQ-VETRPIKGTMPRSSDPNRDKELAEILLKSEKDRAENLMIVDLMRNDLSRVCRPHSVLTPVLCGLESYASVHHLVSVVTGRLATSKSLMDLIGAAFPGGSITGAPKLRAMEIITEIEREARGVYCGSIGFIGFNGNFDSNIGIRTV-LFQGGKAVFQAGGGITALSDPAAEYQETLDKAARIFTVFDQGDAP---------MKSTIFSAA--AVAIAAFAAPAQAAGVGACLITKTDTNPFFVKMKEGALAKSKELGVDLKTYAGKIDGDSESQVAAIETCIADGAKGILLTASDTAGIVSSVKQARDAGLLVIALDTPLDPADAADATFATDNLLAGELIGKWAAGTLGEKAKDAKIAFLDLTPSQPTVDVLRDQGFMKGFGIDVVDINKIGDEKDPRIVGHDVTNGNEEGGRKAMENLLQKDSGINVVHTINEPAAAGAYEALKAFGKEKDVLIVSVDGGCPGVKNVEAGVIGATSQQYPLLMASLGIEAIKKFADSGEKPKPTEGKDFFDTGVSLVTDKPVQGLESIDVKTGASKCWG----------------------MATERYNPRVAEKHWQKVWDDAKLFETPNDDPRDPYYVLEMFPYPSGRIHMGHVRNYAMGDVVARYKRAKGFNVLHPMGWDAFGMPAENAAMQNKVHPKAWTYQNIETMRGQLKSMGLSLDWSREFATCDVEYYHRQQMLFLDFVEKGLVTRKSSKVNWDPEDMTVLANEQVIDGRGWRSGALVEQRELTQWFFKITDFNEELLSALDGLDQWPEKVRLMQRNWIGKSEGMLIRWALDEKSHAGGENEIEVYTTRPDTLFGASFIAIAADHPLAKKAAEANMELAKFNEECRHAGTSVVALETAEKKGFDTGLRVEHPFDPDWKLPVYVANFVLMDYGTGAIFGCPSGDQRDLDFANKYGLPVVAVVMPKDADAATFQITETAYVDDGVMINSRFLDGMSPAEAFDDVANRLEKQTIGNRPQGERKVNFRLRDWGISRQRYWGCPIPMIHCDDCGVVPVPKADLPVKLPDDVEFDRPGNPLDRHPTWRHVNCPQCGKAARRETDTMDTFVDSSWYFARFTAPW-EDAPTDPKAAN---HWLPVDQYIGGIEHAILHLLYSRFFTRAMKLTGHLD-VSEPFKGMFTQGMVVHETYK---GKDG-WVTPAEVRLEEIDGQRRAVRIDSGEEIEIGSIEKMSKSKKNVVDPDDIIASYGADTARWFMLSDSPPERDVIWTEAGVEGAHRFVQRVWRLISEAADVLKTAEPAAASEGESLQVSKSAHKTVKAVGEDIEKLAFNKAVARLYELVNVLSGPLQKVAAGEADKALIGACRDATNMLVAMIAPMMPHLAEECGQALNGSGLVAHQAWPAYDESLIVENDVTIPVQINGKKRGDLTIARDADQLAVEEAALALDFVKAALNGSLPKKVIVVPQRIINVVAMTLTP-----DLVERFVAIVGEKNALSDPHDIEPYLIEPREKFGGRTSLVLRPSEVEEVSSILKLATETGTAIIPQGGNTGLVGGQMPDDSGQQVILSLSRLNRIRAIDPSGNLAIVEAGVVLTMLQEAAENAGRLFPLSLGSEGSCQIGGNLSSNAGGTAVLAYGNTRELCLGVEVVLPTGEILNDLRYVKKDNTGYDLKDLFVGAEGTLGVITAAVMKLFPLPKGKGVAYAGLASPEDALKLLSVAQSHAGPSLTGFELMPRIGVEFSVAHTDGVRDPLEAPHNWYVLIDISSSRSAEDARETIEAILVEAYETGLVEDAAIADSTAQEKSFWHMREAMSHAQRPEGGSIKHDISVPVARIPEFIAEADAAVLELIPGARIVSFGHMGDGNLHYNISQPVGADKEAFLARWSEVNKRVHDIVRSYKGSISAEHGIGQLKRKEMSETKSPVALDLMRRIKKSFDPAGIMNPGKVIMRRIDTLDDIAEGLEALLQTHPELGAIATRAGPLPLRRSEPGFESLASVIVSQQVSKASAASIWNRLAGLIEPLTPENYLAAGEEAWRLAGLSRAKQATLARISEAIIAGELDLHGLCEQPIDEAMAAMTSVKGIGPWTAEVYLLFSAGHADVFPSGDVALQHAYAHAFAESVRPDSKALRQFAERWSPWRGVAARLFWAYYAATRGRDGTPITMASGQANDLKRSVQALKAHWKKGAPKDMRVAFADDPKRFSRYSLQLDDLLLDWSKCLVDDEALSLLGDLSKAAKVEERRDAMFAGKPINNTEGRAVLHIALRNRSGKPIKVDGEDVMPGVKDVLDRMSAFANGLRSGEIKGSKGKPITDIVNIGIGGSDLGPAMATLAMSPYHDGPKAHFVSNVDGAHIADTLAGLDPATTLVIVASKTFTTIETMTNAKAARKWIADALGETAVGQHFAAVSTALDKIDAFGIPADRIFGFWDWVGGRYSIWSAIGLPLMIAIGPENFGRFLDGAHSMDEHFRKAALSKNLPMMLGLVGYWHRAISGYTSRAVIPYDQRLSRLPAYLQQLDMESNGKGVTVDGKPVSGPTGPVVWGEPGTNGQHAFFQLLHQGTDTIPVEFIVAAKGHEPHLDNQHEMLLANCLAQSEALMKGRTLEEAKAQLKAKNLPKSEVKRIAPHRVFTGNRPSITIIHDKLDPYTLGRLIALYEHRVFVEAQLFGINAFDQWGVELGKELATELLPVVSGEVKPKDKDASTQGLVAHLRKRRDK---------------MAFAVKT--GVEPSDKIGRRPGRRVVSAALTAGLGAVTSLGVIATVMVMHGAFLSRAAD-GVPTTQGLLQKPSTGSLIALVSAHEIAPEKPKYPR----VPELTPTPWQAADMEEGAVIGPSGDISFMSSP--KPRIVA-Q--APLAAEVFGARASGIEL--LAEEDFSARLAGLDTMQVASV----QTYTTFDTES----EVTGPSVAAAIAALENAPPPVGMSPQGPFSLVLDEADPSLGD-EDSPEVPLPGMRPSRPA---------------------------LKGVA--------------PQALAYAPQDNDLRDIA-PTYEP--PAPRLAG---RSRTAFYSIEAKTVYLPNGEKLEAHSGLGPMRDKVRYVHKKMRGATPPHTYKLTMREKRFHGVEAIRLNPVEPGKIFGRDGLLAHTYML-GPRGDSNGCVSIKDYRRFLAAFKRGEITQLVVVARMPS--ST--LFASR--------------------MSDRPTQLPFDPTMAQMGFVWFLRLVSIYCLVAGVGYWAQLSGYYEGLLWRFDLMPWQWKVASVSLAMLYPVASTGLWMMVSWGPVIWFVAAAGETLMFTVFSHYFFYRPQIAIVHGCVALLYIAFRIVLFLQKRHQAKPVR--MTTEKDIIQSTETRGRVFDAPSGHWVYRTLPNWLWPYAQLARWDRPIGWWLLLWPCWWATALAATAGA-KVGVHIWSVLPSPYHLVLFLIGAIAMRGAGCTYNDLADEDIDNKVARTRSRPLPSGQVTRKQAKIFLVLQALVGLTVVVQFNLFTILLAIGSLVIVAIYPFMKRITNWPQLVLGLAFSWGALVGWSAHFGSLS-----LPAVLLYAGSILWVIGYDTIYAHQDKEDDALVGVHSTARLFGSRTREALMILYGGALALFLGAFAVAEVPMPAVAGLLAAGAHMYRQIMTLDIDNPTECLRLFKSNTVVGWLVFLGLVFGGLWTVVKPLF-------------MVSPSGSPGTLSAEIKACRICRDAPRYGAPLPDEPNPVCVLSSTAHIVICGQAPGIRVHNTGLPFNDPSGVRLRDWLGVDRETFYDSGKFAIVPMGFCFPGYDAHGGDLPPRRECRETWHERVFRTMPQVELILTIGQYAQAYHLGRRRRASMAETVSNWREYFET--SNAPSILPLPHPSWRNNVWLKKNPWFAENVLPILREKVRILIS-----------------------MSSQPTIAVLGGGAWGTALGTMAASNGNAVRLYARDALAVDSINRNHRNNTYLPDIDLHPMLKASTDAEEVMSNSDLILCVVPAQSLADAMEGWKDLIPTATPLVVCAKGIERKSGLLMSDLVAKILPGHALGALSGPSFATDVAKGLPTAVTVAARSQALADRIAQILSGPTFRCYSTDDLTGVEVGGALKNVLAIAAGAAVGRGFGASAQAALVTRGFVELRRIGQALGAKPETIMGLSGLGDLMLTCSTPQSRNYSYGLALGRGEDLDGRPLAEGVATAAVAAELAVRHKIEAPIIAAVAEILDRRITIDEAMEALLARPLKNED---MKSYTPNR---HIETALTKNDASELDIIELFFFAYRDFTADPDLILEKLTFGRAHHRVLYFINRKPGMTVAELLDVLQITKQSLARVLKQLIDTGHVVQIPGPRDRRQRELYPTLKGRELALELAAPQSRRITSALEQIGLSDRTVIEHFLKAMVNPGQWQQIDSLPKAGMSSTSDIDQTAEIQASLLSAALPYMQRYENKTVVVKYGGHAMGDATLGKAFARDIALLKQSGINPIVVHGGGPQIASMLARMGIESKFEGGLRVTDAKTLEIVEMVLAGSINKEIVALINAEGEWAIGLCGKDGNMVFAEKAKKTVIDPDSNIEKIVDLGFVGEPVEIDRTLLDLLARSEMIPVIAPVAPGRDGHTYNINADTFAGAIAGALAASRLLFLTDVPGVLDKNKELIKELSVSQARALIKDGTISGGMIPKVETCIDAINRGVEGVVILNGKTPHSVLLELFTERGAGTLLVPMAAVEQKTVDTGEAEMRLDRWFKVHYPGLGFGHLQKLLRSGQIRIDGSRAKSDTRLQAGQTVRIPPLPVDSKAEG-HLTGKSIRGREDGDVLSQMLLYEDPKVFVFNKPAGLAVQGGSGVNRHVDDMLEAWRNKKGEKPRLVHRLDRDTSGVLVVARTRGAAQALTAAFRERETKKTYWALVKGIPRKREDKITTWLVKETTPDGDRMRIAQHGEPDSDHAVSYYRVIESAGQVLTWMEMEPYTGRTHQLRVHAAHIGCPIIGDPKYFEADTNWSFPGGIQNRLHLHARRIRVPNPS-GGVIDVTAPLPPHMVQSWNLLGFDENDADEKD---------------MSEAQDKANRLIEKGRLLFAQGWVFIRGVPAMKFLPPEGPLEIAFAGRSNVGKSSLINAIIGQKGLARTSNTPGRTQELNYFVPDGHSGENGDLPPLAIVDMPGYGFAEAPKEQVDAWTRLVFDYLRGRTTLKRVYVLIDARHGVKKNDAEVLDLLDRAAVSYQIVLTKIDKIKAAGIPRLMEETAQAIKKRAAAYPQILATSSEKGAGLDELRAAVALLLEE-----------------MGTLTTLTDEAKIALDTLADRTSALVSPSLRLGVTGLSRAGKTVFITAFVHNLIHGGRLPMFEAQKSGRIARAYLEEQPDDAVPRFQYEDHLKALIDERVWPDSTRAISELRLTIEYESASLWNRMFSGGKLSVDIVDYPGEWLLDLPLLGKSYSEFSKDSFELAQLASHRDVAVDWRQVAESADADAPADEMTAQRLARSFTDYLKAGKADERALSTLPPGRFLMPGDLEGSPALTFAPLPGLGTGRPNPGKSRPGSLAAMMERRYEAYKTHVVKPFFREHIARLDRQIVLVDAMQAMNAGPAAVADLERALTEILSCFRPGNTNFLTGLVQRRISRILVAATKADHLHHESHDRLQAIVRRLVDRAIGRADFSGASVDVLAMAAVRTTREATVTQSKETLPVIVGTPLKGETINGERFDGRTETAIFPGDLPEKPDSIFEQAESV-SEDPAIRFVRFRPPRLEKTAEGFTLSLPHIRLDRALQFLIGDRLAMARSRSDSLLLPFEPGKPDFASERKLMRSGIEFVVGIDEAGRGPLAGPVVAAAVILNPKKIPEGLDDSKRLTAQRREELFEIITDTALACAVASISAPAIDASDILRAALEAMRRAVCGLAILPGHALIDGRDVAPGLPCPATALIKGDQRSVSIAAASILAKVTRDRMMAQTGRYYPLYGLESHAGYATEFHRNAMETHGPVVGLHRFSFSPLKDRQI--MSETTEL----------PERESMEFDVVIVGGGPSGLSAAIRLKQINPELSVVVLEKGGEIGAHILSGAVVDPIGVDKLLPGWREEEGHPFKTPVTADHFLVLGHSGSIRLPNFAMPPLMNNHGNFIVSLGNVCRWLGTKAEELGVEIYPGFAGSEVLYDDKGAVIGVATGDMGIEKDGSHGPAYTRGMALLGKYVLIAEGARGSLAKQLIHTFKLDEGKEPAKYGIGLKELWQVDPAKHKLGLVQHTFGWPLDMKTGGGSFLYHLEDNTVAVGFVMHLNYKNPYLSPFEEFQRFKTHPAIRGTFEGGKRLSYGARAITEGGWQSVPKLSFPGGALIGCSAGFVNVPRIKGSHNAMLSGMLAAEHVAEAIAAGRANDELVAYENAWRSSEIGKDLKRVRNVKPMWSKLGTIGGVMLGGLDMWLNTIFGGWSPFGTWKHGKTDAASLEPASKHAPIAYPKPDGVLTFDRLSSVFLSNTNHEENEPVHLIVKDSALQKTSEHDVYAGLSTRYCPAGVYEWVEEPTGPKFVINAQNCVHCKTCDIKDPNQNINWVPPQGGEGPVYTSMMARHSFFCIDGHTCGNPVRVVAGGGPNLSGANMMEKRAHFLSEFDWIRTGLMFEPRGHDMMSGSILYPPTRDDCDVAVLFIETSGCLPMCGHGTIGTVTMAIEHGLITPKTPGILKLDTPAGLVVAEYVQEGQYVTSVRITNVPAFLYAEGLEVECPDLGTLLVDVAYGGNFYAIVDRQTNFTDLADYRALDLIGWSPVLRQRLNERYSFQHPEKPDINVLSHILWTGAPTQAEAHARNAVFYGDKAIDRSPCGTGTSARMAQLTAKGKLQPGDEFVHESIIGSLFHGRVEQAAEVAGKLAIIPSISGWACMTGYNTIFIDDRDPFAHGFVVKMGDLLPTYWPHILFVLSIALGVPAAIHATMTKEEVRAAIGWVGVIILSPVIGAVIYAVAGINRIRRSSISSQRAFLHEIGQDHLMRFDASEKNILAHFEKRFVAMKTVGDRVSRCRLTTGNEIVMLQSGDGAYHAMLDAIGAAKRSILVETYIFDRDKIGLRIADALIAAVKRGVEVRVLIDAVGARYSIPSIVGYLAKGGVPVDVFNGNIIVGLRLPYANLRTHRKILIVDGTIAFTGGMNIRAGFSAEVVGEDNARDTHFRLTGPIVTDLFHIASEDWRFTTREALSGEPWHIGASEKHAGEPVFIRAVPSGPDRSLEANHRMLMGAFSIAQTRIRIMSPYFLPDRELISAIVTAARRGVEVDVVVPAINNLKLVDRAMTAQFDQILKGHCRIWRASGNFNHSKLLVIDGLWAYVGSSNLDPRSLRLNFEVDLEVMDAQFASDVEALVESAMASAKPVLLQSLRARSFWVRLLDRIIWLGSPYL----------------------------------------------------MIKRYLVPVIALLFVILIGVGLVGFNLFRDKAIQDFFANMQQPAAPVSTYTVEPSAWTPEIEAIGTVSAIQGVELAVEVAGIVKEIPFAANQEVEQGAALLQLDDAIEKADIVAARAQDVLMDQTLARARTLSTRGVGAVSNVDEAEAASLAKKAEIAKLQAVMDQKLLKAPFKGTVGIPRIELGQYLSPGTVVVTLQDLDTMRVDFTIPEQTLSEIKIGQPIKLGLTDSDLSFKGAIIAIEPRIDPSTRLISVRAKVDNPDGKLRPGQFAQVRVTLPREDDVYTLPQTALVSSLYGDYIYVVRPAENK---ADAPKPAGDSSA--GTANAANSTAPADEKKPADAAPEQKLVATQVFVTPGRRSGLLVEITKGIKKGDIVVTAGQNRLSPGAAVKVTNDVNPAAKTDAGLAKKMNDLRFTMDAVHAGHA-----PVLTLPEQLATMQAADAVELLNELELADAIDIIAGMPRDHAVLILDQPELHSAAEIIEALPVATAQELLDAMSADRVTDIFHEFDTETRSRLVPGLGHETKLALNKLLRYPPHTAGSLMTTEFVSVPADWSVARTLQHIREVERSRETIYAIYVIDPATNRLLRAVALRRLITTEPDVSIMHVGRDVAPVTVAPLADQEDVARLFRKHDLLAVPVVNDGNQIIGIVTVDDVIDAMTQEMTEDTLKFGGMEALNKPYMQIGFLHMIRKRAGWLGILFLSEMLTASAMQHFEGELEKAVVLTLFIPLIMSSGGNSGSQATSLLIRALALQELRLKDWWRVALREIPTGLVLGSFLGLIGVVRITTWQMLGLYDYGEHWPLIAATVGTALIGIVTFGSLTGSMLPFILQKTGFDPASASAPFVATLVDVTGLVIYFSIAVAILSGTLLMQTVVIVIHLLIVLALVGVVLMQRSEGGGLGIGGGSGFMTARGAANVLTRATAILATGFFITSLALGIMARYGESPTDILNRIPTTTGNQPA---GQQPPTSGGVLNQLGGPSSNNGAPAS-DTPAAPPESGSAAPG--TTVPAAPESTLPAPTAPVTPAPAQPANPVPNSQ------------MATGKRPDKSGSV-DAPGAGKMPASDELDSRRRSLEAKLVSKGVGPKASSDDKGTETAAGVAQAMKLSSEFIAGVLAGAGLGWLADRFLGTSPWGLIILLLLGFCAGTLNILRSAGHVAENRGLTSIKE----VDRDKPE-------------------------------------MSEFLLPTFAAIAFPDIDPVIFSIGPLSVHWYGLGYVAGILFAWWYAKKLISKPRLWANNTPPMKPADLDDFVLWAALGVVLGGRIGYILFYDFARYIAHPLDIFKVWEGGMSFHGGFLGTALAMILFARSHRINVYSMFDTIAAGAPVGLGLVRVANFINSELWGRPTDVPWGMIFPTGGPFVRHPSQLYEAALEGIVLFTVLAILIFFGRKLKSPRFISGAFVTLYGLSRIFVEFFREPDSQLGYLYGDWLTMGMVLSIPMVLLGLGFVMSARNPEVATGR-----MKERAMAHSHEG--RDHTPKITTD----NQRKVLIAFFITFVFMIVEVIGGLLSGSLALIADAGHMVTDAAALALAFAAFHFGKRAADERRTFGYLRFEVIAGLINAIALFAILVWITIEAIDRFRNPGEVLAGPMLVVATIGLLVNMAVFWILTR-GDSEHVNIKGAVLHVLGDLLGSVGTIIAAIVIYYTGWTPIDPILSVFVCLLILRSAWALLRNSLHILLEGAPANAAPEEIEQYLKSTVPSLANVRHVHVWMITSGKALATLHVQPQPDADPRLLVKAVEKELVAKFDIEHATIAIDWP-DTAADDCCLGANATAGNDH---GHS-HHHDEDDHS---DHDHGV-------HAHSHEGHKH-MTEPVNSREFVDNAAALVEAARRAGADAADAVVVRSRSTGVSVRLGKVESTESSESDEFSLRVFVGRKVASISANAGSNPDVLAERAVAMAKVSPDDAYEGLAASENLVRNIRDLDLYDATNIDAAQLTEAALAAEAAALAVEGVTNSSGASASAGMGGLVLVTSGGFSGQYMGSRFSRSVSAIAGAGTAMERDYDFSSRIYFNELEDAAVIGRSAGERAVRRLGARQAKTGRVNVVYDPRVARGIAGHLAGAINGASVARKTSFLKDRMGTKILSDAINVTDDPLRVRGASSRPFDGEGLEGMPLNMVENGVLKHWFLSTSTAKELGLTTNGRGVRSGSTVVPSSTNFAIEPGEKSPEELIHSVGTGFYVTELFGHGVDMVTGEYSRGASGFWIENGETAYPVSEVTIASNLKDMFLNITPANDIDRHFGVAS-PTLVIEGMTLAGKMTYENIIVETREKVGFVQLNRPQALNALNSALLAELNVALEAFDKDDNIGAIVITGSEKAFAAGADIKEMQTLAFADAYLGDYFS-EWERVTRLRKPLIAAVAGYALGGGCELAMMCDFIIAAENAKFGQPEITLGVMPGMGGSQRLTRFIGKSKAMDLCLTGRMMDAAEAERCGLVSRVVPSGDLIEETLKAAGKIASFSLPVVMMTKETVNRAYETTLSEGLRFERRVFHSMFALEDQREGMAAFAEKRPPKFKNRMSGLPDDIPFFDEPDL-PRQ-------PSGIAARAMQAR-GA--PPYLAGLNPEQRLAVETTEGPVLVLAGAGTGKTRVLTSRIAHILSLGKAWPSQILAVTFTNKAAREMKERIGVLVGGAVEGMPWLGTFHSIGVKLLRRHAELVGLKSDFTILDTDDVVRLIKQIIQAEGLDDKRWPAKQFALMIDGWKNKGLGPSEIPEGDARAFANGKGRELYTAYQNRLKTLNACDFGDLLCHPIRMFRAYPDVLKEYHAKFRYILVDEYQDTNTAQYMWLRLIAQGSK---------------------------------------------NVCCVGDDDQSIYGWRGAEVDNILRFEKDFPGAVVIRLERNYRSTAHILGTASFLISHNEGRLGKTLFTDAANPEDAKVNVHAAWDSEEEARAVGEEIEQAQRRGHNLNDMAILVRASFQMREFEDRFVTLGLNYRVVGGPRFYERLEIRDALAYFRVVAQGADDLALERIINTPKRGLGEAAIRQVHDYARARDISMLDAATDLIETEELKPKPRSALREVVENFRRWQSQIESTPHTELAEKILDESGYTAMWQADRSAEAPGRLENLKELIRSMEEYESLRSFLEHVALVMDAEQNENLDAVNIMTLHSAKGLEFETVFLPGWEEGLFPHQRSLDEGGRSGLEEERRLAYVGLTRAKKNLHIWFVSNRRIHGLWQSTIPSRFLEELPEAHVEVVSTGDSYGGYGG-RGGYGQSRFDR-ADPFENSYSTPGWQRAQQHRSDATRNNWGSRSGARVERIGYGETDSGHGAGRGSVKGRTIDGELVAKSVSETPSNFDVGDRVFHIKFGNGNVSSIEGNKLTIDFDKAGQKRVLDSFVKGIMSGTRTLVIALAAGSLWSF----AAPAQETTTYTLEKTDDGYVRMNNKTGEMSICQEKAGQLICKLAAEERTAYEEDIADLKTRVTKLEETVAGMGKIPPVVRDALPSDEEFEKGLSYMEKFMRRFMGIAKEFDNDNSGTKPEQP-TPQKT-------MIRSS----------------SAFARRMLMVAAVAGIGFAGAVNNAAADAKFQSWIADFRGTAMKSGITAATYQRAFAGVTEIDPEVLEKARFQPEFTEPVWGYLDNRVNEHSVATG------REMARKYSRWLGAIEKSYGVDRNILLAIWSMETNFGEIMKRDDVMRDAVRSLATLAYADPRRAKFARTQLIA----------AMKILQTGDIDRSH----LTGSWAGALGHTQFIPTSYLAYAVDMDGNGKRDIWNSVPDALATAANLLRKNGWQSGRTWGYEVVLPAGRKFPSGTRTIAEWQSLGVVRANGKAFPHGSEKATLKVPDGRNGPTFLMTRNFFVLKNYNNADKYALGVGLLADQIAGASGLRQDWNRPFTPITMKEREELQTHLRELGYYSGKVDGKIGGSSRDAIQAFQKQMGLDPDGHPSKEVLTIIRKKIE----------------------------MAKALDGSICRVKLTFGNLSAGQARSVADAAQRFGNGVIEITNRANLQIRGVRPETENALIAQLLAAGLGPLTDKGDDVRNVMISPIAGHSKGK-IDVRPLATRLLTELQTNPRYEGLSPKFSLLIDGGEDLAMVDHPHDLWLCPIDPDNPSSAYAFGAAGAPPTQAEDQPALGVVAGSQAFDLMTITLDAFVDWNKRHPAASRMRHMITGARTEGLAERIETCAGFPVRSEELAGWRRALPKPNGHLGIWNE-CDGATRLVGAMPKLGRLNPDLLRSLAGLADRYSNGILRMTPWQSVLLPEVSRDDATAALTDLVSLGLGADPEQPLATMISCSGSAGCGSALAATQADGLKLAALLDGEAD----IPPIHMSGCSKSCASPSARPVTLVATSTGHYDIFLRATN-GPSRFGKLLAANVTIEEAAELI-GR-------------NSGSGGPLHA----MSTQSAIENKLTQAFHPVSLAVINESHLHAGHHHSDGDHHGTFDGSGETHFRVRIVAEAFAGMSRVARHRAINEVLEEELKGPVHALALEPAAPGEATRG--MHISYEIDADVSLQERLRFAGLRPTRQRVALASLIFSRGDRHLSAEELHEEAQGANVPVSLATVYNTLHQFTQAGMLRILAVEGAKTYFDTNVSDHHHFFVEGANEVIDIPDGSA---MVGALPPVPEGME---------IVNVDIIIRLRKKRG-------MRHVDFSPLYRSTVGFDRLFTILDSLAQPEGAQTYPPYNIERTGENAYRITMAVAGFSEDEIQIEAHRNQLKVTGEKSTENSDENAETLYRGIASRAFERRFHLADFVEVSGASLKNGLLHIDLKREIPEEMKPRKIAVTSLSDKEAKQIEAKAAN------------MKKV----IKSSIFGSTNEPHTIIIARGETIRHFTIRPWAGLLGGTLALGLAGSYLLATSYLVFRDDLITGSVARQARLQQAYEDRIATLRTQLDRVTSRQMLDQKLVDGKVAELIQRQKALAERHDKLVPAMQRAARAGATTDSDP----VMDRDDQDLVKSED--SDDFYGIDPIITGPTTSTRKPV--------------P--RDDKTKT-TIDKAE-LLDSVDHSLNDIESRQAQQIQTLSNTAYESADKIMEALAATGAKHVLDDGKA----GSGGPLLL--ANAGPTSGLDTKLADLDEALARLEGAKALAKAVPIANPIPGMPVSSPFGVRKDPLLGMVAFHSGMDFRATSGSSVLATANGTVTAADYNGGYGNMVEIDHGNGLSTRYGHMSRIVVSVGEHVKPGDVLGKVGSTGRSTGPHLHYEVRKNGNAINPVGFMTIGRQLAAEL-MTATGDTDARLIDVELDETIGRSTPDVEHERAVAIFDLIEENTFRPVNDEGAGPYRLKLSLIESRLIFAVARENGDDVVTHILSLTPLRKVVKDYFLICESYYEAIRASSPSKIEAIDMGRRGLHNEGSQALMDRLSGKIDVDFDTARRLFTLVCVLHWRGMTQRSCLTVILAAGEGTRMKSALPKVLHPIAGLPIVAHVAKAVHGAGGSDIALVVGRGAEKVEAAVRAVVSNVSVHEQTERLGTGHAVLAARNAIDAGYDDVLVVFGDTPLVETAALEQARAKLASGADVVVMGFRPDNPHGYGRLIEQDGRLIAIREEKDTSDAEKNIGFCNGGLMALRGDSALALLGKITNNNAKGEYYLTDVVEIANADGKSVIAIEISPD-NVIGINTRAELAEAEAIWQNRKRRAMMLAGVTMQAPETVF--------------FAHDTLIEADAILEPNIVFGPGVS-----------IASGAIIHAFSHLE-----GAKVGPNATVGPYGRLRPGADLAAKAKVGNFVEVKNAKIGIGAKISHLTYIGDATVGAEANVGAGTITCNYDGYNKALTEIGANVFVGSNSSLVAPVSIGDNSYIASGSVITANVPADALAFGRARQEVKEGRAVQLRARYAAQKLAKKVD-MNGSGTIRTGIGGWIFEPWRGTFYPEGLSKTRELEYAGQHLQTIEINSTYYGTQKPATFAKWASQVPNGFIFSVKGNRFVTNRRVLAEAADSIQYFVKSGIVELGDRLGPVVWQFAPTKKFDADDFGAFLDLLPKKEAGIGLRHAVEVRNPSFIDPNFIALANKNGVAVVYAEHFEYPEIADVTADFVYARLQKGDDAIETAYPAEALDKWAERTKVWAQGGIPEDLPLADPGQHPEKRPRDVFVYFIHEGKVRAPQAAQALKERLTT----------------------------------------MAFA-----------FCSNQ--GFAA-----SKSEIEAQFQIWLANDLWPEAKAKGISKATFDQAFAGVTLNFDLPDLVMPGEEPKTPQKQAQSEFGSPGKYFNTKTVNAVTSGGTARAGQYGRILKAVESKYGVPGEIALAIWGRESGFGSVKIPYNAFTVLGTKAFMATRKDMFRGELVAALEIIEKGYIGAGSMKSSWAGALGQPQFMPTSYLEHAVDFDGDGKRDIWNSVPDTLGSIAHYLAQYGWQRGRDWGYEVNVPESVSCALEGPDQGKPFAAWAKLGIARVNGKPFPSGETKREGFLLMPAGRHGPAFIVTKNFYVLKEYNMSDLYALFIGHAGDRIAHGAAEFSAGWGDVGGLYRSDIAGLQKTLEKKGYDAGGADGFPGFKTRRSIGDWQAKQGMAPTCFPDKDLIKAIGMASFLVLAPR----SQNGERDNDSTVFIRDGFAALAFILPVPWLLVHRLWFEAALVLGATIAISMVGNFTGHEDMAALVTALLSLLVGFEANAWRAAALERRGFEQLAVVDARNAGDAETAWFLGPNFAPTAKPAMSS-GVKSDGPLLPATQRPAFQPAIGGMVGLVSHRGEN-----------------------------------MTKKFGMIFALLGAMALYGSSSALSSS--A-APTNLADFIKAKRAN---ELKEKA-SDKSTKAVQSKGKVKSSAVKAPVVKKSAAAKQPRVSKANAT--------AKQQRVSKANTKQQRVSKKAASHKRPVVQAKTRV-----------KTVVTNTTIGSVDTMT---------TGSVAS----AMGSTKYSTIINSYAASYGVPVALANAVISVESNYQPNTTGSAGEIGLMQIKLETARGLGYTGSREALYNPDTNIRWGMEYLAGAHKLGNGTTCGTILRYNAGHGATRMNPVSANYCAKVKTQMASNMKTIKGPAIFLAQFAGDTAPFNSFDAICEWAAGLGYKGVQIPAWDGRLIDLKKASDSKDYCDELAGVAASHGLTITELSTHLQGQLVAVHPVYDELMDGFAAPEVHGNPKARQEWAVDQLKRAARASKHLGLKAMPTFSGALAWPFLYPFPQRPAGLVEAAFDELAKRWTPILNYLDEQGVDAAYEIHPGEDLHDGITYEMFLERVKNHPRANLLYDPSHFVLQQLDYLDYIDIYHERIKAFHVKDAEFNPTGRQGVYGGYQSWVNRAGRFRSLGDGQVDFGAVFSKLTAHDFDGWAVLEWECALKHPEDGAREGAEFINAHLIRVTERAFDDFAGAGTDDAANRRILGLS------------------MNDGQSNDRPIIAFDSGIGGLTVLREARVLIPDRRFVYIADDAGFPYGDWEEEALKQRIVGLIDGFIGKYDPELVIIPCNTASTLVLEDLRRVFPSVPFVGTVPAIKPAAERTSSGLISVLATPGTVKRAYTRDLIQSFATKCHVRLVGSQSLARMAEMHMRGELIDDEAIRAEIAPCFYEKDGARTDIVVLACTHYPLLVNVFRRLAPWPVDWLDPAEAIARRALSLLTPNGTGVEPEHGDDIAVFTSGNPDFPTRRLLQGFGLATGVIA--------------MLTVHLATTEETERLGQDLALSLRKGDLVTLSGDLGAGKSTLARGLIRTIADDNGYEVPSPTFTIVQSYPELRLPISHVDLYRLSSADELDELGLDEALDDGAVLVEWPERGADALPASSLRVTLLGEGDGRKAIIEGKPDAVARLQRSLAIRTFLGRAGFPVAERRYLLGDASARGYETITAEGKPPLILMNSPYNPGGPVLRNGKTYMQIAHLSQSVTAFVAMDKLLQSKGFSVPEIYAEDLDGGLLLLENLGSEGVLDASGAPIPERYEAAVRMLAHLHQHSWPQNIDAAKGYAHHIHSFDRDAMMIEVELLSDWYAPRLSGRPLDSQQKAQYVAAWDHVFSQLINVEESLLLRDVHSPNILWRGDATGMDRVGLIDFQDAMIGPSAYDVASLIFDARVTITPEFQDILLAAYIDERQTLTAPFDETRFRKAFAIMAAQRNAKILGIFVRLDERDGKPFYLQHLSRIQAYLNRVIDHPALAPVKDWCEKAGVL-TSEI-----------------MTTQTPATPQHRLVIVGGGFGGLETVNYLRNTDIAITLIDQRNHHLFQPLLYQVATSTLATSEIAWPIRHLLRKYKKVKTLLGTVESVDTAARTVVTADGEAIPYDTLVLATGARHAYFGHDDWEPYAPGLKTLEDATTIRRRILTAFEAAEREPDPGRRGEYLTFVIIGGGPTGVEVAGTIADLARDTLKGDFRVIDPATARVILIEGGPRVLSAFSEDMSAYAKQALEKLGVTVHLGNPVTECHADGVEFGGQSLRAKTIIWAAGVQASPAAKWLNAPADRAGRVMVNADLTAPGHPEIFVIGDTATVANGGKGMVPGIAPAAKQQGVHIAKTIKARLAGDQRPRPFTYRHAGDLATIGKRAAVTDFGWIKLKGYMAWWLWGLAHIYFLIGLRNRLAVALSWLWISITGARSARLITQKDAAEEVGVRSHTP-------MLSHLSIRDIVLIERLDIEFREGLSVLTGETGAGKSILLDALSLALGARGDASLVRHGAEQGQVTAVFDVMGSHPARSLLRDNDIDDDGDIILRRVQTADGRSRVFINDQAASVTLLRELGRRLVEIHGQHDDRALIDIDIHRTLLDAFGALETKAGLVRAAHKTWRDAEHTLVRHRAKVEAATREADYLRASVEELAKLDPEPQEEELLAIKRTEMMRSEKIAGDVNDANEVLSGNASPVPSLASLVRRLERKVPEAPQLLEPVVKAIDEALNALAEAQNGIEQAIRAIDFDPRFLEQTEERLFALRAAARKYSVPVDDLAALRDRMDADLADIDAGAERLAVLEKEAMAAREAYDQAALALSNDRRETAVHLKQAVMAELPALKLERAEFIVEMTTNTDSRTAEGIDTVEYWVRTNPGTRAGPMMKVASGGELSRFLLALKVALADRGSAPTLVFDEIDTGVGGAVADAIGQRLRRLAGQVQVLSVTHAPQVAARAQTHFLIAKSASGN--DRVATSIRAMETGDRQEEIARMLAGASVTEEARAAAVRLLRENAAVVS-MNPSSAPWPTLKRDLIAILRGVKPDEVLAIGEELVSAGIDVIEVPLNSPDPFQSIELLVKNLPPHVLIGAGTVLDPADVVRLDAAGGRLMISPNVDANVLAAAAGSGMVSMPGVFTPTEALAALKAGASGLKFFPASVLGADGIKAISAILPKGTVIGAVGGVDESSFAAYAKVGVRTFGLGSSLYKVGASSSFVRERAKVTVEAYDRIFAGV----MSAFTLADLERIVAERAASADAKSYTASLYTKGIGKAAQKLGEEAVETVIAAVSGDRQGVVSESADLLYHLLVVLGISGIGLEEVLEELDRRTAQTGLEEKAARGHD-----MSKRESSKYKIDRRLGENIWGRPKSPVNRREYGPGQHGQRRKSKLSDFGVQLRAKQKLKGFYGDISEKQFRKTYEEAARRKGDTGEQLIGLLESRLDAIVYRAKFVPTIFAARQFVNHGHVNVNGRRTNIQSYICKAGDVIEVREKSKQLVIVLESVQLAERDVPEYLEVDHNKMVAKYNRVPAFSDVPYAVQMEPNLVVEFYSRMTADILSTKDKILQAAAEIAKEVGPVHLSLDAVARRAGLSKGGLLYSFPTKAKLLEALVEQHVGEFDESLREEEAKRNHGPDSVVQACIEVYRAEFVCNEPEPSGILAAIANDPGFIEPIRRYNRQLWQRMKENSEDPTLALIAYLTIEGLRSLKLFEMDVPSRDEQIEAIDRLRNLFERVDSNRASQPIDA----MSDYLKDVRHYDAAADEATVAKIVKHLGIALRNRDSSLVSCTDPDELKRVRTNWVEKKLGITDVAKADAAIESVCSAMKADNTKSRVTFYYLTAKALDALGGL---------------MVAEILEDDEERLDLIDCPVLVTERLVLRAPRNEDVDAIARLADNLHVAEMLSRMPHPYTKEHAVDFVRRANAGEIGKCVYAITLAQSGTFIGCCGINYRRNKDEHAP-SHKDELEIGYWLGEPYWGKGYATEAAHALVDLVFRATQVEKLHASCRVSNIASRRVIYKSGFQYANTGMMDSLAAGNVPVERYMLDRRTWIGLRSWTSMLKLYEKQIFSRLLLGTAQYPSPAILEGAIESSGTEIITVSLRREMMGGNSGNKFWSMIQSLARHVLPNTAGCHTVKEAVLTAQMARDVFATDWIKLEVIGNHDTLQPDVFALVEAARILTEDGFKVFPYTTDDLTVAERLLDAGCEVLMPWCAPIGSAMGPLNLHALRSLRGHFPNVPMIIDAGLGRPSHAATVMELGFDAILLNTAVAKAGDPVAMAGAFAKAAEAGHAAYHAGMLEPRDVAVPSTPVIGKAVFS-MKKYATALAGAAVLAVSIAGPALAKDKVVGVSWSNFQEERWKTDEAAIKKALEANGDKYISADAQSSASKQLTDVESLISQGANALIILAQDSGAIGPAVEKAVAEGIPVVGYDRLIENKDAFYITFDNKEVGRMQARGVFAVKPEGNYVFIKGSSADPNADFLFSGAQEVLKEAIDSGKIKNVGEAYTDGWLPANAQKNMEQFLTANDNKVDAVVAANDGTAGGAIAALQAQGLAGNVPVSGQDADFAALNRVALGTQTVTIWKDSRELGAQAAGIASALADGKKMEEIPNVQKFAGGANKVEVNAVFLTPVPVTKDNLDVVIDAGWVTKDVVCQGVKAGAVPACKMSSGFFGDIKPITYEGPESINPLAYRFYNPDEIVLGKRQEDHLRFAIAYWHSFVWPGGDPFGGQTFERPWFNDTMDGARLKADVAFEMFSILGAPYFCFHDADVRPEGDTIAESNKRLYEIADYFEGKMAMTGTKLLWGTANLFSNRRYMSGAATNPDPDVFAYAAATVKTCIDVTQKLKGENYVLWGGREGYETLLNTDMKRELDQMGRFLSLVVDYKHKIGFKGAILIEPKPQEPTKHQYDYDTATVYGFLKRFGLENEVKVNLEQGHAILAGHSFEHELATAAALGILGSIDMNRNDYQSGWDTDQFPNNVPEVALAYYEILKAGGFTTGGTNFDAKLRRQSLDPQDLLIAHIGGMDTCARGLKAAAKMIEDKVLTSFVDERYAGWSKPDGQAMLSGKLSLEDIAKKVEGNNIQPQPRSGKQEYLENVVNRYVMTIIVRFAPSPTGYIHIGNTRIALFNWLFALKNNGQFILRFDDTDTARSKAEYAEATIADLEWLGIDPVRIEHQSKRIPIYDAAAETLKAAGVLYPCYETAEELERKRKLRLARRLPPIYGRDALKLTDADRAKLEAEGRRPHWRFLLPNFKEDPFSVARTEVHWDDLVHGRETVDLASLSDPVLVREDGTYLYTFTSVVDDIDMGITHIIRGNDHITNSGVQISIIEALGAKVPELGHINLLTTASGEGISKRTGALSIGSLRRDGYEPMAVASLAVLIGTSESVVAVPDMGSLAEIFDPAATSKSASKFDPADLDALNRALIHAMPYAQARDRLAALGIEGDRTEELWLAVRGNLSRVSDAVIWWNIVKNGPASDEQLVEEDRDFVRAAFDLLPPEPWSRDTWKQWTDAVKAESGRKGKPLFMPLRIALTGLTSGPELADLLPLLGREGTLARQP---MIDGAG--QLAHATAVVVGDRGILIIGPSGSGKSSVARSLIDRALAKGTFAAVVSDDQCQLQAVSGRLICTVPAALRGGLEVRGSGLHAVDHE-DSAVMHLVVELVEPDRAVRFADETEIQLEGVSIAYALLP-----KREIESACRAIEARLFTPPWKKR--MMVFTLDSKLAADTFSIARLGLCDLRLMNDRRWPWLILVPQRPGICEIHELTPLDQTMLTFETGIAAQALKAVVNCEKINTGALGNIVRQLHLHVIARNTGDPAWPGPVWGHGTRE---------------------------PYGEKDA--RDFANAILKAI-MMPSAIFPDLKDRSVLITGGGSGIGAALTEGFVRQGSRVAFIDIADAPSQALVERLDKEYGNAPLYLKADITDVEHLRNAIAKAIDNNGLITVLVNNAAWDDRHDIDDVTVEYWDKNQSINLRPQFFAAQAVVPGMRQSGGGSIINFTSTSYMINQGNMPSYTAAKAGIIGLTKGLAGRLGPEKIRVNAIAPGWVMTERQKTHWVTEEGLKAHLNKQVLREEMQPGDMVGPCLFLASDASRMLSAQTLIVDGGYLMKPSRDIARLLDIMAALRTPVTGCLWDLEQDFKSIAPYTLEEAFEVIDAIERNDIDDLREELGDLLLQVVFHARMAEELKAFDFGDVVQAITHKMIRRHPHVFGEEAARGAGMAKGMWDKIKAEEKAERRERRAALNLGLAEAGGFLDDIPHGFPALMRALKLQQKAAKVGFDWSEAAPILDKIEEEIGELKEAIASGDRQDTEEEYGDLLFALVNLGRHLKLEPESALRGTNEKFRKRFHYIERKLAEQQQSLDAATLEEMEALWQQAKTAK--MSSADHPVTKRRTFAIIAHPDAGKTTLTEKLLLFGGAIQLAGEVKAKKDRIQTRSDWMNIERDRGISVVTSVMTFEYKDCIFNLLDTPGHEDFADDTYRTLTAVDSAVMVIDAAKGIESRTLKLFEVCRMRDIPIVTFVNKMDRESRDPYDILDEIEQKLALDTAPITWPIGRGKSFAGTYNLQTNTVRRRDEEEVPTPVNGPEAAAASGLL---PENERQAWIDEVTLARDACNPFDLQSFREGHMTPVYFGSALRNYGVRDLIEAFCDYGPSPRAQDADTRKVEATEDKMTGFVFKIQANMDPNHRDRIAFLRVCSGKLSRGMKAKLVRTGKPMGLSAPQFFFARSRQIADEAWAGDVVGIPNHGTLRIGDTLTEGEDLLFRGVPNFAPEILRRVRLDDAMKAKKLREALQQMAEEGVVQLFLPDDGSPAIVGVVGALQIDVLTERLKVEYTLPVGFEPARFTVARWISADDPAELQRFINAHRADIAHDLDNDPVFLAQNSFSLSYEAERWKAIRFAAIKDYQVREKAAMTRETGTHNAAIALGTSGLKGLSAVYYNLGAAELYEETLRRGEAQLTAQGALVARTGQHTGRSPKDKFVVRDASTEDHIWWDNNAPMSPEAFELLYADFMEQAQGKELFVQDLIGGADADYSLNTRVITEFAWHSLFIRNLLIRPERETLTSFVPQMTIIDLPSFRADPARHGTRTETVIAVDLKRLIVLIGGSAYAGEMKKSVFTALNYILPAKGVMPMHCSANEGPDGDTAVFFGLSGTGKTTLSADPARTLIGDDEHGWGEHGVFNFEGGCYAKTIRLSAEAEPEIFATTQRFGTVLENVVLDENRQPDFNDGSLTENTRCAYPLDFIPNASKTGKAGHPKNIIMLTADAFGVMPPIAKLTPAQAMYHFLSGYTAKVAGTERGVTEPEATFSTCFGAPFMPRHPSEYGNLLRQLIADHNVDCWLVNTGWTGGAFGTGKRMPIKATRALLTAALDGSLKNAEFRTDPNFGFQVPVSVPGVDGAILDPRSTWADKTGYDAQANKLVDMFIRNFGKFESHVDSEIRAAAPHTPIAAE------------------------------MKTLIIPGYRGSENGHWQRQWLAEDETARLVEQDDWENPVLSQWLHVLEAALAETPGAILVAHSLGCVLVSHLASRPSAAHVAGALLVAPADAERMAVQDPKFRSFAPLPRHNLGFPSIVAASRDDPHMSFNKARALADIWGSGFVDMKYAGHINIESGFGSWPEARILAESMRRPQEALRTQRAAMSLSMHKISVPAFIRGFAVLSSLIDKAEAFAAEKKIDQAVLVNARLAPDMLPFAGQIQRASDTAKATIGRLTTIEIPKFPDEEQTLGELRERVAKTVAFLETVDQSALEGSETKEVTLSAGPLKVTLDGVEYILKFVLPNFYFHVTTAQNILRHNGVQVGKLDYLGPLS----MNRILLKCAMVMV-ISVACSLLITGIMMSANDMHDPRGYLIATVCPLVISPAISFLVFRQSEKLRLTLETLNRVMQELEITNAKLFEKSSRDSMTGLLNREAFFNYMEGARQH-QMGSLLIIDADHFKKINDMHGHYNGDGALMAIGQCINNCIRPDDSIGRIGGEEFAVYLATDDESQVKSIAETIRMEVNRIEYRTPENERVPLSVSIGGVVGVSKGTIADYFQIADRRLYEAKRNGRNCVCIQSKMQNAA----------------------MTRHDDHHHRDSGLARPGKVKEVTSLTNPIVKDLRSLAIKKYRDQQGAFIAEGLKLVIDALDLGWTIKTLVYSKAGKGNSLVEQVAARTIAKGGDVLEVSEKVIAAITRRDNPQMVVGVFEQKVLSLSAIKPQGNDVYVALDRVRDPGNLGTIVRTADAVGAKGIILIGDTTDPFSLETVRATMGSVFAVPVTRATEAEFLTWRKGFSGLIVGTHLKGAVDYRTIAYGNKPVILVMGNEQQGLPDHLASACDKLARIPQEGRADSLNLAIATGVMLFEIRRGALKLD---------------SGT--------------------------------------------------------------------------------------------------------------------------------------------------------------------------MTVHFIGAGPGAPDLITVRGLKLIQSCPVCLYAGSLVPPEMVAEVPAGARVIDTSSLTLDEIVAEFERAHTEGKDVARVHSGDPSIYGAVAEQIRRLDALGIDYDVTPGVPAFAAAAAALKKELTVPEVCQTIILTRTSMKSSSMPEGEDLATLGKSGATLVIHLSIRNLARIEAELIPLYGADCPVIVAYRVGWPDEQFVHGTLADIGGKVRSSGLSRSAMIFVGRGLSVDAFRDSALYHKDHSHSLRSRSE-------------MAKTLGIGIIGAGNISTTYLRLAPLFKGIEVRSIADINRTAATGRASEYGVEARSIDALLTSDDIDLVVNLTVPDAHFDVSKQILSAGKHLYSEKPLTLSLKDGLELQKLAARKNLKAGCAPDTYLGGAHQLARKLVDDGQVGTITSGTAHVLSHGMEHWHPNPDFFFRPGGGPILDLGPYYVANLINLIGPVRRVAALTSMATATRTISSEPRRGETIQVTTPTTIQALLEFESGASVTLSASWDVWAHHHPNMELYGTDGSIFLPDPNFFGGEVKLAKPG-KKAKPVKAWAHPFGTPNQKHPAGMMANYRTAGLADMAVAILQGR-DIRCSLERALHGVDVMVSILRSGEEKKFIDIKTSCSRPEALGIKEAKSLLKKTMEIDFGGEGEIVFERRGKAGLVRLTRPKALNALTRTMVAAFHRALVAWSTDPDVLCVIVEGEGRAFCAGGDILAVYQAGRAGKPLYEFFAEEYRLNAYIRHFPKPYISLIDGICMGGGVGISVHGSHRVVTENVMFAMPEVGIGFFPDVGGSAFLPHLPEHFGTYIALTGNRIRQGDCLQSGIATHAIKAEDKERVRRSLIRTGNPDTALRG--KTINPDNETPEKTRDMISVLFDSETLGGCLIRLAAAGVN-----GNEWAQHILDLIKTRSPTSLHVTFRQIEEGRDLEMDQCMQMEYRILSRMLENHDFYEGVRALLVDKDNKPVWQPANIDDVTPEMVDAYFAPLGE--------RELQLTMPKFETSRPVVHPALQMFDLVADVESYPQFLPMCESLKVRSRKESHGKTLLVADMTVGYKLIRETFTSQVLLKPEEKVILTKYIDGPFRYLDNRWQFVPDANPEKSTVKFYIDYEFKSRTLGFLMGSMFDIAFRKFTEAFEKRADAIYGKGEKSPVVMSEPQ------ALLSIENVETYYGNIRALSGVTVHANPGEIVTLIGANGAGKSTLMMTIFGMPRARTGRIIFDGKDITKLPTHDIARLRIAQSPEGRRIFPRMTVQENLQMGASLDDLKYFDEDSRMVFDLFPRLKERINQRGGTLSGGEQQMLAIGRALMARPRLLLLDEPSLGLAPLIIKHIFEAIKELNKNTGLTVFLVEQNAFGALKLADRGYVMVNGIVKMSGSSADLLANPEVRAAYLEGGRHMKPIVDIEPKESDSVFAKALKEGLIAGALALGLFCLIVGFRTDQNIRNELILTQRWGLLTIFVLVAAVARFVIT-YTAPWREERKKTKRVVE---AKEPSAFRKSFPKIGLALLFVYPFLVITILALSQWYFTGQWSYQSGVQGSLKYVDNFGIQILIYVMLAWGLNIVVGLAGLLDLGYVAFYAVGAYSYALLSTHFGFSFWLLLPMAGIFAATWGVILGFPVLRLRGDYLAIVTLAFGEIIRLILINWTAVTKGTFGISGIAKATLFGIPFVPGPNGFAALLGLPNSGVYYKIFLYYLILLLALLTAWVTIRLRRMPIGRAWEALREDEIACRSLGINTTTTKLTAFATGAMFGGFAGSFFAARQGFVSPESFVFLESAIILAMVVLGGMGSLVGIAIAAAVMIGGTELLRELEFLKRVFGNDFTPELYRMLLFGLAMIVVMVWKPRGFVGSREPSAFLHKRKVVSGEFTKEGHGMVERPHCRVFSIAPGIPFLPALVDALQSGRLIPGFPADPADPMALARATIYVPTRRAARTLRSLLVERSPVKSAILPVIRPLGDVDEDAAMFDMGSDALFDLAPPISATERLLLLARLVRPWRERLPDHVRSLFGNEEIAIPATTADAIWLARDLASLMDQVETEASGWSSLANIVSEDLPNWWQVTLDFLGIVTGLWPDILTERHRSNPAAHRNRLIELEAERLVRNPPPGPVIAAGSTGSIPATAKLLASIASLSQGAVVLPGLDRDMDAASWEVLANLADNPSIFGHPQYGLRKLLSELGVLRGDVIPLANVSPQKRAREHLLAEAMRPADTSEAWSIIDRKSDPFSTAIAPVALVEAVNEREEALSVAIALRDAIDKRGKTAALITADRDLARRVSTELARFNIAADDSGGRALRETQAATLMRLTMEAVFNPGDPVALLALLKHPLTRIGLERSVVLQAAETIELVALRGGTGRASLSGLAAFLEKRLGEGSSAPFEPVWLRQITVAQIEAARAACGALSRCMEPLIAFAGKAEPVQMAEIVHASVACLENLARDDKGDIAALYTGAAGEQFAAFLRNLVSADSGLDFLPLEWPGMIEALMSGEVVKPKAGAHPRLFIWGALEARLQTVDTVIIGGLNEGSWPGKTRNDPFMSRPMKSIINLEPPERRTGLAAHDFQMAMGMDRVILTRSQRSANAPTVASRWLQRLETVLGQPAAAELRARGAKYLNWARQIDHAADEDFVKRPEPRPPLEARPKHFSVTEIETLRRDPYAIFARRILRLRPIDPLIRDPDVAERGSLFHDILAHFTEEEIDPLRQDAAACLLEIGRRHFDNLALPEEIDAVWWPRFQSLVPEYLDWERDRAPLIAQRHPEIASKKISVGTTGVTLSGRADRVDLRRDGTVDIIDYKTGSTPSKRQAHVLLSPQLALEAALLARGAFHEVGNRAAADLLYVRLRPSGRVDPESILKVGTSMNASEKTAPELGELSWARLTELLTAYRDPQKGYLSRALPFKESDLTGDYDHLARVLEWSAGGADDGGEE------------MHRYRSHTCAALRKSDVGSTVRLSGWVHRVRDHGGILFIDIRDHYGLTQIVASPDSPAFKLAETVRSEWVIRVDGEVKARTEDTVNPNMPTGEIEVFARDIEVLSAAKELPLPVFGELEYPEDIRLKYRFLDLRRDTLHKNIMSRTKIIAAMRRRMTDIGFNEFSTPILTASSPEGARDFLVPSRIHPGKFYALPQAPQQYKQLIMMSGFDRYFQIAPCFRDEDPRADRLPGEFYQLDIEMSFVEQNDVLSTMEPVLRGVFEEFAAGKRVTQEFPRIAYDDAMRKYGTDKPDLRNPIEMQAVTEHFAGSGFKVFANMIANDPKVEVWAIPAKTGGS------RAFCDRMNSWAQGEGQPGLGYIFWR---------------------------------KEGEAL---------------------------------EGAGPIAKNIGPERTEALRIQLGLDDGDAAFFVAGDPKKFVSFAGAARTRAGEELNLVDRDQFKLCWIVDFPFFEWLEDEKKIDFAHNPFSMPQGGMDALENQDPLTIKAFQYDMVCNGFEIASGGIRNHLPETMVKAFEAVGHSRETVEERFGGLYRAFQYGAPPHGGMAAGIDRIVMLLVGAKNLREITMFPMNQQAYDLLMNAPSDVSPAQLRDLNIRLNPVKKEDMAVPKRKTSPSKRGMRRSADALKAPTYVEDKNSGELRRPHHVDLKTGMYRGRQVLTPKEGMLRNVFLALITLVIAFGGGTLSAWYVVTRFDGFGALSIGQWTSHPEAGTPFSDPYAKARAAREGAFPLGSTEGLAFYAYNDDQGRTLDRQCSYKIEGNSPNSRFWTLYATDRRLTALSPGTDRLPAIHSRQIFRKEDGAFMVSVSPKAQPGNWLATTGTGRMVLVMTLYDTPVGSNSGLVDMKFPAVTRI----SCDGMDRLDRKILRLLQEDATLAVADVAKKVGLSTTPCWRRIQKLEEDGVIQRRVAILDPVRVNTKVTVFVSIRTGSHSNEWLKRFSEVVQEFPEVVEFYRMSGDVDYLLRVVVPDIAAYDVVYKRLITKIDIRDVSSAFAMEQIKYTTELPLDYMSIEKEPAMNNPRHNIREIRAPRGDQLNTKSWMTEAPLRMLMNNLDPDVAENPNELVVYGGIGRAARTWDDFDKIVSTLKTLNEDETLLVQSGKPVGVFKTHANAPRVLIANSNLVPHWATWDHFNELDKKGLAMYGQMTAGSWIYIGTQGIVQGTYETFVEAGRQHYGGNLKGKWILTGGLGGMGGAQPLAAVMAGACCLAVECNPDSIDFRLRTRYVDAKAETLDEALEMIDRWTKAGEAKSVGLIGNAADVLPELVRRGVRPDIVTDQTSAHDPINGYLPKGWTMAEWREKRESDPKAVEKAARASMREHVEAMIAFWNRGIPTLDYGNNIRQVAKDEGLENAFAFPGFVPAYIRPLFCRGIGPFRWAALSGDPEDIYKTDAKVKELTPGNTHLHNWLDMARERIAFQGLPARICWVGLGDRHRLGLAFNEMVRNGELKAPIVIGRDHLDSGSVASPNRETEAMKDGSDAVSDWPLLNALLNTASGATWVSLHHGGGVGMGYSQHSGMVICCDGSDDAAERVGRVLWNDPATGVMRHADAGYEIAIECAREKGLNLPGILG-----MIILGLTGSIGMGKSTSAQMFVDEGIPVYSADEAVHRLYSGAAAPLIEAAFPGTTKDGKVDRTKLSAAVMGKPDKLKQLEAIIHPLVRAEENSFRDAARSRGAKLIVLDIPLLFETGAETRVDKILVVTAPADVQRKRVLDRPGMTPAKLDAILERQTPDAEKRARADFIIDTRHDFGVTREEVRKIIRLLSG----MKSHEGLVRLKLFQVKEKRRQLGQLDLMMGEFERMAAELDAQILSEEKKAGITDISHFAYPTFAKAARQRRDNLFVSIRDLKAQKDDAEAALVESEAELAKAEALEQRDGRPRD-EEMP-VTDRRAMIGMPSSSPIKKTGSVQHMDPIWHAIRSEAEETIRNEPLLATFLYSTILNHQSLEEAVIHRISQRLDHPDMESELLRQTFTAMLEANPEWSQILRVDIQAVYDRDPACTRFIEPILYFKGFQAIQTHRLAHWLWNEGRKDFALYLQSRSSSVFQTDIHPQVPMGQGVFFDHATGIVVGMTAVIEDNVSILQGVTLGGTGKESGDRHPKIRHGVLIGAGAKILGNIEIGHCSKVAAGSVVLKPVPHNITVAGVPARVIGETGCAEPSRAMDQLVTNFEMRKICNDWRLAVRMLCALALVFVAFAHKPITTAYAD-----PIDLAAYTLPDGTVPVLCLPRGGD-QDQH---KSAWHGTGCEACRLSASFILPVPPVASGPKVQPAKSLAIQREAILIAHSLYPPSAPPRAPPFA------------------MAKIKVDGKEIEVPDHYTLLQAAETAGAEVPRFCFHERLSIAGNCRMCLIEVKGGPPKPAASCAMGVRDLRPGPNGETPEIFTNTPMVKKAREGVMEFLLINHPLDCPICDQGGECDLQDQAMAFGVDSSRYKENKRA--VEDKYIGPLVKTIM------------TRCIHCTRCVRFTTEVAGISELGLIGRGEDAEIT-----TYLEHAMTSELQGNVIDLCPVGALTSKPYAFQARPWELNKTESIDVMDAVGSAIRVDTRGREVMRILPRVNEQVNEEWISDKTRFIWDGLRTQRLDRPYIRNDGRLVAASWPEAFDAIKAAVAAAKGDKIGAIAGDLASVEEMYALKALMTALGSANIDARQDGSVLDPANGRASYIFNPTIDGIEQADAILIIGSNPRFEASLLNSRIRKRWRASGTPIAVIGEQADLRYKYEYLGSGAETLADLAAGSVKFLSVLKKAKNPMIIIGQGALSRPDSAAVLGLAAKIAADTGALSTEWNGFGVVHTAASRVGALDIGFVPGKGGKAVAAMKGKLDVLFLLGADELDWAKTDKAFTVYIGTHGDQGAHNADVILPGATYTEKSGTFVNTEGRVQMTSRAGFAPGNAKEDWAILRALSDVLGKKLPFDSLAALRSALYAEYPHLAATDSIAAGDPADIEKLANASGSVEKTAFVSPIKDFYLTNPIARASAVMAECSALAKGGFKQAAEMSTFLSPSIMPILLLIVSNVFMTFAWYGHLKYPAAPLTPVVFGSWGIALVEYWFAIPANRIGHAVYSTAELKTMQEVITLVVFSVFSVLYLKESFTWQHVAGFALIAGGATLIFKA------MKKFGSSAIDRRAFLRGVATAGFAAATSTAFAQQSDIGDILAAPRRGNWDDQFDARSTSKGKVASFQPIASPETVSFIESAINTYSQIVANGGWPTVPATVKLKLGVIDPSVAAIRKRLMIAGDLSESAGLSNSFDTYVDAAVKRFQARHGLPADGVMGEFSLAAMNVSADVRLGQLQTNLERVRTLASTDQGPRYVMVNIPAAQIEAVEAGRVAQRHTAIVGKIDRQTPILDSNIQEVILNPYWTAPKSIIQKDIIPLMRKDPTYLSRNKIRLFDERTREEIAPETVDWNTDDAVKLMFRQDPGKINAMSSTKINFPNPYAVYMHDTPQQGVFNKLMRFESSGCVRIQNVRDLNVWLLRDTPGWDRQTMEATIKTGVNTPIQVTNPVPLHFVYVTAWSTGDGVVHLRDDIYEMDGQAALSIGTNT-----------------------------MPADANAAGDIFGGWVMAQMDLSCGIRAAERARGRVVTAAVKEMAFAKPVKIGDTLCIYTHIAHVGRTSIVLKVEAWAQRYLSDLMELVTHADFVMVALDKEGKPTPVPET----MEALLSHFDFIFQPAGLIALVTLVVMEVVLGIDNLIFISILTNKLPQEQQARARRLGISAALILRLALLFTISIIVQLTEPVFEAFGHGFSWRDMILIAGGLFLVWKATKEIHHTVDHEDAKDDVIGKAVNLTMGAAITQILVLDLVFSVDSIITAVGMTDEIAIMVIAVLAAVTVMLLAAEPLSRFIAANPTIVMLALGFLLMIGMTLIADGFGFHVPKGYIYAAMGFSALVEGLNMLARRRRKKAQAAK---------------------MNDRTCIVTRTSGSVDDMIRFVAGPD-GTVVPDFKRNLPGRGCWVKAQRRHVDEAVKRKLFPRALKNSVTVPDNFGLTVDQMLAKSALGSFGLARKAGVVVTGAAKVDAAIRNGTAALVLHAFEAAEDGVRKLDQARRSVVYAGGPEIPALSLFESSEMDLALGGVNVIHAAVLKGTAAAGFVKRAWLLQHFRDGRDDQADAKAAAAAKETETE------------------------MKSKFYLLVAMLVLAN-PAFAANSDWTKTPGGSVRLIIDTPTQPTTEIRGALQINLDPGWKTYWKEPGDAGVPPELDLTESSNIKSYSISFPTPHRFEDGGTHWAGYKRPVALPVTLTLIDPAKPVHVKGHAFLGICETICIPVTAQFDISIHGT-ASDPLTKTIIDNAFSQLPQEASATFGATSAMRKDDRVRITVSLPDEYPEPDIFVAGDGAVTFGMSKLKGREAKRAVFSAPIVAGKDKKPIQLHYTLVQGDKAVSGSIDVQEMTVKAAQLHQNDEAAS--RRPKLYSYRRIVVKIGSALLVDRTSGLKRDWLESLGDDIAALCANGVEVMIVSSGAIALGRTVLGLPKGSLRLEESQAAAAAGQIELAKAYAEVLSHHKLRAGQILLTLSDTEERRRYLNARATIGTLLRLKAVPVINENDTVATTEIRYGDNDRLAARVATMMSADLLILLSDIDGLYTAPPHQNPDGKFLPVVEAITPDIEAMAGAAASEYSRGGMKTKLDAGKIANAAGTAMIIASGTRLNPLAAIDKGERSTLFKASPTPVNAWKTWISGNLEPSGRLVIDEGALVALKSGKSLLAAGVREVAGQFMRGDTVAVFGPDKREAARGLIAYDASDAVKIAGRKTNEIAEILGYDARSAMIHRDDLVVRA-RRDTTAKE---MPVRLKKLIGTFLLVALVCIYAIVATIFAVALLGNASPWIHLLYFFGTGILWVVPAMFIISWMEKAPKKKAQ---MVRIKGH-RIRSCGLAAAM-------TLTAVLSAAATAGADTGIHVIMNQAKILKLARPADTVVVGDPEIADAVVKDARTVVLTGKGFGITNIVIMDADGAAIVDDQVMVSRSVANTTRVYRRAYVQTLSCTPYCETAQKTEAEKASDAQIGGN------MSKIRSICVYCGSSPGRDPIYKQSGKLLGKSIAEHGLELVYGGGTKGIMGAVADGVMSAGGRVTGIIPKFLMNKEATEHALGQLSELIVTEDMHERKHKMFERSDAFVTLPGGIGTVEEIVEIMTWAQLGRHRKPMVFANINGFWNPMLALIDHMKAEGFVHTSHLVNPLVVEAPEEIVPAVLSASAKGDREGNEAIIGRM-------MTHILTTENLEVVFNRFRALRGVSIEVAPGESFGLVGESGSGKSTLLRAVAGLAPVSGGTITVNGKTLGSRRTKAFYREVQMVFQDPYGSLHPRQTVDRLLLEPLAVHGFGDIEKRILRALDEVGLGSSFRFRYSHQLSGGQRQRVAIARALILEPSILLLDEPTSALDASVQAEVLNLLEQIRRDRKLTFLMVSHDLAVITHMCDRLMVMQNGAEVERLTAAELTKRKVSQDYTRNLLVASQGFVRPGASL---------------MSRQPI-----LTARGLVKRYGRVTALDHADFDLYPGEVLAVIGDNGAGKSSLIKAISGAIHPDEGEMTLEGKPIHFRSPMEARDAGIETVYQNLALSPALSIADNMFLGREIRKPGVLGSWFRMLDRKAMEKKARDKLTELGLMTIQNISQAVETLSGGQRQGVAVARAAAFGSRVVIMDEPTAALGVKESRRVLELIQDVKKRGLPIVLISHNMPHVFEVADRIHIHRLGKRLTVINPTEYTMSDAVAFMTGAKEPPA----------------------------------MI--DLPALPVTEIFPALDDALVAGNAAVLVAPPGAGKTTLVPQHLLGAPWRDNRTIILLEPRRLAARAAASRMASLLGEDVGATVGYRMRLENKVSARTRILVVTEGVFARMILDDPDLKDIAAVLFDEFHERSLDADFGLALALDVQAALRPDLKLLVMSATLDDARVAKLLG----DAPVLESKGRSYPIDIRYRPRNPDERIEDAMAKAIRDTLADETGSILAFLPGQREIERTAETLQGRVPADVMIVPLYGALEGRDQDAAIKPAPTGKRKVVLATSIAETSITIDGVRVVIDSGLARLPKFEPATGLTRLETVRASRAAVDQRAGRAGRTEPGSALRLWRAEQTAALEAFAPPEILEADLTGLVLDCAAWGVADPTNLAFLDAPPAPAIKEAKALLENLGALESN-RVTPMGNAMRALALPARLAHMVLTARARGQSQRAAELAVLLTERGLGGNDIDLDVRLSRFQRERGDRATRARGLAKR---LAG--NGSAAA-DPDSVGRLLIGA--YPDRIAKARGN-GQ-FTLANGRGGEVDPATSLAKSPWLVVADLAGRAGRTRILAAAEVTEAEIREALATSIISGRQVTYDPTRNALQARDATRIGAIALSEKTLPAPTGEEADLGVIAAVRAHGLDILPWSREATILRRRLAWLHKGLGSPWPAMDNDALIASLDDWLLPFLKGSSQLGQIPAHVVIEGLRSLVPYDLQRKIDALAPTHFEVPTGSNIPIRYEREEPVLAVRVQELFGLAAHPAIAGGTIPLLLELLSPAHRPIQITRDLPGFWRGSWADVRSDMRGRYPKHVWPEDPANASPTARAKPRGTMSKTNLHANRPLSPHLQIYKPIPTMVMSIVHRITGGALYFGTLLVAWWLIAASTNEDYFNFVNAIYGSWIGRLVLLGYTWALIHHMIGGIRHLIWDTGRGLEKETTTKMAWASLVLSVILTILVWIAAFTIA---------MNMTSTDLDRLVAEEKKLVAIEYQNEVWADGTLEGIEPEIMAEAAFATALTELIRDNGEVSALALLEALRERISAGEFSSNRVLQ---------------------------------------MPKRLYARSLIIIIAPMVLLQSVIAFVFMERHWQTVTQRLSTAVVRDIAGIIDLMDAFPQDQGYENLIRISRERLALNISILPADPLPAPGPKPFFSILDGILSEEITKQINRPFWIDTIGDSDLVEIRIQLEDRVLRVFARRSQAYASNTLIFLVWMAGSALVLLAIAILFLRNQIKPIQQLATAADSFGKGRAPPPDFKPRGAEEVRRAGAAFIVMRERIERQIEQRTTMLSGVSHDLRTILTRFKLQLALVGNKVDTEAMEQDIEDMQSMLEGYLAFARSEAEEETGTFNLERFFAKLKEEGALLERGFISSIRGEPEIHVRPNAFSRLISNLVSNSFRYAKNVSVAAEHREGWLTITIDDDGPGIPKEMRDEVFKPFFRLDEARNQDAGGTGLGLAIALDIARSHGGDITLDESPTGGLRAVVRIPAMSEIVFKGFGDKALPFLKALDFHQNREWFLENKDLFEGHLYEPLGDLVEDLLARFEKAGLPFRGDRKKSQFRIKRDTRFSKDKSPYNRHLSALLSPDGNKWAESGCFYVCIGLEEYRGCYAAIAWWQPKPELLLAMRKAIVEKPEKYRAMVRALAKNGLELNDTNRLKRTPRGFETVTDPDLIEALRNRNFVVRFPIDPAGITLPELADDLLDFAVRAKPLIDWGRAIEGTLVA----------------------MTD-VVGAP----ALPVRRPR----LSPLNARRWQNFKANRRGWWALWIFLFLFVASLFAEFIANDKPILVSYKGEILFPVLVDYPEEKFGGFYAVTDYRDPVIQDEINANGWAIWPPVRYSYRTVNNELPDTAPSKPFWLYTPEERCARYPQGVNDPNCTFGNMNVLGTDDQARDVFARALYGFRISVLFGLILTAASAVIGVTAGALQGYFGGWLDLIAQRFIEIWSSVPTLYLLLIMASILPPGFWILLCIMLLFSWVSFVGIVRAEFLRARNFEYVNAARALGVRNRTIMFRHLLPNAMVTTLTFLPFILNGSITTLTSLDFLGFGLPPGSPSLGELLAQGKNNLQAPWLGLTGFVVISLMLSLLIFVGEATRDAFDPRKAFKMDQIPLSATVDGVSAVCIRDQAFLLVERGREPSKGWLAFPGGRREEGETPQEAAIRELLEETGLVATEVSYLTTVTFDYSTAEFPSPKPFRLAVFLAHDPMGEAVAADDAASVHWLRVEDMAAFDVTTSVLEVAHHLARSFKS-------------------------------------MSDGKKGFVFALSAYLLWGVLPFYLKTVAHMPALEVVAHRVIWSVPIAGALLWWLGLFGDLKVALTTPRMLAMAVLTASLITLNWGTYVWAIGSGHAIDTALGYYINPLVNVVLGGIFLSERLSKPQILAVALAASAVILLTVSSGGLPWVSLVLAFSFGFYGFFRKTLPIGPTQGFMLEVLLLSVPALGYIFWTVSQGTSHFFNGNAHDIALLLFAGPATAVPLILYAFGAKLLRYTTIGLMQYIAPTIVFLSAIFIFGEPFSHIQFIAFALIWSALAIYTWSMLNEARKARSVETA-----MTAVNDANLDKRLNAFRPDLADESLRGMAAAERFVPGKPMRVADPVVDVRSEPRGDAGIITQLLYGDDVLVFEDDNGWCWVQNERDGYVGYVVDTTLDRRSTDPTHIVIAPRTFVYPGSDLKFPRTRALSMGSLVTVIGGEERRGTLYGMLPSGEAVIAKHLVPIDEVTDDHVAVAETLMHTPYLWGGVSGFGIDCSGLVQLSLLMTGQPVLRDTDMQATSVGELIEPDRDYHNLQRGDFVFWKGHVAMMASHSMLVHASGHTMSVTLEPLRDAIERIDYLYGQPTAVRRL-MGNRENETARRPRRSLLSFANQPDTAAETHVDP---EPVATP--PRHEDSLDHYHRMRALRLAA-GSEISQ------PAPSEIPASLTPEEAEQEAIDRMRADMAAIKAELNRRVDAAEREQVVPLH---------HVAAP-LRDEPDPVWPETPIIAEPAEP----------------------------EPAIAPAPAHRPAPRPVEVR---QAEVQSDEWRPLFDPRIVINRVARSKSLIIATTIIGTLLGVGYAMTLPKLYASTVEVLVDPRDLKIVDKEISSSQLPVDASLAIAESQLRVIQSSSVLTKVIERTGLAKDPEFNGDLADRGL---LASIGDLFSTDSTPDATARETLLLRNLYDHLDVERSTKNFIFNITVKSRDPEKAALIANTVSDVFREEQGNIQSDTARQATESLTARLNNLRTGVEAAENAVQKYKADNDLVDVQGRLISDDEISRTNDELTAARNQTIRLNAQAASIRNASVEGVL--GNTLPEEFRSGVIVALRSQYGALKQQADGLATKLGPRHPQLIQAQSQVNGIRSEIRYELSRIASSVQVELKRSVQQEQDLAARLAQLKARTASNNEDMVKLRELEREATAKREVYEAFLLRAKETGELESMNTTNIRVISPARPALESTGGSRKLVAVAGLLGGLLTGLGIAILLGMIDSFRAGNRPAVE-------DGAPVDPTPPSGGTRTKPEE---PAP-AAPSESRLGAAIRRAQS-RTMALD---------EVEDESVDDEVAIAEFLYAYADMMLAP-RHHDNSNIEDMLSDIAEIREALRLRAGRQAS-MSYTNFKFDVDADGIALITWDMPDKSMNVFTEEVMKELDAIVDRVTQDEAIKGAVITSGKDTFSGGADLTMLKRMFQLFQEEKAKDPKKAVELLFENTGKMGGLFRKLETCGKPWVSAINGTCMGGAFEMSLACHGRVVSDDPSVKMALPEVKVGIFPGAGGTQRVPRLTNQQDALQMMTTGSSLTASRAKAMGLVHEVVPAKKLVDAAKKMIKAGLKPVQPWDEKGFKLPGGAIYSAAGANLWPAATAILRRETYGNYPGAASILKSVYEGLLVPFDTALKIEQRYFTKILQTTEAGMMIRSLFVSLQELNKGARRPADVKPTKFKKIGVIGAGFMGAGIAYVTAKAGIPVVLLDQSLEAAEKGKAQSAELITKEMQRGKATAEEKEKLLGLITPSADYADLEGADLVIEAVFEDREVKRIATEKAEAVLKSAAIFASNTSTLPITGLSKVSQRPKNFVGIHFFSPVDKMLLVEVILGKKTSDKALAVALDYVRAIKKTPIVVNDTRGFYVNRCVLRYMSEAYNMLVEGVPPAMIENVARMAGMPVGPLALNDETAIDLSQKILKATLADLGPKSVDPRHVELVNTLVDKYDRRGRKNGKGFYDYPAKPAKKQLWPELKTLYPQQDPDKIDVNVLKQRFLFTIALEAARVMEEGIVTDPREADVGSILAFGFAPYTGGTLSYIDGMGAAKFVEIAKGLQRKYGAQFKAPKLLLDMAEKGDTFYERFNPYPVEEKKKAA-MIPGEIIPATGDIELNTGASDITLKVANTGDRPVQVGSHYHFFEANPALEFAREQARGMRLDIASGTAVRFEPGQERDVALVPLGGNRTVYGFQQKIMGKLMFSAALSGFLLGASLIIAIGAQNAFILRQGLLREHLFILSLICALSDALLIAAGVAGLGTLIAQSPTLISFVTLGGAVFLLWYASIAFRRAFHPEVLQAATSGNGNLKVAISTVLALTFLNPHVYLDTVVLLGGLSARFEGVNRAAYGAGAALASFIWFFGLGYGARLLQPVFAKPAAWRVLDVLIGLVMAGIALSLLVSFLNN-MNSPSPPDPPDVRPQRASGSARLAVKPVNGHTRIATLYQEGCAKIRLPETDDRSLEAVMINTSGGLTGGDEMQWQFDIADDCTAQITTQACERIYKASAD-VAHVTTKLTAGERASLAWLPQETIVFEGSALQRSLTVELAGTSRALIVEPIVFGRKAMGEDVEKCILRDRWRIQRNGVLIHAEDFSIGPDAASMLGRPALFGGMRTMATVLLLDNDAERLLDASRKIIGKKGGASFWSGKLLARLIDGDAYSLRKRLVPLIELMNEKAGVPKVWSIMEA-LLPIIIQLISGGVGGNIVGGLLKNLSLGTTGNTVAGGIGGLIGGYLTQFLGAGGAEMVANAAASSGLDLGSIIGQVAGGGVGGLILTAIVGAIKNSMA-KTMNAILKATLSRLIHTGTLIITDSSGKSRTFGDGTGDPIHFRINSAAAERKIAFDPSLHFAEAYMNGELDVLDGDIYDVLKIIFENTGATVAREPWMLAIEGIRRATRRLHQMNTLTRASSNVQRHYDLSEDLYRLFLDTDMQYSCAYFERPDATLEEAQMAKKRHIAAKLLVEKGHKTLDIGSGWGGLGLYLAKHLKADVTGVTLSQEQHGIANERAFEEGLTAQARFDLRDYRTIEESFDRIVSVGMFEHVGIGHFPEYFHHTARLLKKDGVFLLHTIGRSDGPSYTNAFIQKYIFPGGYIPALSEVIPHIEKAGLIVTDVEILRLHYADTLRIWRERFMVNREMAKAIYDERFCRMWEFYLAASEAAFRWQNLVVFQIQLAHRQEAVPLTRNYI-EKEEKRLKRLECERSSANSQVE-TKETAAGRMIRFGAYMFSFALRS---IAVGLLTLSMGTLA-------------GTSMAAQQSGTVKSTHGAWSILCDTPAGAKSEQCALIQNVVAADRPEMGLSVVVLKTADNKAKILRVLAPLGVLLPNGLGLNVDGKDIGRAYFVRCFEDGCYAEVILEDQLVQTFKTGKAATFIVFQTPEEGIGIPVELNGFGEGFDALPMSHYAEQATMLGKDVKIPQHPDEAMLDAVP-NPQAGTLFLARFACPEFTSLCPVTGQPDFGHLVIDYAPDQSLVESKSLKLFLGSFRNHGAFHEDCTVNIGKRIVEAVKPLWLRIGGYWYPRGGIPIDVFWQTGPVPEGLWLPDQGVPPYRGRG-------------MPMISRRQLLIGAGSVAVFGASGCTQTTFEMP--ELNIDAMPTSGIRPQISIDKSVTVPDVMYAAVQEGPYSLPAIPYQKIPAQFRRQIVVDPTGEQPGTIVVRLQEKYLYLVQPGGDAIRYGVGIGKAGFLWNGRANIQYKKEWPRWTPPREMIQRKPELAKYEHGMEPGPENPLGARALYIFKDGVDTGYRIHGSPEWWSIGQSMSSGCVRLINQDIIDLYNRVPGKATVVVGMTETV------KPTPEAIRQARAGNPKMRERDFAQQLQISEADLVAAWCGINVIRISPRIETFLTEIQSLGEVMALTRNESAVHEKIGVYDKPIVGKHASLLLGEQIDMRLFPSAWAHGFAVEKAEGE-TVRRSLQFFDAAGEAIHKIHLRPASSLDPYNVLVAKLRLDDQSQTIELTSLPKPEEKILEIEELDTSSLRDRWSQMKDVHEFFGILRSLKMTRHQAVQVIGEDYAWQLDTSALTAMLHHSAQEQIPIMCFVGNRGCIQIHSGPVQNVTPMGAWINILDETFHMHLRTDRIAEVWAVRKPTKDGHVTSLEAYGAEGEMIIQFFGKRHEGSGERSDWRRLIEGLPRLMCAAA---------------MKKIALAALAISLIATPAAFAQQQNYR------GHGYGHQQ-----KEVIVKKKVVVKKT----RWSRGHALPSSYRRNVVRDYHRYHLRTPPRGYQWVRADNDYVMISIATGMISTLVQIRMTSNSKYFDSIRIRPKKAEEEKSRTPVCQWDGCDKPGPHRAPVGRMKEGEFFHFCIDHVREYNKGYNYFSGLSDAEVARYQKEALTGDRPTWTIAGLPGSGAGASTQ--AASSGGSKASADFSRLRSGRAAYQNRFRDPNSVFNEAR--V----HVRKPKPLEAKALDTLGLGAKASGEDIKSRYKELVKRHHPDANGGDRASEDRFRDVIQAYQLLKQAGFCMAPTDT----------------KAYTFVDHKFDVVVVGAGGAGLRATLGMAEQGLKTACITKVFPTRSHTVAAQGGIAASLSNMGPDSWQWHMYDTVKGSDWLGDTDAMEYLAREAPAAVYELEHYGVPFSRTEAGKIYQRPFGGHMMNYGDGPPVQRTCAAADRTGHAILHTLYGQSLRNNAQFFIEYFALDLIQDEDGTITGVVAWNLDDGTIHRFAAKMVVMATGGYGRSYFSATSAHTCTGDGGGMVARAGLPLQDMEFVQFHPTGIYGAGCLITEGARGEGGYLVNSEGERFMERYAPSAKDLASRDVVSRCMTLEIREGRGVGKNKDHIFLHLDHLDPAVLHERLPGISESAKIFASVDLTKEPIPVLPTVHYNMGGIPTNYWGEALNPTKEDPDRIQPGLMAVGEAGCASVHGANRLGSNSLIDLVVFGRAAAIRAGQVIDRNSKVPDINEASVTKIMDRFDRLRHANGGTPTAALREKMQRTMQEDAAVFRTSESLKSGVDRMEKIWSELPDIKVTDRSMIWNSDLVETLELENLMANALTTVVAAEARKESRGAHAHEDFPSRDDVNWRKHSLAWLAPDGKVTLGYRPVHLDPLVKEEDGGISLAKIAPKARVY-MKIALIGATGFVGQEILKEAVSRGDSVTALVRNPDKVEKLAGVTAIEADALNTADLASKLKGHDIVVSAYNPGWGNPDIKAIHIAAS--KSIAEATKQAGVGRLIVIGGAGSLYAPDGSQFVDGEGFPAEYKEGALGARQALNDLRGETELDWSFVSPPFHLAPGPRTGNYRLGKDNPVFDAEGNSAISASDLAVAIMDEANTPKHAKQRFTVGYMSIERYDMHPVRDATTDVDHHAEVARQFAASDKDSTVFRAAERHSRRVRFLKFALPAAALLGAAVFSWFTFFSTSSVPSNISLDNAGIEDGKLVMTNPKLDGFTKDKLPYKMSAVRALQEVGNSNVISLEGIDAEIPLGTELRAQVKAKSGVFDNANRQLTLDSDISLTTSDGITARLQSADIDIAGNSMSTDQPVSINNSNSQITADSMQITESGKVMTFEKRVRLVIQPAKLQEGGKEVKSPE-MTLFSR----QSRCSTFVLLSLTACIASATLISSTDDADARHRRGHRAKVVRAPQPPAIALPAVPPLPTDNPR-AAAPEAPKPVEKPAEEMQKADPQ-TPLPDQKPA---PEAKPAEKPKEPPKPGEQFGPNPLPKQGA----AGKDEHEEIVEKADPSISPDRVYQNACPALMNGEVKGELIAPLSEGMCGERSPLKITAIGKDNPVKLAAPITTNCAMAGSLATWVIEIQKEAQANFGAEIESITTGSDYQCRKVNNGHKGRVSEHAFANAVDIVSFKFKNGKTTELGSGWKGKPEEQTFWRALHKASCDRFMTVIGPDGDAAHQGNLHLDLGCHGRKCEARLCQMAGSVNKVILVGNLGADPEIRRLGSGDPVVNLRIATSESWRDKNSGERKEKTEWHSVVIFNDNLAKVAEQYLKKGMKVYLEGALQTRKWQDQTGNDRYTTEVVLQKFRGELQMLDSRGENA--GGGRSFDNSSGRG--QVSDN--DYGSDFGRSSASSS--SSSGA--GGNFSRDLDDEIPFMASDPFDQLEGYIWMNGEFVRWADAKVHVLTHGLHYASSVFEGERAYGGEIFKLNEHTERLHESAKILGFKIPFTVEELNDACRKLLAKQGFQNAYVRPIAWRGSESMGVSAQANKINVAIAIWQWPSYFDPAQKLKGIRLDLAEYRRPDPRTAPSKSKAAGLYMICTISKHAAEAKGYADALMLDWRGQVAEATGANVFFVKDGVIHTPTPDCFLDGITRRTVIDLAKRRGYEVVERAIMPEELDSFEQCFLTGTAAEVTPVSEIGPHRFTVGEIAINLMNDYAAEVMPKKAAAEMGIASIIDTHLHLIDQSTLSYPWLSSVEALNRDFRYEEYALQAHRVGITQALHMEVDVAPSDIEAESGYIAGLAKREGSLIAGCISSCRPEEDGFAAFLERQQANPLIKGFRRVLHVMPDNLSESALFRENIKQLEGTGLTFDLCVLPSQIEKAIALIDLAPGVQFILDHCGVPDIKSEGLSEWHSKITDIAKRPNVTAKISGVVAYADSDTWRIETLEPYVHHVIESFGWDRLVWGSDWPVCTLGGGLATWVAATHALLQGCSEAEKAKLFSANAKRIWSIA--------MLTHVLTYAPEEPVHDDAIEDINKEAFGPGRFTRAAYRIREGGPHDPKLSFVALNGEHVIASVRLTPILIGFTPAMLLGPLAVRPAWKNQGIGAALMRTSMEAARLAGHSLVILVGDEPYYAPFGFRQISGHQIEMPAPVDPARFLACELTPRSLQNVQGKVRHADSGGI----MKKIEAIIKPFKLDEVKEALQEVGLQGITVTEAKGFGRQKGHTELYRGAEYVVDFLPKVKVEIVLGDESVEAAIEAIRKAAQTGRIGDGKIFVSNIEEVVRIRTGESGIDAIMVKQRSKW----------RHRLFHTYFLFHRPMTLGARGVVIDDATRSVFLIRHTYVPGWQFPGGGVETGHTIEQTLAKELMEEGNIELTRRPELFAIYHNAHASRRDHVALYICRSFRQTTPFAPTREIAEAGFFKLDALPEDTTPSTRRRLAEILDGVEVPLTW-----------------------------------------MRFACGAASSFLILIAVLAGIAFVILRVGIGGEALTTRAQLALKTALGPEVDATLQSAHISLDKRRHVALEARNVNIADTKRGIEIDDVRSVRIGLATLPLLQGDVKVERLELDGAHIKV-TSQVPFDFMALVPLDDRGLVNLDAATNVIFAGLETAVSLLDQRETRDITIADTSFDFAMAGKPEQLHIVNLDLSETGGPVAISGTVEWRGKAIEVTATAKRSAAGAKVEAFSLAISNIPLTGKFGKAP--VPDVDGIKPDGAYLAYDNQAEIKLDGTAATASEPARLSGTLNIGDGPVTIGNVDDMPVETQISFEHVSGTDKVQIRPSNIHFGAFKGVVEGKISPVSRSTGSA---PPDFPGYEFELKTSQAISAPQDSTEPPMPFDARLAGWYYPEEKQLEVSEIAVRGNGESELYGRAGATFGKGSPAMTLGLHIARMPVAHAKQLWPVWVATGARRWVLENLHGGTAKDSRIDVVFAAGRFDGPGKPPPLKPDEVQVDFAVENSRFDIVGDLPPVRNADGKISVRGAYTTINLEKGSSFTPNNREARIINGTLIIPWGPQRPVLSELDLNLEGDASAVAEIIGFKPINGLKNLPFAPEDIKGNVKTHVLVTFPVTRRSPPGSLKWSADMALSDVSISKKIDGQIISSADGTLSVTQDEARIDAKAQLNGIPADVSLFEPLGNNTAKREQSVTLQLDDKTRNALFPALDPLLSGPISVDIGKAADGSRLATADLTKAEIKLAPLGWTKGAGVKASAKFALQQDG---DNATIKDLDVSGDTFRLRGDVNVVNGDLASADFTDVRLNRGDDIAAKVAR-TKYGYRVDVNGGSFDARPLLNQITDKKKKGA--ASDSTQRVLVNADIDEVAGFYSESFRNVSLSYEGVGSNVSGLAFNAITKSGQKANATDSSESGARSISLQSRDAGAMLRFFGFYDKMSGGSISVALDSSGDGPLRGQVDAQNFTLVNEPRLAKLVSTSPS--GTSLNEAVQKNIDVSKVTFDRGFSQIEKGSNYINLANGVIRGSTIGATFQGVLSDPQGNMSLTGTFMPAYGINRIFGEVPILGLVLGNGRDRGLIGITYKLQGPLKQPQIFVNPISVIAPGIFRSIFEFQ-MNIAVDSLGENLFGPMGSPRDGSEVFAALTKAARERILILDGAMGTQIQGLNLNEESFRGDRFADCSCHLQGNNDLLILTQPQAIEDIHYAYAMAGADILETNTFSSTTIAQADYSMEEVVYELNRDGARLARRAAIKAEQKDGKRRFVAGALGPTNRTASISPDVNNPGYRAVTFDDLRIAYAEQVRGLIDGGADIILIETIFDTLNAKAAIFATQEVFEEKGITLPIMISGTITDRSGRTLSGQTPTAFWYSVRHAKPFTIGLNCALGANAMRDHLAEIASVADAFVCAYPNAGLPNEFGQYDESPEAMAAQLEEFAREGLLNIVGGCCGSTPEHIKAIAEAVGKYGPREIPETEIHMRLSGLEPFTLTKDIAFVNVGERTNITGSAKFRKLITSGDFATALDVARDQVANGAQIIDINMDEGLIDSEKAMVEFLNLIAAEPDIARVPVMIDSSKWDVIEAGLKCVQGKPLVNSISMKEGEQAFIDHAKRVRAYGAAVVVMAFDTQGQADTLERKVAISTRAYELLTREAGFPPEDIVFDPNVFAVATGIEEHNGYGVAFIEATRQITETLPHVHISGGISNLSFSFRGNEPVREAMHAVFLYHAIQVGMDMGIVNAGQLAVYESIDPELREACEDVVLNRRDDATERLLDLAERYKGAAGKEARERDLAWREWSVEKRIEHALVNGITEFIEADTEEARLEAERPLHVIEGPLMAGMNVVGDLFGAGKMFLPQVVKSARVMKQAVAVLLPYMEAEKLANGGSGQRESAGKVLMATVKGDVHDIGKNIVGVVLACNNYEIIDLGVMVPSAKILQTARDEKVDIIGLSGLITPSLDEMVHVAAEMEREGFDIPLLIGGATTSRVHTAVKINPRYHKGQTVYVTDASRAVGVVSNLLSLEAKPGYVDGVRAEYTKVAEAHARNEADKQRLSLAKARANAQMVDWVSYTPPKPSFLGTRVFENYDLAEIARYIDWTPFFQTWELKGRYPAILEDEKQGPAARQLFDDAQAMLKKIIAEKWFNPKAVIGLWPAGAVGDDIRLFTDEARGQELATFFTLRQQLSKRDGRPNVALSDFVAPVDSGKEDYVGGFVVTAGIEEVAIAERFERANDDYSSILVKALADRFAEAFAELMHQRVRKEFWGYAPEENLASDDLIGEAYRGIRPAPGYPAQPDHTEKETLFRLLDAEAKIDVRLTESFAMWPGSSVSGIYIAHPDSYYFGVAKVERDQVEDYAARKSMPVADVERWLGPILNYVPAPR---VQAAE--------------------------------------------------MDPIVNKLSAVLAIPFLVLSASVASPHKD-THFVEPKLYIARGGNITPQVALTLDACSGQTDMRMLNALVENHIPATIFVTGRWLKHNAHSVAIMKAHPDLFELENHGLNHIPAIDNQPTMFGLKTAGSLAAIRTEIEGGANAMTAATLTKPAWYRDATARYSDDAITLVHQMGYRVAGYSLNADVGASLLAGQVEKRISAAKDGDVIIAHVNQPTRVAGEGVVKGILALKQKGFHFARLEDVQESE--QPAR--------------PGV----------MQGQKPHDGKKIPATVITGFLGSGKTTMIRNLLENANGKRIALIINEFGDLGVDGGLLKGCGIEACRDEDVIELNNGCICCTVADDFIPTMTKLLDRPDRPDHIVIETSGLALPQPLVAAFNWPEIKTQVTVDGVVTVIDAAAVSEGRFADDHDKVDAQRVQDESLDHDSPLEELFEDQIRAADLIVLNKTDLINGDQLDGVKGDVQSRSSRALNMVPASFGKLSNQLLLGLGVGTEEDIANRKSHHEMEHEAGEEHDHDEFESFVVGLGPVAEPKSFVEKLKGVIADHDVLRLKGFVDVPGKPMRLVVQAVGSRIEHYFDRSWHPDEERQTRLVVIGLHDIDTAAIETAMNAAAA--------------------------MPGRDEILQFIKENPNLAGKRDIAKAFNLKGEARIALKDILRDLADDGLVEKRAKRLTMPGSLPSVAVLDITGREGDGGLIAKPAEWDVNHQERPPVVFIRASRNQKGPTPGVGDRVLARIFVSADPSGPAYTARVIKRIEHRKDAVLGVLRKLDNGEWRLEPVERRQVELSIDPAQLNNAKVNDLVEVEVSKVSRYGLALGRITQVVGSVASEKALSMIAIHEHEIPHVFPDSVIVEAEKAKSATMA----HREDWRHVPLVTIDPADAKDHDDAVYAEPDTDEKNPAGFIATVAIADVAAYVTPDSQLDREALKRGNSVYFPDRVVPMLPERISNDLCSLKELVDRPALAVQMIFSSEGRKVSHSFHRIMMRSHARLSYPQAQAAIDGGPDEKTAPILDTILKPLWAAYATLKRGRDAREPLELDLPEKKILLTPEGKVDKVVIPPRLDAHKLIEEFMIQANVAAAETLERK-----KQPLIFRIHDAPSLAKQETLREFLRTLDISLARGAELR-PNQFNNILKRVEGSEQQDLVNQVVLRSQSQAEYNPENIGHFGLNLHRYAHFTSPIRRYADLIVHRALISALGLGPDGITAKEEAALPEIAALISTSERRAMAAERDTVDRLIAHYLADKVGSSFEGRVQGVTKSGLFVSLATYGADGFIPISTLGDDYYLYDETNHALSGERSGKGFRLGDIVEVRLVEALPVAGALRFEMLSEPHPVPSGVRSHHKAKRFVRGNQKRPTSVSR---GGRRGRG--------------------------------------MPDGRIRQVAALVYRVKKNQPEILLITSRGTGRWVLPKGWPQIGKTFAQSARAEAFEEAGVRGDIAPFSIGTYTYAKHDMSDGEVGDFIVDVFPMHFSHQQKNWPERGVRKLEWLSVEESVRRVEEPELKALLAGFDP-ALVDVR--MRFNIPIVALVLLATSVSF-SAHAHDHSKM-SKGNAGAM-----------------------------------------------------------------------------------------------------------------------------------------------------SDD-TMQ------PVKEGELVLSGAFTRATLPGAKVGGGYLSISNPSKEDDRLLGGSSPAAARVEVHEMKMDGNVMQMRQLKDGLKIPAGGTAELAPGGAHLMLMDITKPLKQGDMVPVTLEFAKAGKVDVQFMVGPANATSMEHH-MARQEQA----NDIFALTSFLYGGNAHYIEELHASYEKDPASVGPEWQEFFANLNDPKEDVLKNAKGASWEKPNWPIAGNGELVSALDGNWGEVEKHITDKLKAKSANGAA-AANGVA-----APA-VTA-SDADIIHAARDSVRAIMMIRAYRMRGHLHAKLDPLGLAEVKEDYNELSPESYGFSPADYDRKIFIDNVLGLEYATIPQMLDILKRTYCSTIGVEFMHISDPVEKSWIQERIEGPDKGVAFTAEGKRAILSKLIEAEGFEQFIDVKYKGTKRFGLDGGESLIPALEQIVKRGGSMGLKEIVLGMAHRGRLNVLSQFMRKPHRAIFHEFKGGSYTPDDVEGSGDVKYHLGASSDRE-FDGNKVHLSLTANPSHLEIVNPVVMGKARAKQDLLAGR-ERDEIVPLSERSKVMPLLLHGDAAFAGQGVVAECLGLSGLRGHRVAGTVHFIINNQIGFTTNPRFSRSSPYPSDVAKMIEAPIFHVNGDDPEAVVYVAKIATEYRMTFHKPVVIDMFCYRRFGHNEGDEPAFTQPIMYKTIRSQKTTVQLYSTKLIEEGL--INEAELEKMRAAWRENLEQEFEAGQAYKPNKADWLDGAWSGLRTADNADEQRRGKTGLPVKTLKEIGKKLAEVPAGLNVHRTIQRFQDNRAKMIETGEGFDWATAEALAFGSLVTEGHPVRLSGQDVERGTFSQRHSVLYDQDTEQRYIPLNNLQKGQALYEVINSMLSEEAVLGFEYGYSLSDPRALTLWEAQFGDFANGAQVIFDQFISSGERKWLRMSGLVCLLPHGYEGQGPEHSSARLERYLQLCAEDNMQVANCTTPANLFHILRRQMKRDFRKPLILMTPKSLLRHKRAVSSIGEFTGDSSFHRLLWDDAQLLKDQPIKLQKDSKIRRVVLCSGKVYYDLYEEREKRGIDDVYLLRVEQLYPFPAKALITELSRFRNAEMVWCQEEPKNMGAWAFIDPYLEWVLAHIDAKHQRVRYTGRPAAASTATGLMSKHLAQLEAFLEDALGSMCASLAGIGEPMQVEPEKARDDQEAAKAAIPSAVLFICGMNSIRSPIAETLARSLLPSTVYIASAGVEKGEPDHFVDEVLAEIGLKRISALPKTFDELEDHYFDLIITLSPKAHHMALELTRTTSVHVEYWPTPDPTLVRGRRSQILDAYRDVREHLRRHIQERFGQPF----------MREVPAMQTIALVDDDRNILTSVSIALESEGYRVETYTDGASALDGLMARPPNLAIFDIKMPRMDGMELLRRLRQKSDLPVIFLTSKDDEIDELFGLKMGADDFITKPFSQRLLVERVKAVLRRVAARETA-AKPGSPQAKSLERGQLVMDQERHTCTWMGEPVTLTVTEFLILHSLAQRPGVVKSRDALMDAAYDEQVYVDDRTIDSHIKRLRKKFKVVDDDFDMIETLYGVGYRFRET-MSKKKPVDPQSLPYRPCVGIMVLNKQGLVWAGHRIVIANDEMDGATQLWQMPQGGIDKGEDAEPAALRELYEETGMKTVTLLAEAPDWINYDLPPHLVGVALKGKYRGQTQKWFAYRFEGNESEIAINPPPGGHTPEFDRWAWKPMAQLPDLIVPFKRTVYEQVVATFRHLARS-MVALSETPQLWGLATAQLWRRFRRRVRMGPLYRWRFTGFTPERVLIAPQDLRPADPQLAEEFYHGRFALAGKVVETGGRSPFLIEPPNAAWQAALHNFGWLRNLRAAGTELATANARALLGDWITTHGRSIGGPAWAPEVTAQRIIAWLQHSNLILAGADLRAYRQFMRSLAVQVRYLRTMAAVMDDGEEKLRARIALALAALALPVASSTSRSARRNLEFELVRQILPDGGHISRNPLTVLELLADLLPLRQTYASGQEAPPKALIEAIERMLPALRFFRHHDGSLALFNGVGATMPERVIAVLRHDDIGGLPLIHAAHSGYERLSMDSTTIIADTGLAPPVTASRDAHAGCLSFEMSSGRNRYVINAGVDRYGPPEFRPLARSTAAHSTATINDTSSCRFSNTSGLSNMIGTPIIAGPTKVNIERIEREGVIGFVANHNGYVRPFGIYHERQIVLSHNGSVIEGIDSFFAAGRNPLRDTGAEDVVIRFHLHPSVAITVDDNGLIVLQAENDDIWMFSAT-VQPFIEDSLFFAGFRGPVKTRQITLSLNASQRSEVEWQFTRTTLGVYG---MQRRQWVKMAVAAVAATWLGISSVAAPAQAAEKVTVFAAASLKNALDDITAAWRQETSKDATISYAASSALAKQIESGAPADIFISADLDWMDYVEKKNLIKKDTRTNLLGNRIVLV--APK-DNAEPVEIIKGFDLAKLLGDGRL---AMGAVDSVPAGKYGKAALESLGVWSSVESKVAGAESVRAALLLVSRGEAPYGIVYQTDAAADKEVKVVGTFPEDSHKPIIYPIAVL-SESKNADAVSLFDYIKSDKAVPFFEKQGFTMPK----MSLVVNEREKQLSRDRFGLSAALVTPFDAKMRVDVPKAIAHARHCLNSGCNSVTLFGTTGEGSSIGDDEREAILKAFLDAGVKPDQIVVGVMANSYLEAAAQAGAALDAGCRAVLLAPPSYFKNVSDDGLFAWFSAVFAQIGASARDIIVYNIPSVTAVEISVDLISRLREAFPAVISGVKDSSGNWAYTENLLAKHKDLAILIGDERDLAAGVRRGGQGAISGMANLYADRLLPMINEGRDDTAMVEAVQELLKFPVVPAVKALVAHHTGDAGWRRARAPLQALPDSDFSRVTSIFDRLFVAAAAMAEKSQKLQNLTEEEINQRHADKMRKKKAVRDKILATKTDEKGLVIVNTGKGKGKSTAGFGVVFRALGHGMKIGVVQFVKGSWDTGERWVLEKFPEQVTISAMGEGFTWETQDRSRDIAMARSAWEQAKTMILDDETELVLCDELNIALRYDYLPVEEIIEVLKQKPHMKHVIITGRNAKDELIEFADLVTEMEMIKHPFRSGIKAQKGIEFMRVDLFDFDLPEENIALRPAEPRDSARLLLVKADASGFEDSTVRNLADFLRPGDALVFNDTKVIPAQLEGFREREGVNAQISATLHMRAGPDRWKAFLRPAKRIKQGERIDFGHGNNACLLGSLQATVTEKGDAGEALLVFDFSGADLDQAIAAVGHIPLPPYIASKRADDIRDREDYQTVFAREEGAVAAPTAGLHFTPELLDNLKRRGIEEHFVTLHVGAGTFLPVKAEDTADHKMHAEIGTVDQTTAEALNAVRARGGRIVSVGTTSLRLLESATDEHGVIKPWSGPTDIFITPGYNFRAVDMLMTNFHLPRSTLFMLVSAFSGFERMHAAYDYAVKNHYRFYSYGDASLL-------WRSASMTVRFGLLGAGRIGKVHAKAVTSNPEAKLVAVADAFEQAAKDLSAAHGCEIRTIEEIEKASDIDAVIICTPTDTHADLIERFARAGKAIFCEKPIDLDVKRVEECLAVVRETGTTLMVGFNRRFDPHFAAVRKAIDDGAIGDVEMVTITSRDPGAPPAEYISRSGGIFRDMTIHDFDMARFLLGEDPVAVTAHASVLVDKKIGELGDFDSVSVILSTASGKQCVISNSRRATYGYDQRIEVHGSKGMVQAENQRAVSIEVANGNGYTRPPLHDFFMTRYTEAYANEIAAFINAIATGTKAAPSGEDGLIALALADAAVRSVKEGRTIKL-----MSERLEDIAIAMVANGKGLLAADESTATIKKRFDTIGLESTADSRRDYREMLFRSDEAMKNNISGVILYDETIRQSANDGTPFVQIMKAAGCIPGIKVDAGAKPLAGYPGETITEGLDGLRERLQEYYGLGARFAKWRGVIGIAEGIPTWGAVKQNAQALARYAALCQEAGIVPIVEPEVLMDGKPATHSIERCYEVTEWVLKTVFDELYDARVKLEGMILKPNMVIDGKNARKASVEQVAEQTVRVLKSTVPAAVAGIAFLSGGQSDEEATAHLSAMNANFTTPWKLTFSYGRALQAAAINAWNGKPENVAAGQRAFTHRAKMNGLAATGGWKKDLEKAA----MHRIEGDVEIFDTAGGVTISRSRIATAYASAIDSYVDALDERRGAVFSSNYEYPGRYTRWDTAIVDPPVVITSNGRHMTIEALNKRGEILLAPIRQAIAALEEVSLLSAGVTRLELEIALPSRHFTEEERSRVPSVFTVLRAIVALFFTEDDANLGLYGAFGYDLAFQFDPIEYKLKRPDDQRDIVLYLPDEILVVDHHAAKAWLDRYEYAFDGASTEGLPRDTAAEPFKPTDVIPGRGDHHPGDYAELVKKAKDSFKRGDLFEVVPGQTFYERCETSPSVISRRLKAINPSPYSFYINLGNNEYLVGASPEMFVRVVGRRIETCPISGTIKRGEDAIADSEQIIKLLNSKKDESELTMCSDVDRNDKSRVCEPGSVRVIGRRQIEMYSRLIHTVDHIEGRLREDMDAFDGFLSHAWAVTVTGAPKLWAMRFIEENERSPRAWYGGAIGMVNFNGDMNTGLTLRTIRIKDGIAQVRAGATLLYDSVPEEEEAETELKASAMIAAVREAHKSNVLPEERAVARVGEGLSILLVDHEDSFVHTLANYFRQTGATVSTVRTPVPDEIFDRIKPDLVVLSPGPGTPEDFDCKATIKKARKRDLPIFGVCLGLQALTEAYGGTLRQLHVPMHGKPSRIRVTKQGKIFSGLPKEVTVGRYHSIFADPVRLPDEFVVTAETEDGVIMAFEHKSEPIAAVQFHPESIMTLGHNAGMRMIENVVAHLPRRAKERAAMNVMRTAMLLAFMTALFMGVGFLIGGTGGMMIALVIAAGMNLFSYWNSDKMVLRMHHAVEVDEQNAPEYYGIVRDLAARAGLPMPKVYLIQNDQPNAFATGRNPQNAAVAASTGLLQRLSPQEVAAVMAHELAHVENRDTLTMTITATLAGAISMLGNFAMFFGGGNRENSNPLGFIGVLIAMIVAPFAAMIVQMAISRTREYAADRRGAQICGNPLWLASALNKIARGAQQIVNEDAERNPASAPLFIINPLSGRGADNLFSTHPSTDNRIAALEAMVEEFRQ-------APPAP-RQANAPEPPIDDDEPREDRPNPWG-----RDGASRKGPWA-------------------MDQLAPSEFSPYRFFSAERWAEFRADTPMTLTYDEVKRLRSLGDPIDLDEVSRIYLSLSRLLSAHVEASQLLFYQRRRFLNTDDAFKTPFIIGIAGSVAVGKSTTARVLKELLARWPSSPKVDLVTTDGFLYPNEVLRAKGLMERKGFPESYDVGMVLRFLSQIKAGQRNVNAPLYSHLTYDVLPGMFQTVDAPDILIFEGINVLQVRDLPEDGKMVPFVSDFFDFSIYIDADEEQIHKWYISRFMRLRETAFKDPSSFFHRYSTISEDSARAIGESLWANINLKNLRENILPTRPRADLILRKGDNHLIEEVALRKLMVEAFMREKTP--DDR-LKLADAIRGAQVSAADRGDIAMDAKEADLARIEILADDLRPVFEDVPIDDLQWDFAVSKGGQPRLWIDATSHVMMGRDRRTYRFVRDTRLGRVVVAESTEVRPIADAVTNYIAERIVERQRLMEGQRVSLLPA-------EPSNDADSSARGLVYGA----PKR-TRLSAFVTATFWFLIGASAGAGALLLLFWDRLAPFIKPD---------MD-NNLSFVRTEMLQGQTPPLGERGIVRWMRTNLFATPLDTALTLLAILALAWFLPGIINWLFVNAVWSGPDRSVCSTVAQGGIQPEGWSGACWAYVNANFNQFMYGPYPVDQHWRVNLVAIIFVLLLVPLLIPKAPYKVLNAVVFFFIFPIVAFILLVGGWFGLPYVETSSWGGLLVTLTLSFVGIAVSLPLGIVLALGRRSKMPVIKMLCVIFIETIRGIPLITVLFMASYMLPLFLPPGVSFDRFLRALIGVALFASAYMAEVVRGGLQAIPKGQYEGADSLGLGYWQKTGLIVLPQALKLVIPGIVNTFIGLFKDTSLVTVISMFDLLGTVKQHFSDANWISPQTPISGLIFAGFVFWIFCFGMSRYSIFMESRLDTGHKR---------MMTKQH------FGTSHVPLSPAVRAGDFIYVSGQVPVGSDGTVIIGGIEAQTKQVMDNVKAALALAGAELSDVVKTFVILEDAREFAAFNKVYATYFQKDPPARTTIESRLMIDIKIEIEATAYKPL------------------------MRTDTGQVFHLDDYKPTDFLIPETHLDFALHPERTVVKSTLQIERRPGTPADVPLILDGDELKLVDIRIDGAAPLGDNTFLATPDRLEIRGLPASGRFTLEITTEVNPTTNRQLTGLYRSSNVYCTQCEAEGFRRITYFLDRPDLLSIYTVRIEADRKEAPLLLSNGNPKDKGTLTNGRHFAVWHDPHPKPAYLFALVAGDLGVIHDTFTTASGRHVDLGIYVEHGKEPRALYAMDALKRSMKWDEDVFGREYDLNVFNVVAVSDFNMGAMENKGLNVFNDKYVLADPETATDTDYAGIEAVIAHEYFHNWTGNRITCRDWFQLCLKEGLTVYRDHEFSADMRSRPVKRIAEVKGLKAHQFPEDAGPLAHPVRPRQFREINNFYTSTVYEKGSEVVRMIRTILGPDLFRQGMDLYFERHDGDAATIEDFIKVFEDASGEDLSQFALWYDQAGTPNLAITYDYDPATKTFEIEIEQSLKPTPGQPSKKPMHIPVQFGLLGAKGRELKPRSAKGGLVKHDVMHLRKRKERFSFSGILERPVPSLLRGFSAPVTLTSELTRSDLVFLARNDGDEVTRWQALTQLLNAKLTSNSKRVRGGKPATIENSSIKLLGEIAFNPALDEAFRALCLSIPSESDIARELGTNIDPDAILGSRNALISAVAEGHAGKFAELYDGLDHNGPFQPDAASAGRRSLRNVLLDYLSVAEGNPKLAAAQFKHADNMTDRVAALGVLVQRFGGSQATCEALRAFEERFGNDPLVMDKWFIVQAMQPGDEALGKVRGLMAHKLFSLDNPNRTRALIGSFSSGNQTGFNRADGHGYKFFAETVLTIDKKNPQLAARLLTAMRSWRSFEPVRREHAREALATIATVTGLSSDVRDIVERTLAMNEDTALYPPVDPITAGVKARCPRCGQGKLFDGFLKPAERCSSCGLDYSFIDTGEGPAVLVMLLIGFIVVGLALWMEVTLDPPLWLHFILWIPLALVLCLAALRWMKGVLIALQYKHKASEGRLDRPTAHDMSETFDKVADIIAETSEIDRDSITPESHTIDDLGIDSLDFLDIVFAIDKAFGIKIPLEQWTQEVNDGKVPTEEYFVLKNLCAKIDELVAAKSA-MIKMKARASRTSLAMSFAIPLL-MAALPAQAAQSYQTDYRISLYGLSIAKASFSTKVTD-GSYRVTGTLASSGLASLFDDTDGKLDVSGQFANSGPVPNSYVVKYRHGNRNKSTSITFANGNVTNTTNVPPIR-KKKNWVELSTADLLSVADPISGVMVSADSPDHVCGKTLHLYDGQTRVDLKLSPSGKAQPFATKGFKGNAITCSAKFVPIAGYQSGRKAIEYLKNSSQISLTFASLGDSAIYSPVLAKVGTQIGTVTVYATRFEKVQ-------------MEYLSTRGEAPVLGFSDALLAGLARDGGLYLPKEFPQFSASDIRALRGKTYAEVAIHVLTPFVAGDIDQADFERMVHEAYGTFRHEAVCPLVQTRHNEFILELFHGPTLAFKDVAMQLLARLMDHILTKRNERATIVGATSGDTGGAAIEAFANRDRTDIFILFPHNRVSPVQQRQMTTSAASNVHALAIEGNFDDCQSLVKGMFNDLNFRDSLSLSGVNSINWARIMPQIVYYFTAALSLGSPDRAVSFTVPTGNFGDIFAGYVAKRMGLPIDRLVIATNDNDILTRTIETGKYETRGVLHTTSPSMDIQVSSNFERLLFEAHERDAASVRRLMDSLKQSGSFTISDPSLAKIRAEFDAGRSDKLETANTIKDVLGQSGYLLDPHSAIGLKVAR--EKMTS-GTASVVLATAHPAKFPDAVQEASGIHPDLPLWLGNLMQRKEQYTVLPNDLKNVEEYVSRHSRAAR--------MRRFLVRVVFGF-ALALAATGAQAVAPDEMLSNPALEKRARTISAELRCMVCQNESIDDSNADLAKDLRLLVRERLVAGDTDEQVLEFLVARYGEFVLLKPRLQTSTLLLWGFPIAALIAGGVAIVVAIRRRRTAAVEIAPLSESEKQQLKKLFTASRD-------MASAAEQLASNLNFSAFSKAEELKKRIWFTLGALLVYRLGTYIPLPGINLDAFAQAFNNQAQGILGMFNMFAGGAVERMAIFALGIMPYISASIIVQLMTSVVPALEQLKKDGEQGRKVINQYTRYGTVLLATVQAYAIAVGLQGSQGIVTDPGPFFLFSTVITLVGGTMFLMWLGEQITARGIGNGISLIIFSGIVANLPQAISGTLELGRTGALSTPIILAVIVLTIAVIGIIVFVERAQRRLLIQYPKRQVGNRMFQGDTSHLPLKLNTAGVIPPIFASSLLLLPATIAGFAQSHNMPSWATTVLASLGHGQPLYMALYAAMIVFFAFFYTALVFNPKDTADQLKKHSGFIPGIRPGERTAEYIDYVLTRVTVLGAIYLVIICLLPEFLISWAGVPFYLGGTSLLIVVSVTLDTVAQIQGHLIAHQYEGLIKKSKLRGGKRNR
[truncated: 8,050,731 more chars]
