# Supplementary material for: Third Generation Genome Sequencing Reveals That Endobacteria in Nematophagous Fungi Esteya vermicola Contain Multiple Genes Encoding for Nematicidal Proteins
Source: Front Microbiol. 2022 May 3;13:842684. doi: 10.3389/fmicb.2022.842684 (PMC9111515; doi:10.3389/fmicb.2022.842684)
Supplement: Supplementary file 2 [file Data_Sheet_2.docx]

>orf00039

LGETTMKLASKCRQHVAVLLLAVCFSGTAFAQTAGDPTNPETWRTPEYKTQFWLDYIFADYAYAMGVDGSGVKVGVIDSGFARDHPNLPDAMMRVSPSSPTSPGMSIQAR

>orf00041

LPYLIPELERGWLAVTGYDYEYGNQCGVAKNWCLTAPAYSVITVDGRGGYQEINGTSFAAPHVAGAAALVKQMFPYMTMDQVRQVLLGTAFDIGAPGVDDVYGYGLLNAGRAVLGPGKFDWGDFNVKFDGGQSRWFNDITGAGGLVKSGDGALLMFGDSTYAGKTRIDGGTLALAGSIASDTLVGRGGALSGDGIIYGNVDNQGIIYGGWGGAGGTLTIDGNYHQSADASMRVKIGAAEGTSRVDVTGTAQLDGGTVDAFLNPGTFRGDARYTILASGGLTGTFSRVQADYAFLDLTLGYDTANVYLSVLRNKTAFADIGTTKNQRSVGAGVESLGGLVLSGTAGTGNPAASIYDLIIGSNAHDARTIFDSLSGEIHSSVKSALLDESRFARDVISARLRAASGTAPSLSMPLLAYGPGGQIPAAPDASSAVWGQAYGSWGHLSGNANAAKLERSSGGFYMGADGEFGQSWRAGFAGGYGSSSLDAPARASSASVDSYTIAAYAATQVDALALRFWRGPHVAHGGNKPFHGIWCIRSRLHRENGAGFRGGRLCLRLQSCGIGAIRRSRLCKPSHRRHRGKRDCRAQIPRRQ

>orf00848

MMGAFPLALGAPAILLGLLALPVIWWLLRATPPRPVRESFPPLKILAQILKKEETPDRSPWWLTLLRLVLAALVILALAEPVWNPRAETLSGKEPVAIVLDNGWSSNEEWTARKETAERLIADANASGALIYILGTAEKLNADVGPFDAATALERLRALNVRPIPVNRSAAFDRLKAVLANVKGVRVAYLNDGVDTPQAATAIKSMEGSGIASVIWYQPSIAALSAIRQVENNANGLTVHAVRSDDSRASGGITIGAYDEKGRRIAETGLVFRANEATGEAVIAAPYELRNDFHSLRIDNAPHAGATFLVDDNNKRRRVALLSGSEADLSQPLLSPLYYISRALEPFADLLRPRNAELIKAIPELLEQKPSVIIMADIGKLPGETEQAMVDWVAKGGTLIRFAGPRLAGNSEHDPLLPVNLRKGERSLGGALSWKESQPVAAFPDTGPFAGLPTPHDVTVNRQVLAEPAIDLFEKSWANLADGTPLVTGETRGRGQLVLFHVTPEATWSNLPISGSFVDMLHRIVTLSRNTGPSPQDNGAGASRAFPPYLTLNAEGALTPPGADAKPLILKPGETGTVSFDNPPGFYGVEDSMSALNLFKPDSGIKPLVRPELAIPVTTARYAVDESVPLRGPLFGLAALLLALDALAILWLGGHLRRRFKTAAAASLAILIIPAILAFGPSPAHAQQNDSKPGDQTILDAVNATHLAYIITGDRAVDDISKAGLFGLSRALADSTALEPGEPIGVNPETDELAFFPLIYWPVDPAAKMPSQAAIAKIDAYMQQGGTVLFDTRDQDAAAISFDNSSTPANARLRDILSGMNIPALEPVPNDHVLTKAFYILQDFPGRFRGSQLWVQASQAPEANKDRPVRVGDGVSPIMITGNDLAGAWAVNASGAPLLPTIPNDPDQRVLAYRAGINIVMYMLTGNYKSDQVHVPDLLKRLAN

>orf01664

LALIMQRFPYMTNEQALSVLFTTAQNMVASPDVSPTTEQRSRTPVVPGTAQVPNAVTGWGLVDLQKAMNGPGQFLGRFDANWVPVRGTYGATTFRR

>orf01665

LGAGARDIWSNDISQVALDQRKREEAEEVAAWATRKKVQGWENGIGEKQRSELAKIIEPQFSSDKIAAAEALLTTLFKAIGANKSSSAKAVTDANSKITADPLASALLADFKKAYPKSVET

>orf01668

LKVNTTGVTGDLALNGGFASVDGRSGAAVINTGGVLGGIGLVRSFVARNGGTVSPGNSVGTLHVAGDATFDRGSFFDCGDCHGQKPCGPARHWRQCDASGRCGLGAACGRESQREQEQIKDLFAKSYTILIADGTVSGTFENVAAAIHLHHTFPALCY

>orf01669

VLAYDSLSGEAHASLRGTLLQDAGLVSGAASERVRAAFDGVAAKAAPDTTPLAYGPEARDKQSAGEAFAAATPAAPTTALWGQAYGGWSMVRAMQCRGL

>orf01670

MAAGAWFERCNAAAYSRNTGGIVTGVDALIAGTWRWACLPVMARPRCMGRGSSVSADSYQVGIYGGTKIDALTLSLGTILAHHVIDTRRTVAFGTLEETDTAGYSANTVQLFGEAAYRIETPYAALEPFAAAAYTHLKTSGFTETAALPPCRRHHPARTLTTTTLGLRASRAFTLGNATTLTGAAWPAGGMPMAM

>ZGLK1029-RA

MRIVTWALSILGTAAVLAGANANSPGGSIVPGAYIVELADGHELGALYAELKVEGIVVDELQDLRCSAFQGASFQIRNVSADISKDELAAQAQKILDRPSVKNVWPVKMVQHLEPDMATLVDMTFSYTQPLKRRDGADKPKAETKPYGPHVLSQVDRLHAAGYTGKGLRIAIIDTGIDYTHPALGGCFGEGCLVSFGHDFTGELSESHGNQTLDCLGHGTMMTGIIAAQHTEVTDTLQFVGVAPDAQLGMYKVSGCATHPTTDLILAGIAKAVNDRSDVISFSIGRYSGWATDALAVAVSRLVEAGVPIVAPMGNFGTQGLWGMTSPATGHGVIGVGSVDSVTQPLLNERMNYTARGGDGTTDSDGSVAYINGYPPLAGGELPVLFVGENISSSNTACAPLPDNMPNLSGYLVVVGLSNTDGCVPFQQAMHLRAKGAKNIMFYSPTESEVKYDNVMFSFLNNLSGAGVMTMDEARHLVEALDGGRFVSVQMDPPSFPPSEYTIRNSTRTSGLMSAMSGWGPTWEIDLKPQFSTPGGYLLSTTLNGTYAVLSGTSIATAYAAAIVGLVGQARGTMDRDALTAILSSTARPVPWRDGEDIHHDKIAPTVQQGGGVVQALDAVRTPLVVSTAGLSFRDSDQFPGPQTFSIRNVDDTDHDLRLGHLPSVTLYSLQDGLASDSTIVRAPFPPPVAEYAVATLSFDSEEVTVPAGGSVNVTVTLTPPAGIDTARLPIYGGYITLSGTLFNLSVPYVGVVGSLVNTPSIQPGVPGCYLTTTESKRGIAAASNSMFLIPQPNTQTADADNFEFPKVVFSPTLGTSLLRMDLVSASDRPVPGLNTTDFLGMPSLGLLPDMPMRHVSLGRDFLVFNGMLSDSTVVPPGAYRIVVSALNLFGNESNVDDWTIIRTAPFVIEYLQQETTE

>ZGLK1367-RA

MVPVSTLSFLLGLLLATPAVAVPAPAPAPAPASELEARRGGSDRSRLANILGVPINNTQSNRLVTNAYIAVYNKNCTDDDITSHQQEVQAMVAKTNLRRRELSNSSDPVVETTVNAMKINNWHAMCLGQADDQTILSVYNSSMVNYIEQDARVSTFELTEQTGAASGLARLSGNSSADTASKDYTFDTSAGQGITVFVLDSGIRTTHSEFEGRATFGANFADNTNTDGHGHGSHVAGTIGGRTFGVAKKVNLVAVKVLDTNGQGTNSGIIQGMQFVAQNVTAAGTAGKTVVNMSVGGSRSRALNDAINALRNAGVIPVVAAGNDNADATNTSPASATAAITVGAIDQTTDTKASFSNFGSFVDIFAPGVNVKSVGIKSDTDTNVLSGTSMASPHVAGLSAYLMVRDSLTSADAVLSRIQQLATASGASVKNNAGSTTNLIATNGE

>ZGLK1913-RA

MLLLRSLLVLSSVAGSLAARHDPTALHRRANDDDKSLAKNDTDLQSNRFIVEFSGDSDSQAEADGLASRLNLKVVRVFKSNVFQGACLESVEDNLDAIKAEAPVSQAWHAKRVTLAPVLVNQTYGQSAAGVNMSIHHMTGVDKLHEAGIRGKGVTVGIVDTGVWYSHPALGGGIGPGYKITGGYDLVGNTGMSEYPNISRALEFSANSPDWPNDPVRQPDSDPIDQLGHGTHVSGIIAGDSDLFTGVAPDASLRMYKVFSTIDSTTEDVLIEAFLMAYNDGVDIITSSIGGLDGWSDNAWAVVSSRLVDQGVVVTISAGNDGTVGAFSASSGSSGEHVLAVASVDADVKPGPSMNATFAVSGSENNVSIPYMTVADWYPSPINGWPIVPLGFDTTVADEACSPLANSTRNLTNTVVLVRRGGCNFSVKQANIAALGATNVLVYTNESPLVAPTTDYFGGNIAMITREAGAEIIRTIQDGGAVTVDFTTPPEKTLAGAPNEETGGAASYFTSIGATNDLFMKPDIAAPGGYILSTYLDDGYAVLSGTSMACPYVAGIAALYIGQHGGRLTHGAGFGKQLAARILSSGEPIPWNAGTGEVRDYGIPASVAQVGTGMVNATKVLGYDTSLSFSKFALNDTHHFSRYHSVQITNNGADPVTYSFATQEFGGLLTINTDAATWGTPRMAWGDEVLAEPLRGAVPSVSFPRTPFVVPPGETREAEISYSPLDAASLGLNASQLPLYGGTVVIAGSNGESLVVPYQGLAANLHRDMGTVFDYVTGFPTLTSTPNDVPIADKANFTFDLTEADQDYPSLYTRLRYGTRELRWDLFDPAWVERDWVYPPVVGEHGYVGAATSWRGASYTAVFNSTADDPNDVDPLPLTNLPRSVVGEYGVELWWFGRLANGSQIAPGQYKMRIAALLPFSDPTHADNWDVFQTPTITVL

>ZGLK3946-RA

MRGPRLLLSASSMLAAAATPAAAMVGGPGFTSAGQQGVRPAQAPIVPGSYIIEYADDFDSRSKQISHARFYDELATANITRTMDLNFRLFRGASVRFHEQTPAASAEKLLSDMSAIKRVWPVRRYHVPPHTLEWAGNASAVQREKRASLAEDTFSPHVMTQIDRLHAQGITGRGVRVAVVDTGVDYLHPALGGCFGPGCLVSFGYDLVGDAYNGSNAPQPSDSRPPSDCNGHGTHVSGILAAQSGGSSTLGIVGAAPGVELGMFRVFGCHGDVNDDVLIAAFNRAYEAGADVITSSIGGASGWPEEAWSVAVTRIVDSGVPCTISAGNEGSAGLFFSSNAADGHRVTAIASVDNSLAPVLFANATFAVAGRANTSFGYTVGSPANWTDVTLPLWTPTHSTNGNGTNSNGDLEGCSPLGNATNLSGYIVLLRRGTCLFTVKAQNAVAQGARYLLLYNNAPGTVQVDVSTVPGLQGVAMVSNTQGTTWLAALRRGERVDVAMTDPQTAPKTLVNEPNRLSAGFVSMYSSWGPTYEVDVKPQFSAPGGQILSTYPRSAGSYAVLSGTSMACPLAAAAYALVIEARRALPSGGDAGAIERLLASTARPNVFFDGTNSHGYLAPVPQQGAGLIQAYDAAFATTLLSVSSLSFNDSDHRGDGVRNFSLANTGHTSVVYSLSHVPAATAYTFGGDGGGGGSSTAALTPALFPGGLQTTTTAPASLDFGRPDSSGTFDSARLVIPAGQRRIVTVRLTPPAHIDAQRLPVFSGYIAINGSDGSSLSLPYLGVAGSLKSVPVLDVSTSGTYLSETARSPLTNATRGAAGPVPPHHVFLLPPPGEANNSAYANRTTLVQSNIKLLFGSPLLRADLLPLRLCDGASNATTTDVLGYRTIGSVASFPMRWLARDGYNTTWDGALADGTYAPAGLYRLVIRALRVFGDADDPSDYDVTETNPFRIRYARAGKTRREEYLDILCVF

>ZGLK4627-RA

MHYLTLLAVLPLALAAPALKREQPAPLLRPRNMQLVEGKYIVKLKDNLKKGSISTALDLVPSDADHVFNTPGFRGFSLSLDNAGLETLQNHPDVEYIEQDAVVHINTYVSQSGAPWGLKRLSSKGNSSSSYVYDDTAGEGTCAYVIDTGIYTSHSDFGGRATWLANYAGDGTDSDGNGHGTHVAGTIGSTTYGVAKKTKLYAVKVLDSSGSGTNSGVIAGMNFVASDAGNRSCPAGAVANMSLGGGYSAAVNSAAAALITAGVFLAVAAGNSADDASGYSPASEQTVCTVGATTSGDAIASYSNYGSVVDIFAPGTGILSTWIGGSSATNTISGTSMATPHITGLGAYLLALLGKKSPQELCSYIQSSAQTDVLSGIPSDTVNALAFNGNPSA

>ZGLK1483-RA

MGLYYLIQTGAQVFLGAFAIGVDGLSNSTSDDSDEGDEGDEDDEDDDSDLESSSGSDGGECQRLCAFAEYEARTLNHNVAMNGSRGLWDSKNAGAQNKLAERVRARLEKEFGADLESVIQNVTTTGLQPSRQISAKSLEKTTQAFTEMNVALEGDNARAHHLNSTISIRHTSHGIIGLNSTVETKIRTSLEQLYGHLKDIASACSHKVMLQLDGFGTTEPSHATSSHGGKFSLFVSPCPSSDFTKWQETHWKPSKSATSAHADETICTIVEFANEYSQAPVMSFNRSSFRMNPPLKLSFPSANTTVTMRTLLDSNAFTLFAGDGQTAYSWSDRYLLALNLASSLFYLFDGPWGACSWNADNIFFLSSNVNSTTNLVHRRHNPYVCCSLHTETVVGGSLQQRGSLKQLYYPMWLALAEILVELQVGSHMTSRDPASEPRKSSELRNHLRAIVKREMQDGKDSVYLEAVEACLDFGIELVGTAPGPARQKKAQGLISSKIISNLRKSYELWSSTPPERVDLHFQVDKQPENQSFPTPPASPARPSDPARGTDTQVTVPVSPRSQAPMQLHIARVSVPELPSRVSVLRLFDDGGVTSKSDITSYIKFLEKLDLFREQYIDPLKAIRKARKQPAVGVKIAVLDSGVDDVSDPTIKGAINKKRIRSGWSPAGTARENYRDSYGHGTHVTRLLLEIAPFSEIYIAKICDSKDLDESRVQDIVDAINWATKTIDADIITMSFGMKKTIPSVASAISEAVRCNKLVFAAAANEGGLQPRAFPACHPGPASDNFSTVGLGIESVWKGETVYLSGTSFATPVAVGITANILEFAHTWLAESDDQPEKLHSYPTMRALLKGMATERQKYHFISPWDVFPDVPQKTGEKCETSWKEFLDKLRDVIKYQNPSLLSCNKLSYYANWANL

>ZGLK7350-RA

MKHVLALSLAACAYAAPAVNTETIHGDAAPILSSANAQEIPGAYIIKFKDHVNEKTAKDHHSWVQNIHSDWIKTFPEDKDERILELRKRGVFPSADDVFRGLKHTYKIGEGFLGYAGHFDDSVIEQVRRHPDVEYIHKDTIVHTMKYVEIKESKKEDCKPETEKGSPWGLARLSHRKALNFGTFNKYLYSDDGGEGVDAYVIDTGTSIKHVDFEGRAHWGKTVPSGDEDADGNGHGTHCSGTIAGKKYGVAKKANVYAVKVLRSNGSGTMSDVIAGVEFAAQRHLEQVASAKAGKRKGFKGSVANMSLGGGKTTPLDAAVNAAVSAGLHFAVAAGNDNADACNYSPAAADKALTVGASAIDDSRAYFSNWGKCTDVFAPGLNIQSTWIGSNTAINTISGTSMASPHIAGLLAYYLSLQPAGDSEYSVAPITPEKLKSNLLAVATVGALTDLPADTPNLLAWNGGGCSNYTAIVEHGSYKTSPSVSEADDAESRVLTSVSKLEKAIELDLEIISGKVQTGASAFGKKAAKFSKKIHELVDEELEEFLEELTA

>orf00208-pz

MSFDATAAAISARTLAPFRKRLNSTALTALLLAATATGASADGRYFMAPLVPGGPMQMTDDLEKAAASWVTTEFTDTGVLSKLLAQYAYAFGARGQGARIGVMDGGLWTGHDEFTRQKIENIHSEGKFEITLHNAGGKPFAKAGDSFSVDGLWYGPTDLVPLMDRHPQFTAGTILARRNGTGSHGIAFDSTIYVAQSGELIYKDEQGSLDNNVIEAKEKDPELFRQSMQELVKAGAQVILLEMQLLPKPRNADTQGWFVDLMRQYAGGKGGTLLTAMEDAAKAGVVHVVAAGNYNPDTPWIAAALPVFRPELEKSWIAVVEDETKSNPCGPARFFCIAGYSNIYAPTTEADEYDIYTGTSGASAVTAASMGVLLSRYPYLQATAVRDILLTTAKDIGDPGVDAKTGWGLIDLKKSMSGPAQFLGRFEANLPAGLSDTWSNAISQDVLSQRRQEDSKEISDWDKKKEERGWQSGVTEAQTKINLVKDFGGSRLVAAEDLIKAVLKLTPPGTSISALGQLISAQEVLKADPIATGIWKGFTAKYTKWSSSKTALADYTSFRASAEGANNVIIDAILSDRVVADTMENEITQTRINAHPRPTMPA

>orf02284-pz

LKDPSKLGPSSTLITNSQGLAEIATTYGNDVITNAEENKIAPAASSTPVTSDGAITLNLDKPVATVQTPKPGTVVVYEDGKYTFDKKISGNVSLVKTGLGGLILSGDNDYTGDTHIAQGQLAVRGSVQNLTVDKDSAIVQTQADGGWNVRGDATFIPGSQLQIIAKPDGTLNPLKINGKLAITGSTLFHIHAADETIPTREGQIALFTQDHTIIRASKGWRLFNSAVVNEARISGPFLVMPINSKPYLRTLAYAYPSDGSLVITMKKNN

>orf05793-pz

MPPQSENTKPPAGYYYLWHLAALRVIDADFGPLTRPLPSNNESALRNAANPVITGSVWDTIAELGAVSPAKVALIDVGISPDHPNLTTRIDHNASIDLATHRYGARALEILNDTNSSYKEEKQAFFSGLDISGLGNLGLSDDDKEYLDDIVAEYAASNGVQRRLLDSNTMFASHGTACAGLIVGEPAALPSGDSENPSPPEYLLTNPDSTEAHPNKNPNLLPYFGADPFSRLVNITTSFEEDARQFIAAFLYAHLQNVDVIVMPRGIPDPKRSAVEPKNELKADLERWANRDAADLFARIAVAEQGPSELEPKAPQTGSNPDRLWKILKQLIIGVSRKIPVICAAGNSGESQLIYPASLAAPDNGIVAVGAVTVEGFRSCYSNYGEGLTLVAPSDDSEVFNRHQLRVDRLSPFAAKHQYSADGGKEYCYSYFSLLSTDLPGVFGYDEGKAPWSSLLPSANNPGVGGGYYTSFGGTSGAAALVGGVAALMQRTHRAVHGGTGKLNGVAVKNILQEASNLNSNVEPGLRPLTPDCMNADNEDVIDPSYFFGAGLLNAATAVNAVLTS

>orf00209-pz

LRLTGQNTYTGDTIINGGLLAVDGSITSKAIINDSGILGGIGSVGSLVANTGGRVSPGNSVGKLTVTGDATFEKGSIFDVEIAADAGAADQLDVTGKVALLGGAVQARIEGETAFFTKDQVVGLFTRSYDILLAGKGIEGTFETVRPQYNYISAVLDYSHKNKVTLGFMLTPDPVLVVDPEIGAPVGAVVDEFVFEPELESVVEDAERAGLARTEAERLKLEALRERVRTLVLVDAKTKNQKNAGDAIKQMDIGDPLLNTVLFSQVGQVLPYDNLTGEVHATLDGVLIEDSHFISTAATDRIRAAFDGVAAKAQPVIAPLAYGAPVKAKGSESFGTFDLAGGSTGSVPAPAATALWGEAYGAFARGISDGNASRYSRSTGGFVTGLDGVVAETWRFGLLAGYGSSSLNGNGRALVDSYQIGLYGGTTWDSLGLRFGANLGHHEIETKRIAIFGGLANEHEASYDAKTVQVFGEIGYEIKTAYAELEPFAGVSHVHLKTDAFEETGDISNLSGEASTTDLTTTTLGLRVSRDFALSESVNVTARGTLGWRHAYGDVTPQQRLAFAGDQAFSVEGLPVAQDTGFVEAGLDLGIGRNTTLGISYSGQFSKSASDNAVKADLTVRF

>orf00674-pm

MKLASKCRQHVAVLFLSVCFSGTAFAQTAGDPTDPETWRTPEYKAQFWLDYILANYAYAMGVDGSGVKVGVIDSGFARDHPEFAGRYDEGITVQPDKPWYVDSSSVTHGSAVAGVIAANRDGKGMHGVAPGATIVAVNAEEEDGYINTKAAIAGIYGLVSRNVHIINNSYGLEAAITDYAPDVISSRYKAEIAAYRHAVANDTLLIWGTGNDSRSQPSMNAGLPYLIPELERGWLAVTGYDYEYGNQCGVAKNWCLAAPADAVMTVDGRGGYQEISGTSFAAPHVAGAAALVKQMFPYMTMDQVRQVLLGTAFDIGAPGVDDVYGYGLLDAGRAVLGPGKFDWGDFNVKFDGGQSRWFNDITGAGGLVKSGDGALLMFGDSTYAGKTRIDGGTLALAGSIASDTLVGRGGALSGDGIIYGNVDNQGIVYGGWGGAGGTLTIDGNYHQSVDASMRVKIGAAEGTSRVDVTGAAQLDGGTVDAFLNPGTFRGDARYTILASGGLTGTFSRVQADYAFLDLTLGYDATNVYLSVLRNKTAFADIGTTKNQRSVGAAMQSLGGLVLSGTAGTDNPAASIYDLIIGSNAHDARTIFDSLSGEIHSSVKSALLDESRFARDVISTRLRAASGDGAPASMPLLAYGPGGQIPAAPDASSAVWGQAYGSWGHLSGDANAVKLERSSGGFYMGADGEFGQSWRAGFAGGYGSSTLDAPARASSASVDSYTIAAYAGTKADALALRFGAAHTWHKVETSRSTAFGASAADYDARTVQVFGEAGYAFQYNRMALEPFANLAYVNLHTGNIGESGTAGLKSRSDNDDNVFATLGLRARTDIPVGDAAKLTVRGMLGWQHAFGDVSPGTIATLAGGAPFQVEGSPLARDALIAEAGLDFSVTKTVTLGVSYTGQMSSAVQAHGVRGDLSWRF
