# Supplementary material for: Third Generation Genome Sequencing Reveals That Endobacteria in Nematophagous Fungi Esteya vermicola Contain Multiple Genes Encoding for Nematicidal Proteins
Source: Front Microbiol. 2022 May 3;13:842684. doi: 10.3389/fmicb.2022.842684 (PMC9111515; doi:10.3389/fmicb.2022.842684)
Supplement: Supplementary file 4 [file Data_Sheet_4.docx]

**Third generation genome sequencing reveals that endobacteria in biocontrol fungi *Esteya vermicola* contain multiple nematicidal genes**

**Supplementary Figures**


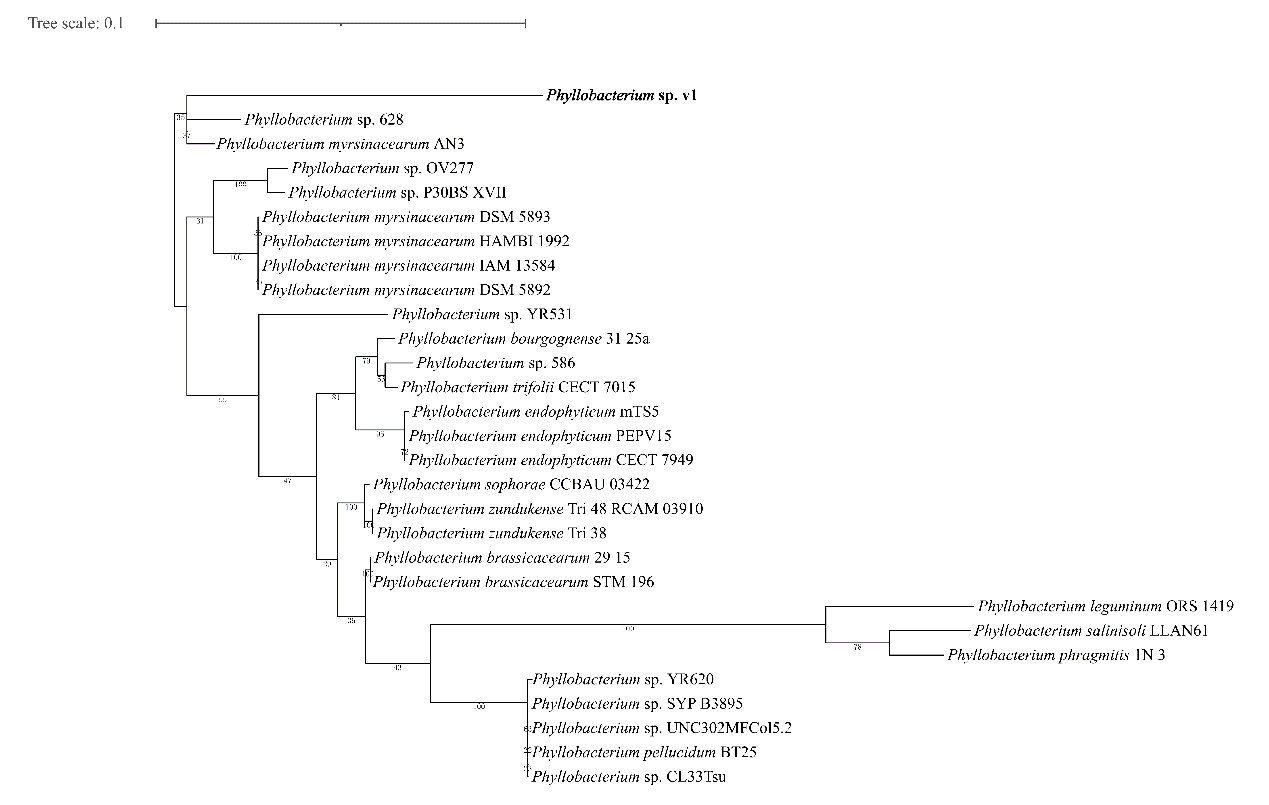


Figure S1 Phylogenetic placement of the endobacteria inferred by maximum likelihood phylogenetic analysis based on conserved atpD and recA.
